# Biomimetic total synthesis of the reported structure of (+)-selaginedorffone B†‡

**DOI:** 10.1039/d4sc04103h

**Published:** 2024-07-25

**Authors:** Sourav Kundu, Debgopal Jana, Nilangshu Mandal, Ayan Mondal, Ranjit Murmu, Nanda Kishore Roy, Ayan Datta, Alakesh Bisai

**Affiliations:** a Department of Chemistry, Indian Institute of Science Education and Research Bhopal Bhopal Bypass Road Bhopal 462 066 Madhya Pradesh India alakesh@iiserkol.ac.in alakeshb@gmail.com; b Department of Chemistry, Indian Institute of Science Education and Research Kolkata Mohanpur Campus, Nadia Kalyani 741 246 West Bengal India; c School of Chemical Sciences, Indian Association for the Cultivation of Science 2A and 2B Raja S. C. Mullick Road, Jadavpur 700032 Kolkata West Bengal India spad@iacs.res.in

## Abstract

The first enantioselective total synthesis of the reported structure of the structurally unique aromatic tetraterpenoid of anti-cancer potential, (+)-selaginedorffone B (2), has been accomplished from two modified abietane diterpenoids through an intermolecular Diels–Alder reaction between a bio-inspired diene 3 (HOMO counterpart) and dienophile 4 (corresponding LUMO counterpart) in a 23-step sequence, whereas the core framework of the monomeric abietane diterpenoid was constructed *via* alkyne-activated ene-cyclization. Computational analysis was conducted to reveal the intricate regio and diastereoselectivity of this novel Diels–Alder reaction, strengthening the experimental results. The absolute configuration of the synthesized molecule was validated through X-ray studies of late-stage intermediates as well as comprehensive 2D NMR analysis.

## Abstract 2

The first catalytic enantioselective total synthesis of (+)-selaginedorffone B (2) on a gram scale has been accomplished from two modified abietane diterpenoids, diene 3 and dienophile 4, through a key intermolecular Diels–Alder reaction.

## Graphical abstract

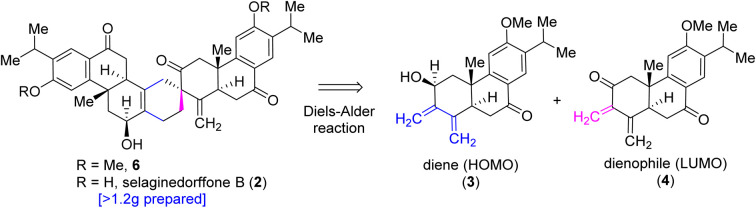

## Introduction

**Fig. 1:**
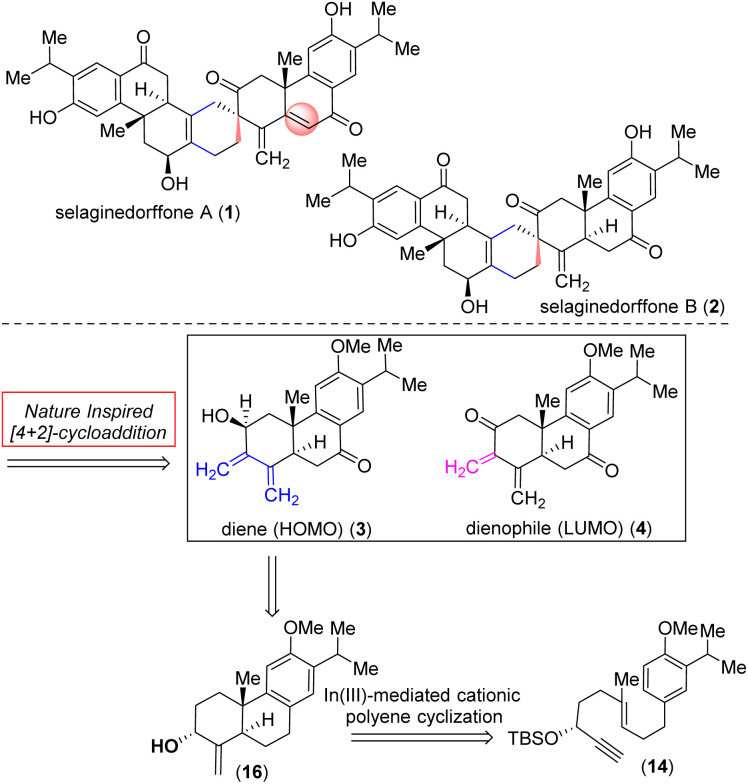
Naturally occurring tetraterpenoids, selaginedorffones A (1) and B (2), and retrosynthetic analysis.

*Selaginella moellendorffii* Hieron (Selaginellaceae) is a perennial herb widely spread across mainland China, Japan, the Philippines, and Vietnam^1^ with a rich history as traditional folk medicine and has been employed to address conditions such as bleeding, gonorrhea, jaundice, and idiopathic thrombocytopenic purpura (ITP).^2,3^ Recently, two novel heptacyclic tetraterpenoids sharing an unusual functionalized spiro[5.5]undecane motif, selaginedorffones A (1) and B (2) (Fig. 1),^4^ were obtained from the methanolic extract of *Selaginella moellendorffii*. The structures of 1–2 were identified by a combination of detailed NMR spectroscopic analysis and ECD calculations.^4^

To ascertain the growth-inhibitory effects of these two dimeric abietane diterpenoids, their cytotoxic potential was evaluated against a range of human cancer cell lines, including SW480 (rectal cancer), HL-60 (leukaemia), SMMC-7721 (liver cancer), MCF-7 (breast cancer), and A-549 (lung cancer).^4^

These studies revealed that (+)-selaginedorffone A (1) had no activity against all five examined cancer cell lines, but the latter, selaginedorffone B (2), shows significant selectivity in its cytotoxic activities against MCF-7 cells with a notable IC_50_ value of 9.0 μM.^4^ The emerging biological activity and the intricate molecular architecture of these complex tetraterpenoids have drawn considerable attention from the synthetic community. The functionalized spiro[5.5]undecane motif of (+)-selaginedorffones A (1) and B (2) was biosynthetically constructed from two modified abietane diterpenoids through an intermolecular Diels–Alder reaction (Fig. 1) between a modified abietane, diene-ol 3 (HOMO counterpart) and dienone 4 (corresponding LUMO counterpart).

**Scheme 1:**
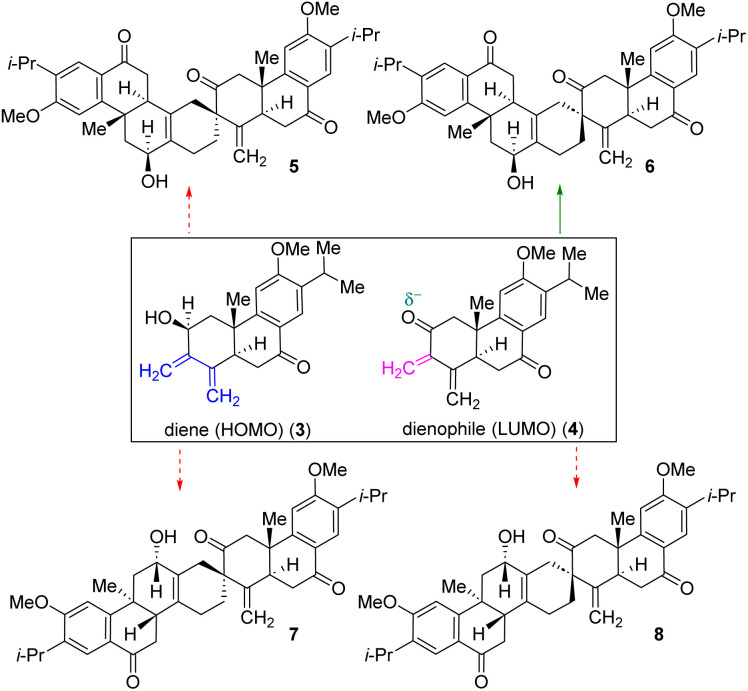
Plausible outcome of Diels–Alder adducts from diene 3 (HOMO counterpart) and dienophile 4 (corresponding LUMO counterpart).

**Fig. 2:**
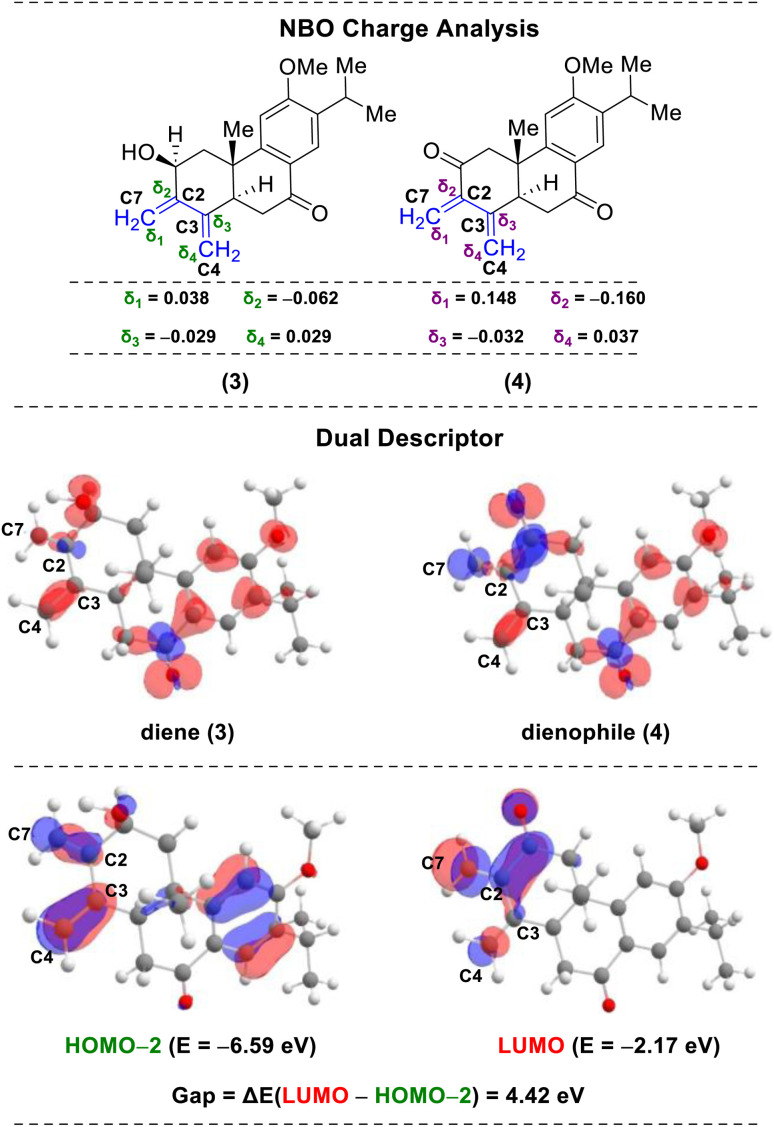
NBO charge analyses, dual descriptor plots, and HOMO–LUMO energies for molecules 3 and 4 at the B3LYP(GD3BJ)/6-31G(d) level of theory.

**Fig. 3:**
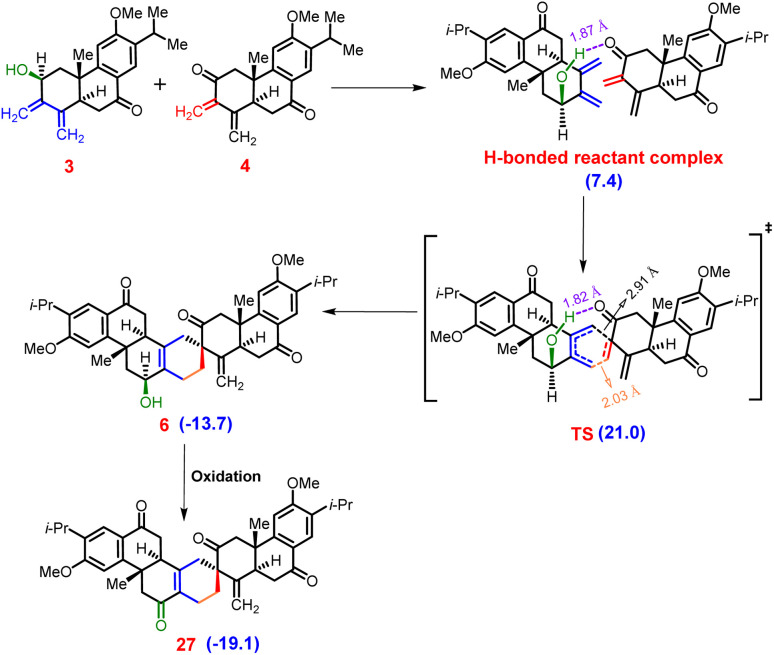
[4 + 2]-Cycloaddition pathway between diene (3) and dienophile (4), with relative free energies (in kcal mol^−1^) calculated using the SMD(DCM)-B3LYP(GD3BJ)/6-311+G(d,p)//B3LYP(GD3BJ)/6-31G(d) level of theory at 25 °C.

Nature uses pericyclic reactions in the biosynthesis of secondary metabolites for the construction of complex molecular architectures.^5,6^ Herein, we describe the first total synthesis of the most potent member of this class of tetraterpenoid, (+)-selaginedorffone B (2), through a bio-inspired Diels–Alder reaction (Fig. 1).^7,8^ The regio- and diastereoselective formation of a predominant single D–A adduct among various potential isomers (Scheme 1) may be directly influenced by secondary orbital interactions, sterics, and electronic factors.^9,10^ This underscores the significance of computational analysis (Fig. 2 and 3) in elucidating the complex regio- and stereoselectivity of this innovative Diels–Alder reaction, thereby fortifying the experimental findings. Therefore, investigating the establishment of this selectivity in this Diels–Alder reaction^11–13^ to validate the biosynthesis of (+)-selaginedorffone B (2) holds immense significance.

Our efforts began with identifying a practical synthesis of modified abietane diterpenoids such as diene-ol 3 sharing a highly functionalized *trans*-decalin system. Towards this, it was envisioned to synthesize an abietane skeleton followed by peripheral functionalization around the *trans*-decalin motif. Prior elegant approaches to catalytic asymmetric synthesis of monomeric abietanes were independently developed by Loh (SnCl_4_-mediated polyene cyclization in the presence of chiral acetal),^14^ Corey (catalytic alkyne activation by In(iii) followed by cyclization^15^ and SbCl_5_-mediated polyene cyclization),^16^ Carreira [Ir(i)-catalyzed enantioselective cyclization],^17,18^ Baran^19^ (epoxide-initiated polyene cyclization), Krische (TiCl_4_-promoted Friedel–Crafts type alkylation/cyclization),^20^ Carter (utilizing Pummerer rearrangement),^21^ Li (In(iii)-catalyzed cationic polyene cyclization reaction)^22^ and others.^23^

**Scheme 2:**
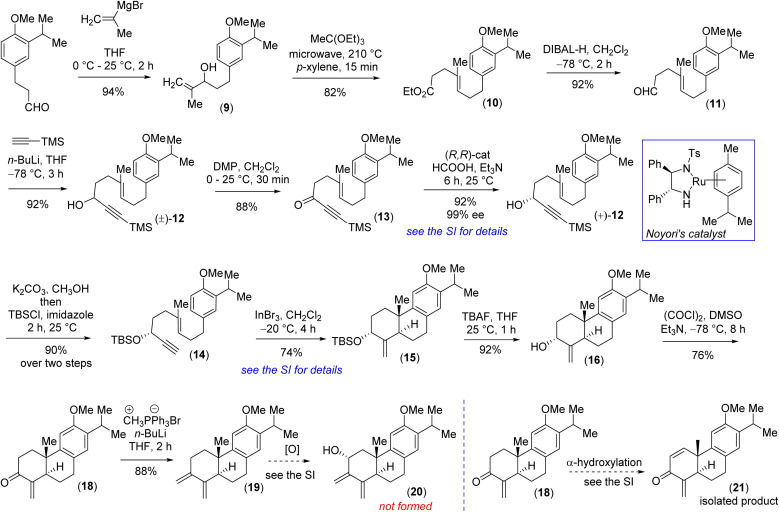
Synthesis of the abietane core.

Retrosynthetically, we imagined accessing the highly functionalized abietane scaffold starting from enantiopure allylic alcohol 16 that in turn can be accessed from In(iii)-catalyzed alkyne-activated ene-cyclization of 14 (Fig. 1) inspired by the pioneering work by Corey *et al.*^15^ The choice of In(iii) is attributed to its potential for enhanced overlap with the orthogonal π-orbitals of the C–C triple bond, facilitated by mixing a 5p orbital with the 5s orbital, which is advantageous due to the smaller energy gap between these orbitals compared to metals of lower atomic numbers. Starting with the literature known compound 3-(3-isopropyl 4-methoxy)-propionaldehyde was converted to allylic alcohol 9 with isopropenylmagnesium bromide in 94% yield. The Jhonson–(orthoester)Claisen rearrangement of 9 followed by DIBAL-H reduction furnished aldehyde 11 in 75% yield over two steps (Scheme 2). Next, TMS-acetylene addition of 12 followed by DMP oxidation afforded ynone 13 in 81% yield over two steps. Gratifyingly, Noyori's reduction^22^ using a 1 mol% catalyst (see the ESI‡ for details) furnished propargyl alcohol (+)-12 in 92% yield with 99% ee (Fig. 3). Later, desilylation of (+)-12 by treatment of K_2_CO_3_ in methanol followed by TBS-protection with *tert*-butyldimethylsilyl chloride afforded 14 in 90% over two steps. At this stage In(iii)-catalyzed alkyne-activated ene-cyclization of 14,^15^ in the presence of 20 mol% InBr_3_ furnished carbotricycle having an exocyclic double bond, 15 in 74% yield (see the ESI‡ for details). Next, Swern oxidation of allyl alcohol 16 furnished α,β-unsaturated ketone 18 in 76% yield (see the ESI‡ for details),^24^ which upon Wittig olefination furnished 1,3-butadiene 19 in 88% yield (Scheme 2).

**Scheme 3:**
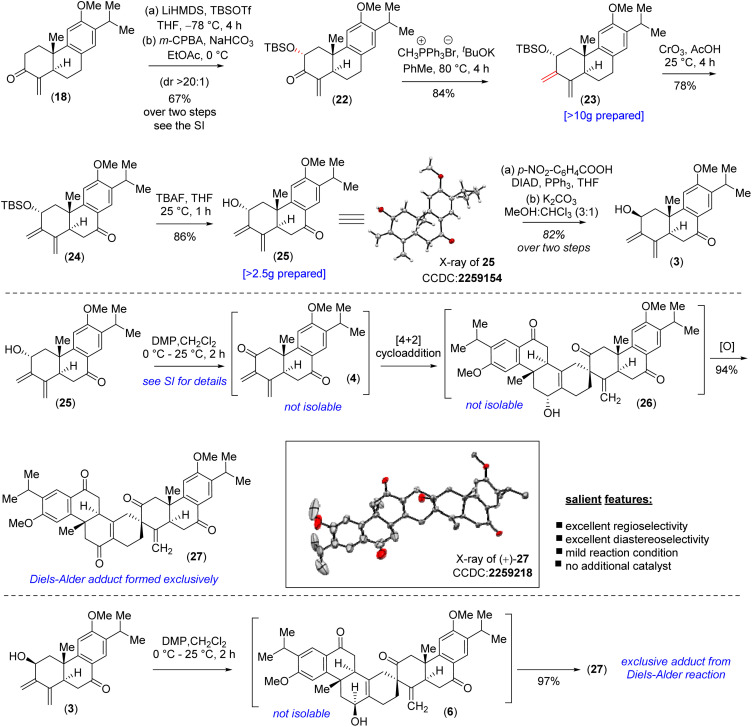
Diels–Alder reaction under oxidative conditions.

At this stage, all attempts to perform the allylic oxidation to access compound 20 under various oxidation conditions were unsuccessful (see the ESI‡ for detailed information). Also, attempts for direct α-oxygenation of α,β-unsaturated ketone 18 under various conditions only furnished dienone 21 in 72% yield^25^ (Scheme 2), without the isolation of the required α-hydroxy enone (see the ESI‡ for details). Next, enone 18 was converted to C_α_-silyloxy enone derivative 22 in two steps (>20 : 1 dr) *via* a modified Rubottom oxidation^26^ (Scheme 3), where the additives found to play an important role (see the ESI‡ for details).^27^

Furthermore, Wittig olefination of the silyloxy enone 22 provided us with the ‘*S-cis*’ 1,3-butadiene 23 in 84% yield.^28^ With the silyloxy butadiene compound, we performed benzylic oxidation with CrO_3_ in acetic acid to afford 24 (78% yield).^29^ Next, TBAF mediated desilylation of compound 24 afforded the electron-rich diene-ol partner 25 in 86% yield (Scheme 3). The X-ray structure of diene-ol 25 (CCDC 2259154) showed the formation of epimeric allylic alcohol of diene-ol 3 (Fig. 1). With diene-ol 25 in hand, we turned our attention to the synthesis of diene-ol 3 (HOMO counterpart) and dienone 4 (corresponding LUMO counterpart) for the key bio-inspired Diels–Alder reaction. Thus, a Mitsunobu inversion of the stereogenic center of the hydroxy group of 25 with *p*-nitrobenzoic acid^30^ followed by saponification afforded diene-ol 3 (HOMO partner) in 82% yield over two steps (Scheme 3). However, serendipitously, attempts to synthesize dienone 4 from a DMP (Dess–Martin periodinane) oxidation (1.1 equivalents) of the diene-ol 25 afforded the heptacyclic enone 27 in 94% yield, without the formation of any trace of neither enone 4 nor allyl alcohol 26 (a clean spot-to-spot reaction on TLC was observed; Scheme 3). To our great satisfaction, X-ray analysis of heptacyclic enone 27 (CCDC 2259218) unequivocally confirmed the formation of all five stereogenic centers required for (+)-selaginedorffone B (2). Interestingly, similar results were obtained under the well-known Ley–Griffith oxidation (TPAP) and heptacyclic enone 27 was isolated in 91% yield (see the ESI‡ for details).^31^

However, the biogenetic hypothesis of selaginedorffone B (2) suggests that the electron rich diene partner should contain the hydroxy group ‘*syn*’ with respect to the angular methyl group. To our pleasant surprise, exactly a similar result was obtained when DMP oxidation (1.1 equivalents) was carried out by taking the diene-ol 3 at 25 °C for 2 h (97% yield) (Scheme 3). From this serendipitous discovery, it can be considered that as soon as the formation of dienone 4 has taken place, it immediately reacted with diene-ol (either 25 or 3), by establishing the Curtin–Hammett conditions, and underwent the 2^nd^ level of oxidation of the adduct (either 26 or 6) to form 27. Several control reactions were performed by changing the equivalents of the oxidizing agent to stop the reaction at the heptacyclic allyl alcohol stage (either 26 or 6). However, we were not successful in this regard. By lowering the oxidizing agent to 0.5 equivalent, we could see the formation of only 27 (∼43–46% yields) in addition to the starting diene-ol 25 or 3 (∼44–48% yields), respectively (see the ESI‡ for detailed studies). At this stage what was left was to perform a chemoselective reduction of α,β-unsaturated enone of 27 over three keto functional groups. However, attempts towards this direction *via* reduction under Luche conditions led to the formation of an inseparable mixture of alcohol/allylic alcohols.

**Scheme 4:**
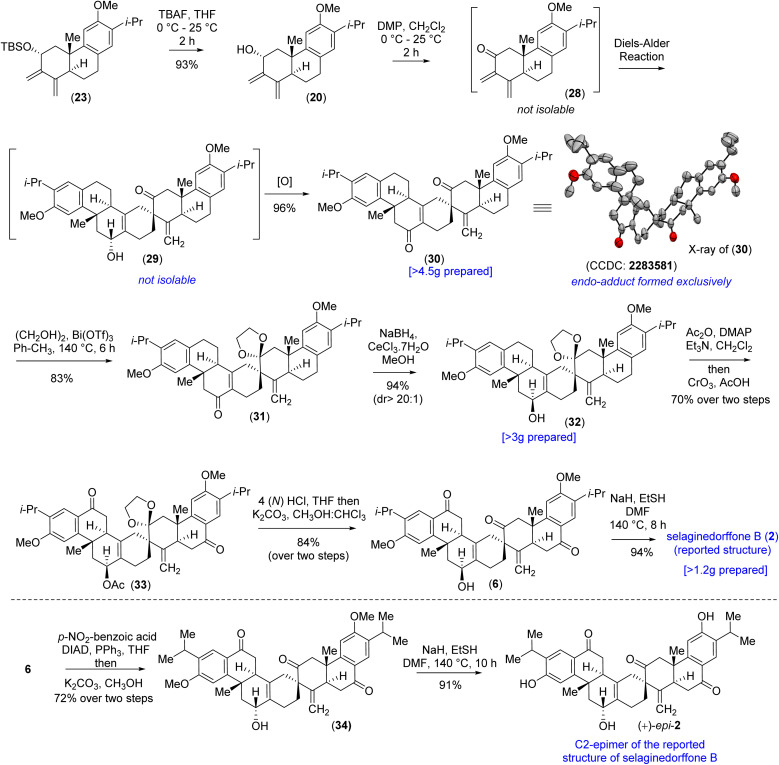
Total synthesis of selaginedorffone B (2).

Also, chemoselective protection of ketones over the enone 27 proved to be difficult; therefore, an alternate route was inevitable at this point. Thus, it was thought of performing the Diels–Alder reaction with diene-ol 20 that avoids benzylic ketone, which was synthesized *via* the desilylation of TBS-ether 23 (Scheme 4). As per our optimized conditions, the DMP oxidation (1.1 equivalents) was carried out by taking the diene-ol 20 under standard conditions (25 °C for 2 h) to furnish 96% yield of enone 30*via* the non-isolable intermediates dienone 28 and allyl alcohol 29 (a clean spot-to-spot reaction on TLC was observed; Scheme 4). Furthermore, X-ray analysis of heptacyclic enone 30 (CCDC 2283581) unambiguously confirmed the required stereogenic centers present in the heptacyclic skeleton of (+)-2 (Scheme 4). Next, an attempt to differentiate the enone over the ketone in 30 under the Luche reduction in MeOH was conducted. To our surprise, this reaction led to a mixture of inseparable diastereomeric alcohols (2 : 1) forming from the reduction of ketone, keeping the enone intact (see the ESI‡ for details). After an exhaustive optimization ketal protection of ketone 30, in the presence of Bi(OTf)_3_, refluxing toluene afforded compound 31 in 83% yield.^32^ Next, Luche reduction of the enone 31 at 0 °C provided allyl alcohol 32 in 94% yield with >20 : 1 dr (Scheme 4). Furthermore, acetylation of allyl alcohol 32 followed by benzylic oxidation afforded diketone 33 in 70% yield over two steps. Later, ketal deprotection using 4(N) HCl followed by the saponification of acetate cleanly furnished methyl ether of (+)-2*i.e.* compound 6 in 84% yield over two steps (Scheme 4). Finally, demethylation with sodium ethanethiolate^33^ of the aryl methyl ether 6 at elevated temperature afforded the reported structure of the tetraterpenoid, (+)-selaginedorffone B [(+)-2] (Scheme 4), in 94% yield. Unfortunately, NMR spectra of the synthetic one, (+)-2, exhibited notable discrepancies compared to those reported by Long *et al.*^4^ Thus, we attempted to synthesize the epimer of the hydroxy group of final allyl alcohol 2 to compare its spectral data with the isolation report by Long *et al.*

Accordingly, compound 6 was charged for a sequential Mitsunobu reaction^30^ followed by K_2_CO_3_-mediated hydrolysis of the benzoate derivative, yielding the epimeric allyl alcohol 34, with a 72% yield over two steps. Subsequently, like the previous procedure, the aryl methyl ether was demethylated with sodium ethanethiolate to synthesize the C_2_-epimer of the reported structure of selaginedorffone B (+)-2 (Scheme 4).^33^ Regrettably, discrepancies were once again encountered in this process. However, the X-ray crystallographic studies of intermediate compounds 27 (CCDC 2259218) and 30 (CCDC 2283581), which are just a few steps preceding the final product, along with the thorough 2D NMR analysis, strongly suggest that the structure of the synthetic sample is unequivocally matching the reported structure by Long *et al.*^4^

To unveil the intriguing dynamics of the regiocontrol reaction between termini 3 and 4, sharing close steric and electronic characteristics, we have bolstered our experimental findings through computational analysis (see the ESI‡ for computational details). In Fig. 2, we depicted the sum of hydrogen atom charges for both 3 and 4 utilizing natural bonding orbital (NBO) analysis.^34^ Notably, the highest positive charge was observed on the C7-atom in molecule 4, indicating the potential of the C2–C7 double bond (highlighted in red) to act as a dienophile, while the C7–C2 and C3–C4 double bonds (highlighted in blue) in molecule 3 can serve as the diene. To further validate our primary diene–dienophile partners, we have employed dual descriptor characteristics to discern reactivity sites within molecules 3 and 4.^35,36^

Favourable interactions between molecules occur when electrophilic regions (Δ*f*(*r*) > 0, indicated in red on the isodensity maps) align with nucleophilic regions (Δ*f*(*r*) < 0, indicated in blue on the isodensity maps) and *vice versa*. In our investigation, the C7-atom exhibited a blue-colored lobe (using iso-value = 0.001) in 4, justifying its nucleophilic reactivity. Conversely, the C7 and C4 centers in molecule 3 displayed red-colored lobes (using iso-value = 0.001), indicating their electrophilic nature. Hence, favorable interaction between these opposite regions is obtained and deemed reliable. Additionally, we endeavoured to compute the highest occupied molecular orbital (HOMO) and lowest unoccupied molecular orbital (LUMO) for molecules 3 and 4 (Fig. 2). Our analysis of the energy levels and orbital coefficients revealed that the interaction between HOMO − 2 of molecule 3 and the LUMO of molecule 4 favours the Diels–Alder (D–A) reaction (see Fig. S4 and S5 in the ESI‡ for detailed insights).^37,38^ Furthermore, the energy gap between HOMO − 2 and the LUMO is small, measuring at 4.42 eV. Hence, molecule 3 functions as the diene, while molecule 4 serves as the dienophile.

In our pursuit to unravel the intricate mechanics of the reaction, we meticulously computed the entirety of the reaction pathway alongside the associated relative free energies (Fig. 3). The [4 + 2]-mode of the Diels–Alder reaction unfolds between the diene (3) and dienophile (4), instigating the formation of an initial hydrogen bond complex. This initial stage sets the foundation for subsequent molecular interactions.

As the reaction progresses, the hydrogen bond undergoes a progressive strengthening process at the asynchronous transition state (TS) for the [4 + 2]-mode.^39,40^ This strengthening is a result of a delicate interplay between the cooperative effects of hydrogen bonding and secondary orbital interactions. Notably, this synergistic collaboration facilitates the traversal of a relatively low energy barrier at the asynchronous TS, quantified as Δ*G*^‡^ = 21.0 kcal mol^−1^. This modest barrier signifies the readiness of the system to progress from reactants (3 and 4) to product 6 (see Fig. S6 and S7 in the ESI‡ for details). Upon surmounting the TS, the reaction proceeds along an exergonic pathway, leading to the formation of product 6.

This intermediate product represents a critical milestone in the reaction cascade. Subsequently, product 6 underwent an oxidation step, culminating in the generation of stable product 27. This final product exhibits a notable decrease in free energy, measured at −19.1 kcal mol^−1^ relative to the free reactants 3 and 4. In essence, our comprehensive analysis provides a detailed portrayal of the intricate interplay of molecular events that govern the progression of the [4 + 2]-mode addition reaction, shedding light on the underlying mechanisms driving this fundamental chemical transformation.

In conclusion, we have accomplished the first catalytic enantioselective gram-scale total synthesis of the reported structure of (+)-selaginedorffone B (2) in 23 LLS. The pivotal steps for synthesizing the rearranged abietane monomer include asymmetric transfer hydrogenation with Noyori's protocol and the In(iii)-catalyzed alkyne–ene cyclization reaction, followed by a series of classical reactions such as Riley oxidation, Swern oxidation, Rubottom oxidation, DMP oxidation (Diels–Alder reactions), ketal protection–deprotection, Luche reduction *etc.* as key steps. Our synthesis demonstrates a unique Diels–Alder reaction under ambient conditions that creates an intricate and complex molecular scaffold, thereby validating the biosynthesis of (+)-selaginedorffone B. Our thorough computational studies unveil the complex molecular interactions driving the progression of the [4 + 2]-addition reaction, offering insight into the fundamental mechanisms at play in this chemical transformation. The unambiguous determination of the structures of intermediate compounds 27 (CCDC 2259218) and 30 (CCDC 2283581) in the synthetic route through X-ray crystallography, which are only a few steps away from the final product, as well as comprehensive 2D NMR analysis, strongly suggests that the structure of the synthetic sample matches with the reported structure by Long *et al.*

## Data availability

Experimental details and spectral analysis are available free of charge from the ESI‡ available with this article. CCDC 2259154,^41^ 2259218,^42^ and 2283581^43^ contain the supplementary crystallographic data discussed in this paper. These data can be obtained free of charge *via*https://www.ccdc.cam.ac.uk/data_request/cif,orbyemailingdata_request@ccdc.cam.ac.uk.

## Author contributions

A. B. conceived and supervised this project. A. D. has supervised the DFT calculation part. S. K. investigated the key Diels–Alder cycloaddition leading to selaginedorffone B and D. J., A. M., R. M., and N. K. R. synthesized the starting materials. N. M. investigated the DFT calculations. A. B. and S. K. wrote the original draft of the manuscript which was edited by all authors.

## Conflicts of interest

There are no conflicts to declare.

## Supplementary Material

SC-OLF-D4SC04103H-s001

SC-OLF-D4SC04103H-s002
