# Supplementary material for: Biomimetic total synthesis of the reported structure of (+)-selaginedorffone B
Source: Chem Sci. 2024 Jul 25;15(36):14946–53. doi: 10.1039/d4sc04103h (PMC11376052; doi:10.1039/d4sc04103h)
Supplement: SC-015-D4SC04103H-s001 [file SC-015-D4SC04103H-s001.pdf]

Supplementary Information (SI) for Chemical Science.
This journal is © The Royal Society of Chemistry 2024

Spectral Data

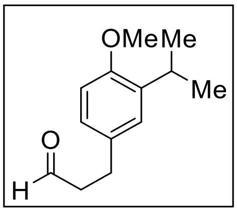

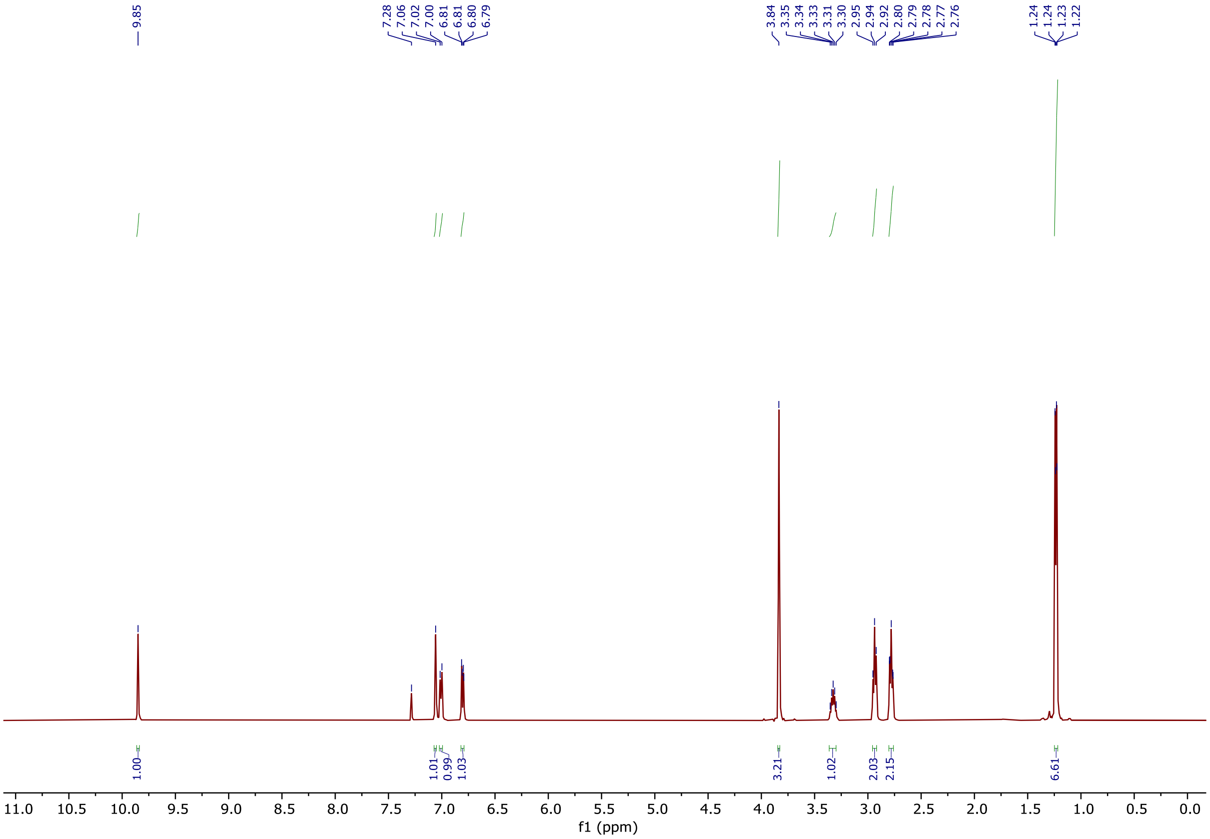

^{1}H NMR (500 MHz, $CDCl_3$) of 3-(3-isopropyl-4-methoxyphenyl)propanal

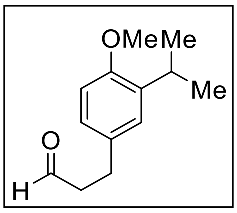

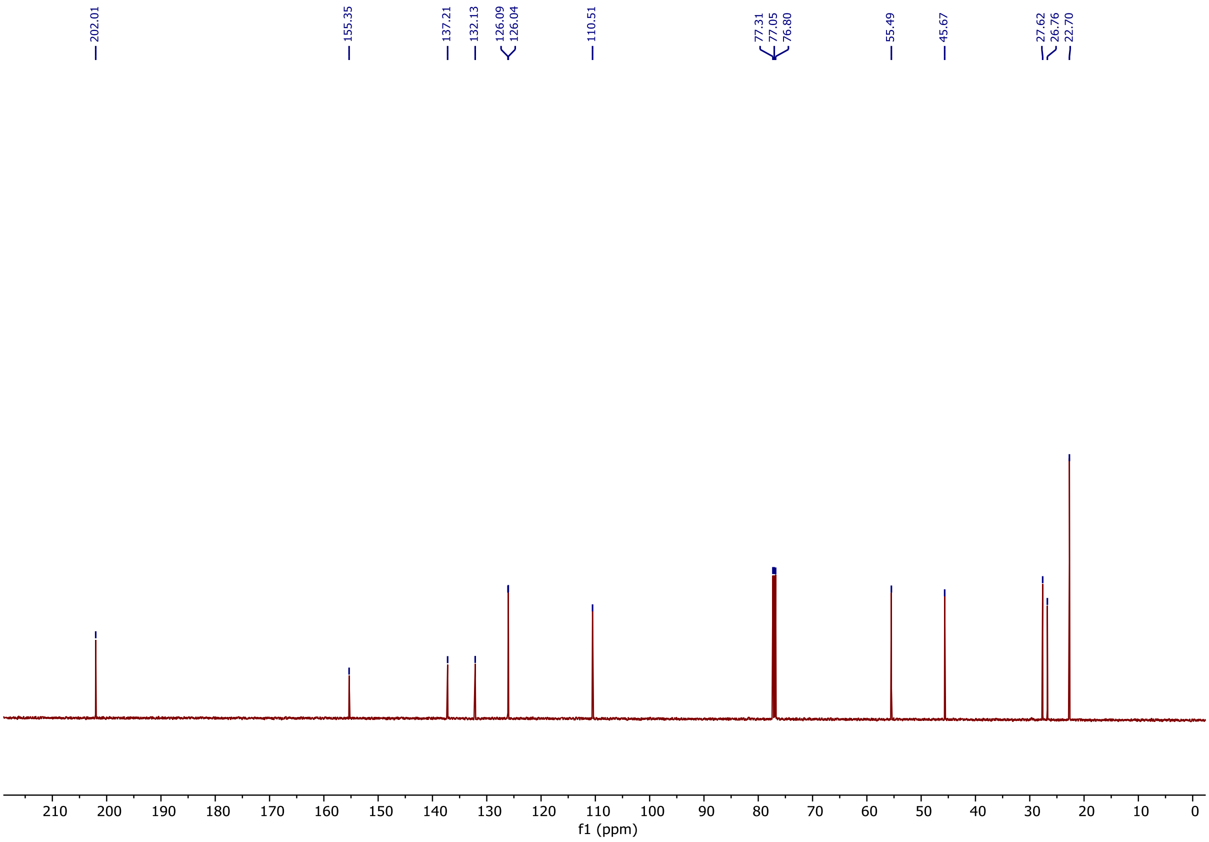

^{13}C NMR (126 MHz, $CDCl_3$) of 3-(3-isopropyl-4-methoxyphenyl)propanal

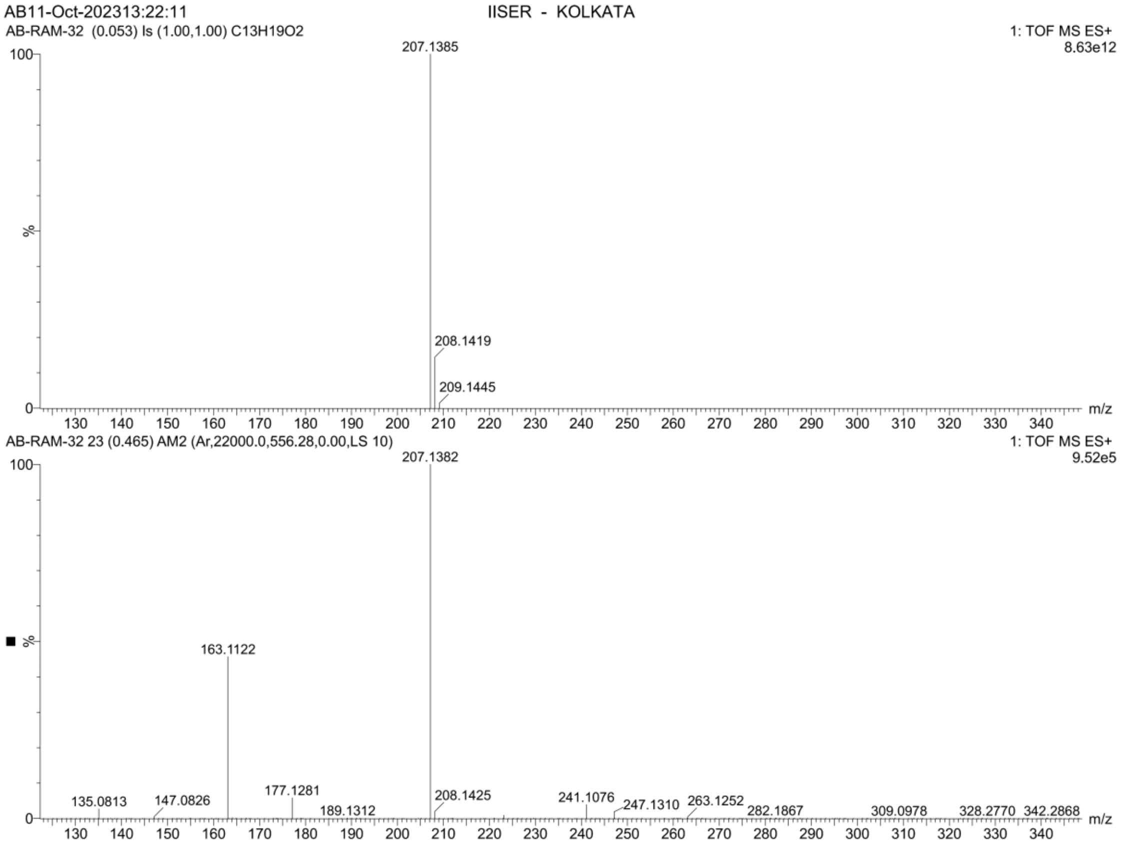

HRMS data of 3-(3-isopropyl-4-methoxyphenyl)propanal

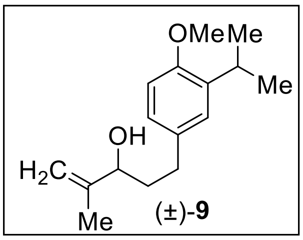

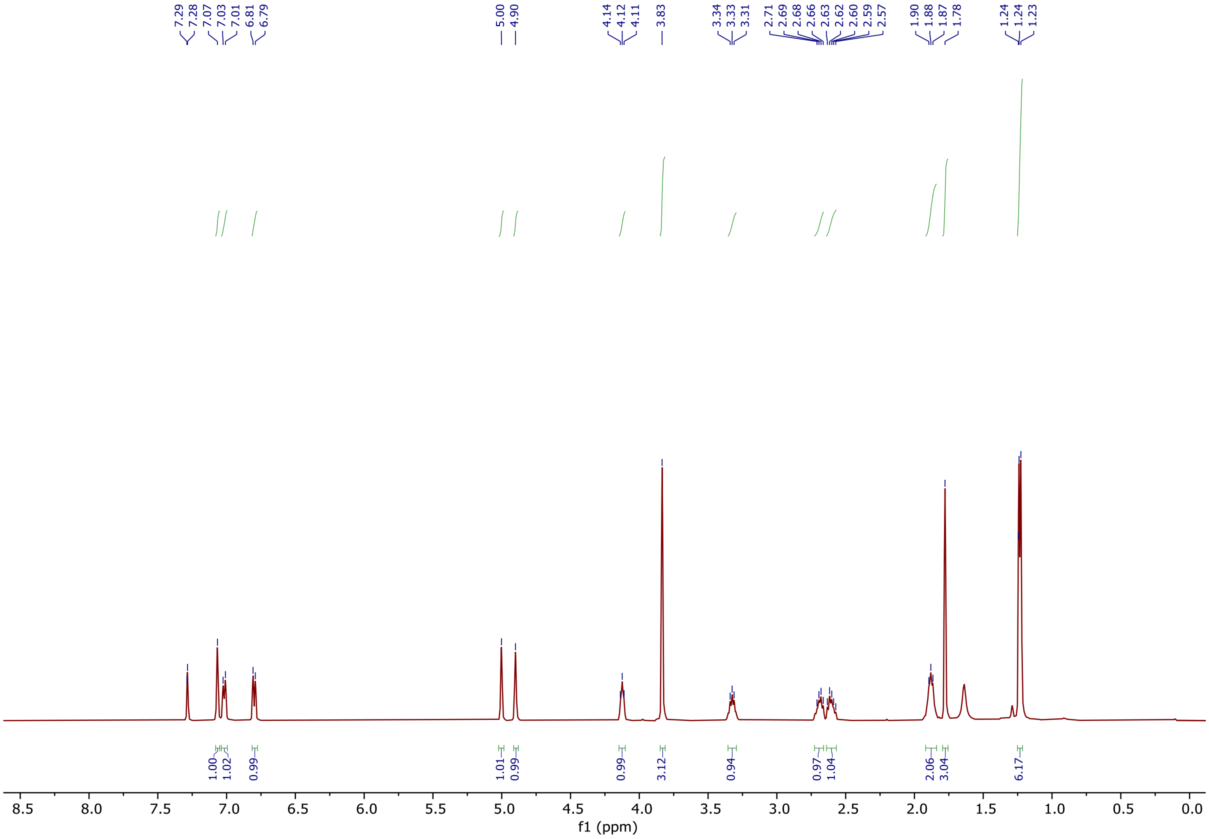

^{1}H NMR (500 MHz, $CDCl_3$) of compound (±)-**9**

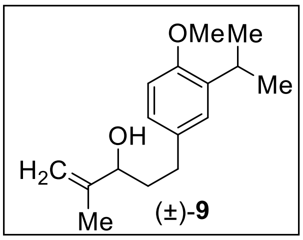

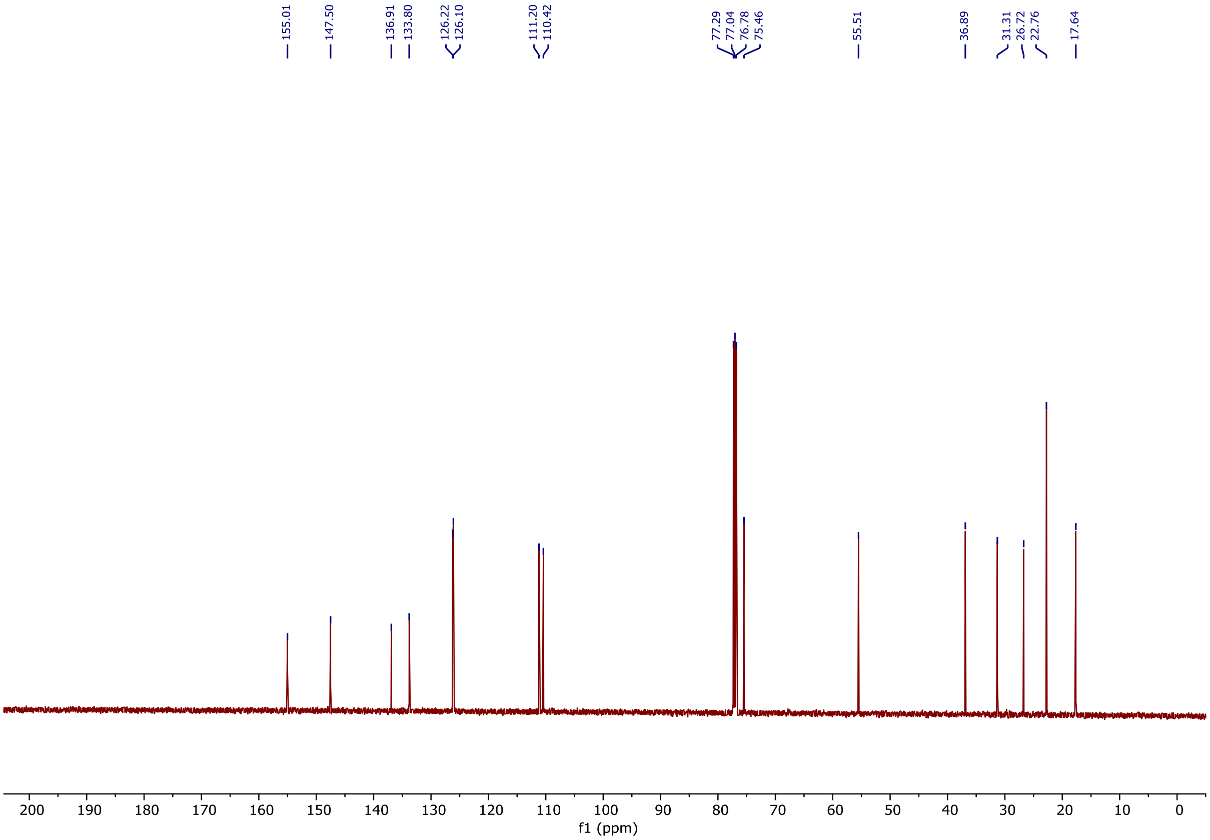

^{13}C NMR (126 MHz, $CDCl_3$) of compound (±)-**9**

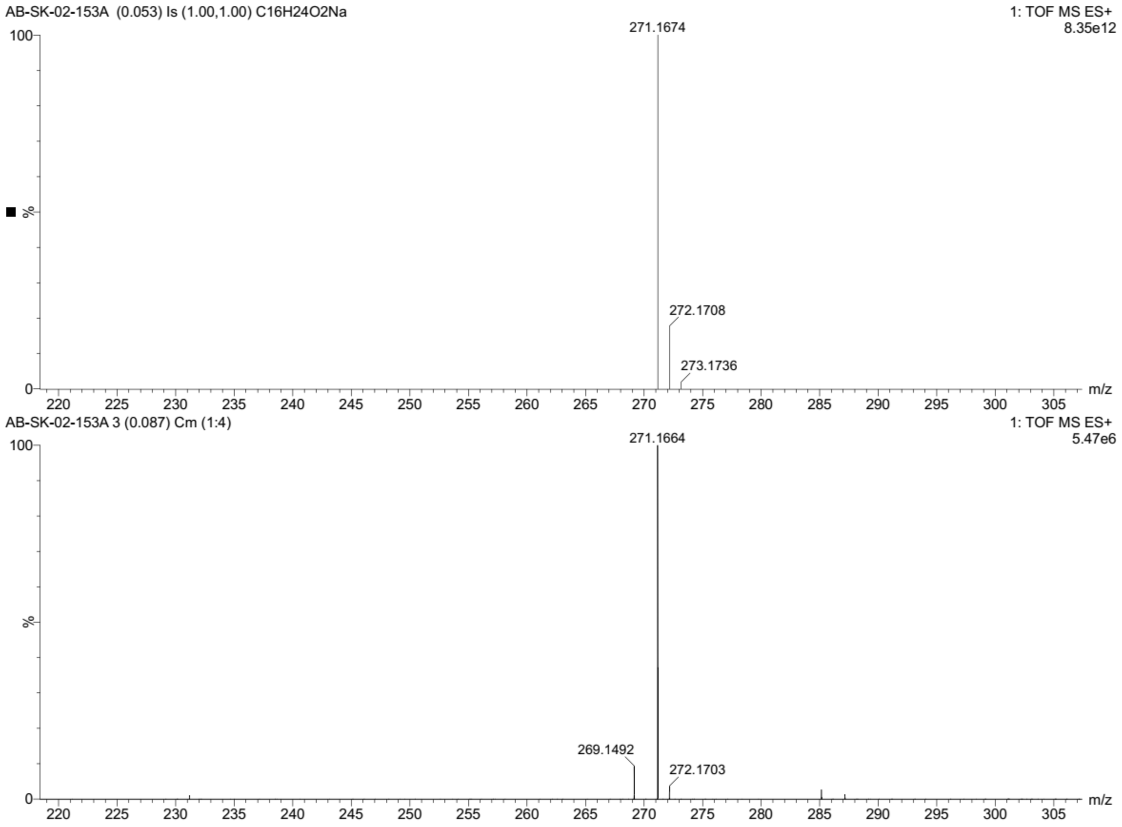

HRMS data of compound (±)-**9**

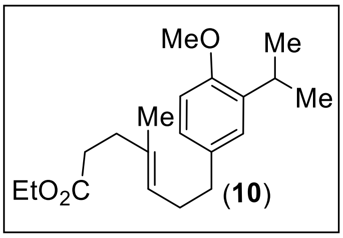

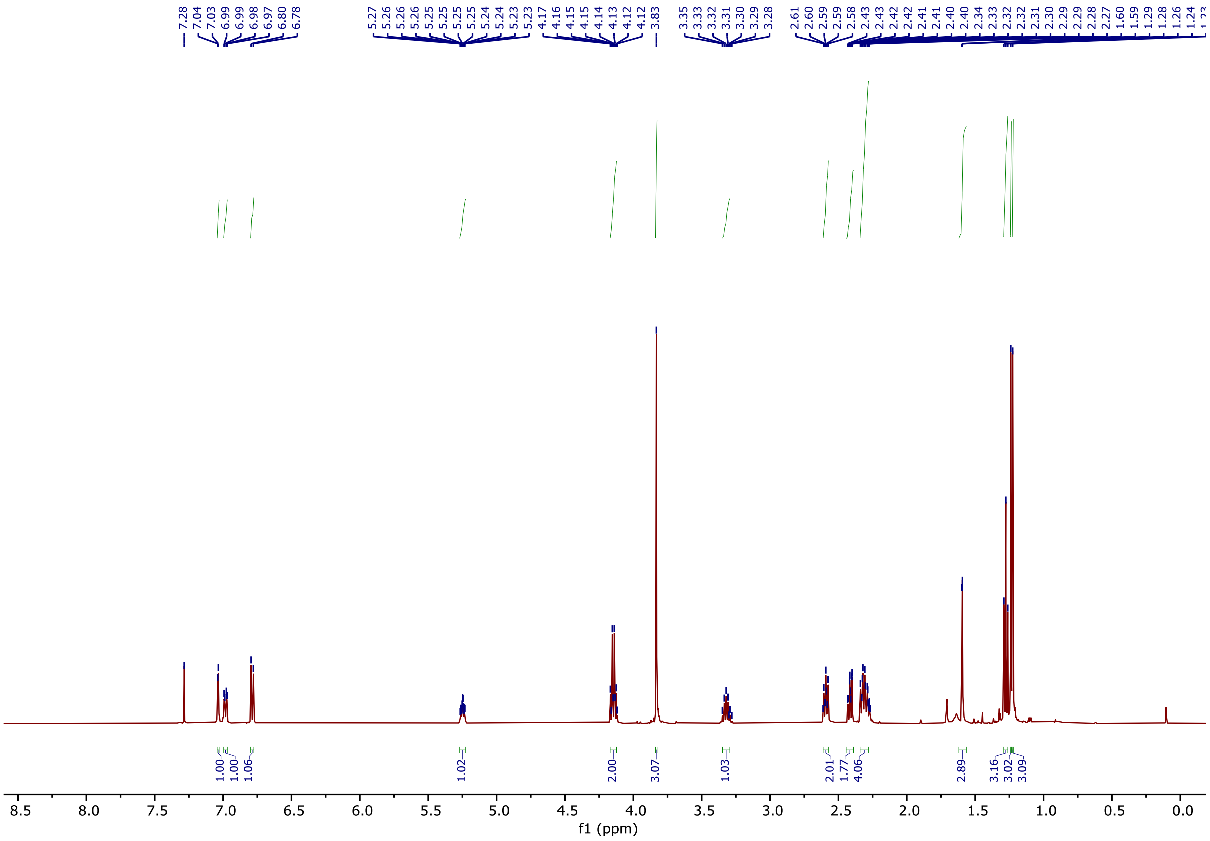

^{1}H NMR (500 MHz, $CDCl_3$) of compound **10**

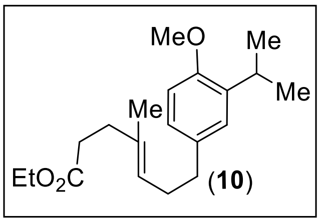

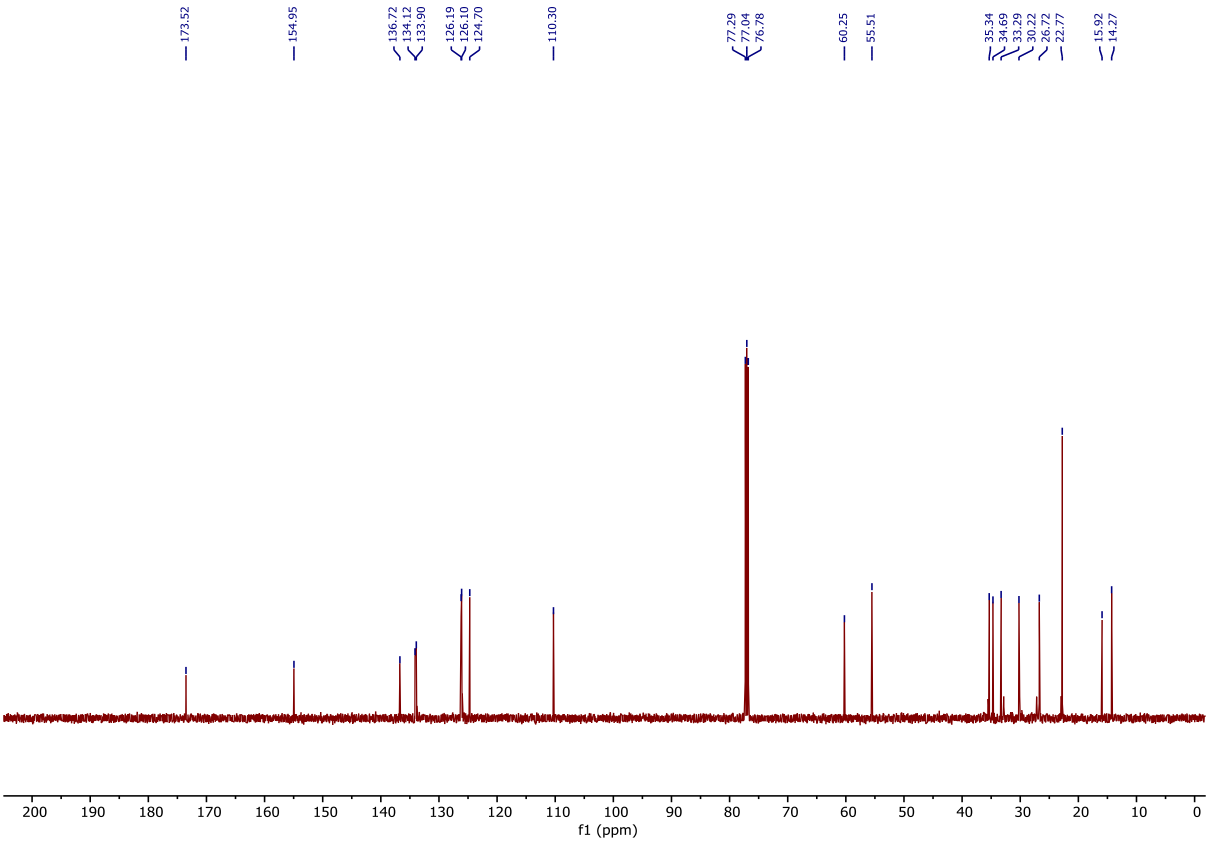

^{13}C NMR (126 MHz, $CDCl_3$) of compound **10**

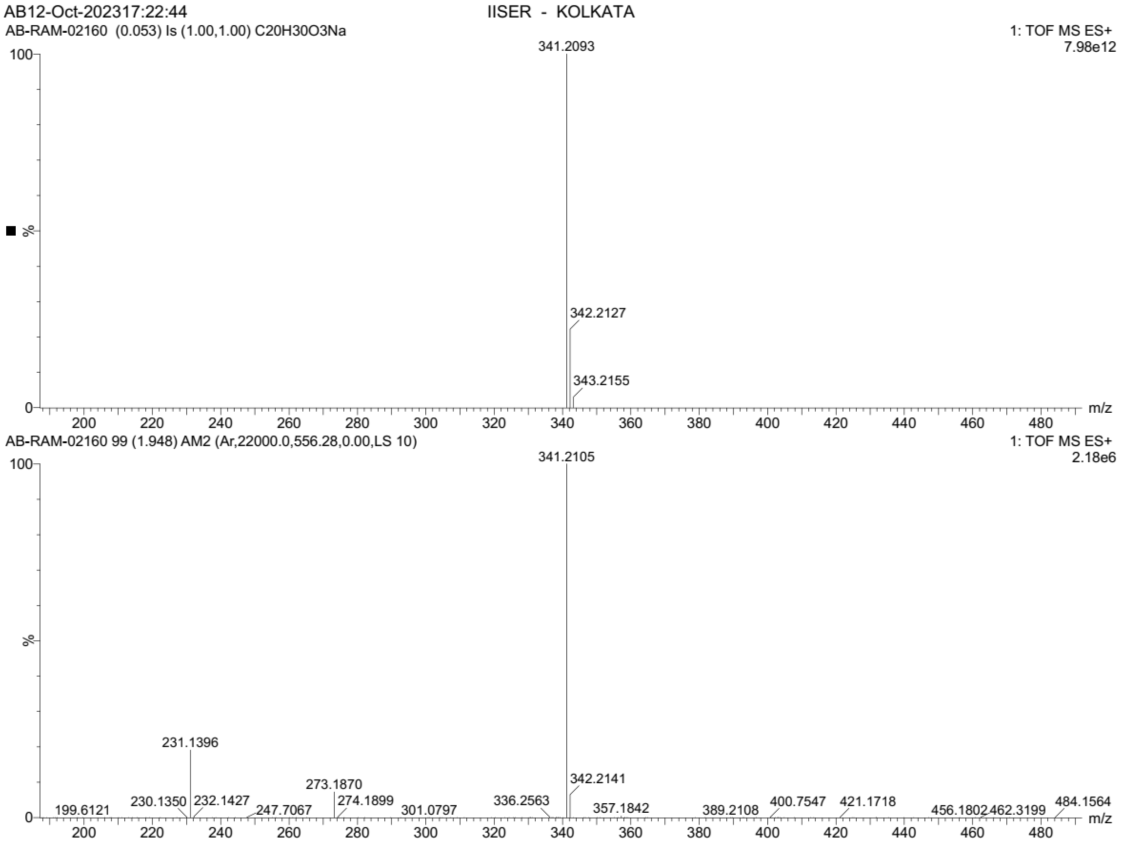

HRMS data of **10**

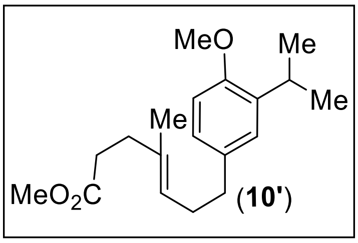

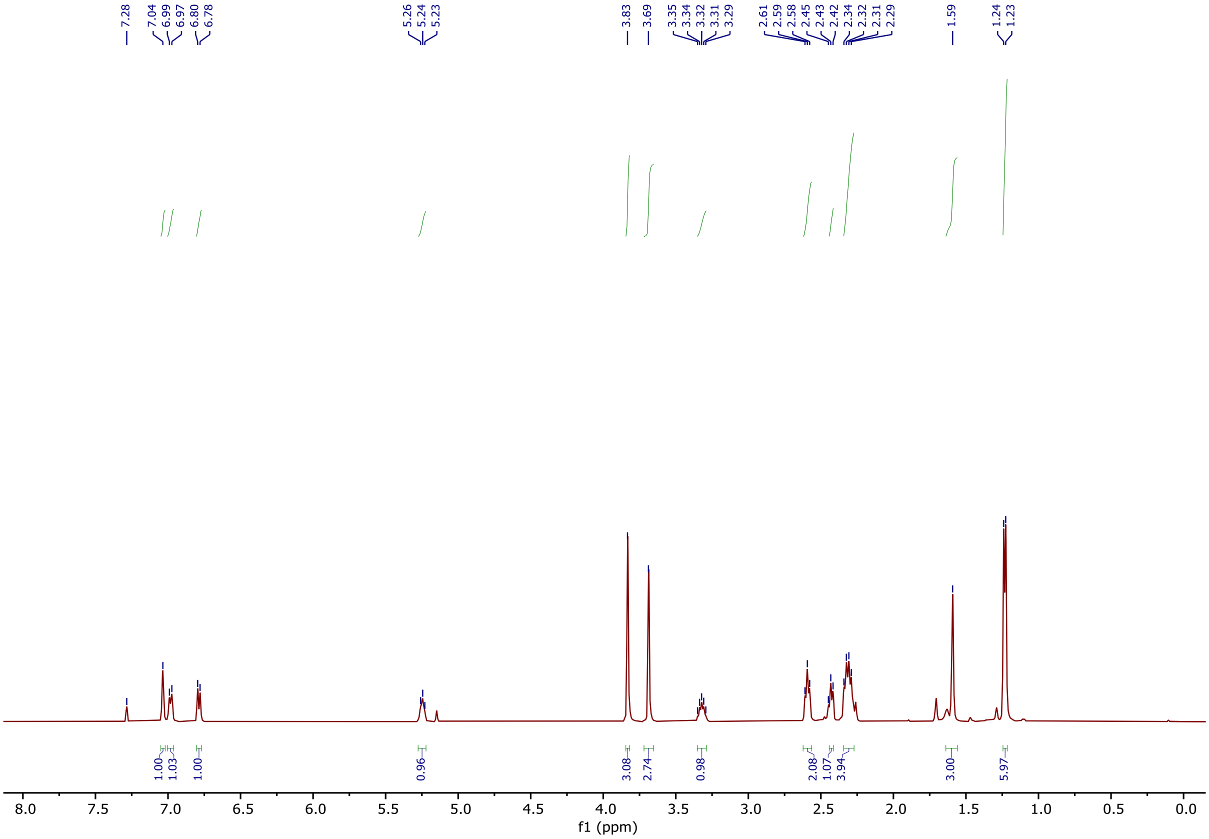

^{1}H NMR (500 MHz, $CDCl_3$) of compound **10’**

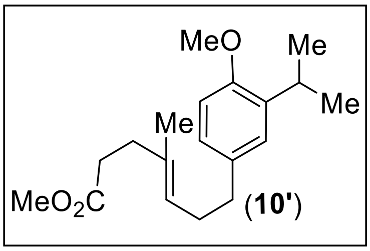

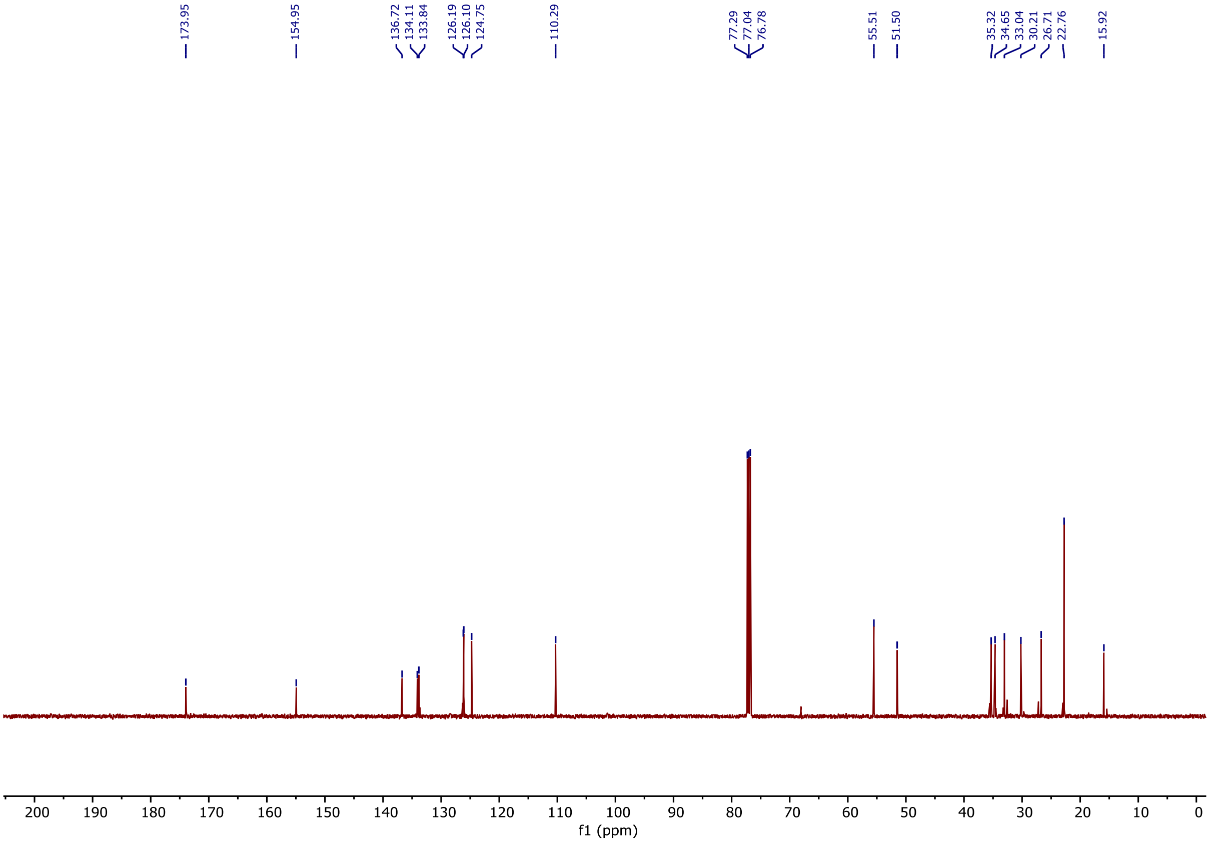

^{13}C NMR (126 MHz, $CDCl_3$) of compound **10’**

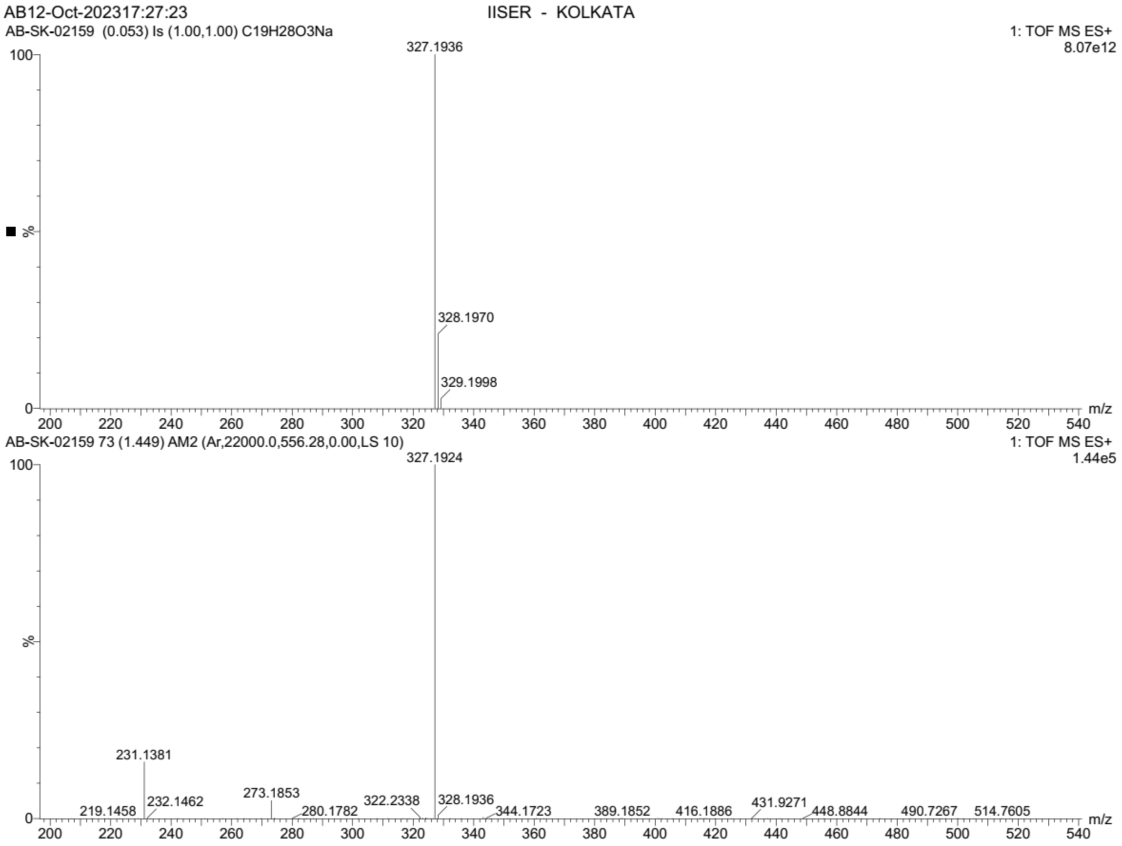

HRMS data of **10’**

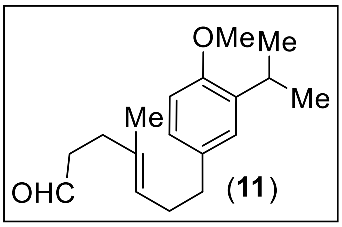

(**11**)

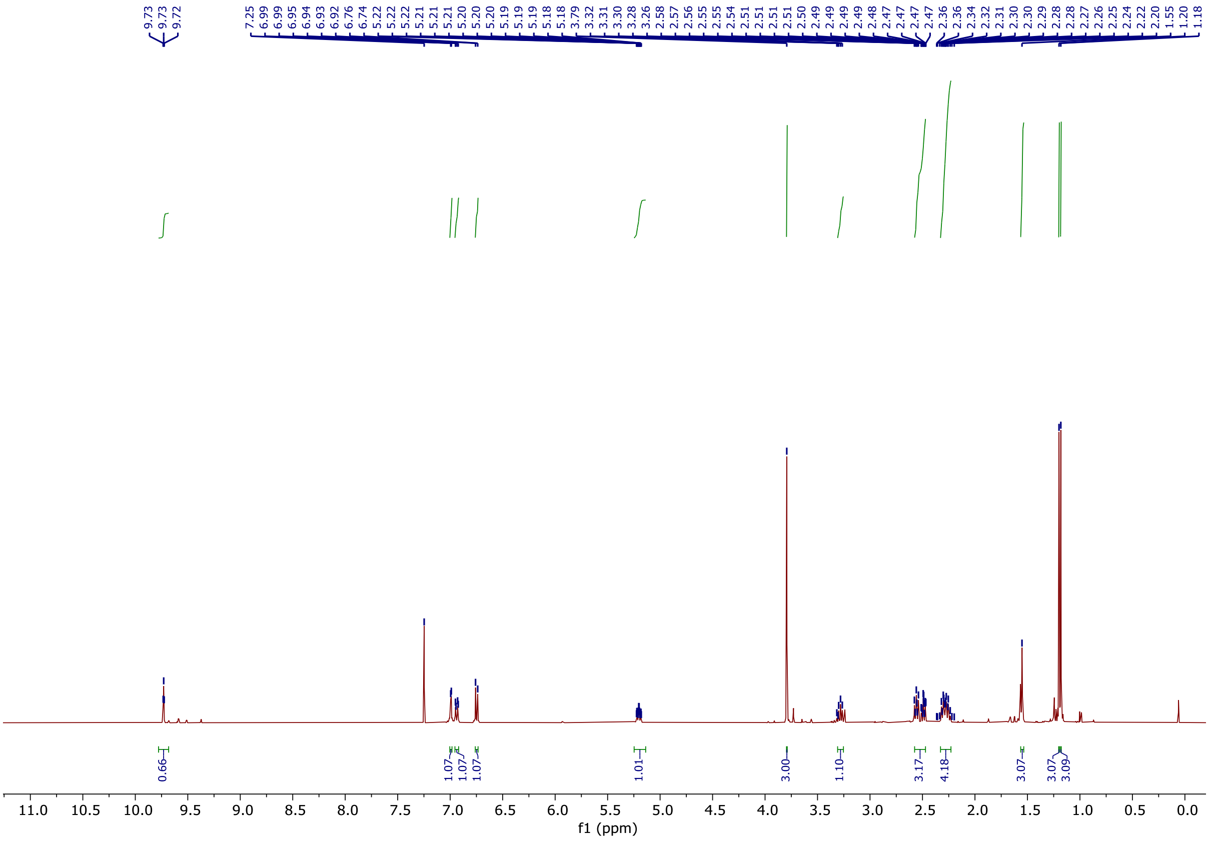

^{1}H NMR (400 MHz, $CDCl_3$) of **11**

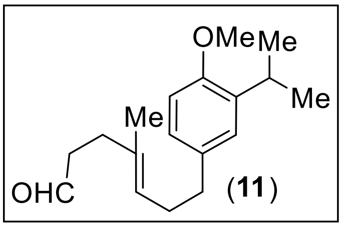

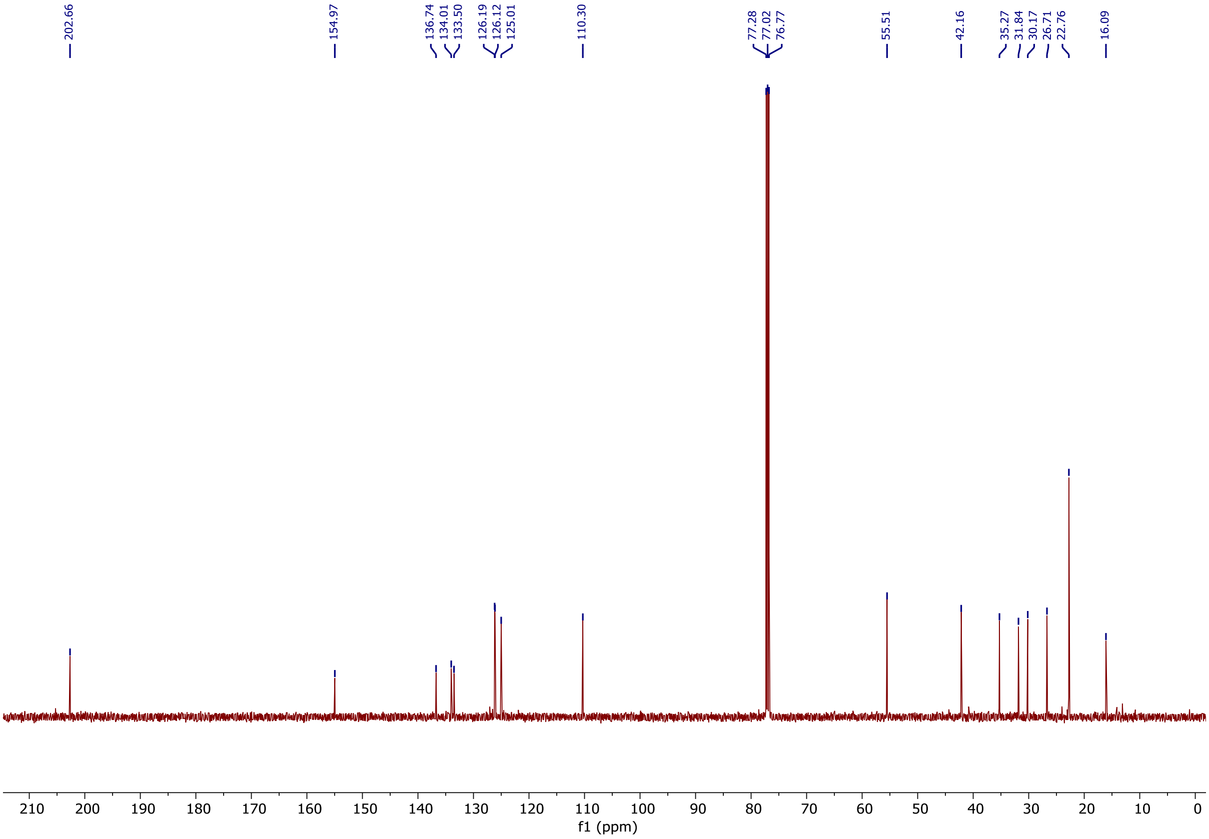

^{13}C NMR (126 MHz, $CDCl_3$) of **11**

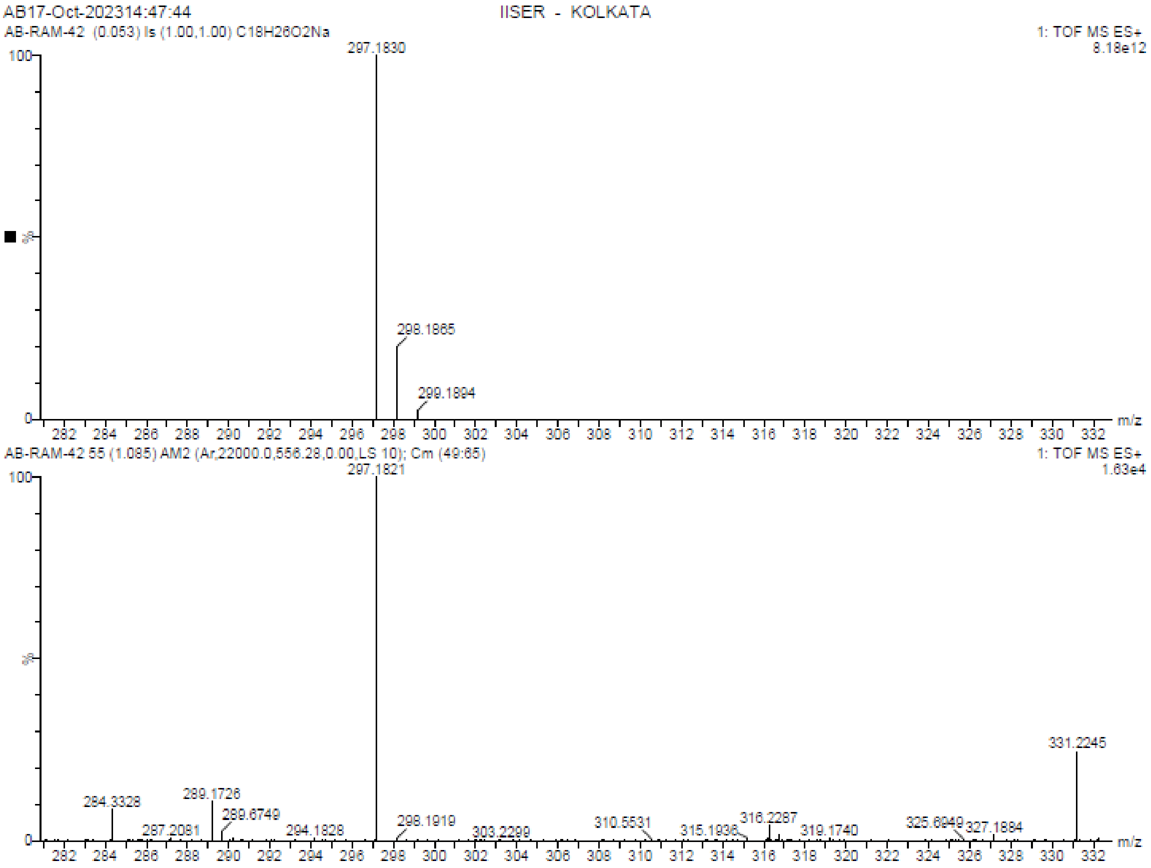

HRMS Data of **11**

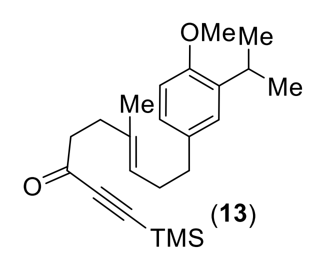

(**13**)

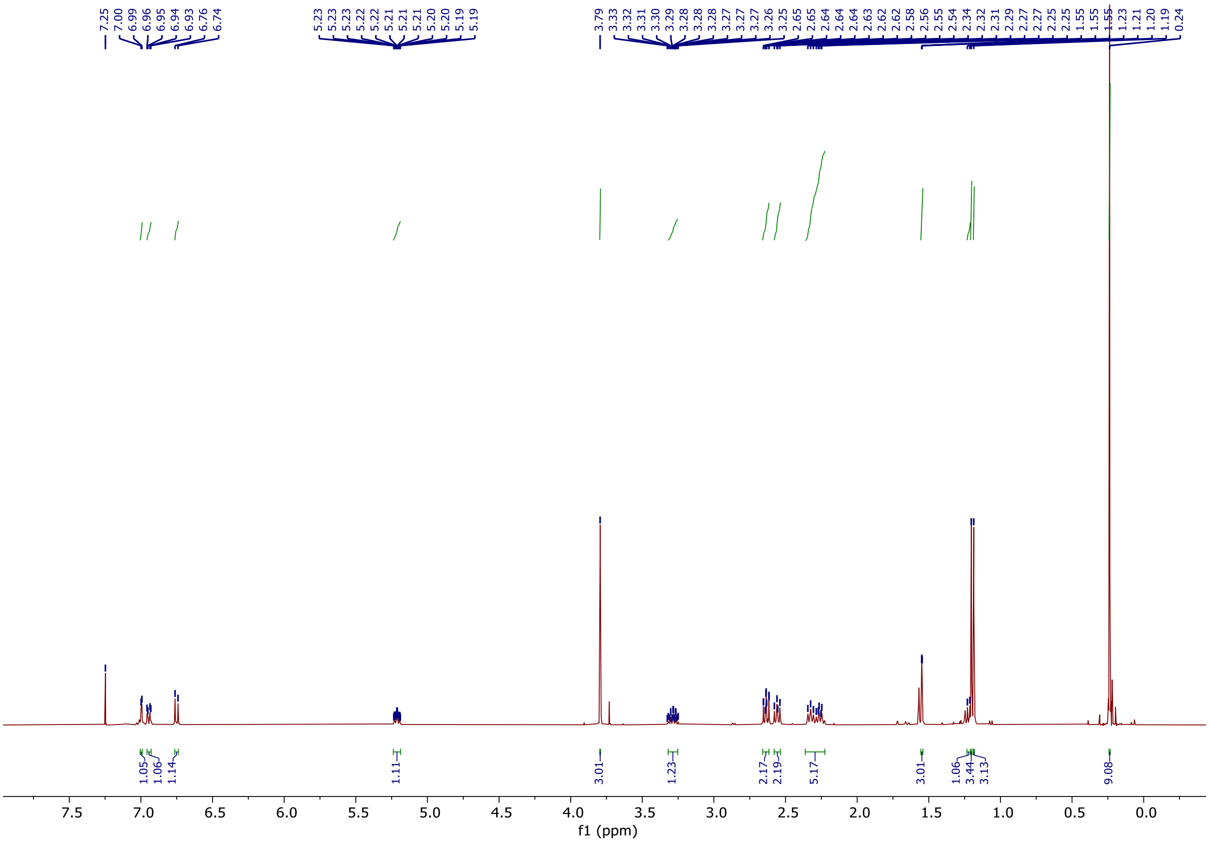

^{1}H NMR (400 MHz, $CDCl_3$) of **13**

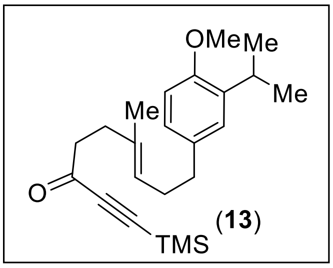

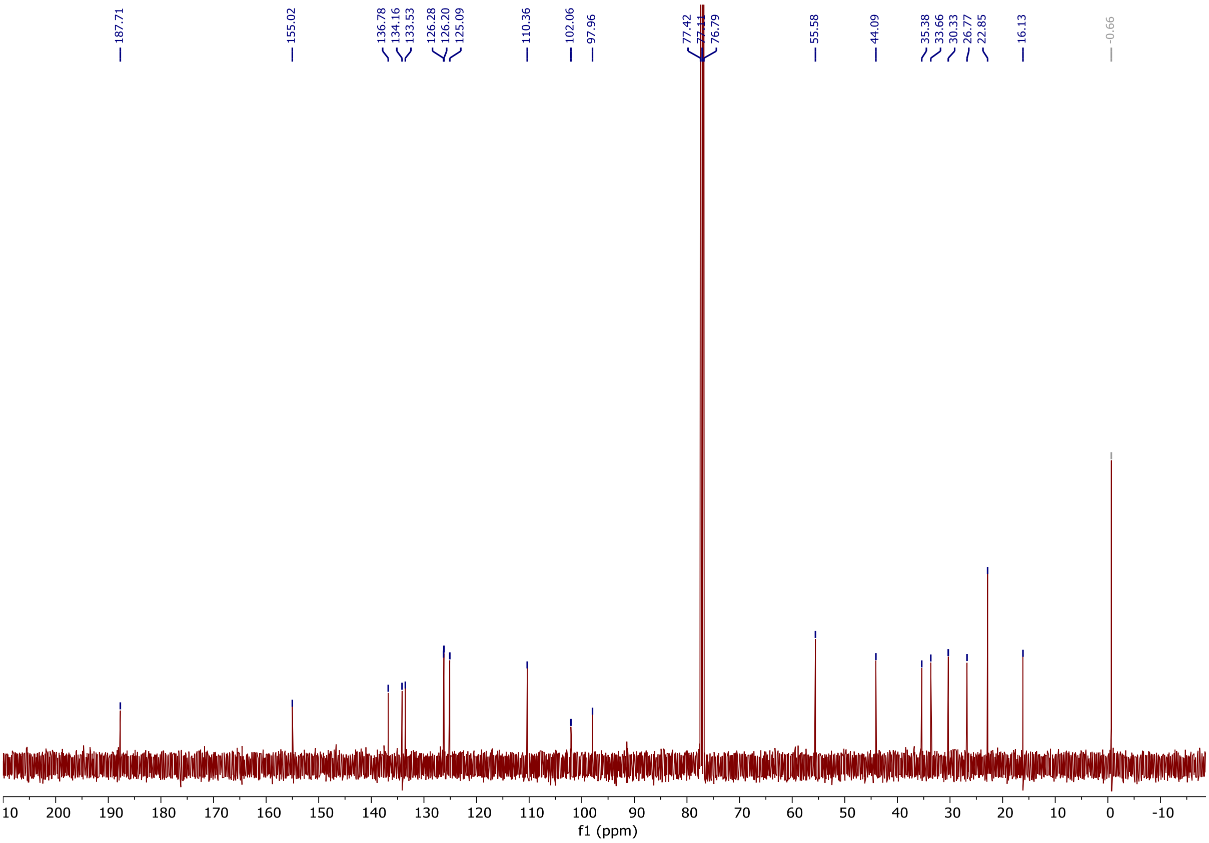

^{13}C NMR (101 MHz, $CDCl_3$) of **13**

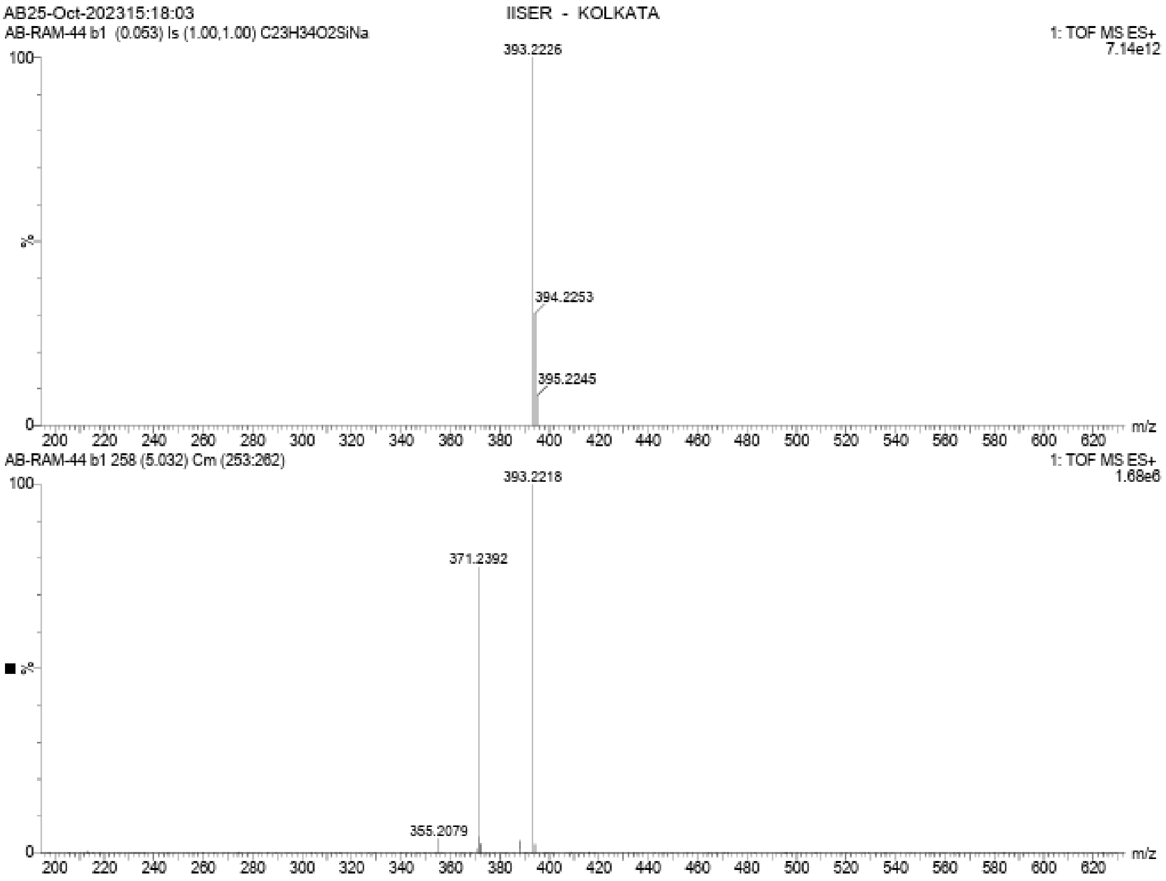

HRMS Data of **13**

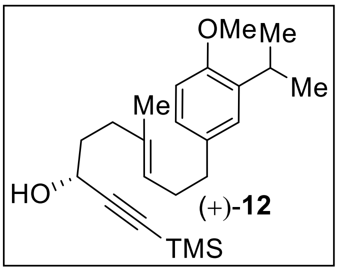

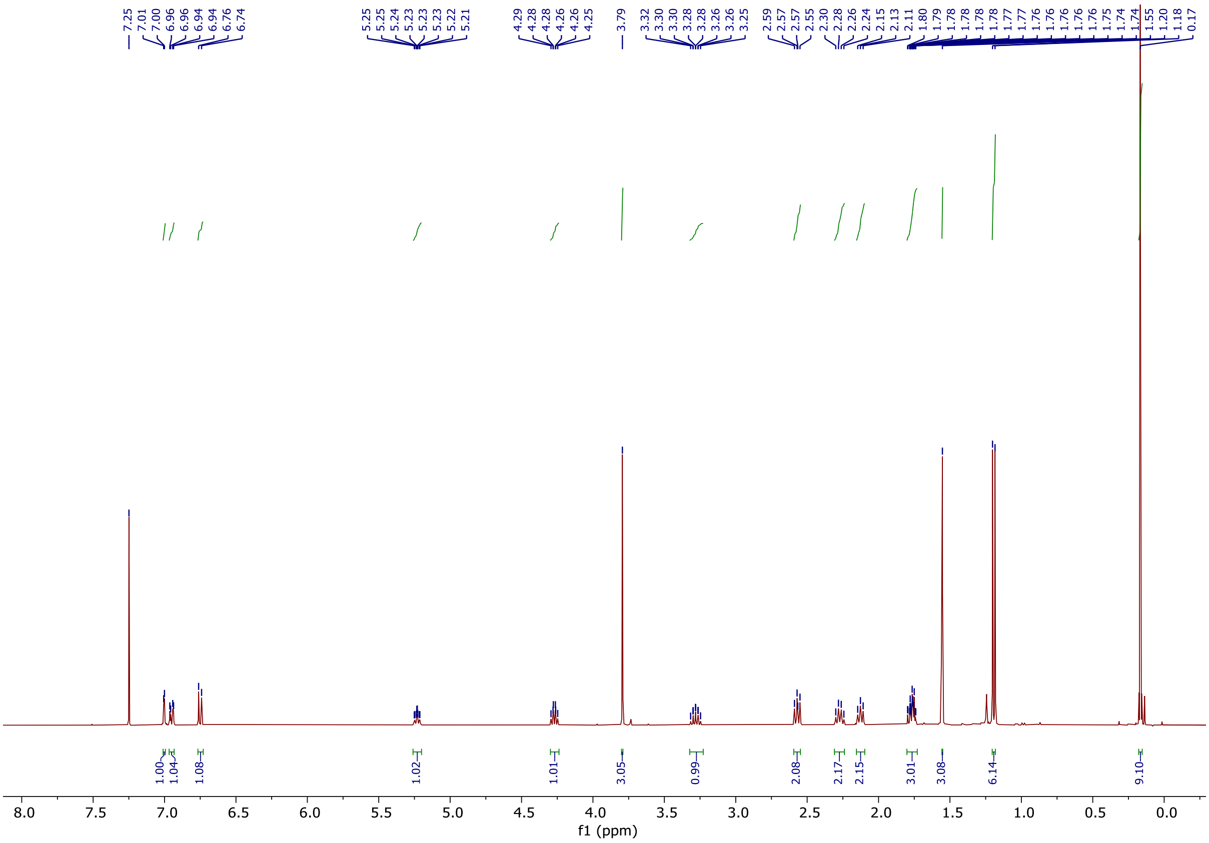

^{1}H NMR (400 MHz, $CDCl_3$) of (+)-**12**

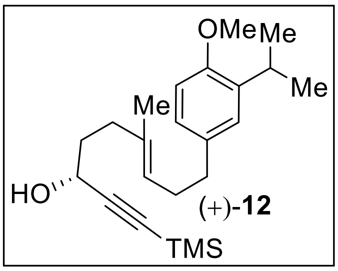

(+)-**12**

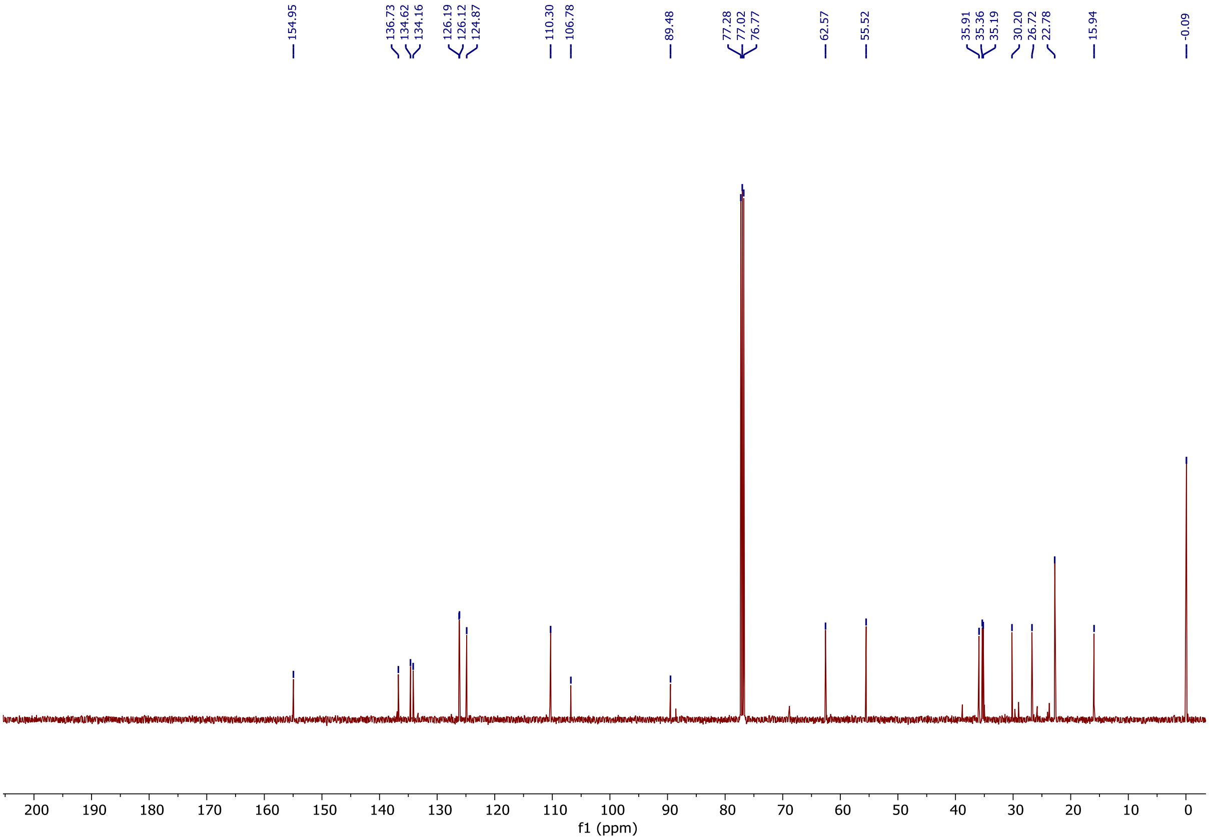

^{13}C NMR (126 MHz, $CDCl_3$) of (+)-**12**

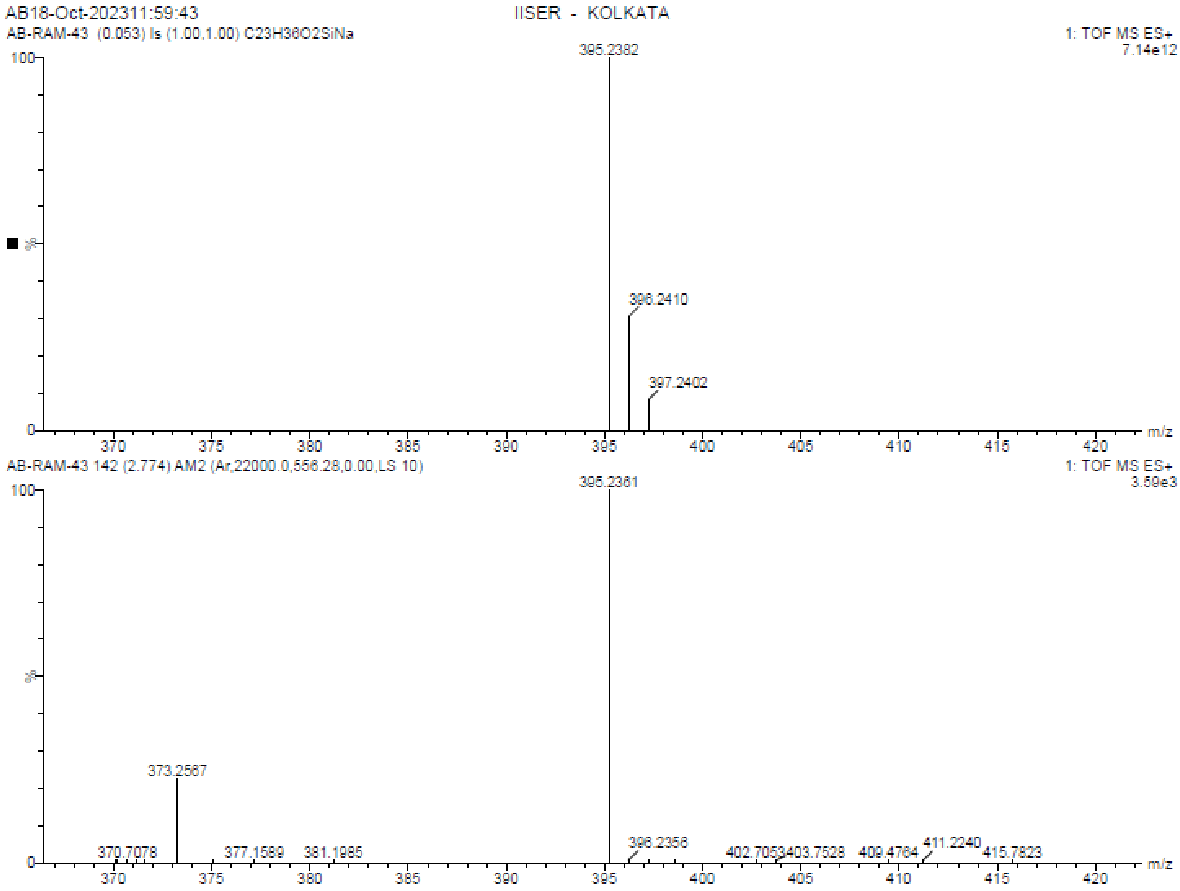

HRMS Data of (+)-**12**

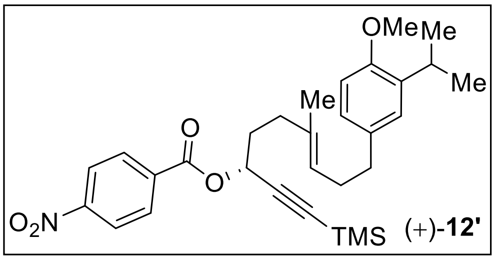

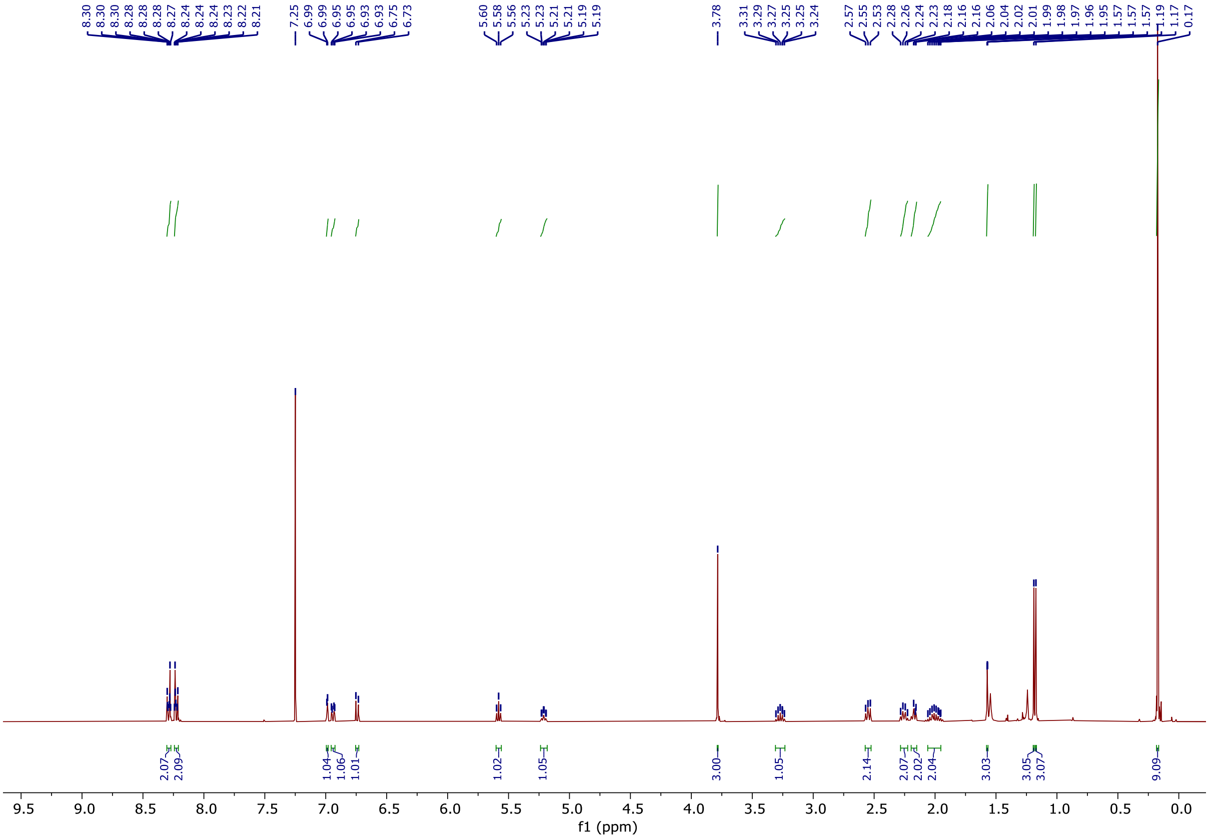

^{1}H NMR (400 MHz, $CDCl_3$) of (+)-**12’**

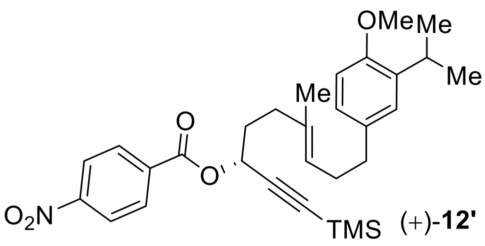

(+)-**12'**

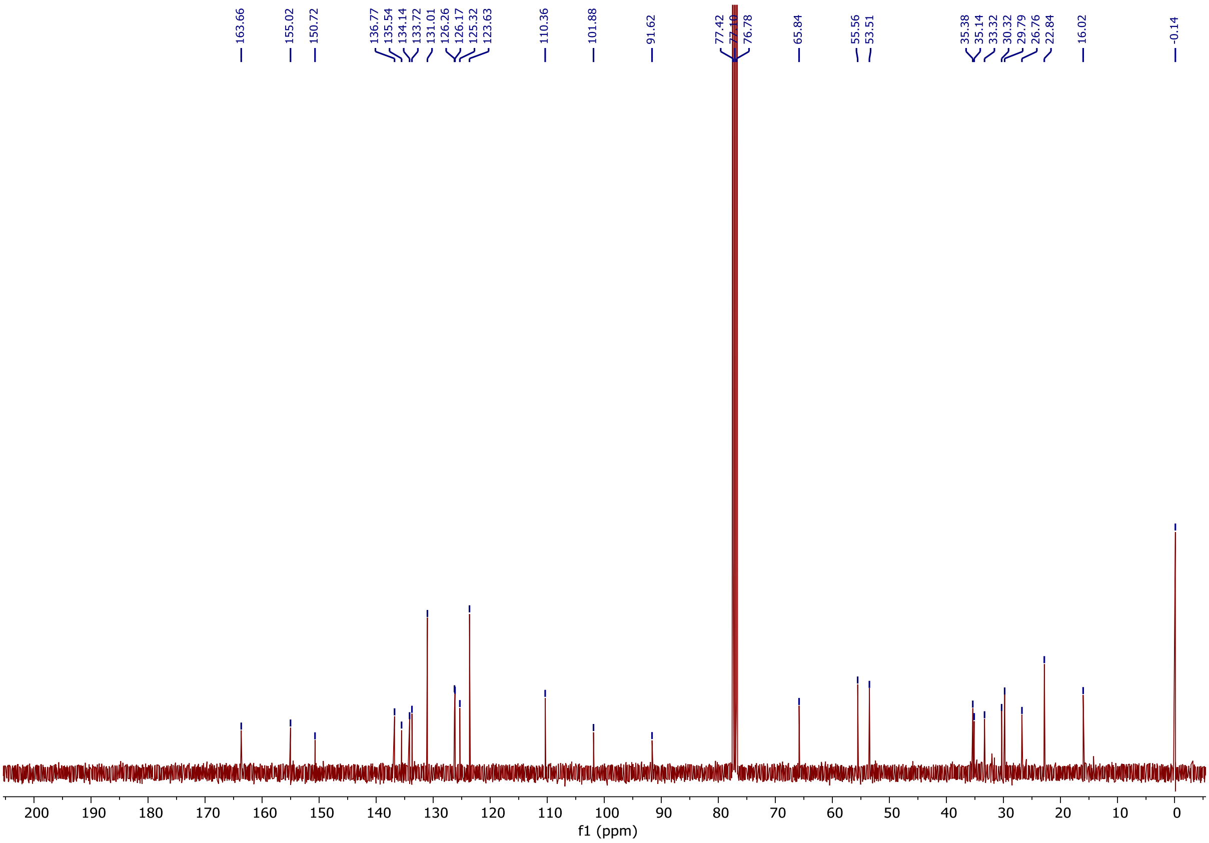

^{13}C NMR (101 MHz, $CDCl_3$) of (+)-**12'**

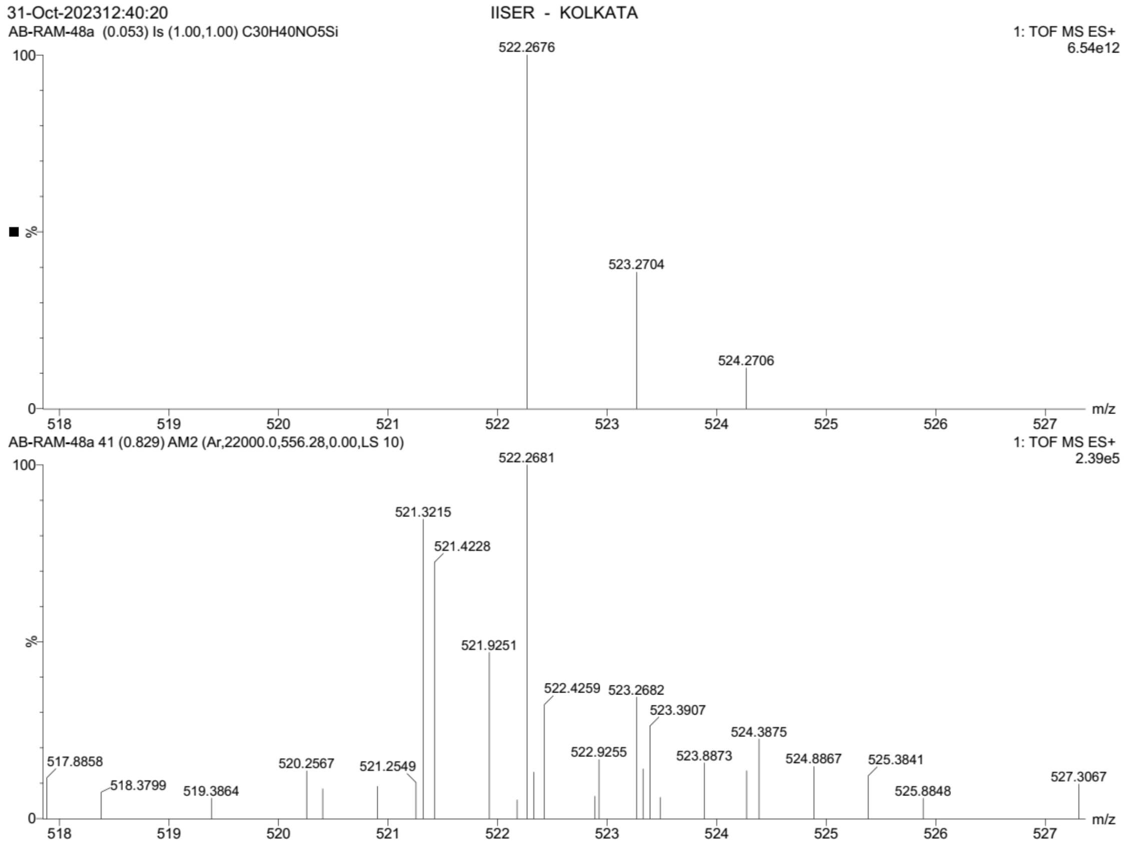

HRMS Data of (+)-**12’**

Peak Summary Report

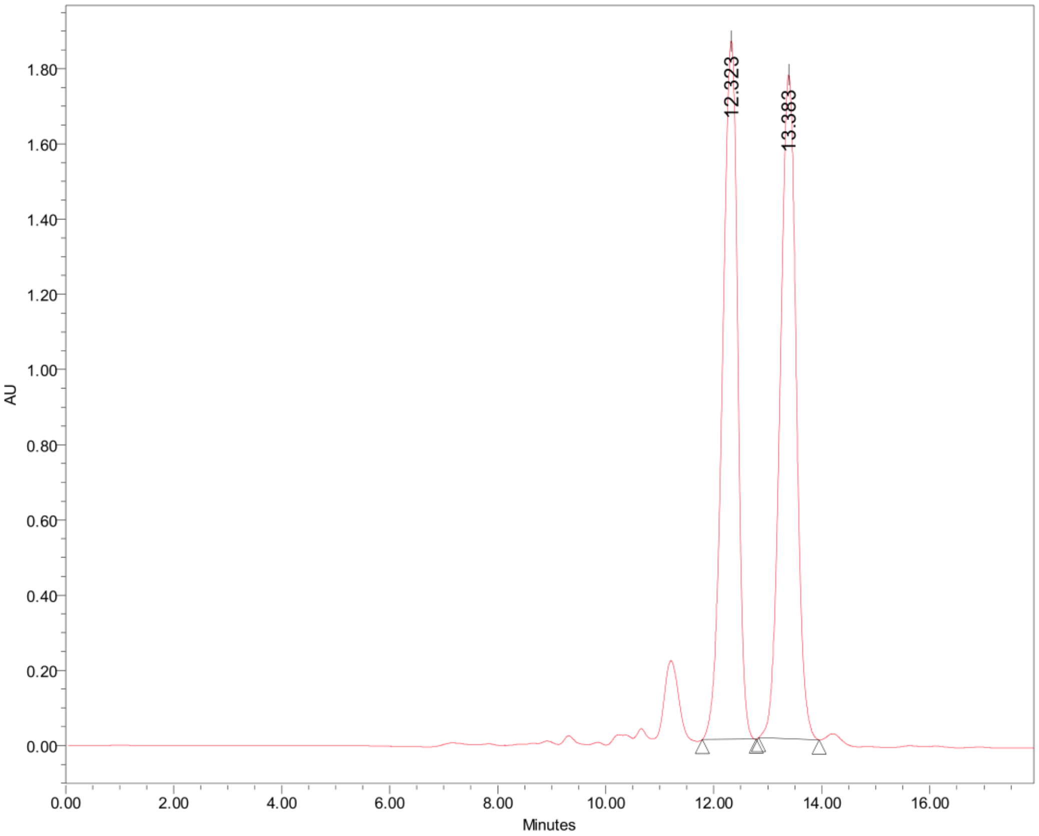

Sample Name: AB_RAM_48_RAC_AD-H_1_1; Date Acquired: 27-10-2023 11:15:04 IST; Vial: 1; Injection:

Peak Summary with Statistics
Name:

	Sample Name	Vial	Inj	Retention Time (min)	Area	% Area	Height
1	AB_RAM_48_RAC_AD-H_1_1	1	1	13.383	36362984	50.18	1765525
2	AB_RAM_48_RAC_AD-H_1_1	1	1	12.323	36102165	49.82	1857250
Mean				12.853			
Std. Dev.				0.750			

HPLC Data of (±)-**12’**

Peak Summary Report

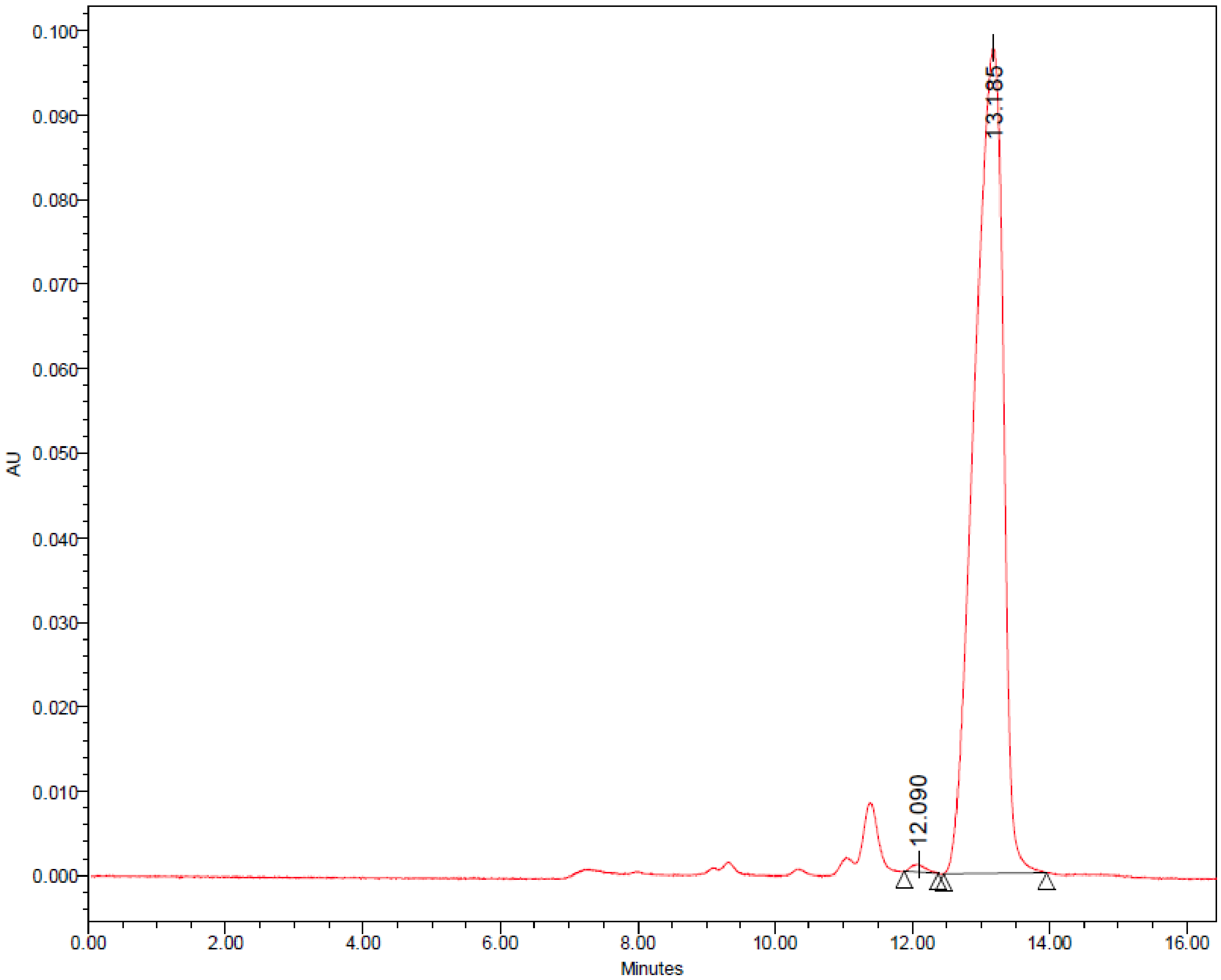

Sample Name: AB_RAM_48_RAC_AD-H_1_0P4_1; Date Acquired: 27-10-2023 21:55:11 IST; Vial: 1 Injection: 1

Peak Summary with Statistics
Name:

	Sample Name	Vial	Inj	Retention Time (min)	Area	% Area	Height
1	AB_RAM_48_RAC_AD-H_1_0P4_1	1	1	13.185	2838946	99.51	97661
2	AB_RAM_48_RAC_AD-H_1_0P4_1	1	1	12.090	13853	0.49	952
Mean				12.638			
Std. Dev.				0.774			

HPLC Data of (+)-**12'**

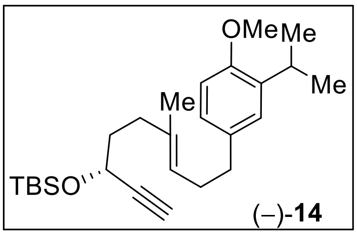

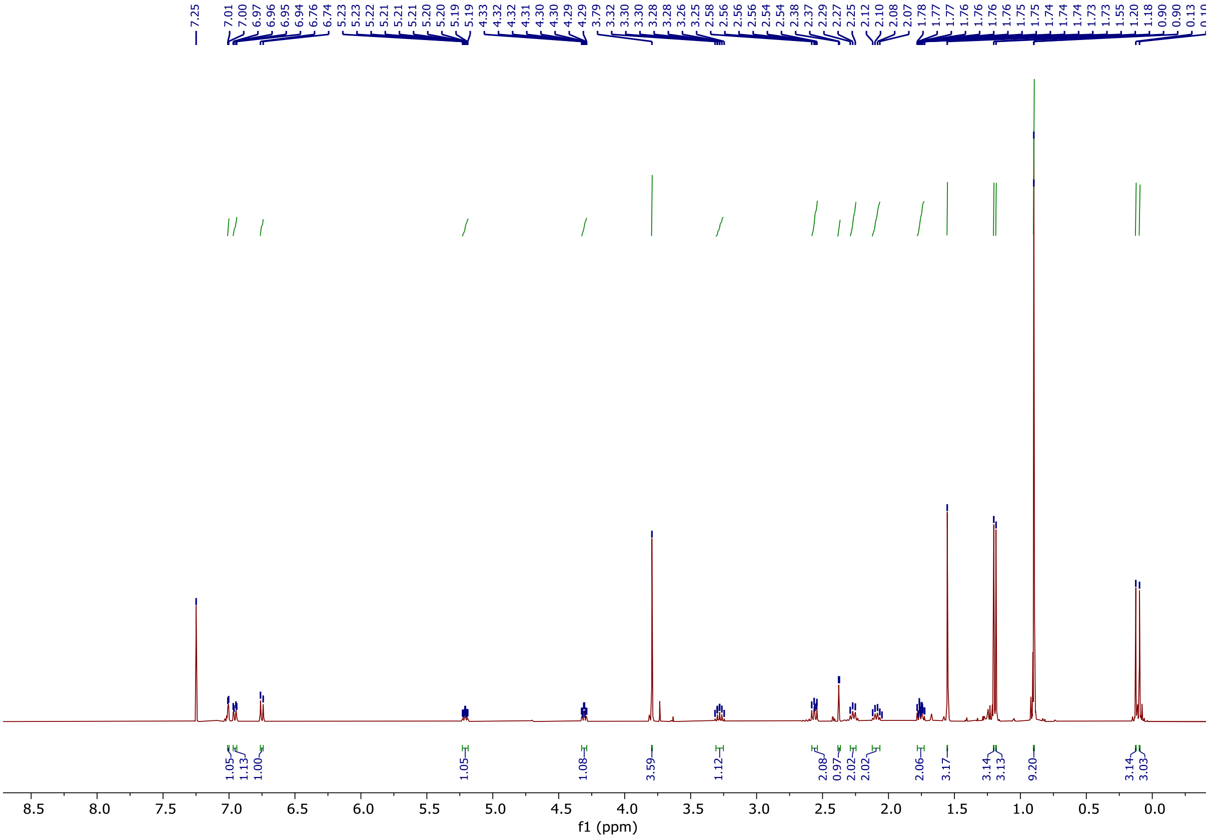

^{1}H NMR (400 MHz, $CDCl_3$) of (–)-**14**

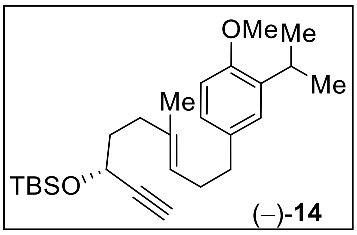

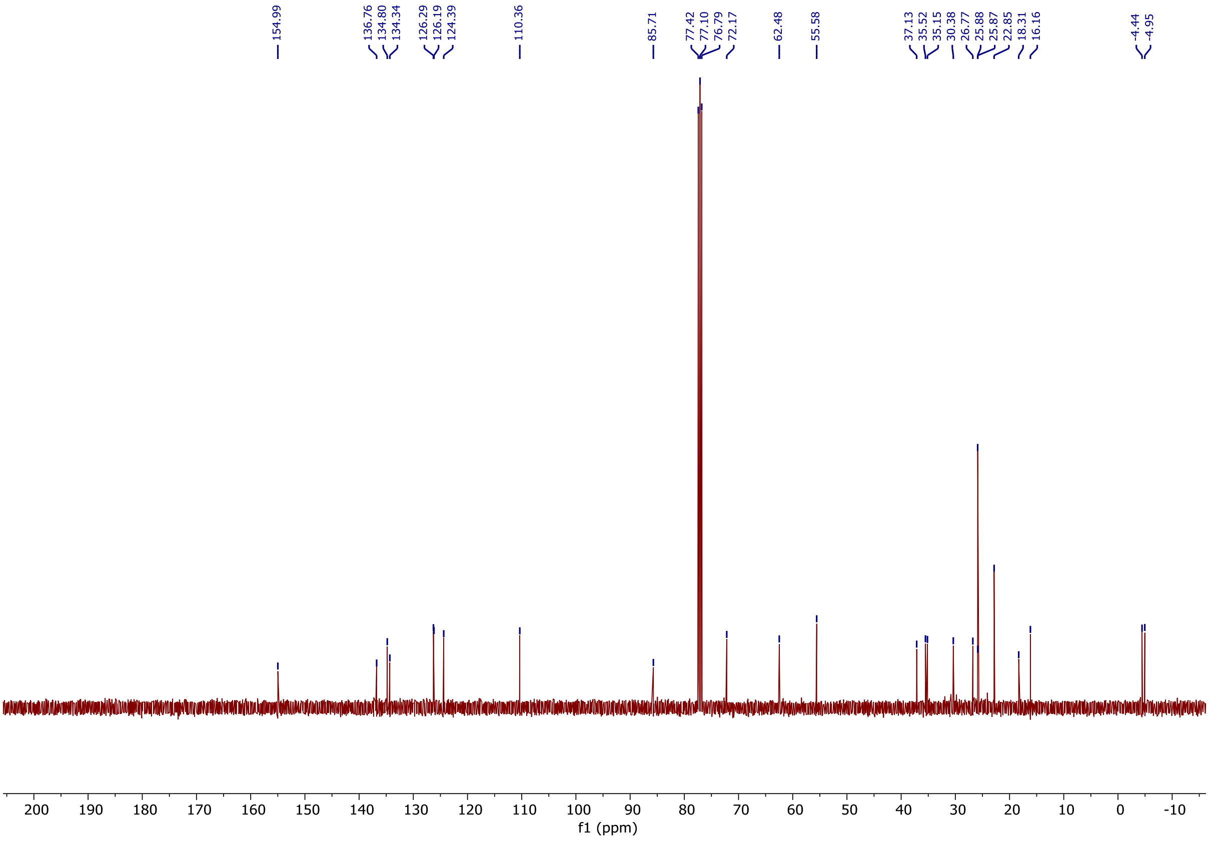

^{13}C NMR (101 MHz, $CDCl_3$) of (–)-**14**

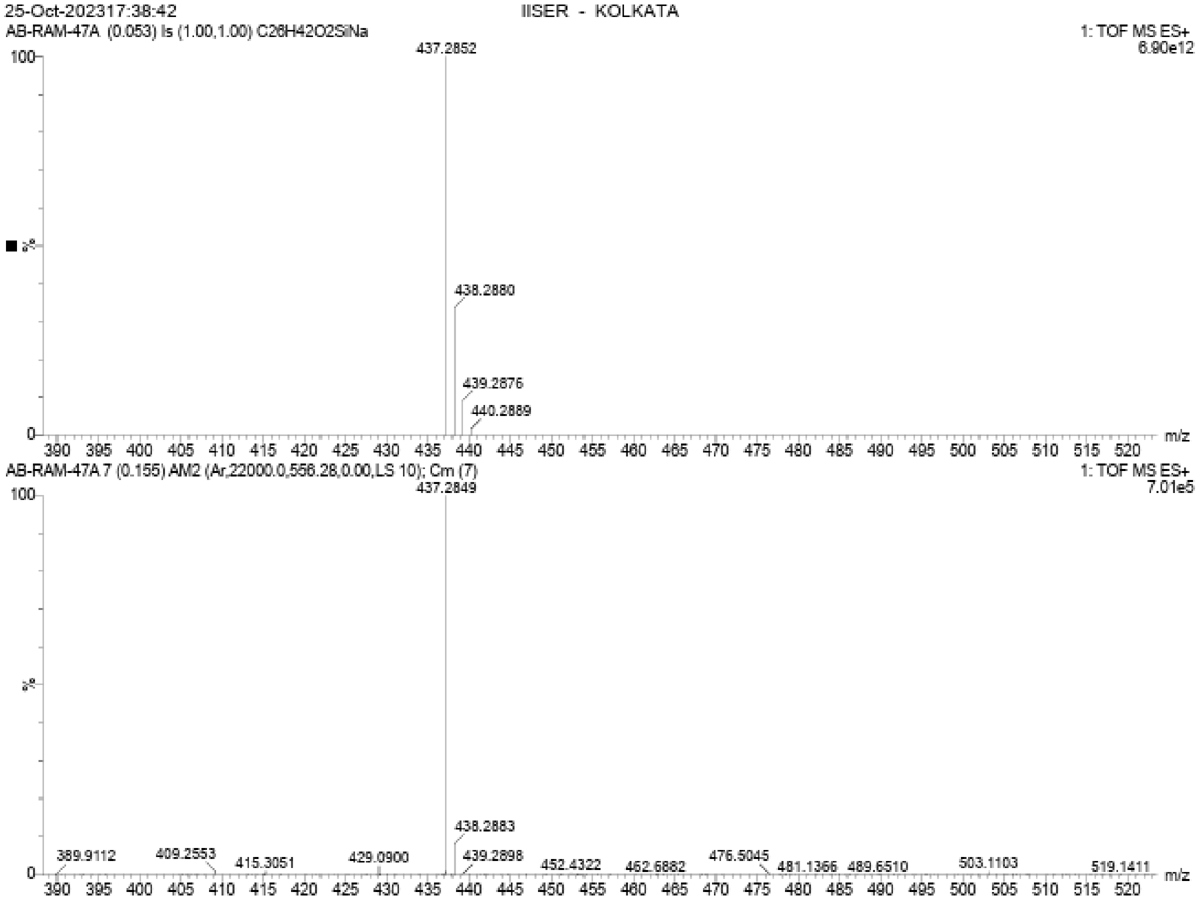

HRMS Data of (−)-**14**

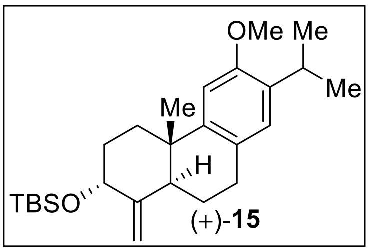

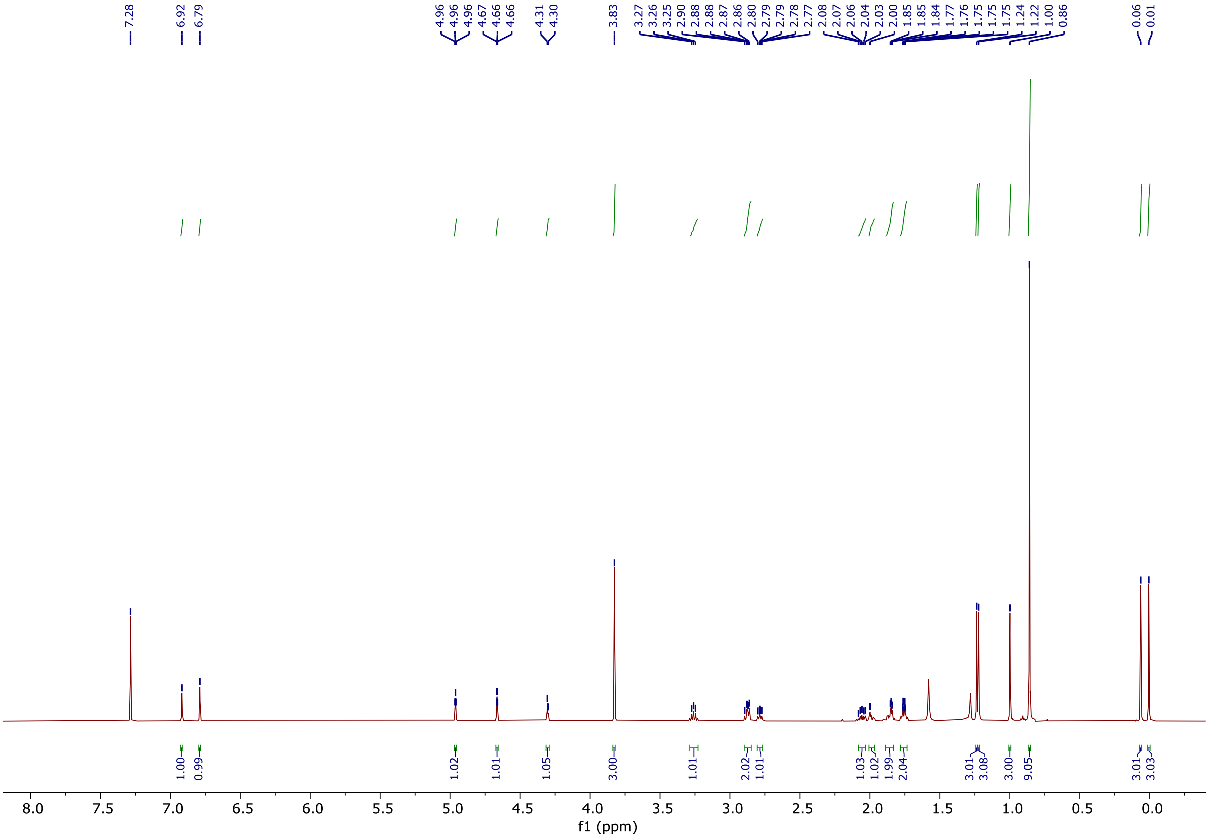

^{1}H NMR (400 MHz, $CDCl_3$) of (+)-**15**

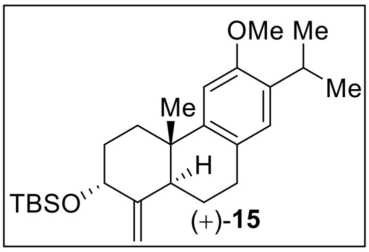

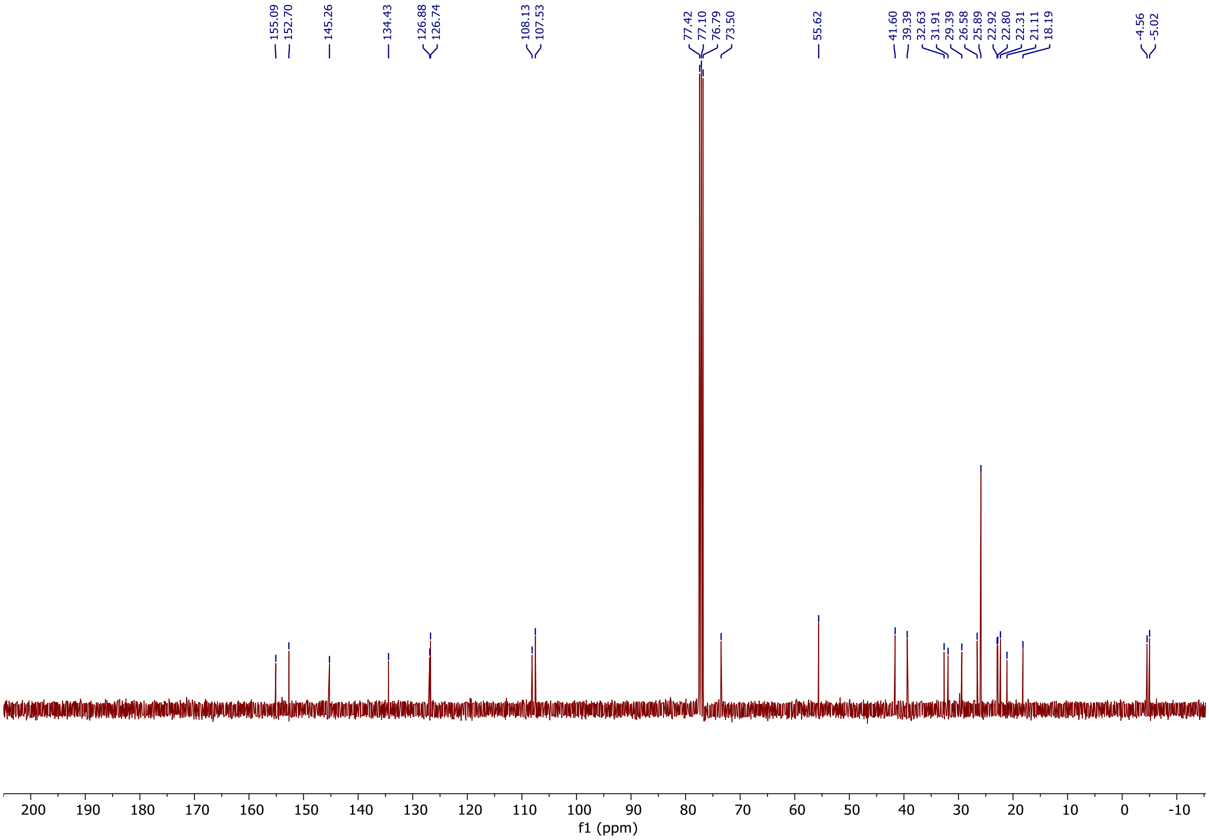

^{13}C NMR (101 MHz, $CDCl_3$) of (+)-**15**

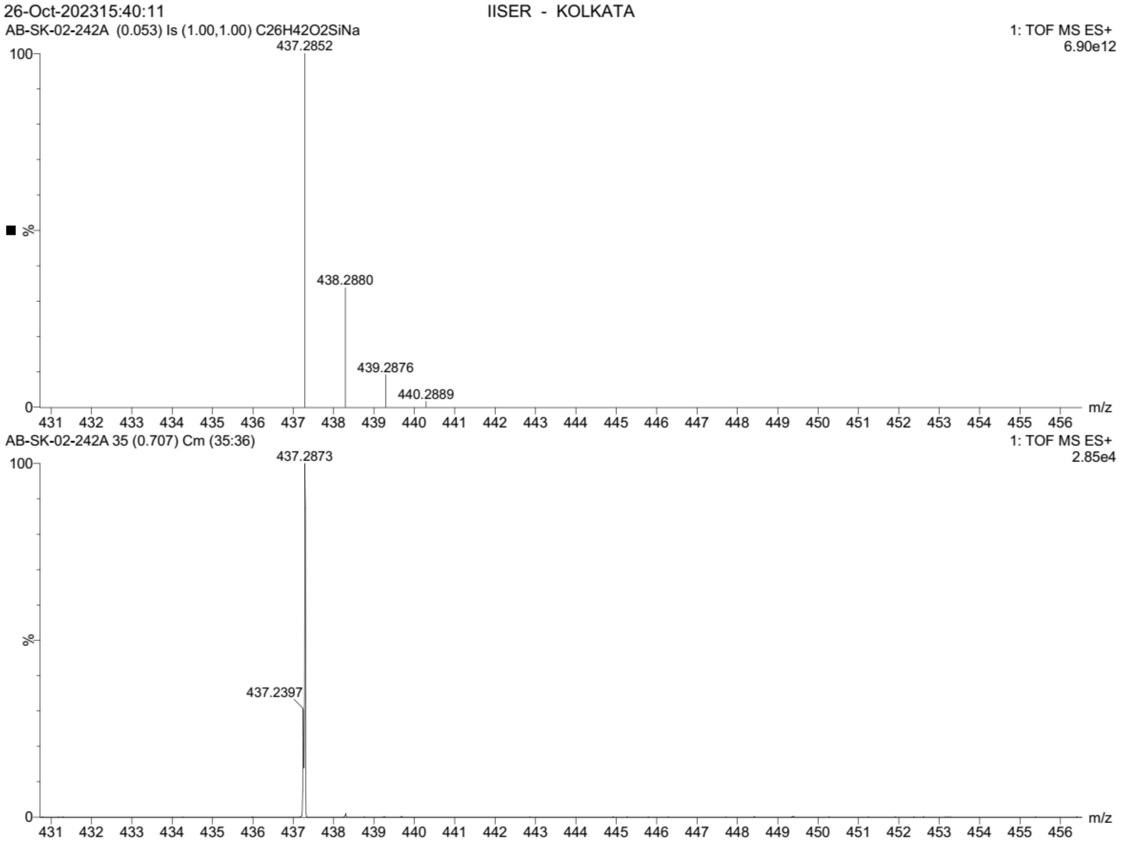

HRMS Data of (+)-**15**

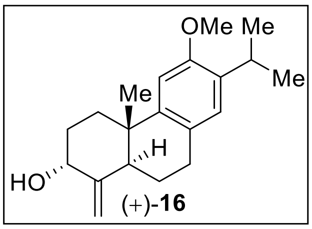

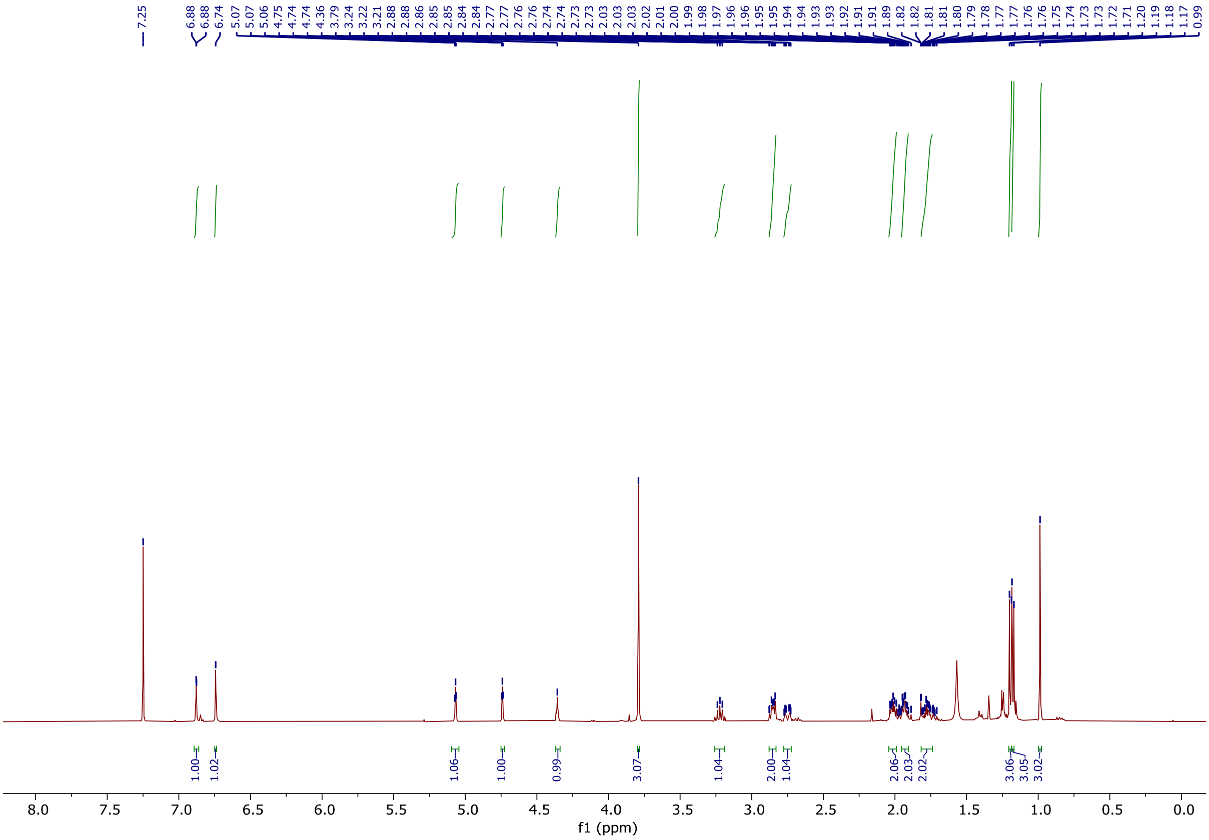

^{1}H NMR (400 MHz, $CDCl_3$) of compound (+)-**16**

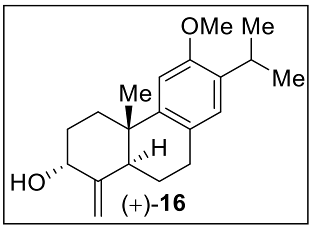

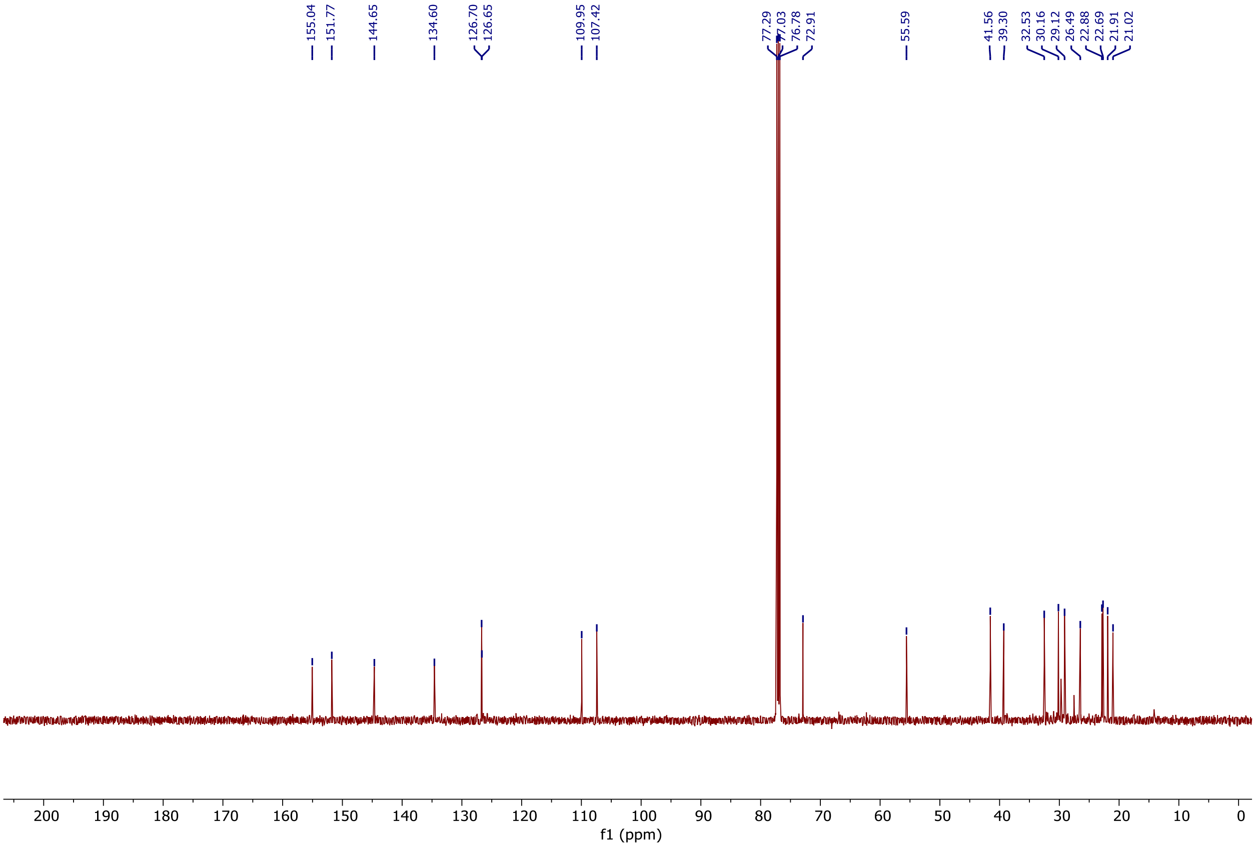

^{13}C NMR (126 MHz, $CDCl_3$) of compound (+)-**16**

Display Report

Analysis Info

Analysis Name	D:\Data\User data\2022\AUGUST\AB_SK_01_86.d	Acquisition Date	8/4/2022 11:17:07 AM
Method	Tune_pos_Standard_July2022.m	Operator	IISER Kolkata
Sample Name	AB_SK_01_86	Instrument	maXis impact 8282001.00127
Comment			

Acquisition Parameter

Source Type	ESI	Ion Polarity	Positive	Set Nebulizer	0.4 Bar
Focus	Active	Set Capillary	4500 V	Set Dry Heater	200 °C
Scan Begin	50 m/z	Set End Plate Offset	-500 V	Set Dry Gas	4.0 l/min
Scan End	1000 m/z	Set Charging Voltage	2000 V	Set Divert Valve	Source
		Set Corona	0 nA	Set APCI Heater	0 °C

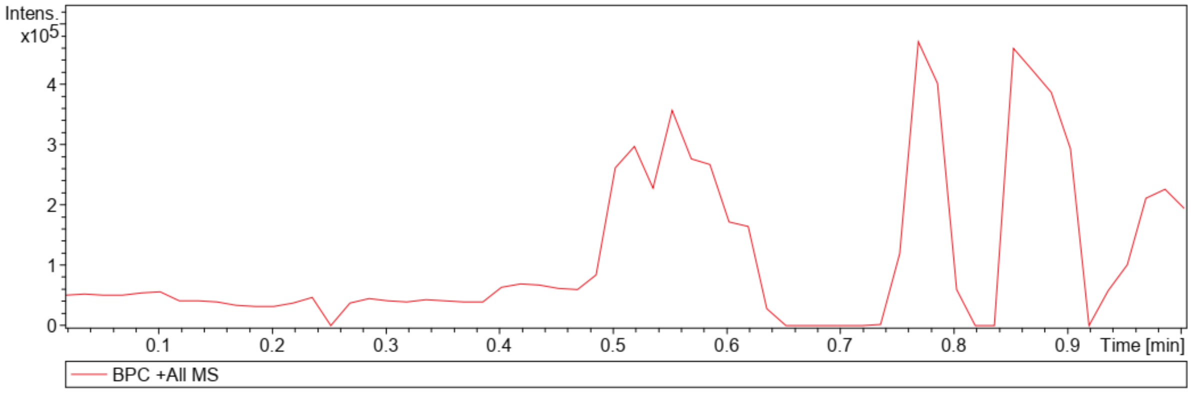

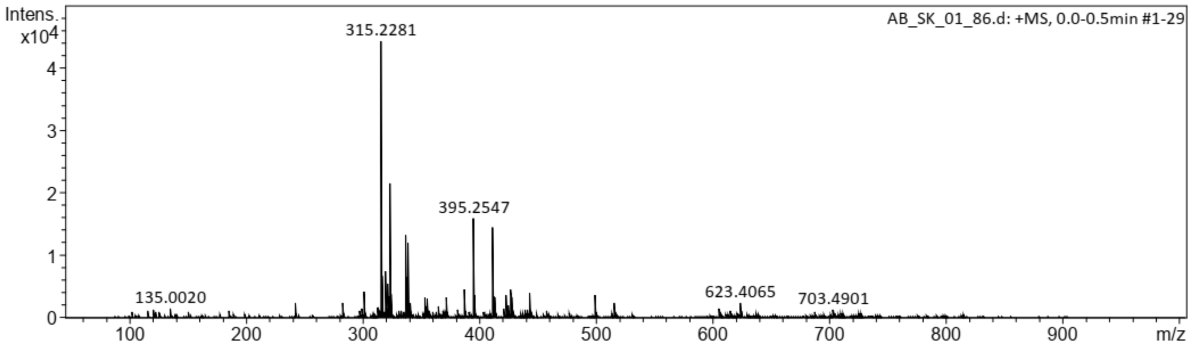

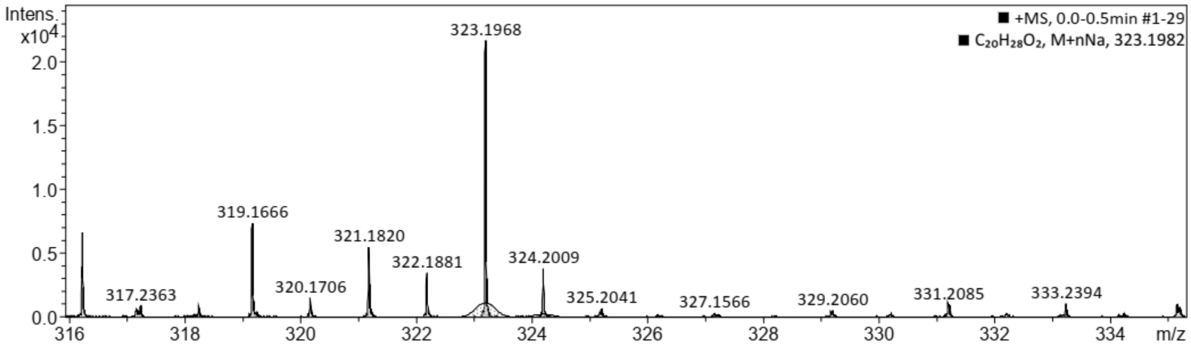

HRMS data of (+)-**16**

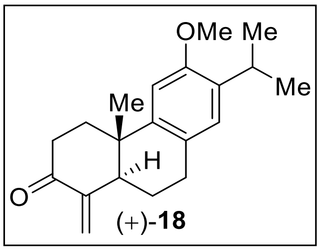

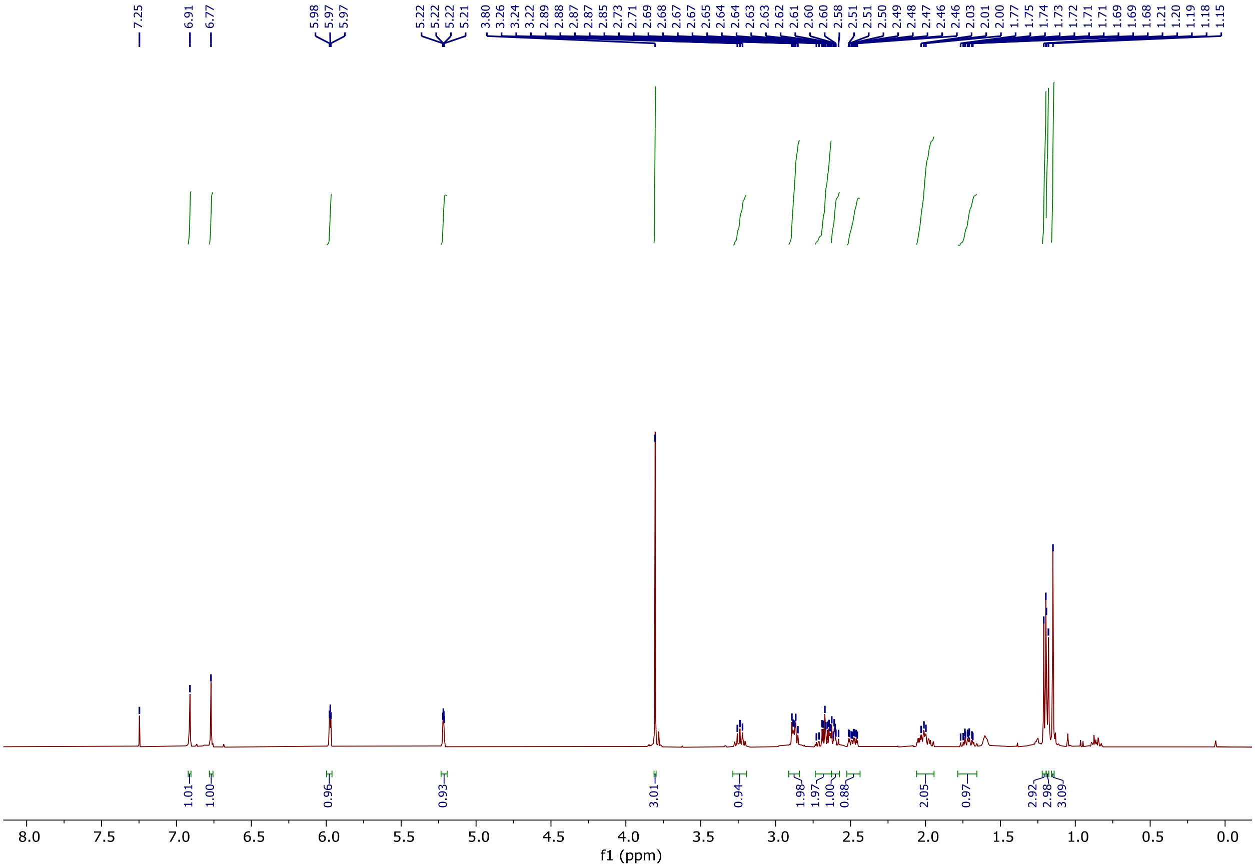

^{1}H NMR (500 MHz, $CDCl_3$) of compound (+)-**18**

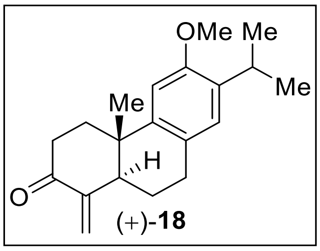

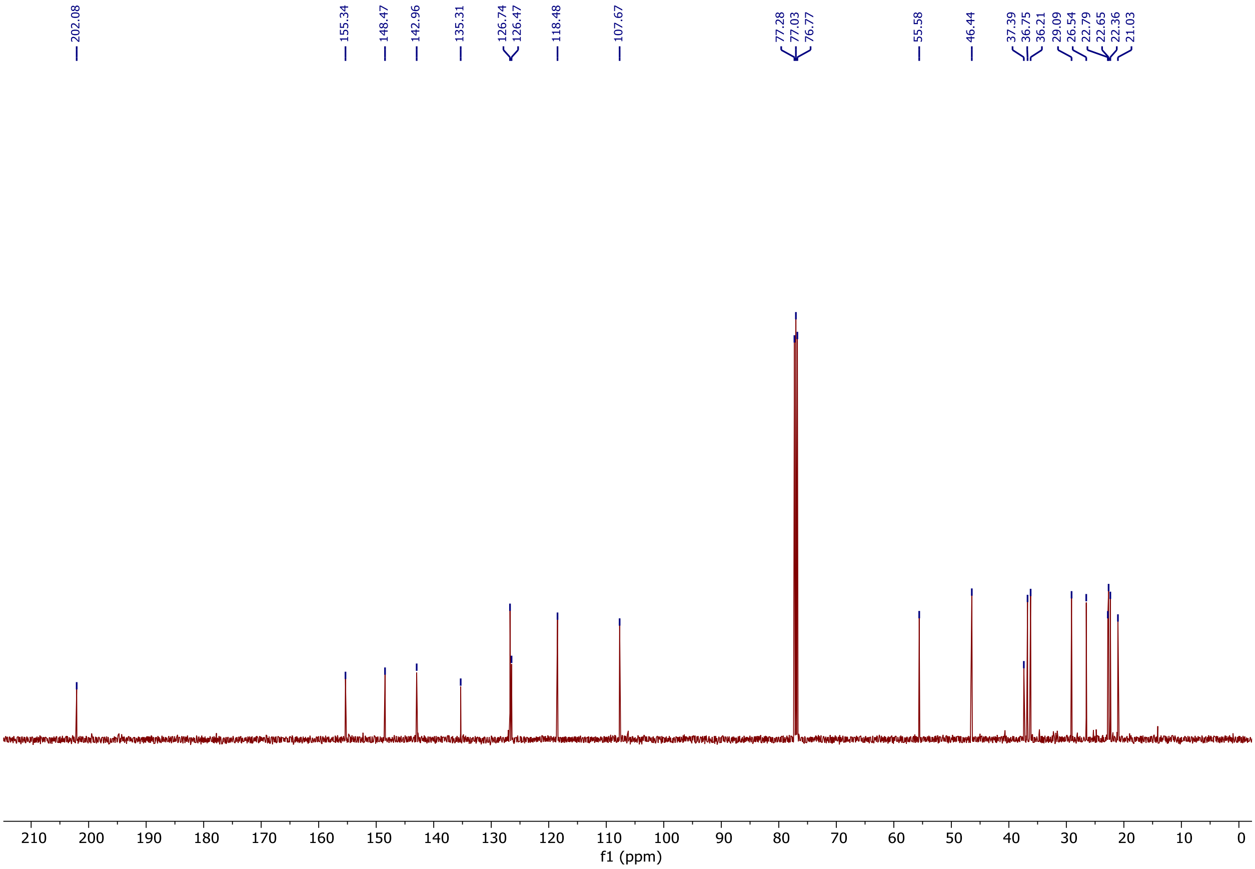

^{13}C NMR (126 MHz, $CDCl_3$) of compound (+)-**18**

Display Report

Analysis Info

Analysis Name	D:\Data\USER DATA 2022\JUNE2022\28-06-22\Prof A Bisai-SK-01-88.d	Acquisition Date	28-06-2022 12:03:16
Method	tune mix_low.New.021117_1.m	Operator	Bruker
Sample Name	SK-01-88	Instrument	micrOTOF-Q 10330
Comment			

Acquisition Parameter

Source Type	ESI	Ion Polarity	Positive	Set Nebulizer	0.4 Bar
Focus	Not active	Set Capillary	4600 V	Set Dry Heater	180 °C
Scan Begin	50 m/z	Set End Plate Offset	-500 V	Set Dry Gas	4.0 l/min
Scan End	3000 m/z	Set Collision Cell RF	100.0 Vpp	Set Divert Valve	Source

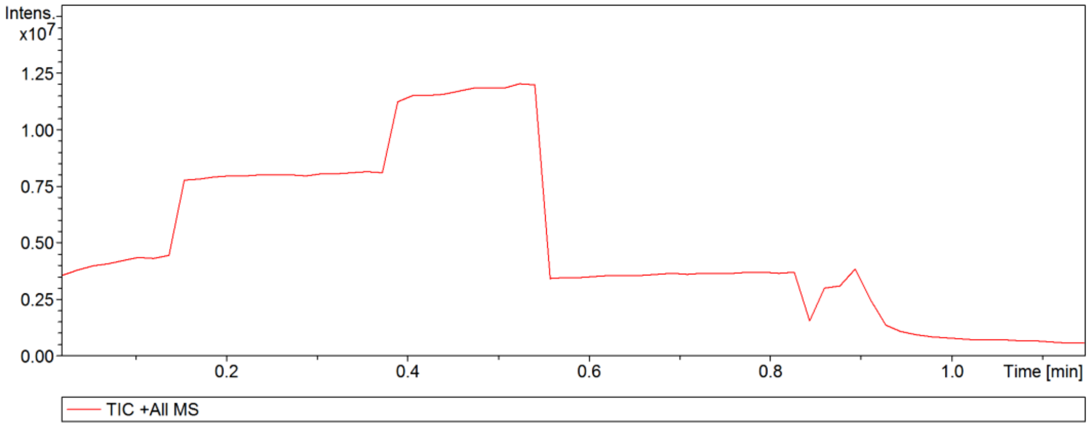

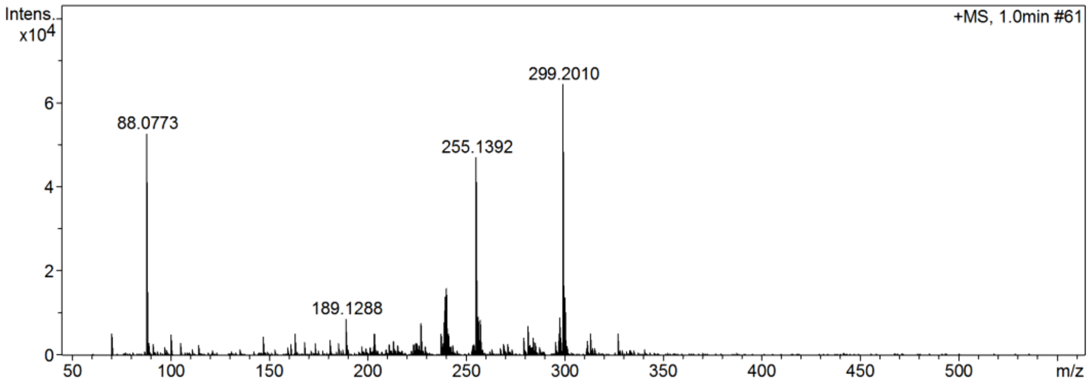

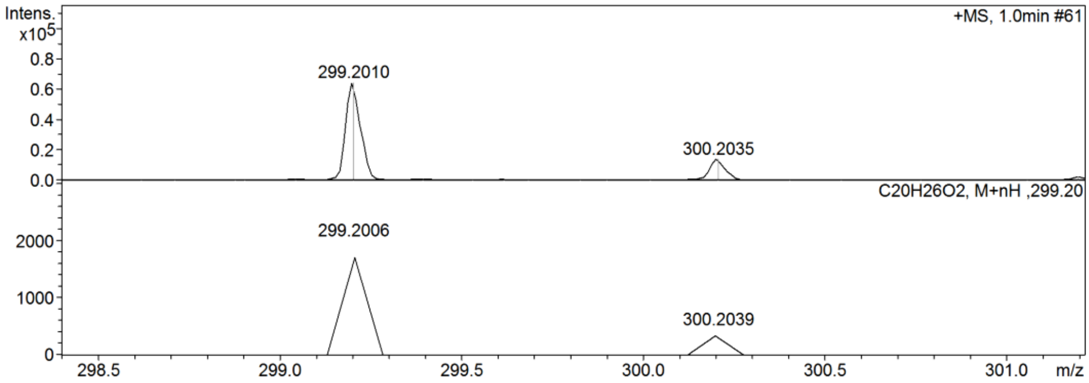

HRMS data of (+)-**18**

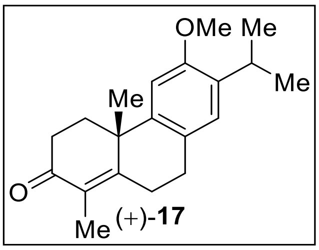

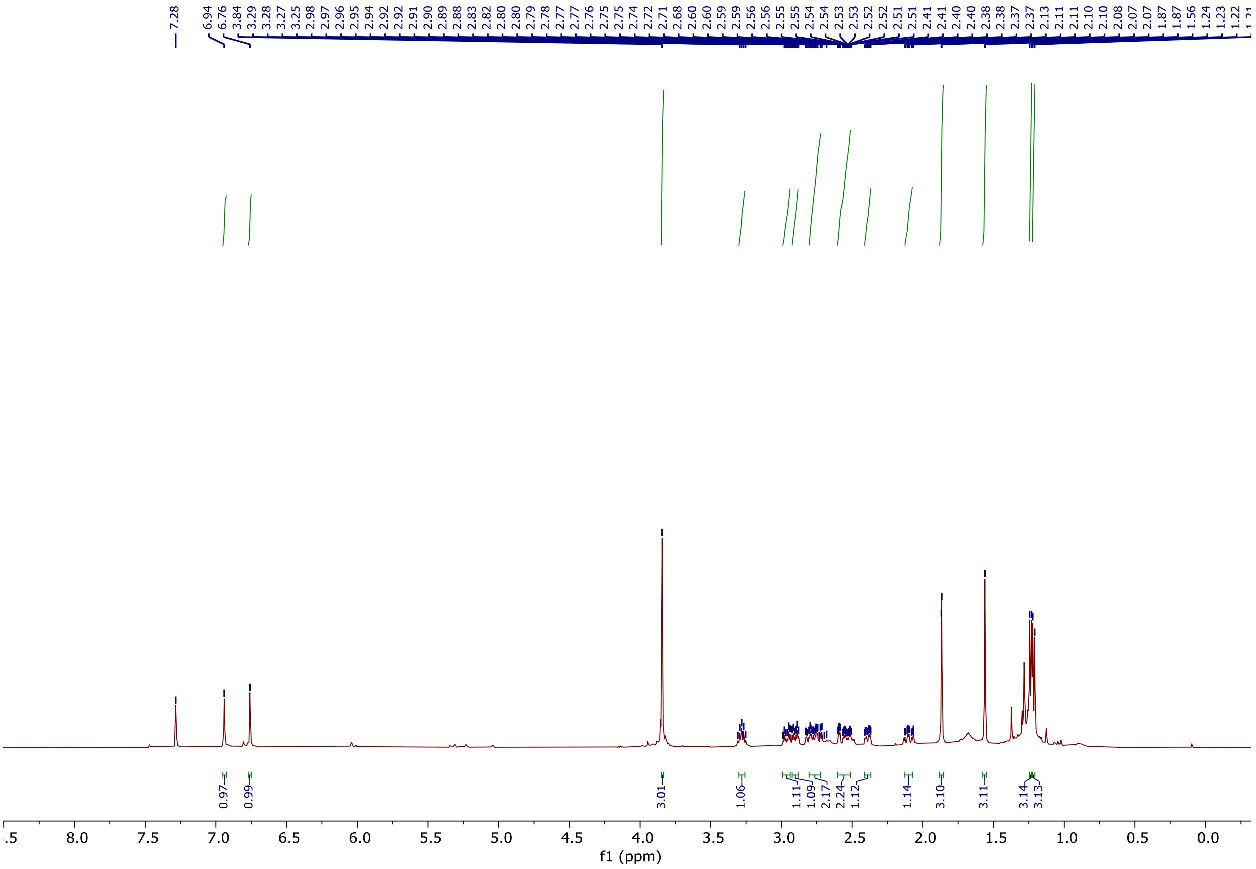

^{1}H NMR (500 MHz, $CDCl_3$) of compound (+)-**17**

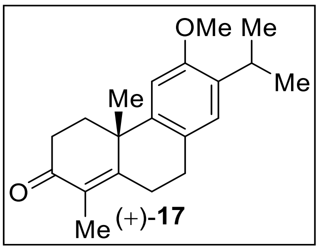

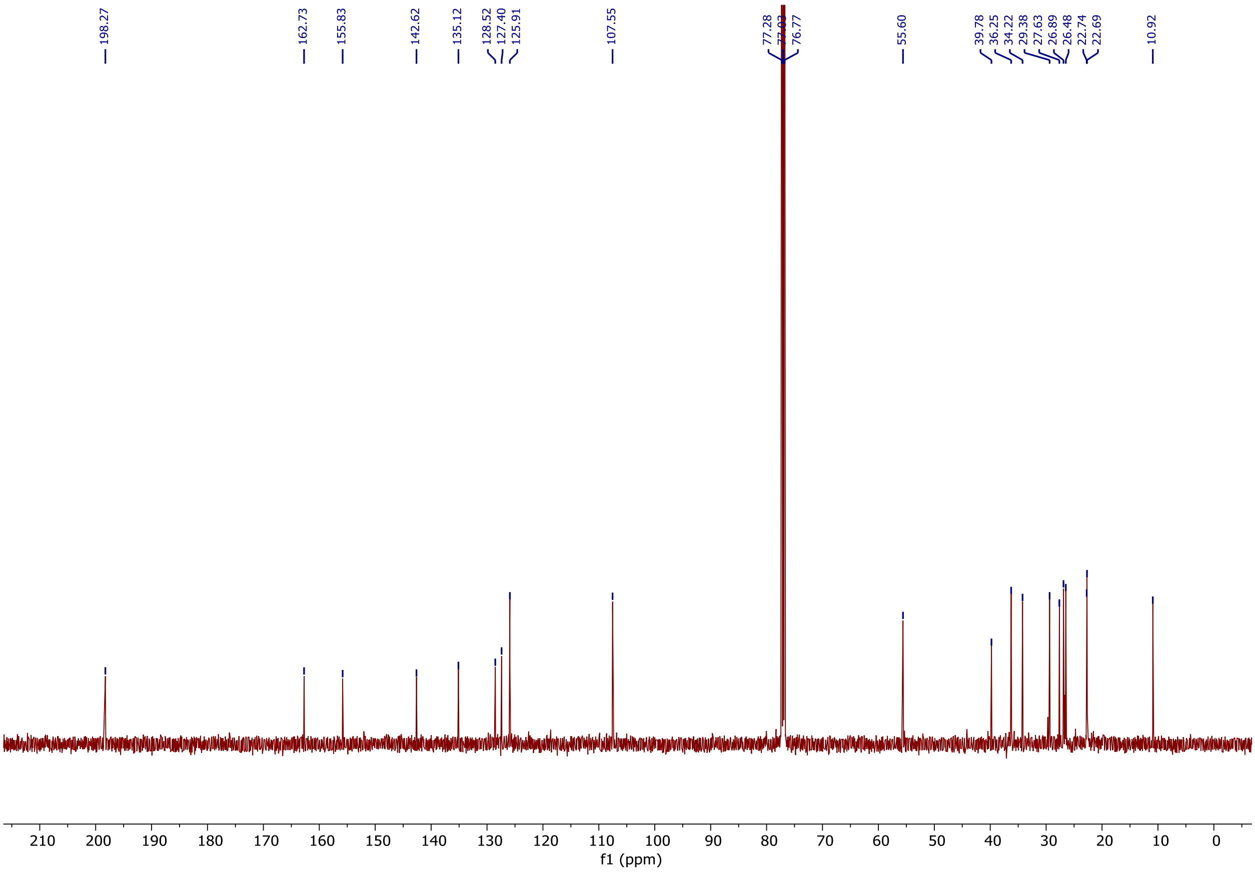

^{13}C NMR (126 MHz, $CDCl_3$) of compound (+)-**17**

Display Report

Analysis Info

Analysis Name	D:\Data\User data\2022\AUGUST\ab-sk-01-88p2.d	Acquisition Date	8/12/2022 4:09:02 PM
Method	Tune_pos_Standard_July2022.m	Operator	IISER Kolkata
Sample Name	ab-sk-01-88p2	Instrument	maXis impact 8282001.00127
Comment			

Acquisition Parameter

Source Type	ESI	Ion Polarity	Positive	Set Nebulizer	0.4 Bar
Focus	Active	Set Capillary	4500 V	Set Dry Heater	200 °C
Scan Begin	50 m/z	Set End Plate Offset	-500 V	Set Dry Gas	4.0 l/min
Scan End	1000 m/z	Set Charging Voltage	2000 V	Set Divert Valve	Source
		Set Corona	0 nA	Set APCI Heater	0 °C

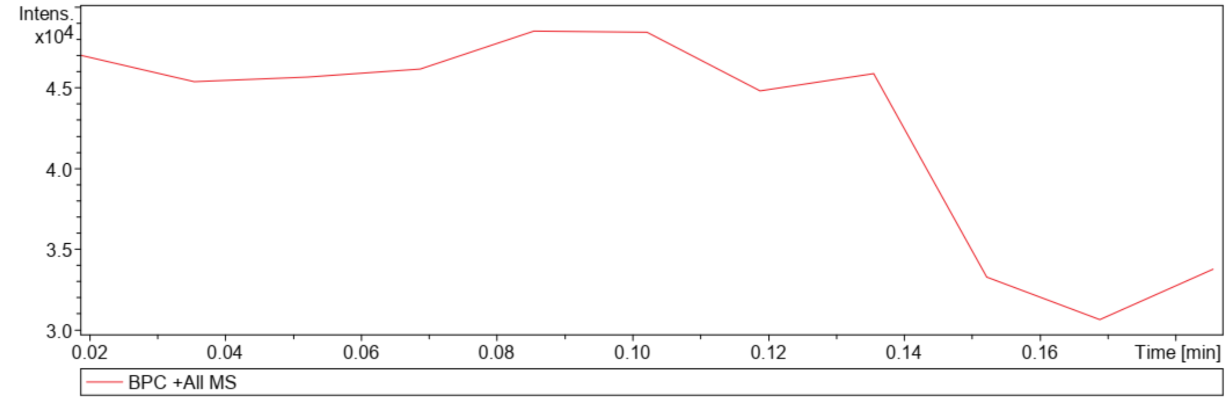

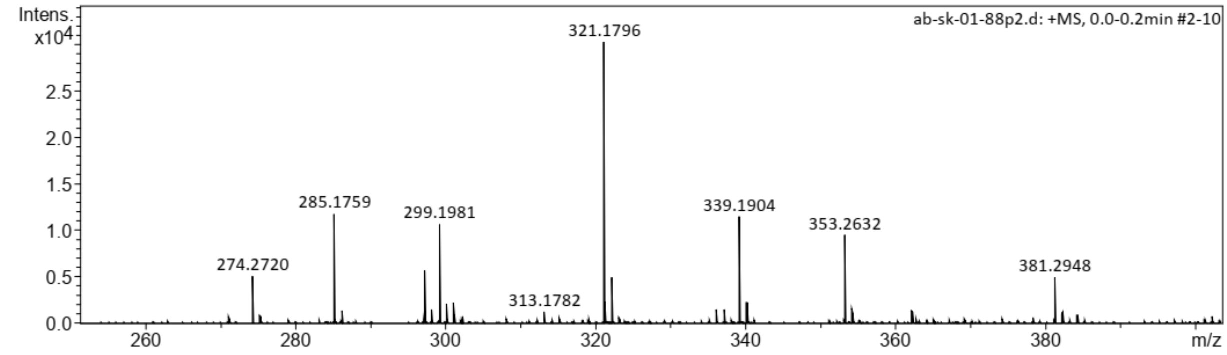

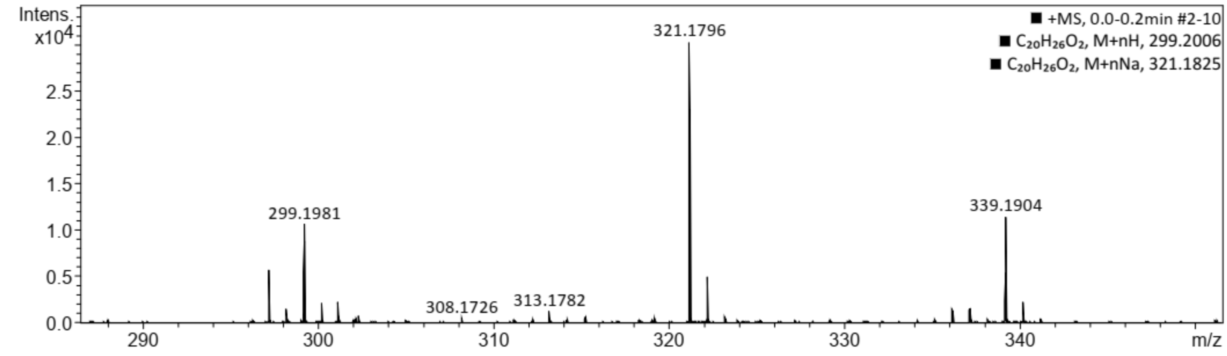

HRMS data of (+)-**17**

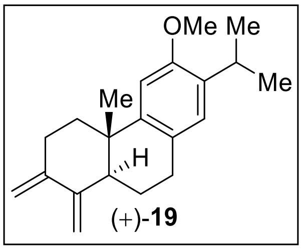

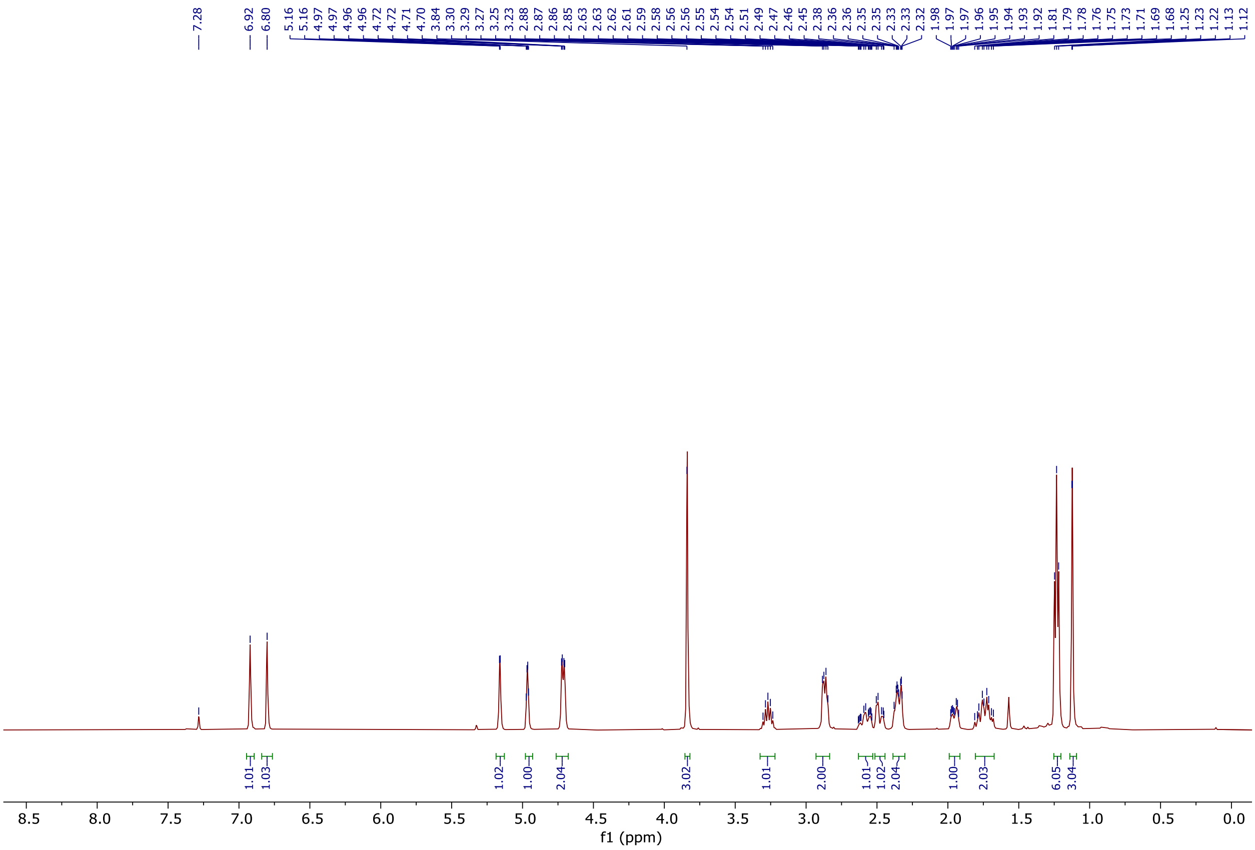

^{1}H NMR (400 MHz, $CDCl_3$) of compound (+)-**19**

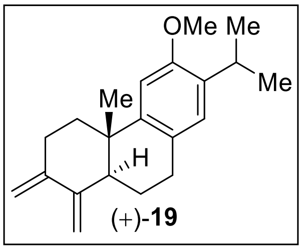

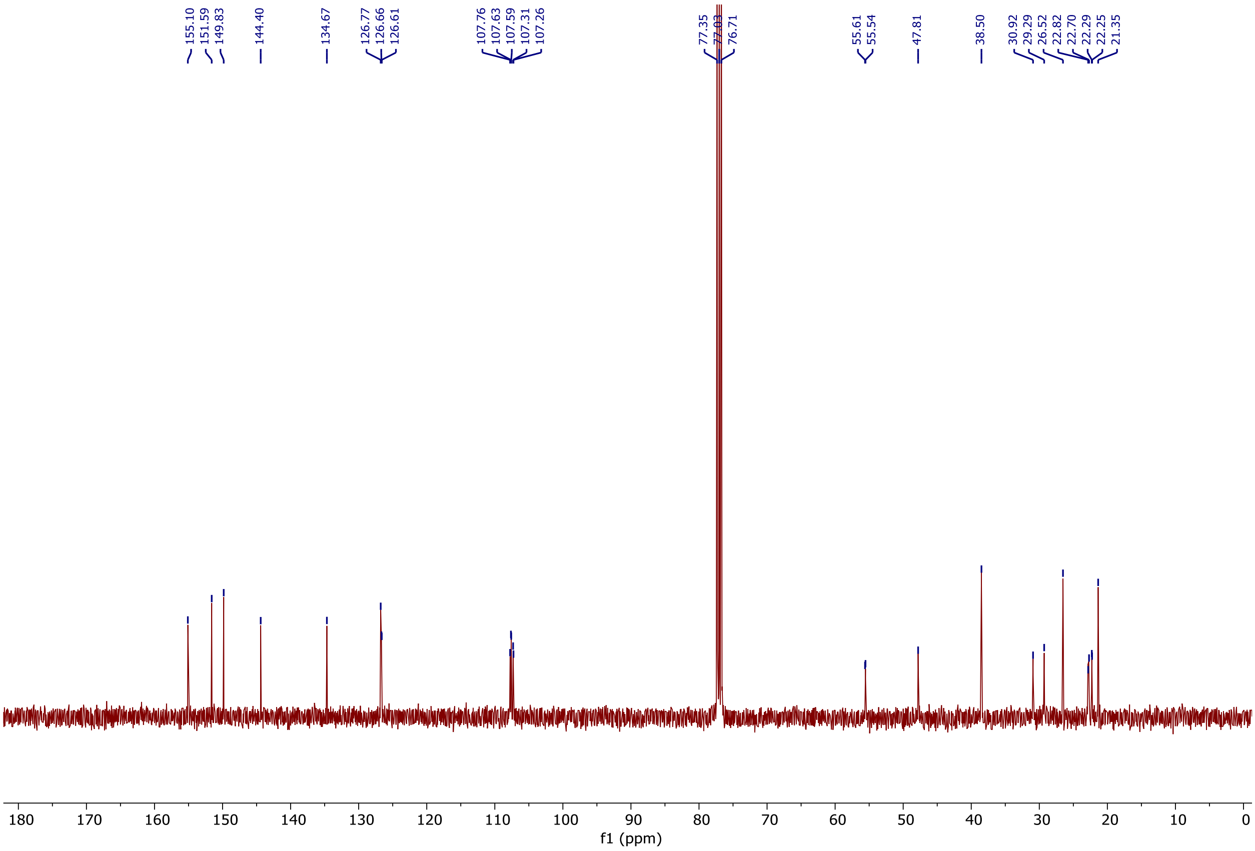

^{13}C NMR (101 MHz, $CDCl_3$) of compound (+)-**19**

Display Report

Analysis Info

Analysis Name	D:\Data\USER DATA 2022\JULY2022\19-07-22\Dr.A.Bisai-ABSK0266.d	Acquisition Date	19-07-2022 14:23:57
Method	tune mix_low.New.021117.m	Operator	Bruker
Sample Name	ABSK0266	Instrument	micrOTOF-Q 10330
Comment			

Acquisition Parameter

Source Type	ESI	Ion Polarity	Positive	Set Nebulizer	0.4 Bar
Focus	Not active	Set Capillary	4600 V	Set Dry Heater	180 °C
Scan Begin	50 m/z	Set End Plate Offset	-500 V	Set Dry Gas	4.0 l/min
Scan End	3000 m/z	Set Collision Cell RF	100.0 Vpp	Set Divert Valve	Source

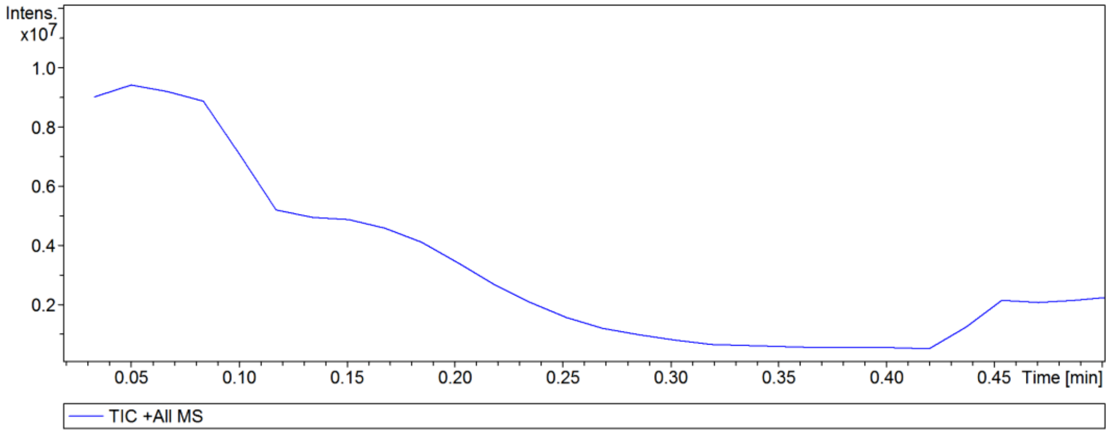

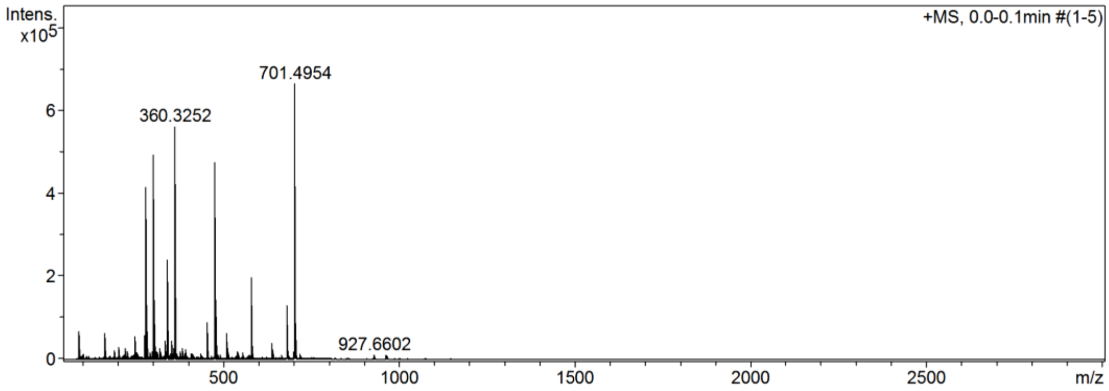

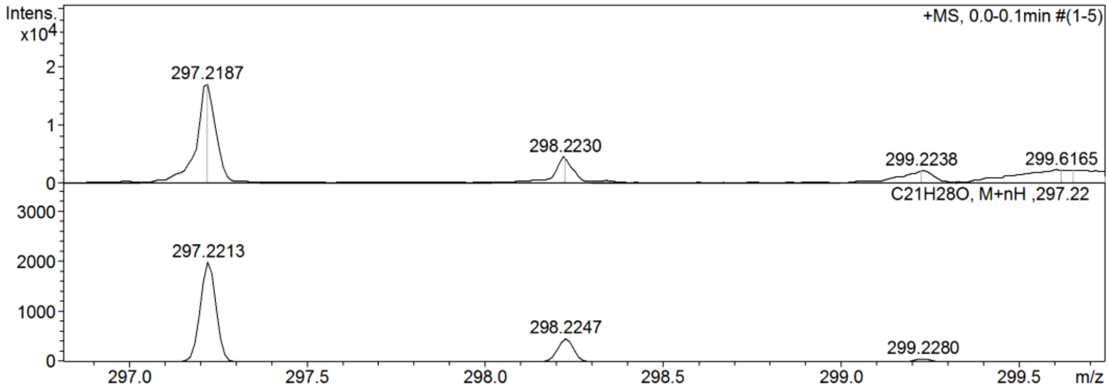

HRMS data of (+)-**19**

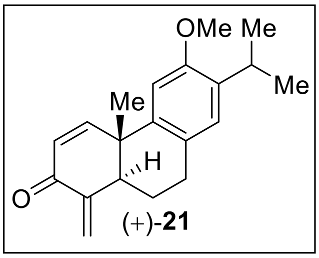

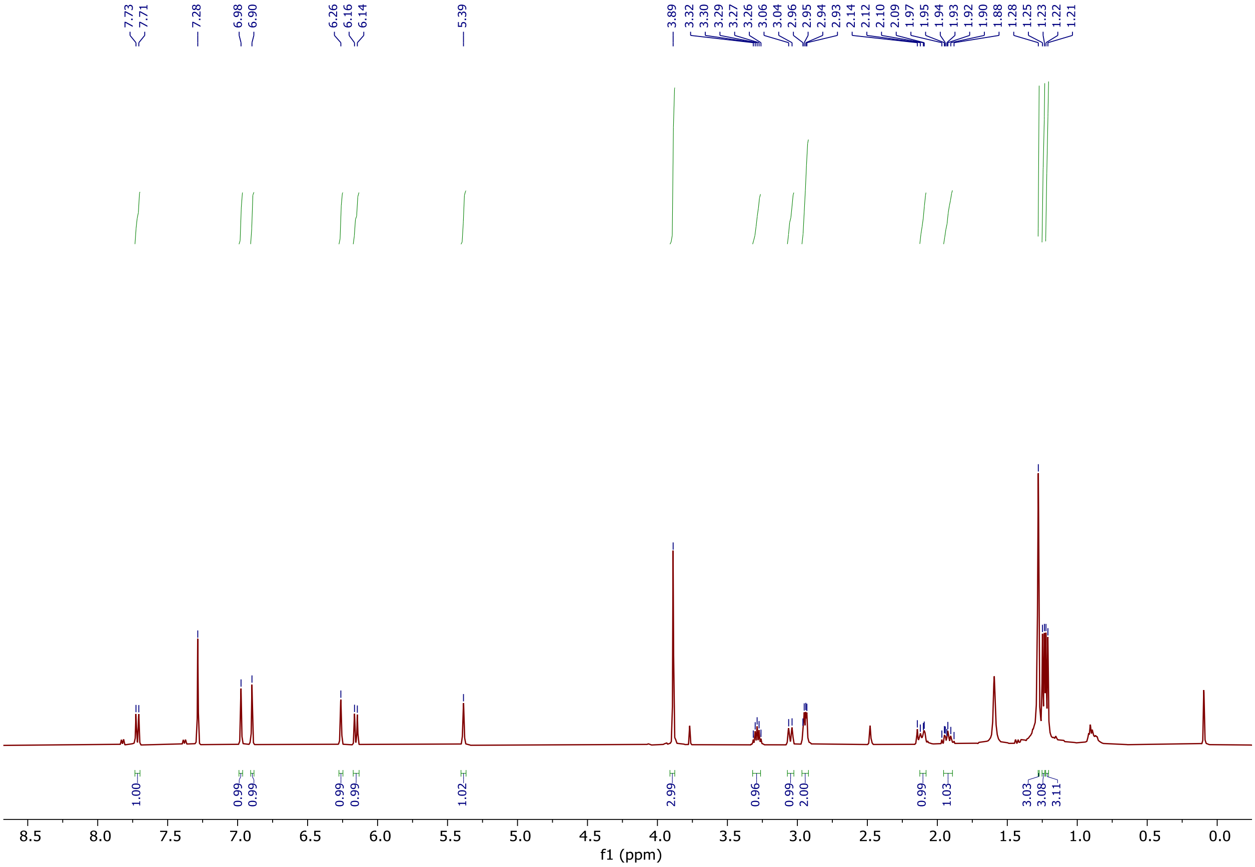

^{1}H NMR (400 MHz, $CDCl_3$) of compound (+)-**21**

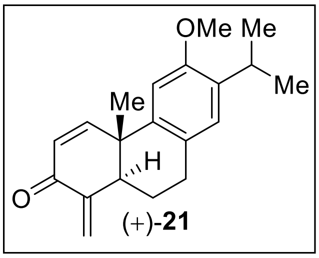

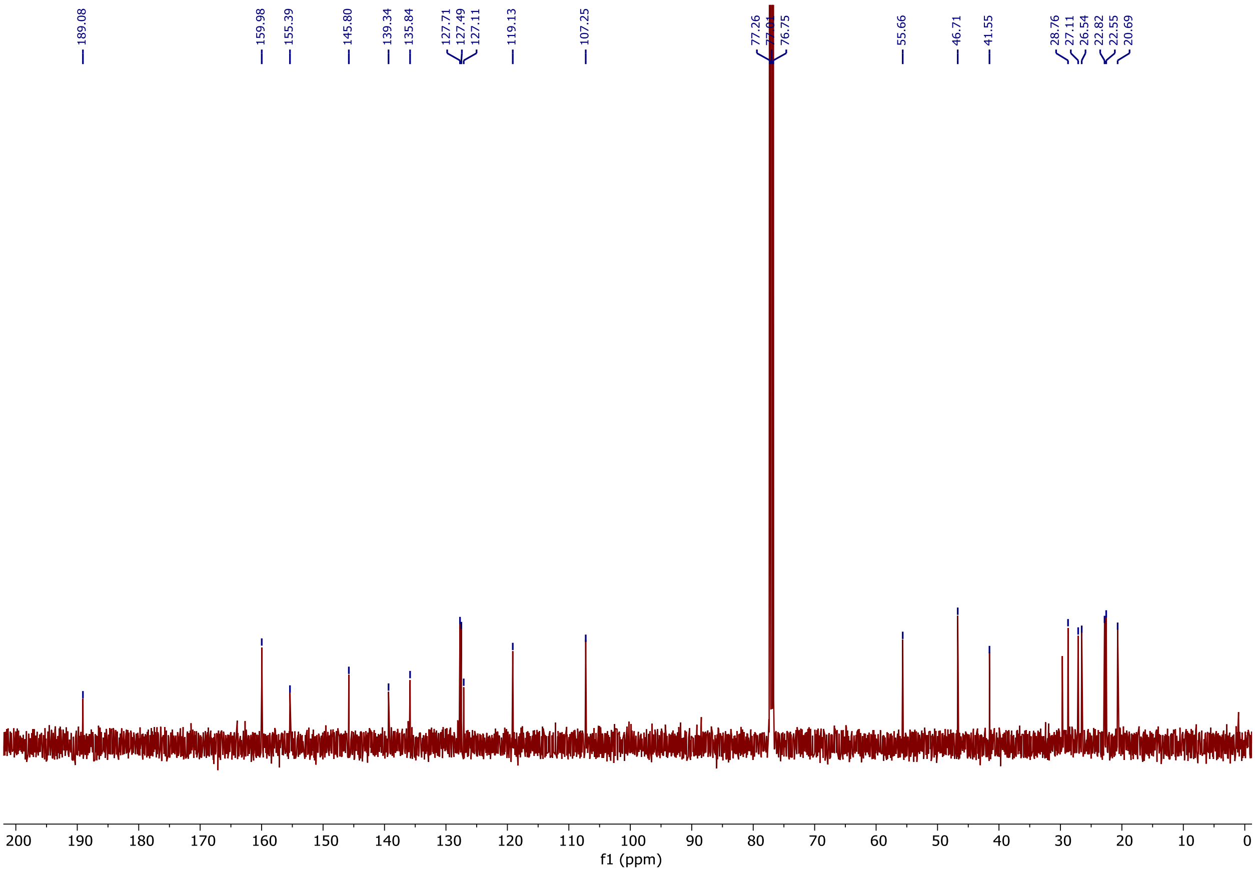

^{13}C NMR (126 MHz, $CDCl_3$) of compound (+)-**21**

Display Report

Analysis Info

Analysis Name	D:\Data\USER DATA 2022\JULY2022\19-07-22\Dr.A.Bisai-ABSK0260.d	Acquisition Date	19-07-2022 14:22:12
Method	tune mix_low.New.021117.m	Operator	Bruker
Sample Name	ABSK0260	Instrument	micrOTOF-Q 10330
Comment			

Acquisition Parameter

Source Type	ESI	Ion Polarity	Positive	Set Nebulizer	0.4 Bar
Focus	Not active	Set Capillary	4600 V	Set Dry Heater	180 °C
Scan Begin	50 m/z	Set End Plate Offset	-500 V	Set Dry Gas	4.0 l/min
Scan End	3000 m/z	Set Collision Cell RF	100.0 Vpp	Set Divert Valve	Source

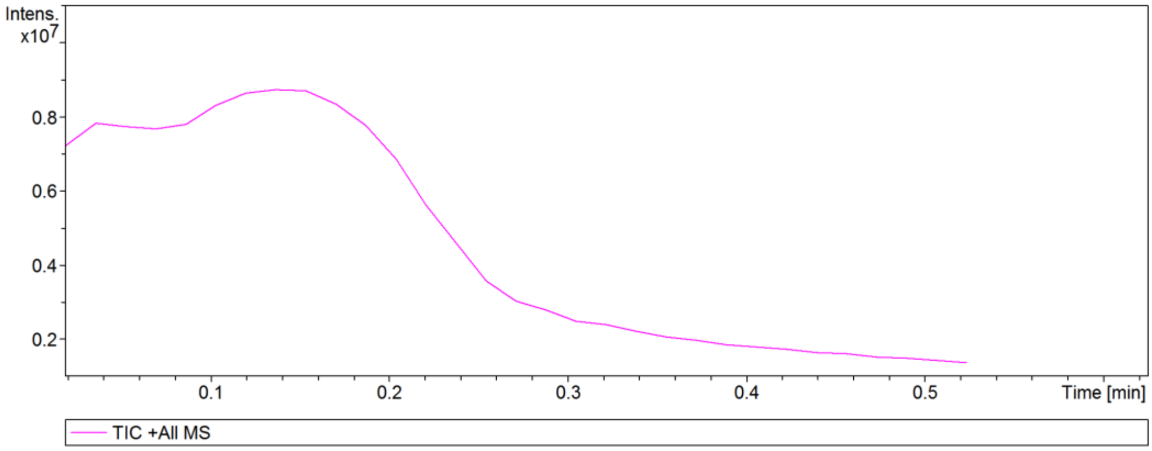

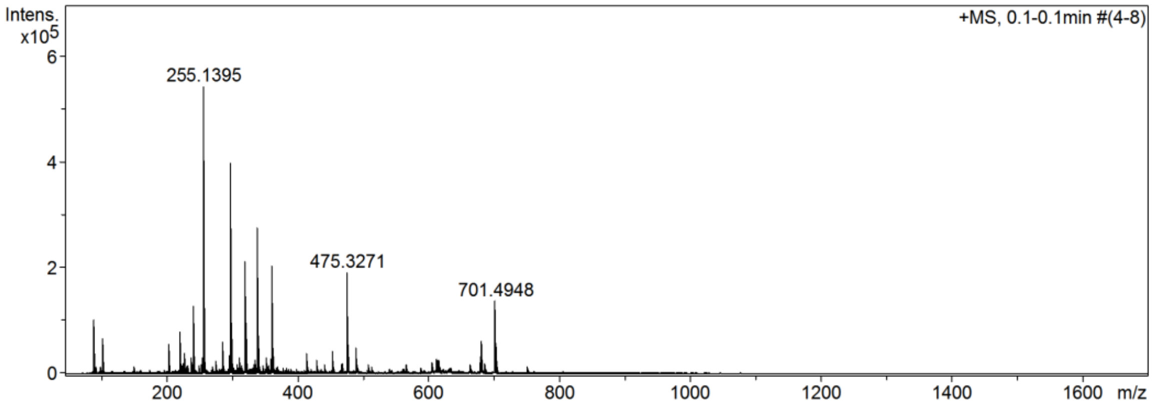

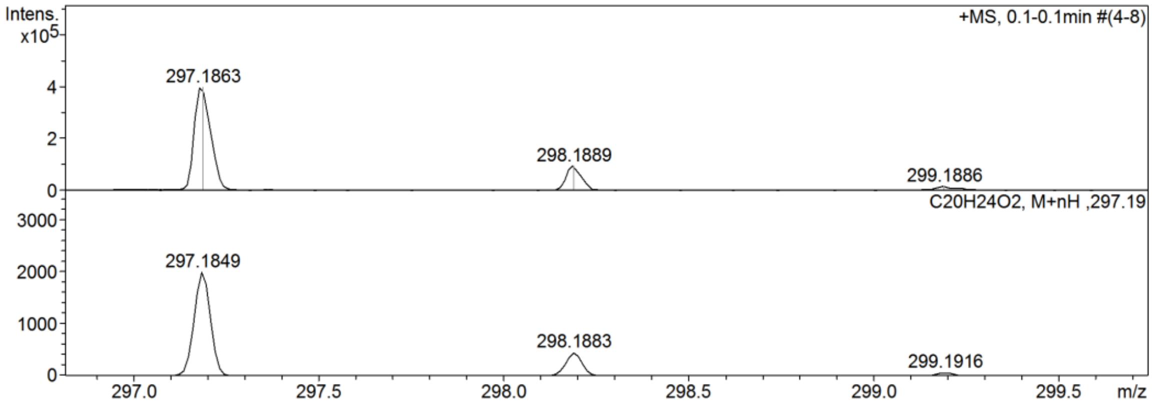

HRMS data of (+)-**21**

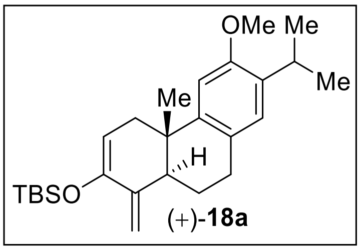

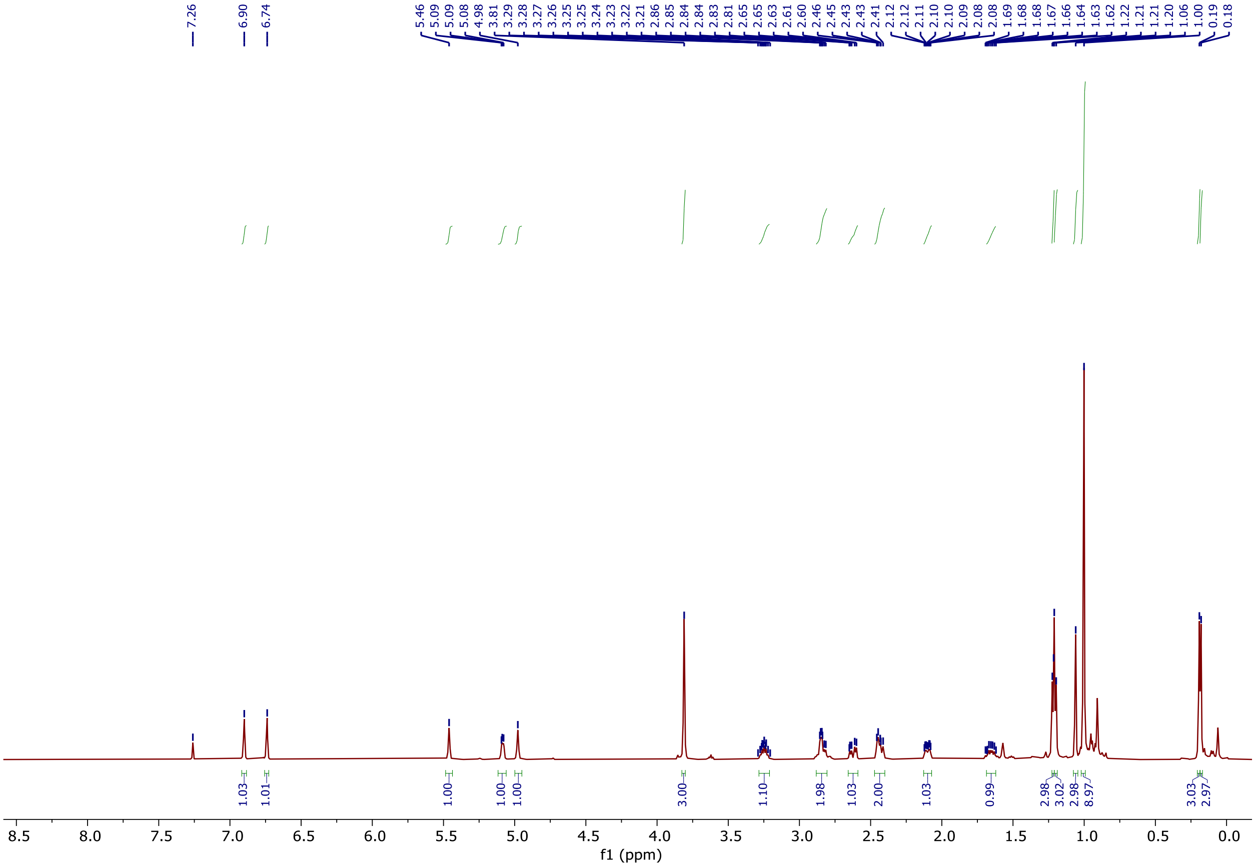

^{1}H NMR (500 MHz, $CDCl_3$) of compound (+)-**18a**

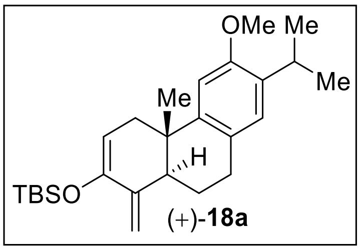

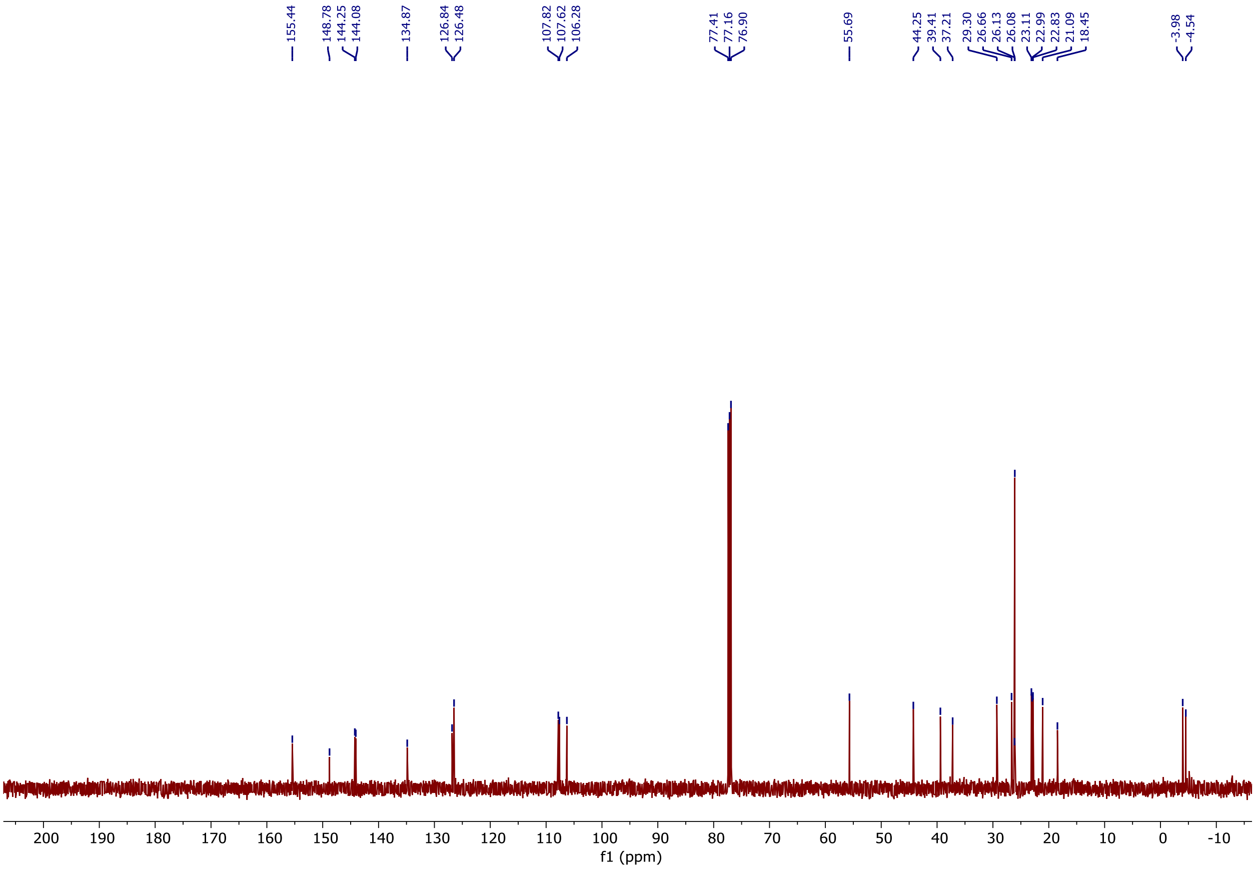

^{13}C NMR (126 MHz, $CDCl_3$) of compound (+)-**18a**

Display Report

Analysis Info

Analysis Name	D:\Data\USER DATA 2022\JUNE2022\28-06-22\Prof A Bisai-SK-02-61.d	Acquisition Date	28-06-2022 11:15:59
Method	tune mix_low.New.021117_1.m	Operator	Bruker
Sample Name	SK-02-61	Instrument	micrOTOF-Q 10330
Comment			

Acquisition Parameter

Source Type	ESI	Ion Polarity	Positive	Set Nebulizer	0.4 Bar
Focus	Not active	Set Capillary	4600 V	Set Dry Heater	180 °C
Scan Begin	50 m/z	Set End Plate Offset	-500 V	Set Dry Gas	4.0 l/min
Scan End	3000 m/z	Set Collision Cell RF	300.0 Vpp	Set Divert Valve	Source

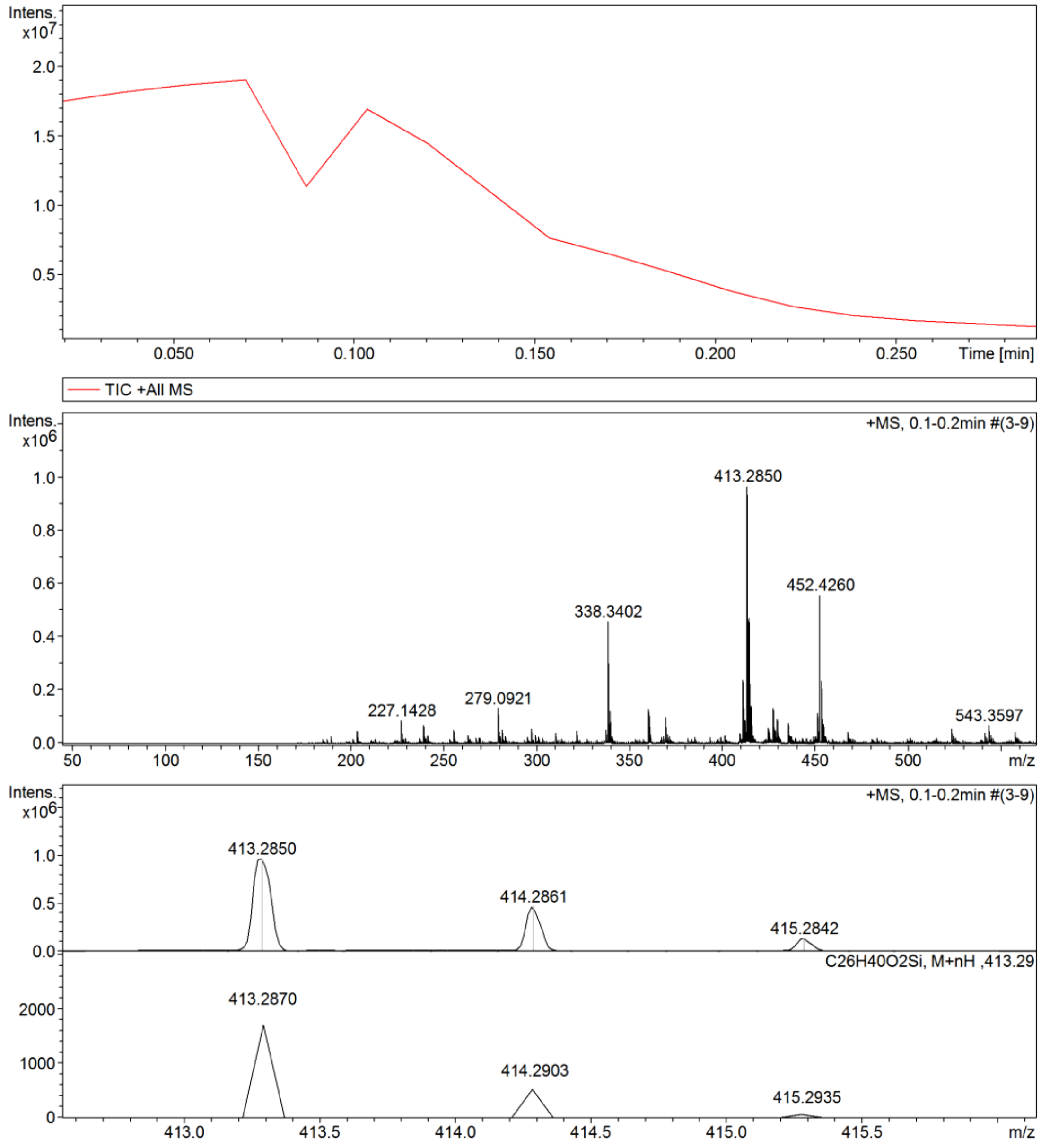

Bruker Compass DataAnalysis 4.0 printed: 28-06-2022 11:18:00 Page 1 of 1

HRMS data of (+)-**18a**

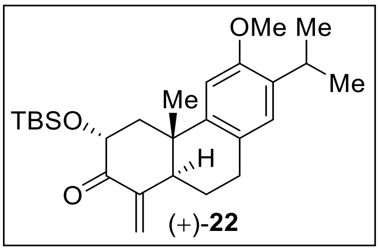

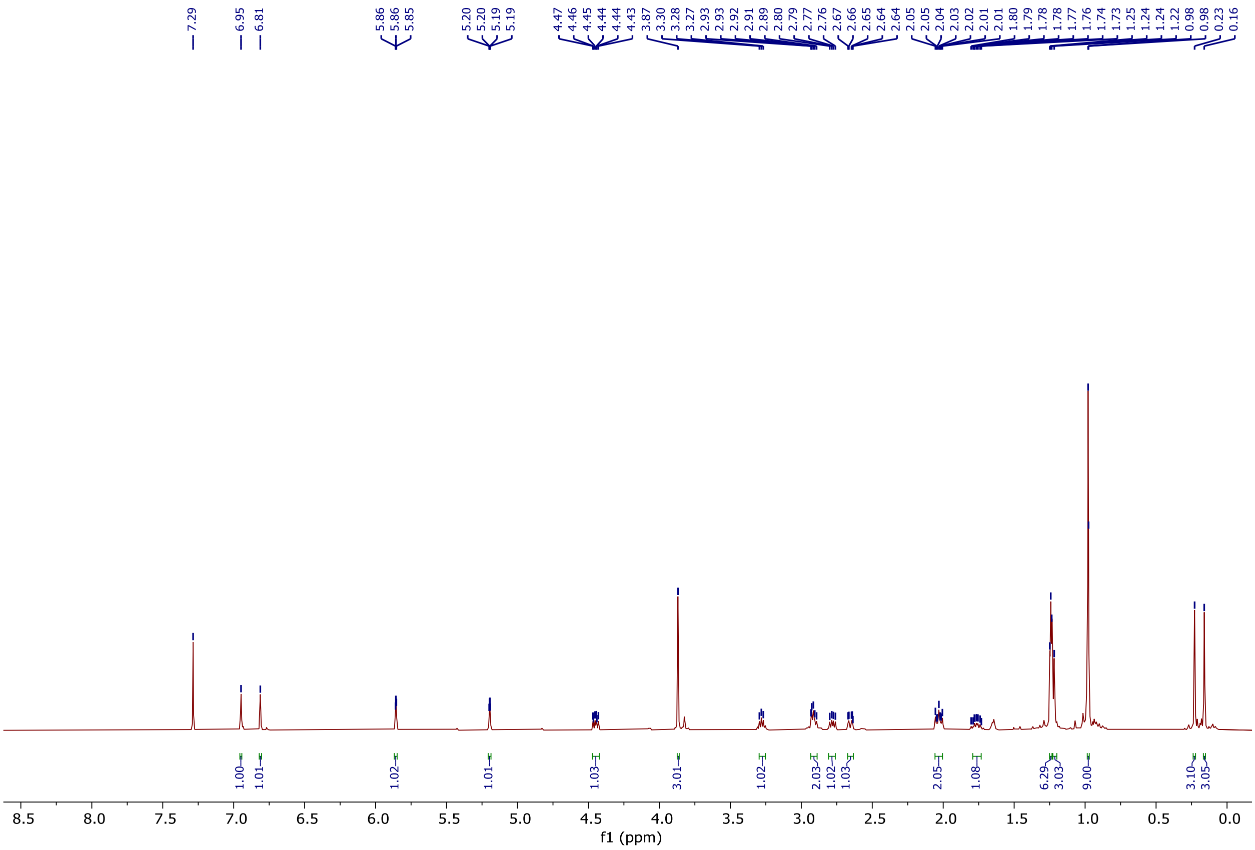

^{1}H NMR (500 MHz, $CDCl_3$) of compound (+)-**22**

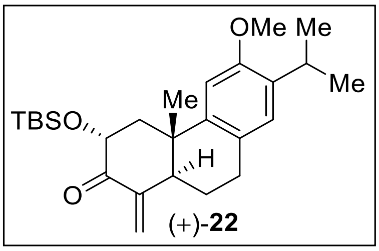

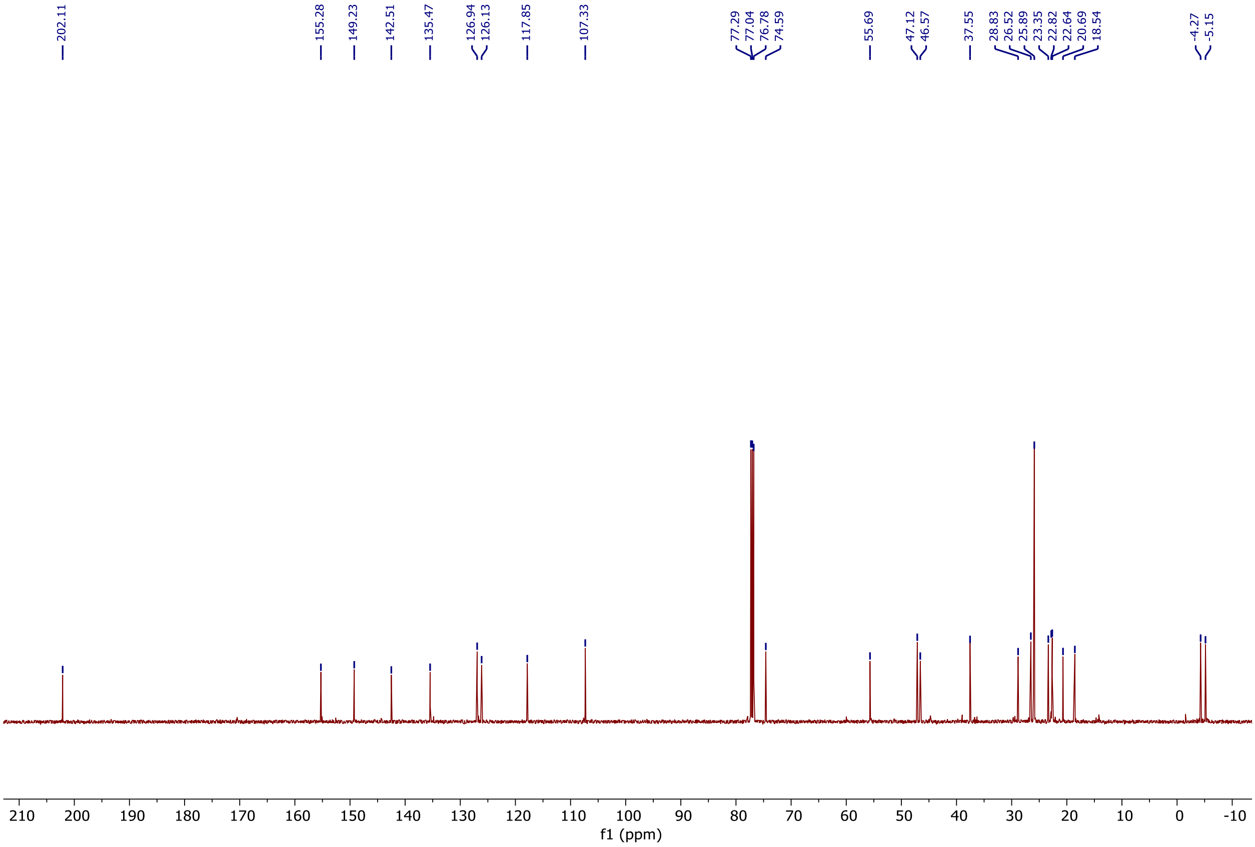

^{13}C NMR (126 MHz, $CDCl_3$) of compound (+)-**22**

Display Report

Analysis Info

Analysis Name	D:\Data\USER DATA 2022\JUNE2022\28-06-22\Prof A Bisai-SK-02-78.d	Acquisition Date	28-06-2022 11:23:57
Method	tune mix_low.New.021117_1.m	Operator	Bruker
Sample Name	SK-02-78	Instrument	micrOTOF-Q 10330
Comment			

Acquisition Parameter

Source Type	ESI	Ion Polarity	Positive	Set Nebulizer	0.4 Bar
Focus	Not active	Set Capillary	4600 V	Set Dry Heater	180 °C
Scan Begin	50 m/z	Set End Plate Offset	-500 V	Set Dry Gas	4.0 l/min
Scan End	3000 m/z	Set Collision Cell RF	300.0 Vpp	Set Divert Valve	Source

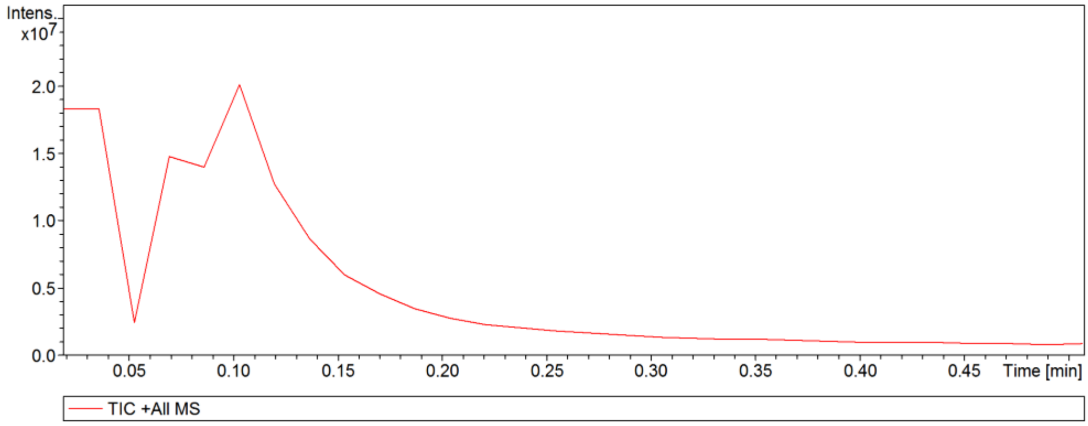

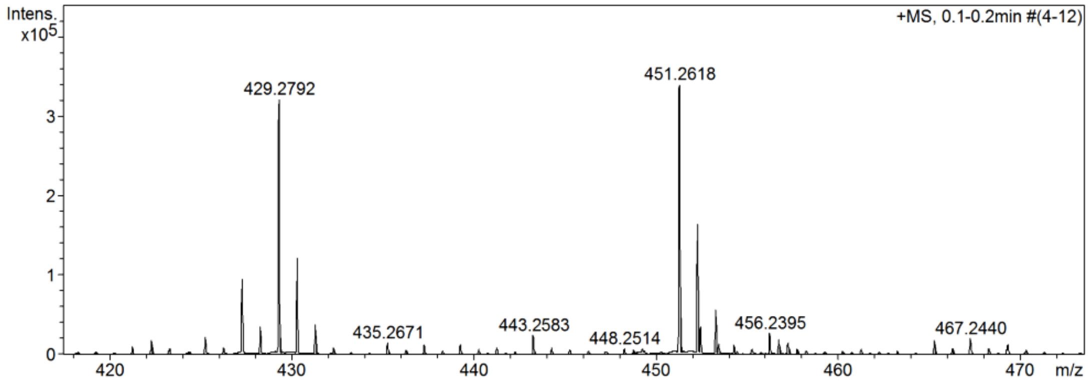

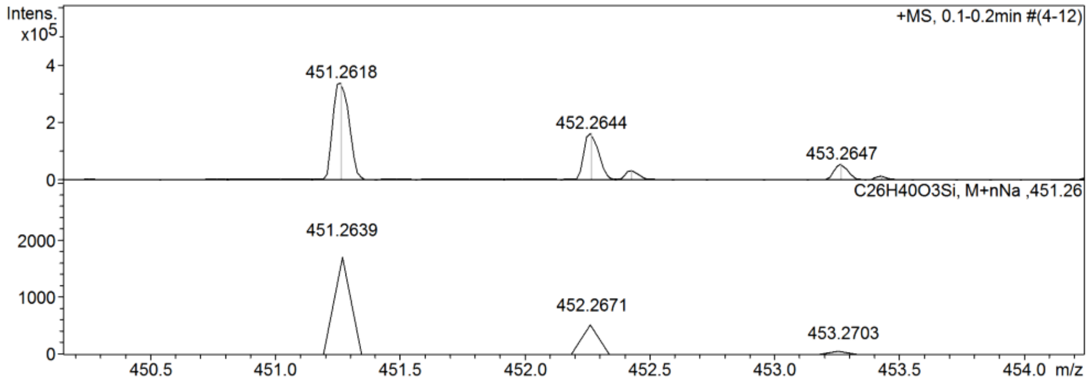

HRMS data of (+)-**22**

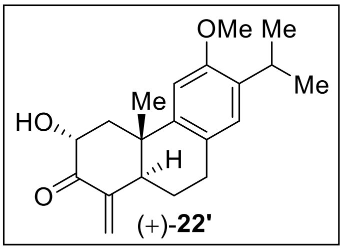

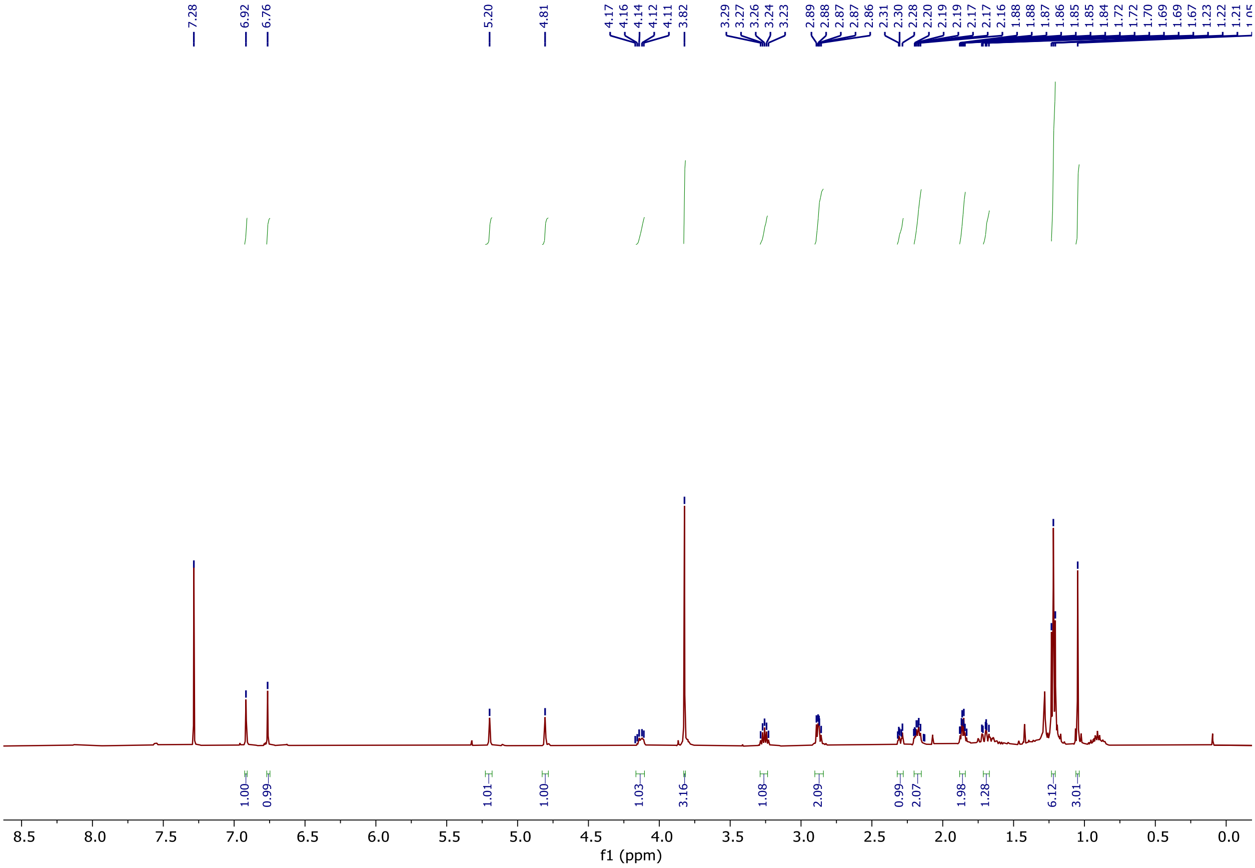

^{1}H NMR (500 MHz, $CDCl_3$) of compound (+)-**22’**

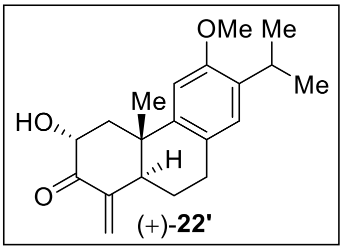

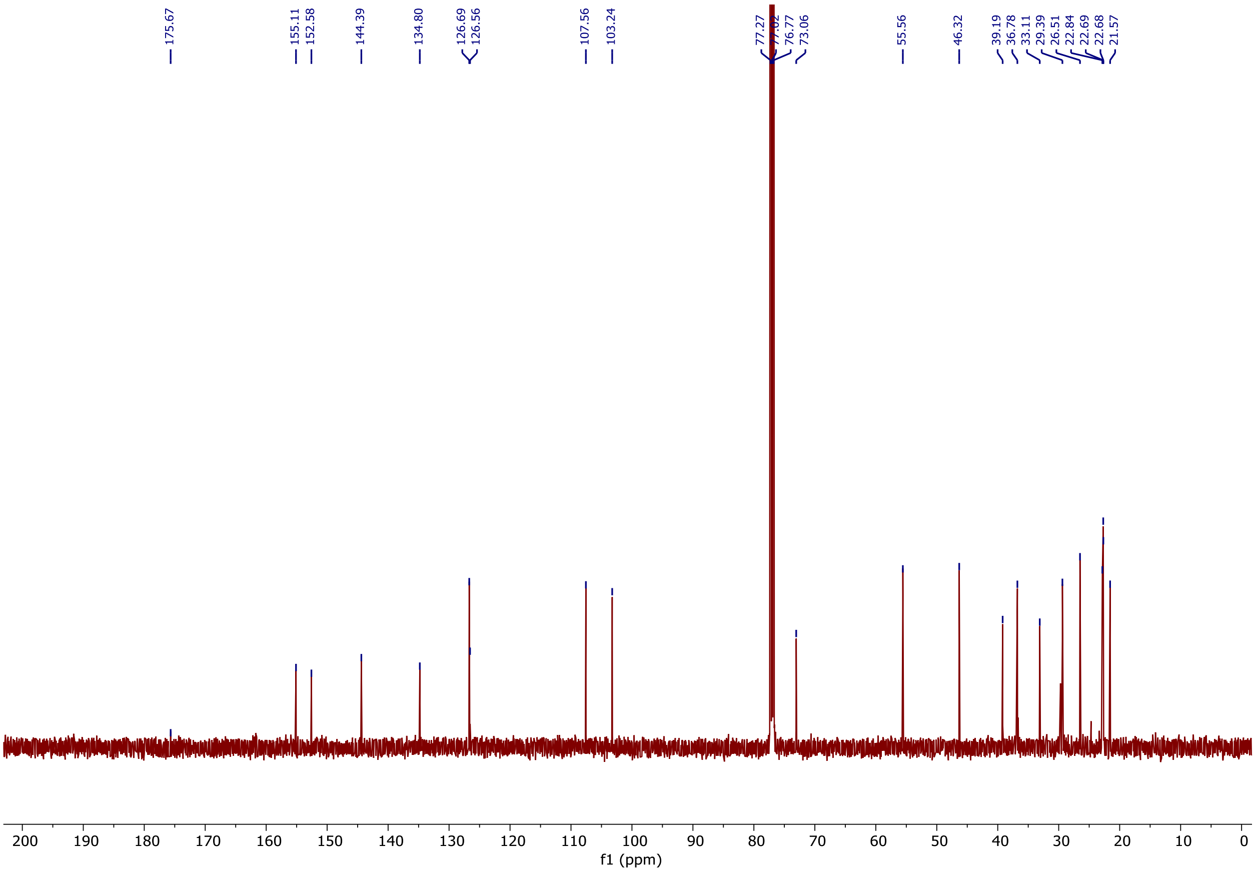

^{13}C NMR (126 MHz, $CDCl_3$) of compound (+)-**22'**

Display Report

Analysis Info

Analysis Name	D:\Data\USER DATA 2022\MAY 2022\12-05-2022\Prof.A.Bisai-ABSK0262.d	Acquisition Date	5/12/2022 3:59:21 PM
Method	tune_low_APCI.m	Operator	IISER
Sample Name	ABSK0262	Instrument	micrOTOF-Q II 10330
Comment			

Acquisition Parameter

Source Type	Multi Mode	Ion Polarity	Positive	Set Nebulizer	2.0 Bar
Focus	Not active	Set Capillary	2500 V	Set Dry Heater	200 °C
Scan Begin	50 m/z	Set End Plate Offset	-500 V	Set Dry Gas	5.0 l/min
Scan End	3000 m/z	Set Collision Cell RF	150.0 Vpp	Set Divert Valve	Source

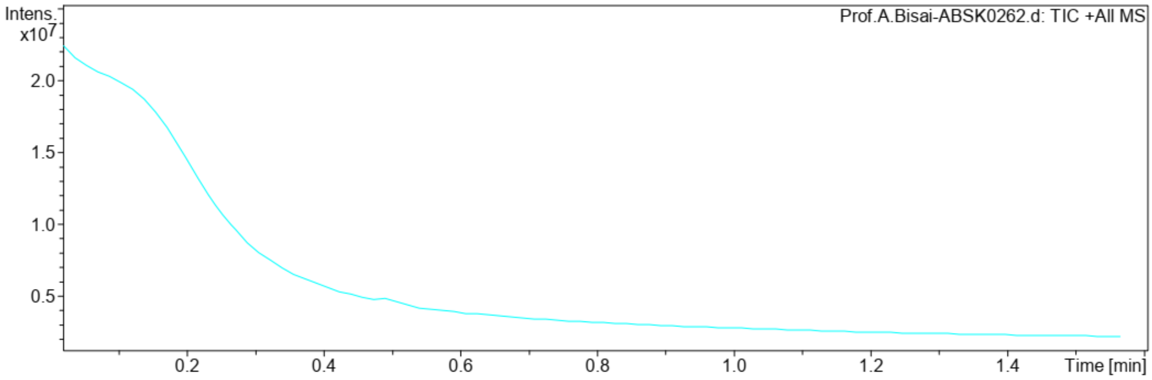

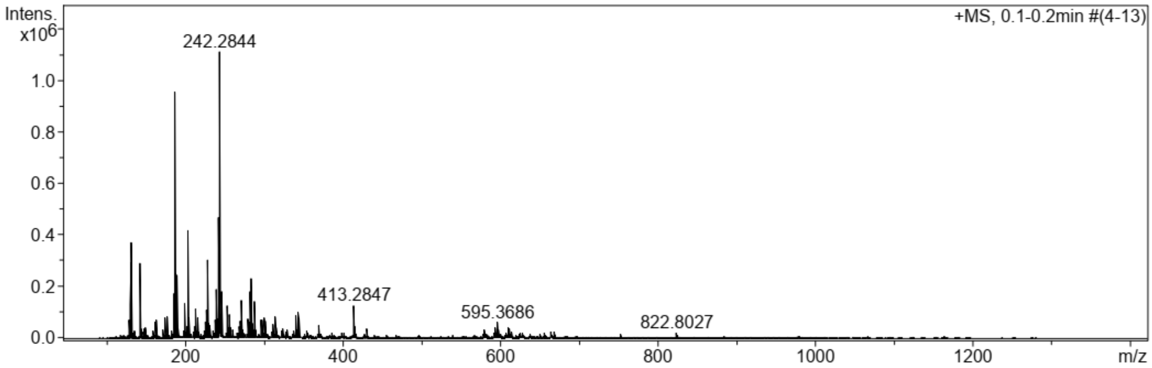

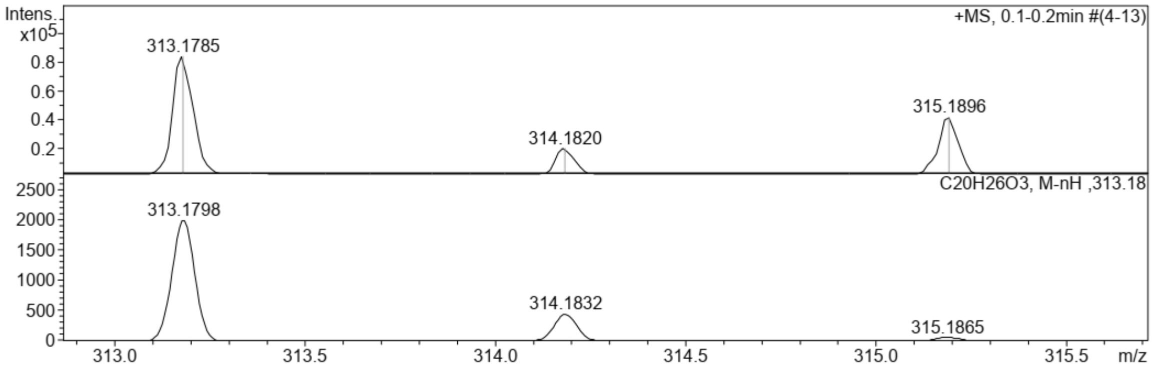

HRMS data of (+)-**22’**

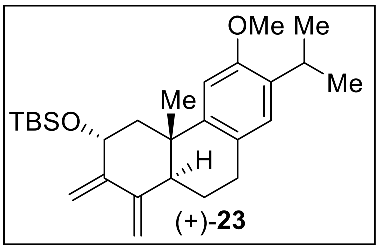

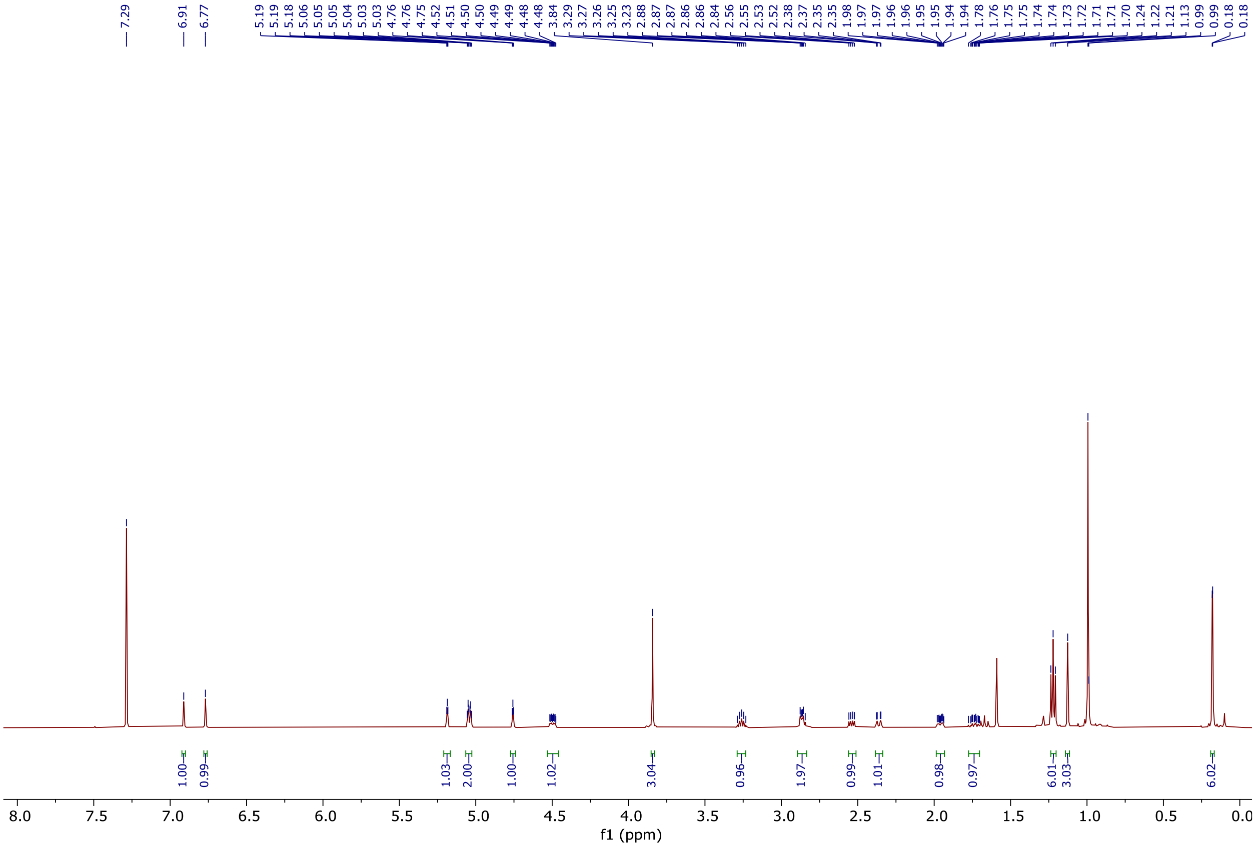

^{1}H NMR (500 MHz, $CDCl_3$) of compound (+)-**23**

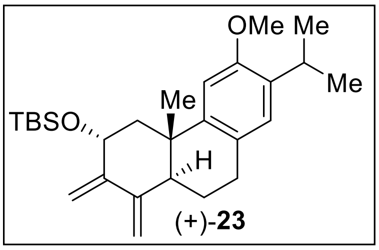

(+)-**23**

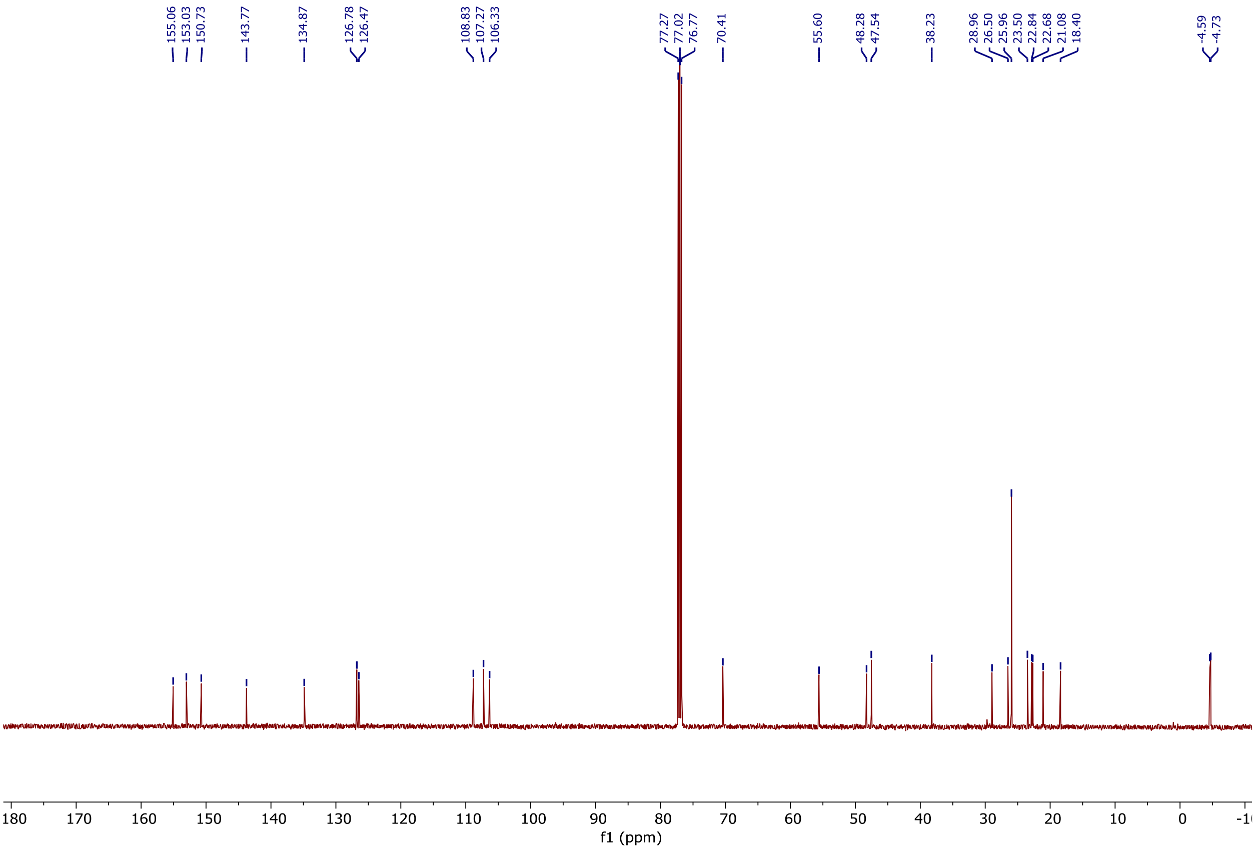

^{13}C NMR (126 MHz, $CDCl_3$) of compound (+)-**23**

Display Report

Analysis Info

Analysis Name	D:\Data\USER DATA 2022\JULY2022\15-07-22\Dr.A.Bisai -ABSK0281-R.d	Acquisition Date	15-07-2022 12:55:43
Method	tune mix_low.New.021117.m	Operator	Bruker
Sample Name	ABSK0281-R	Instrument	micrOTOF-Q 10330
Comment			

Acquisition Parameter

Source Type	ESI	Ion Polarity	Positive	Set Nebulizer	0.4 Bar
Focus	Not active	Set Capillary	4600 V	Set Dry Heater	180 °C
Scan Begin	50 m/z	Set End Plate Offset	-500 V	Set Dry Gas	4.0 l/min
Scan End	3000 m/z	Set Collision Cell RF	100.0 Vpp	Set Divert Valve	Source

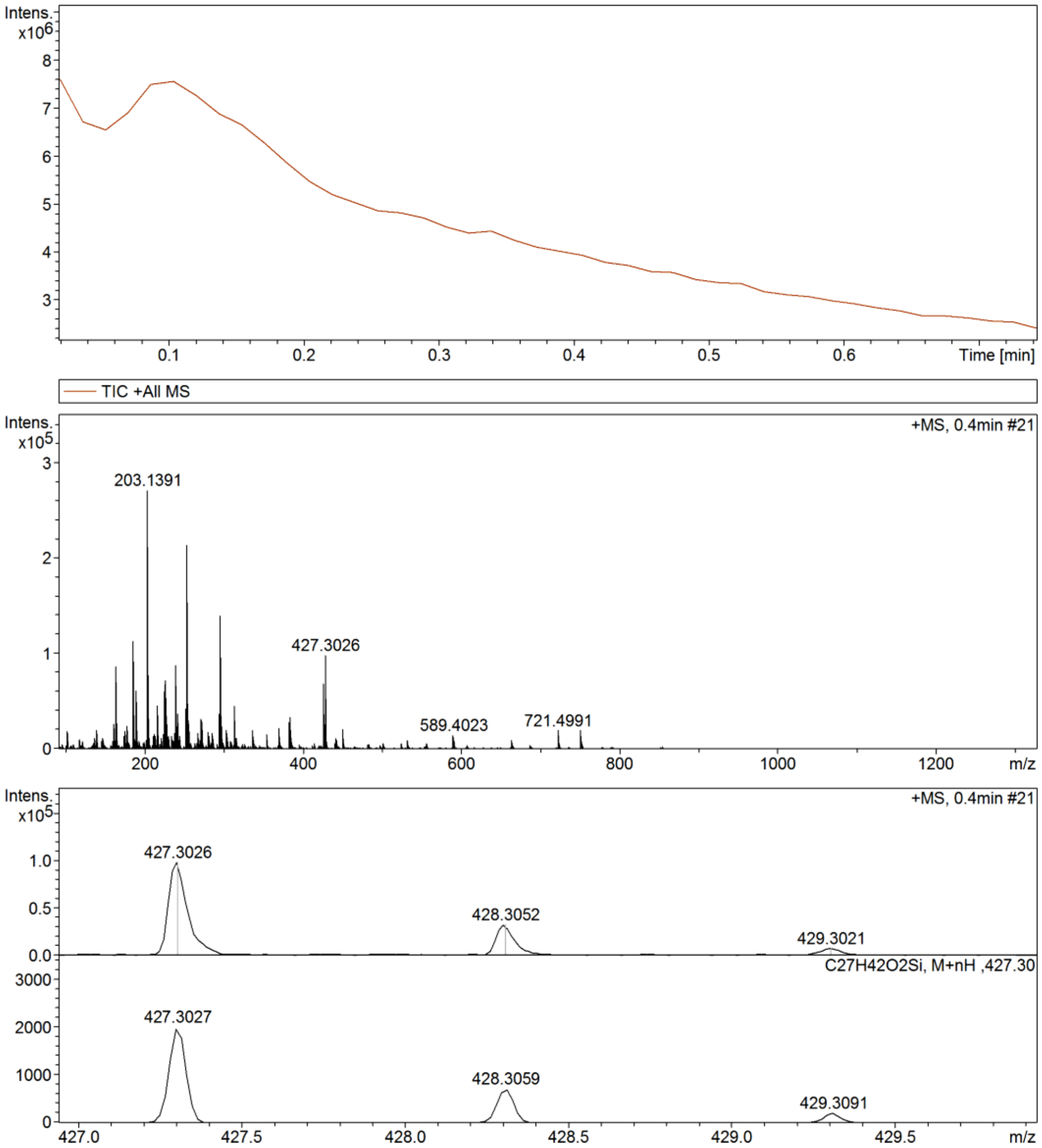

HRMS data of (+)-**23**

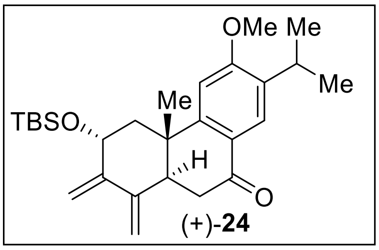

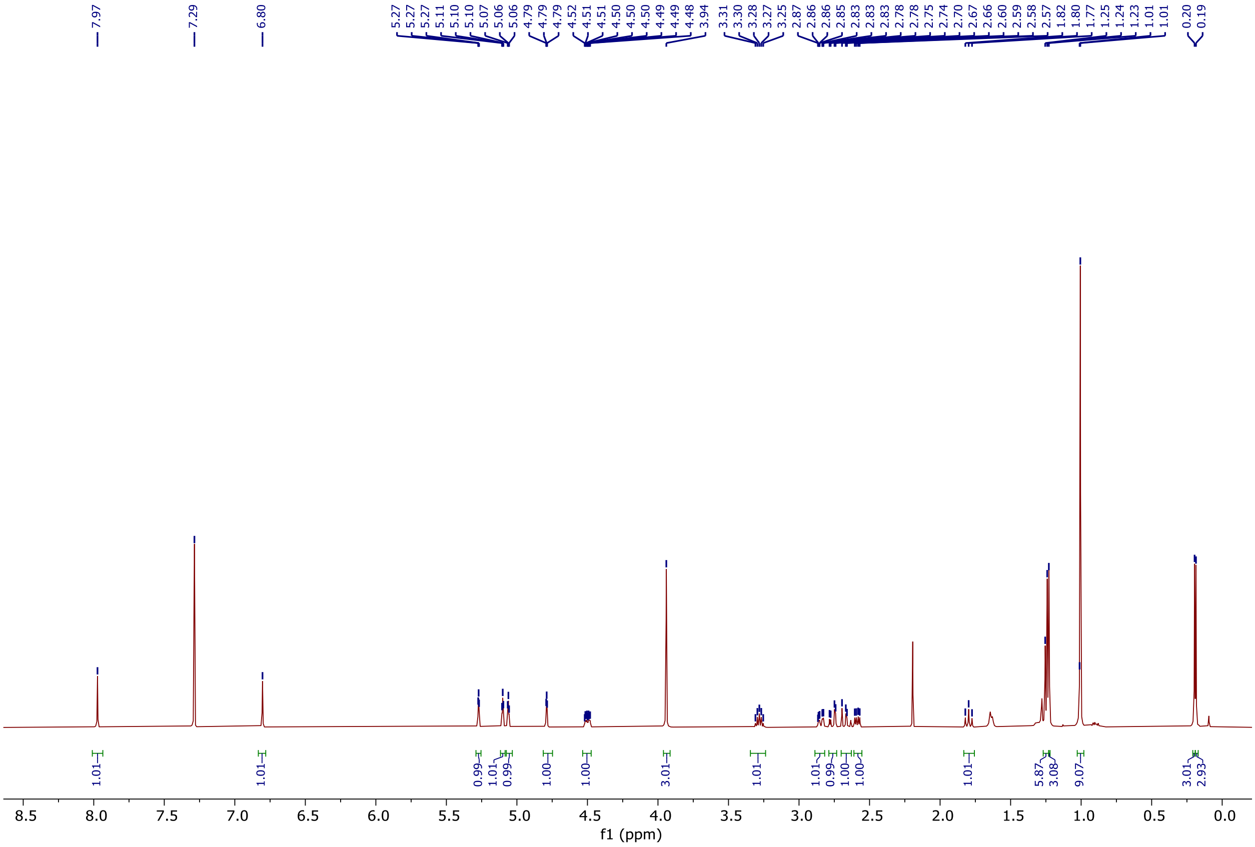

^{1}H NMR (500 MHz, $CDCl_3$) of compound (+)-**24**

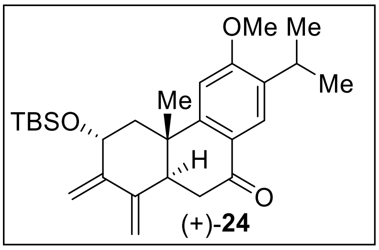

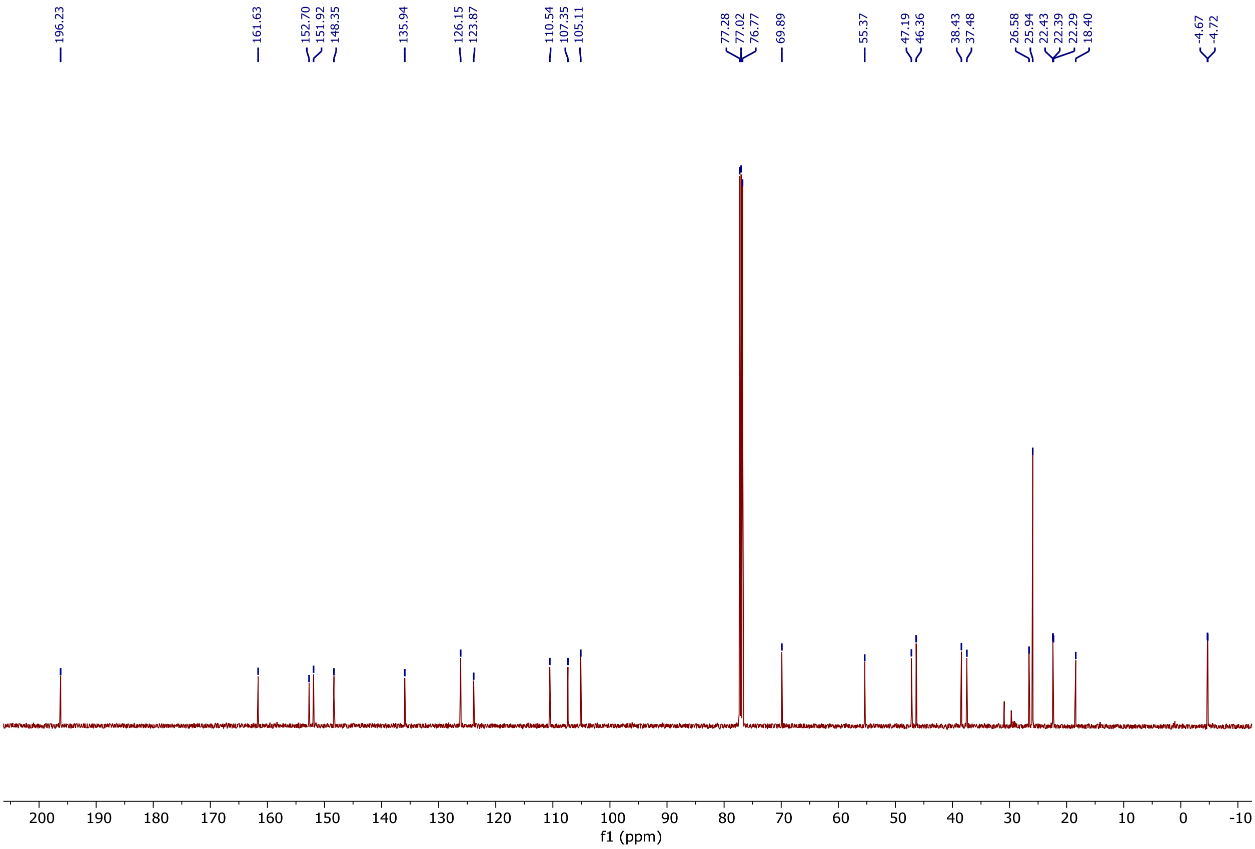

^{13}C NMR (126 MHz, $CDCl_3$) of compound (+)-**24**

Display Report

Analysis Info

Analysis Name	D:\Data\USER DATA 2022\JUNE2022\28-06-22\Prof A Bisai-SK-02-82.d	Acquisition Date	28-06-2022 11:55:30
Method	tune mix_low.New.021117_1.m	Operator	Bruker
Sample Name	SK-02-82	Instrument	micrOTOF-Q 10330
Comment			

Acquisition Parameter

Source Type	ESI	Ion Polarity	Positive	Set Nebulizer	0.4 Bar
Focus	Not active	Set Capillary	4600 V	Set Dry Heater	180 °C
Scan Begin	50 m/z	Set End Plate Offset	-500 V	Set Dry Gas	4.0 l/min
Scan End	3000 m/z	Set Collision Cell RF	100.0 Vpp	Set Divert Valve	Source

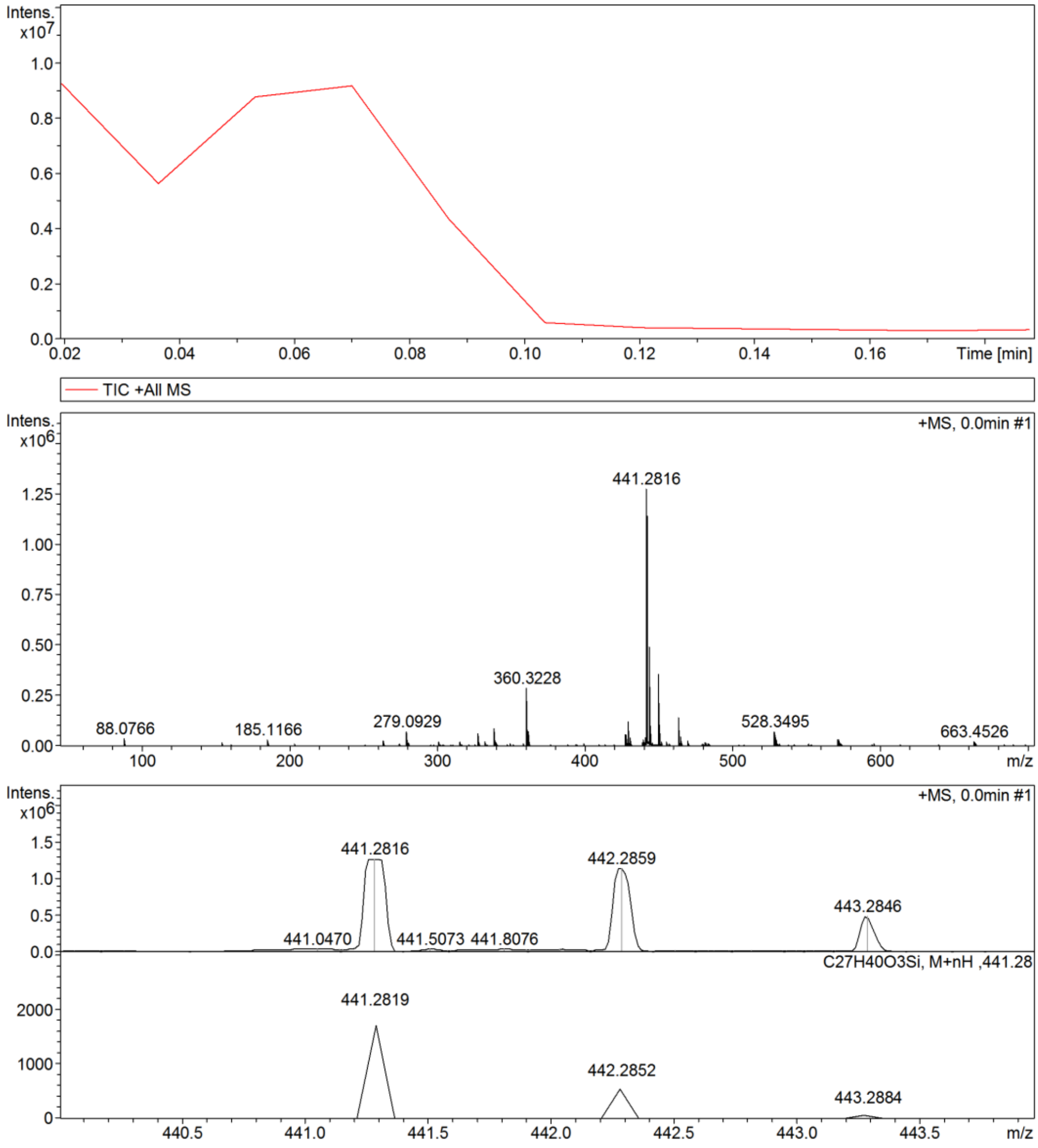

HRMS data of (+)-**24**

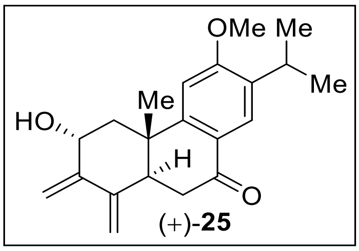

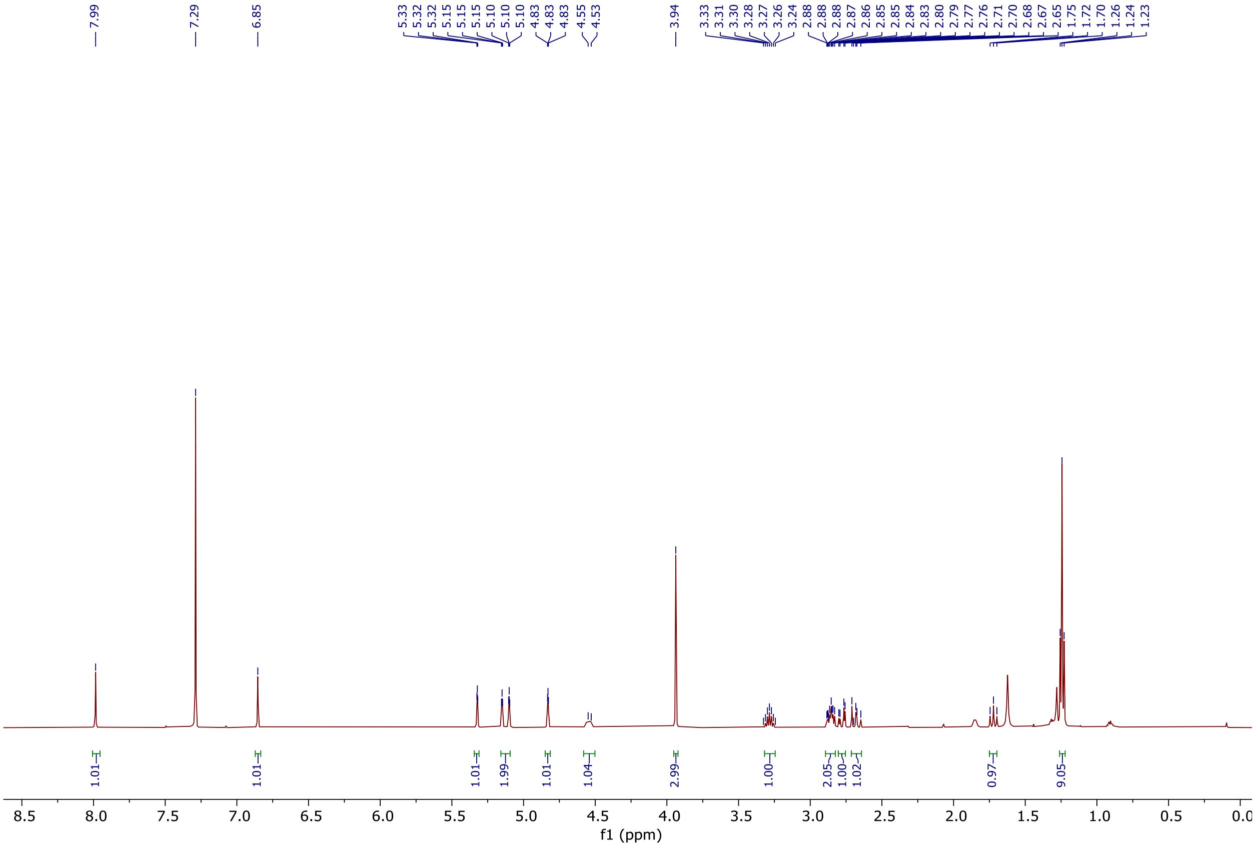

^{1}H NMR (500 MHz, $CDCl_3$) of compound (+)-**25**

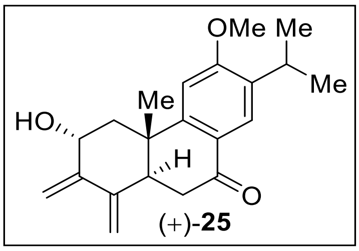

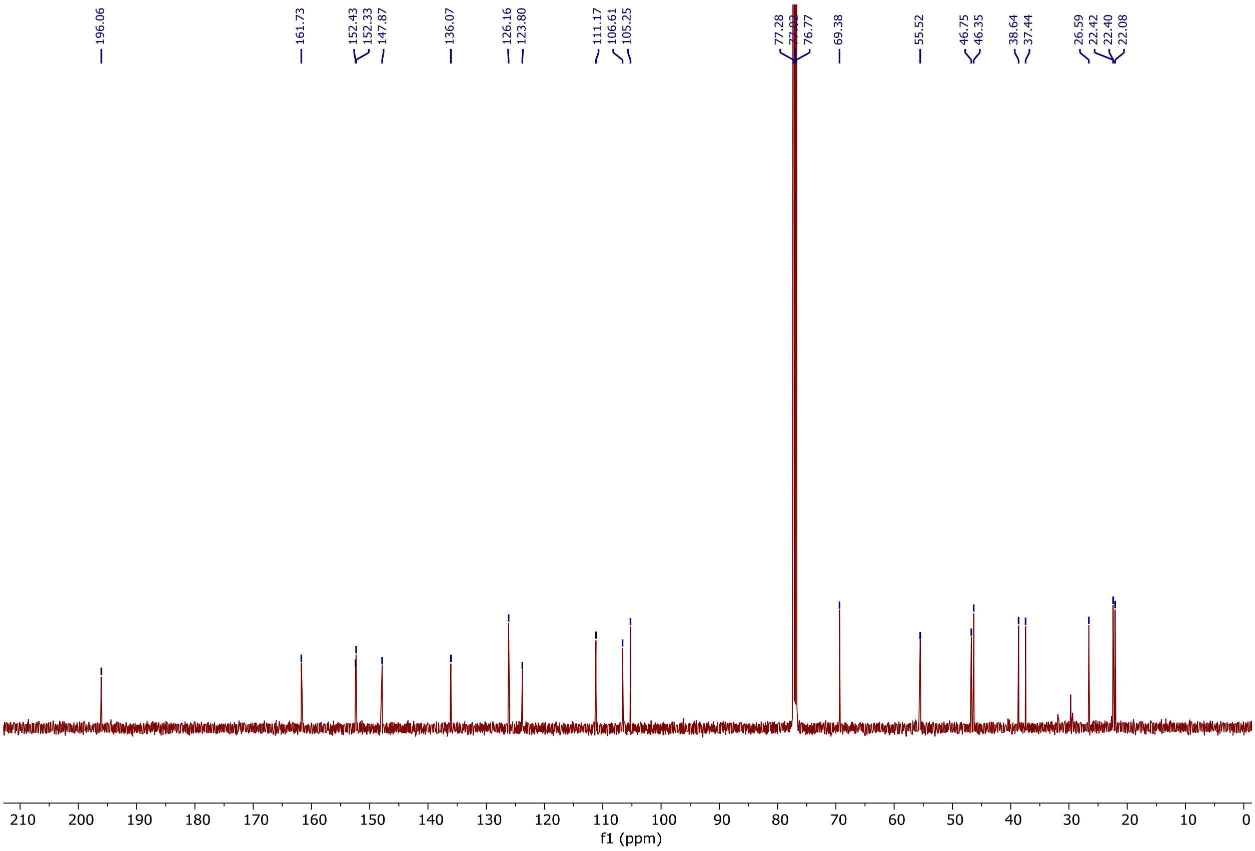

^{13}C NMR (126 MHz, $CDCl_3$) of compound (+)-**25**

Display Report

Analysis Info

Analysis Name	D:\Data\USER DATA 2022\JUNE2022\28-06-22\Prof A Bisai-SK-02-83.d	Acquisition Date	28-06-2022 11:51:44
Method	tune mix_low.New.021117_1.m	Operator	Bruker
Sample Name	SK-02-83	Instrument	micrOTOF-Q 10330
Comment			

Acquisition Parameter

Source Type	ESI	Ion Polarity	Positive	Set Nebulizer	0.4 Bar
Focus	Not active	Set Capillary	4600 V	Set Dry Heater	180 °C
Scan Begin	50 m/z	Set End Plate Offset	-500 V	Set Dry Gas	4.0 l/min
Scan End	3000 m/z	Set Collision Cell RF	100.0 Vpp	Set Divert Valve	Source

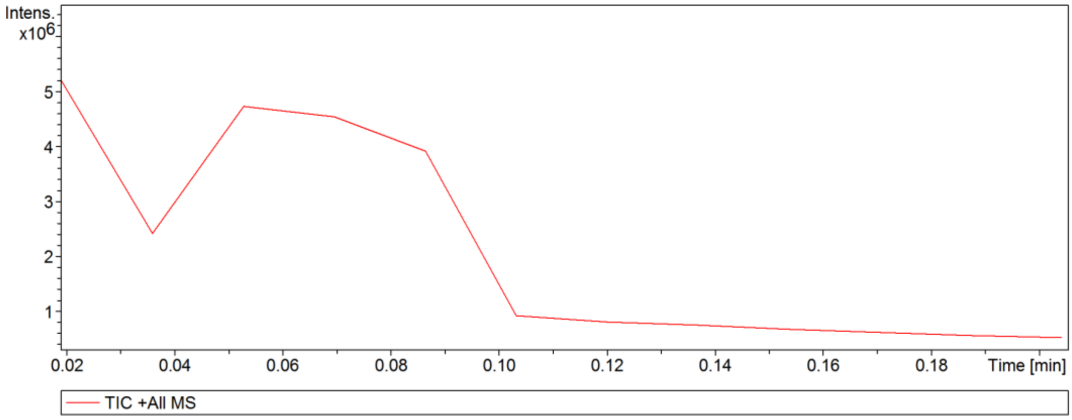

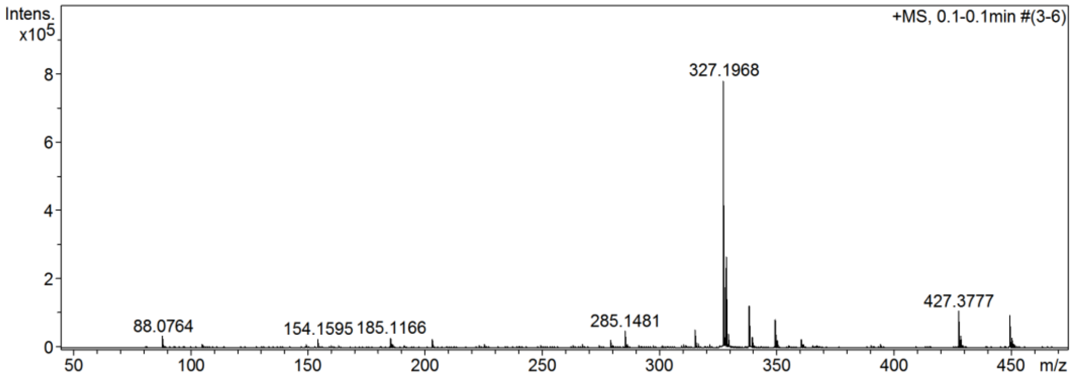

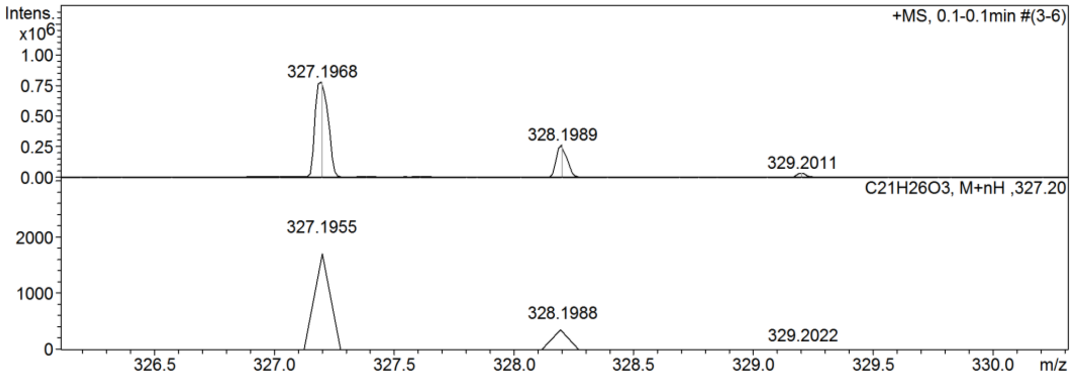

HRMS data of (+)-**25**

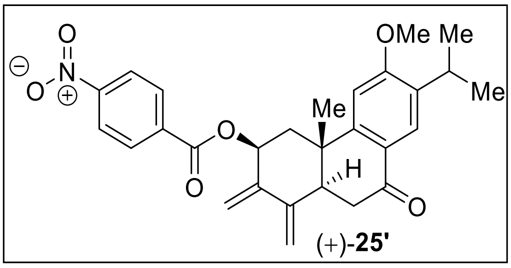

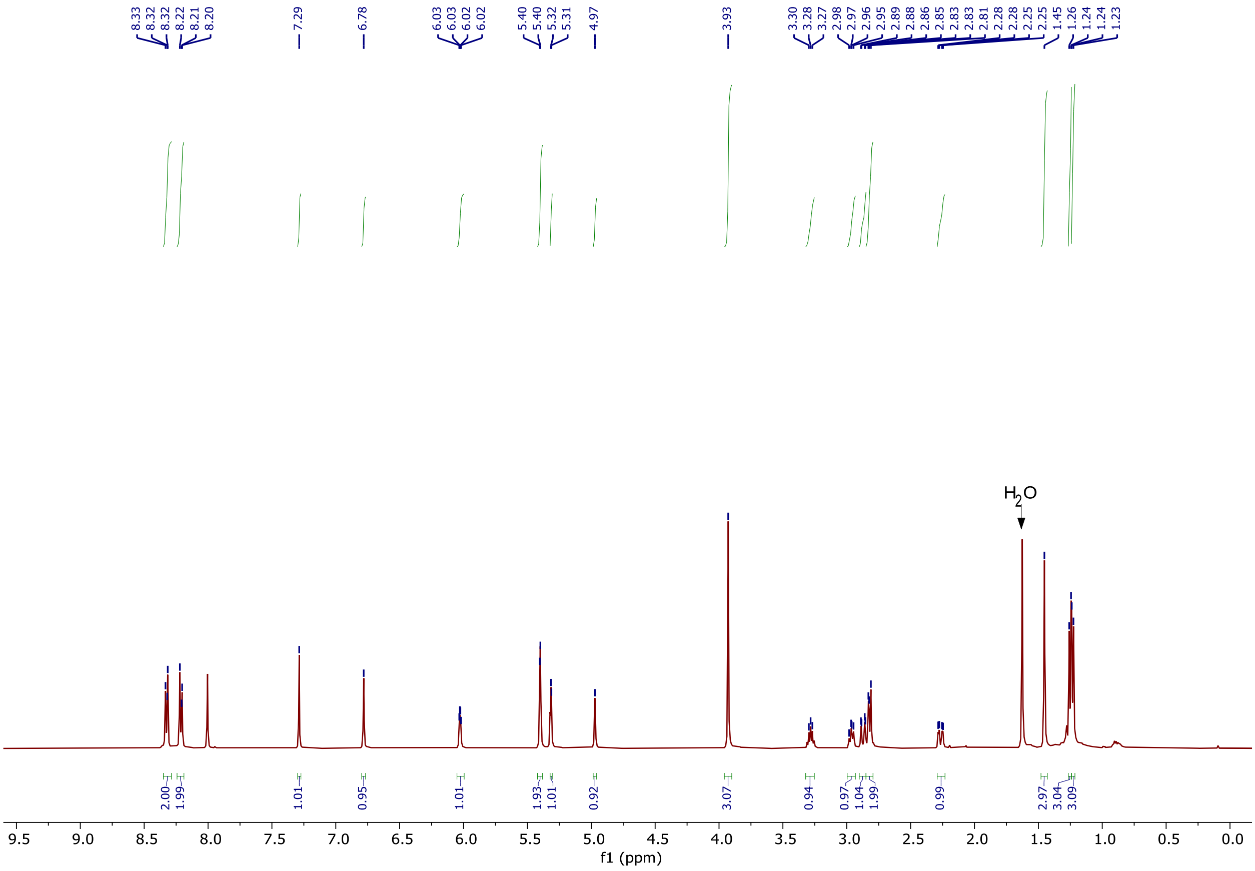

^{1}H NMR (500 MHz, $CDCl_3$) of compound (+)-**25’**

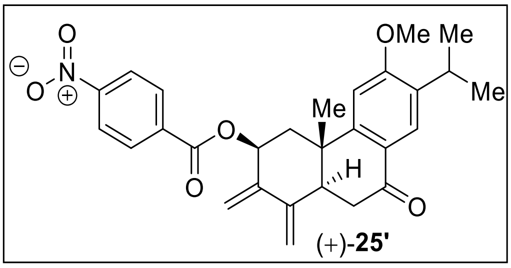

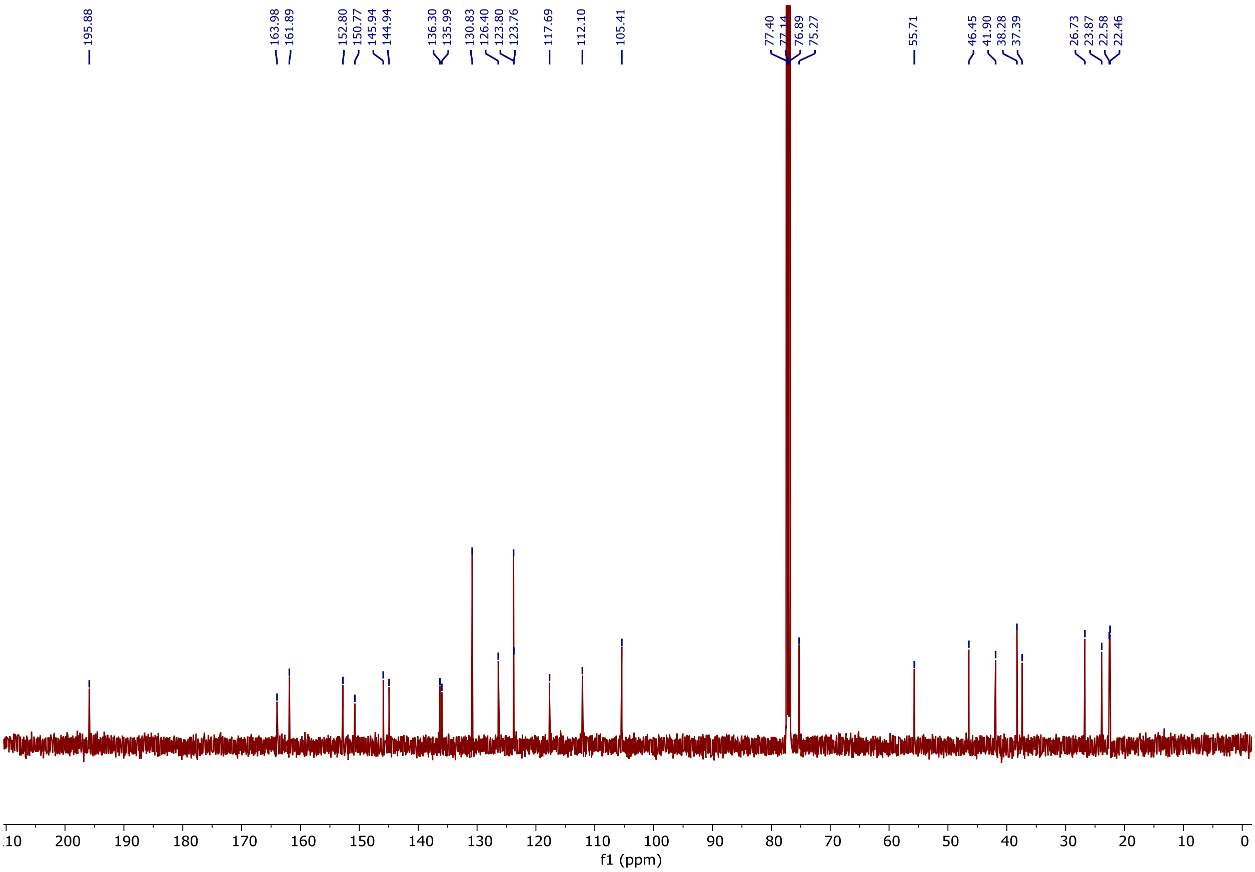

^{13}C NMR (126 MHz, $CDCl_3$) of compound (+)-**25'**

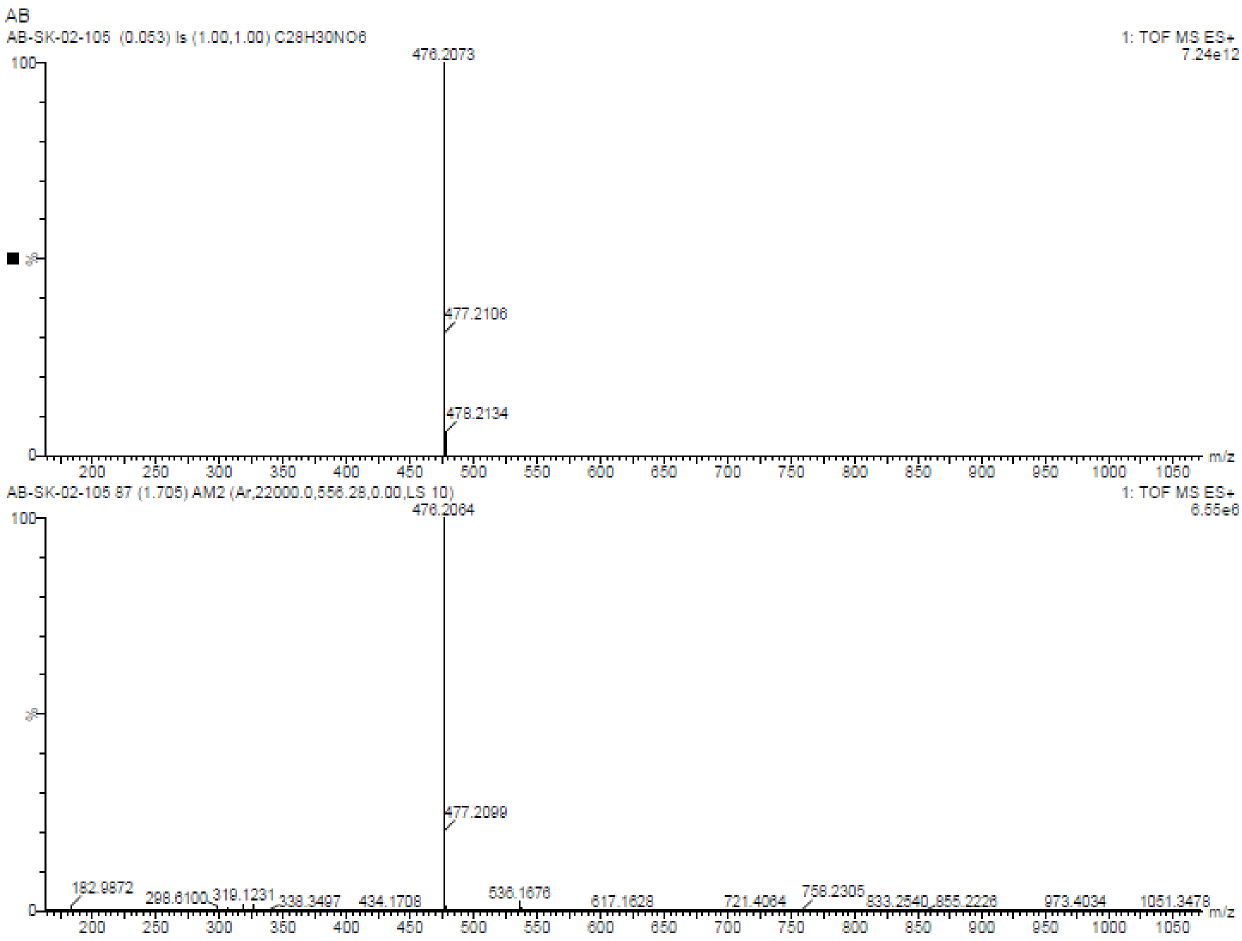

HRMS data of (+)-**25’**

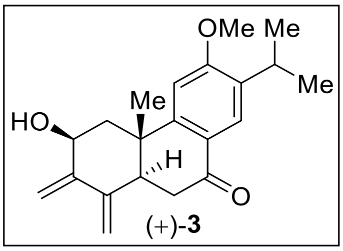

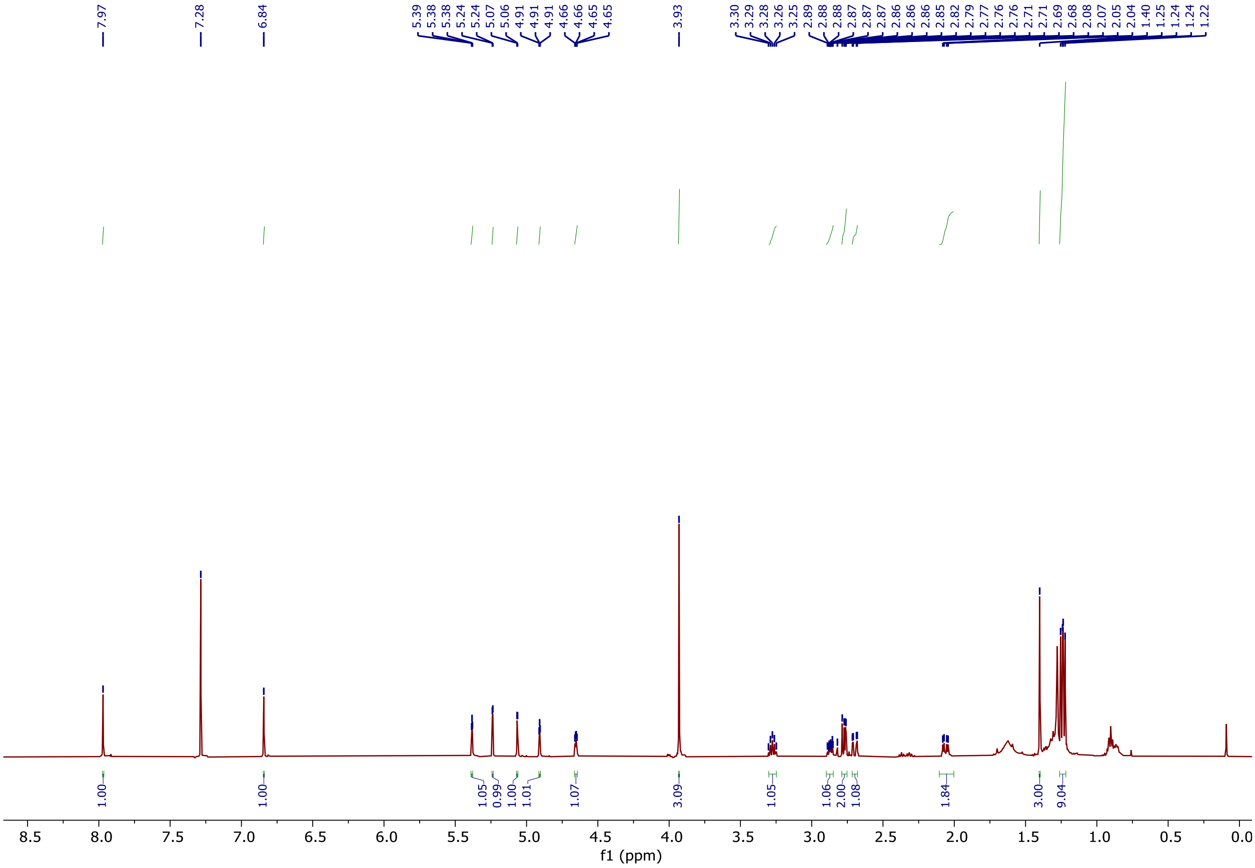

^{1}H NMR (500 MHz, $CDCl_3$) of compound (+)-**3**

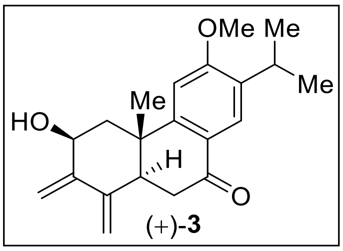

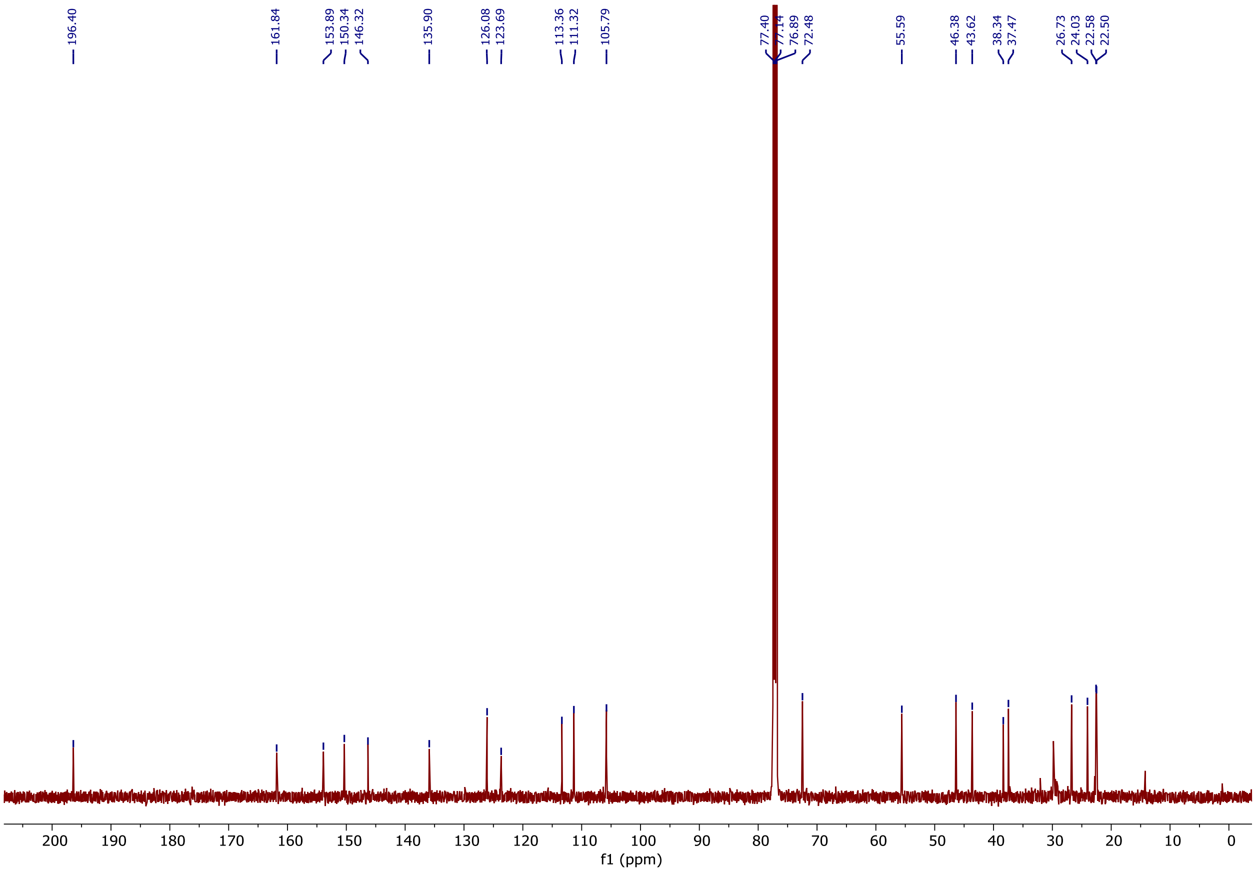

^{13}C NMR (126 MHz, $CDCl_3$) of compound (+)-**3**

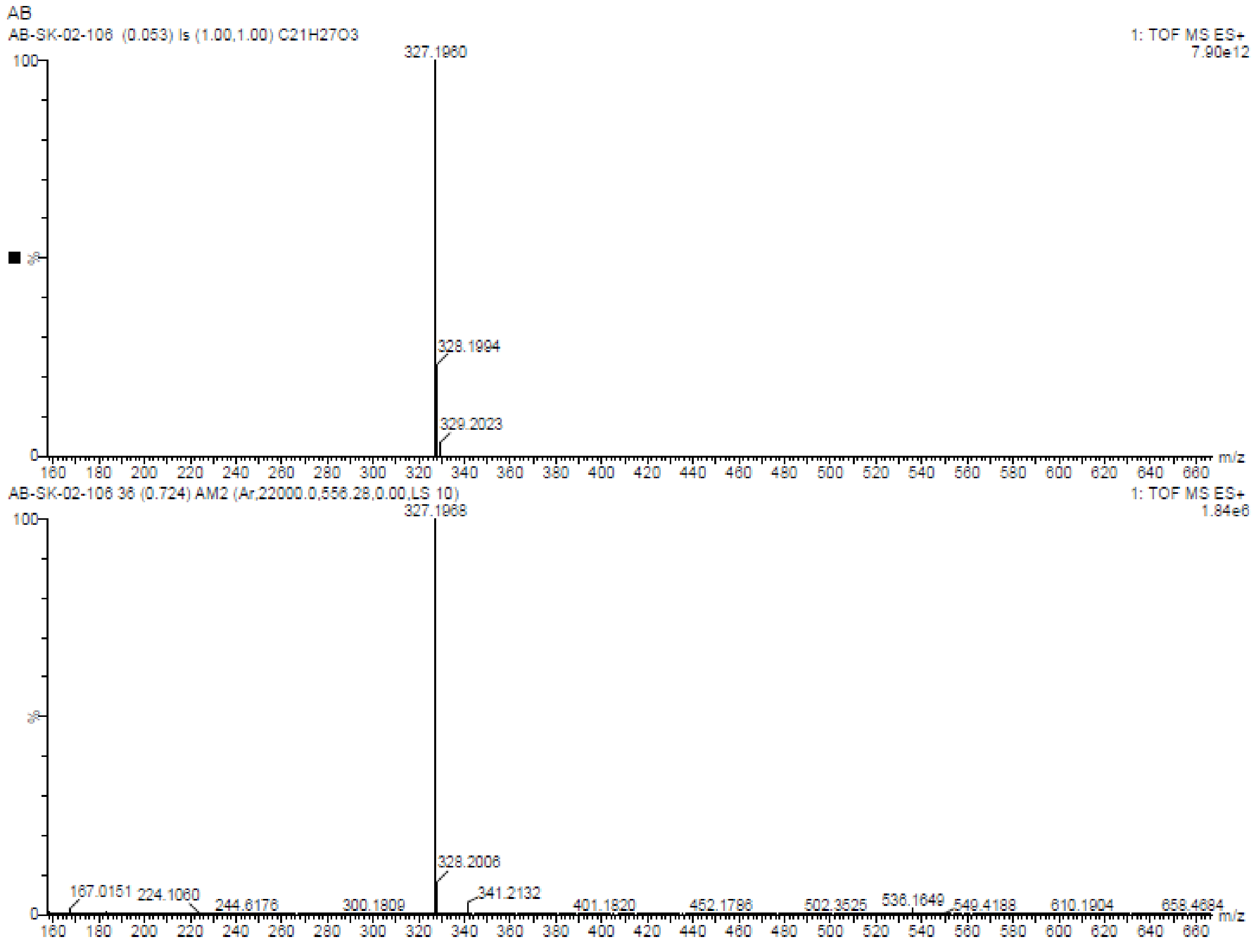

HRMS data of (+)-**3**

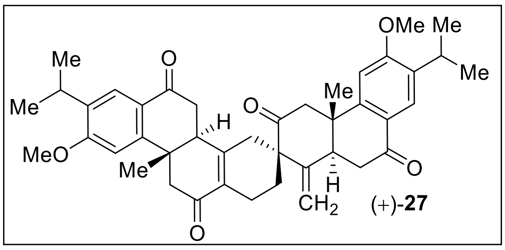

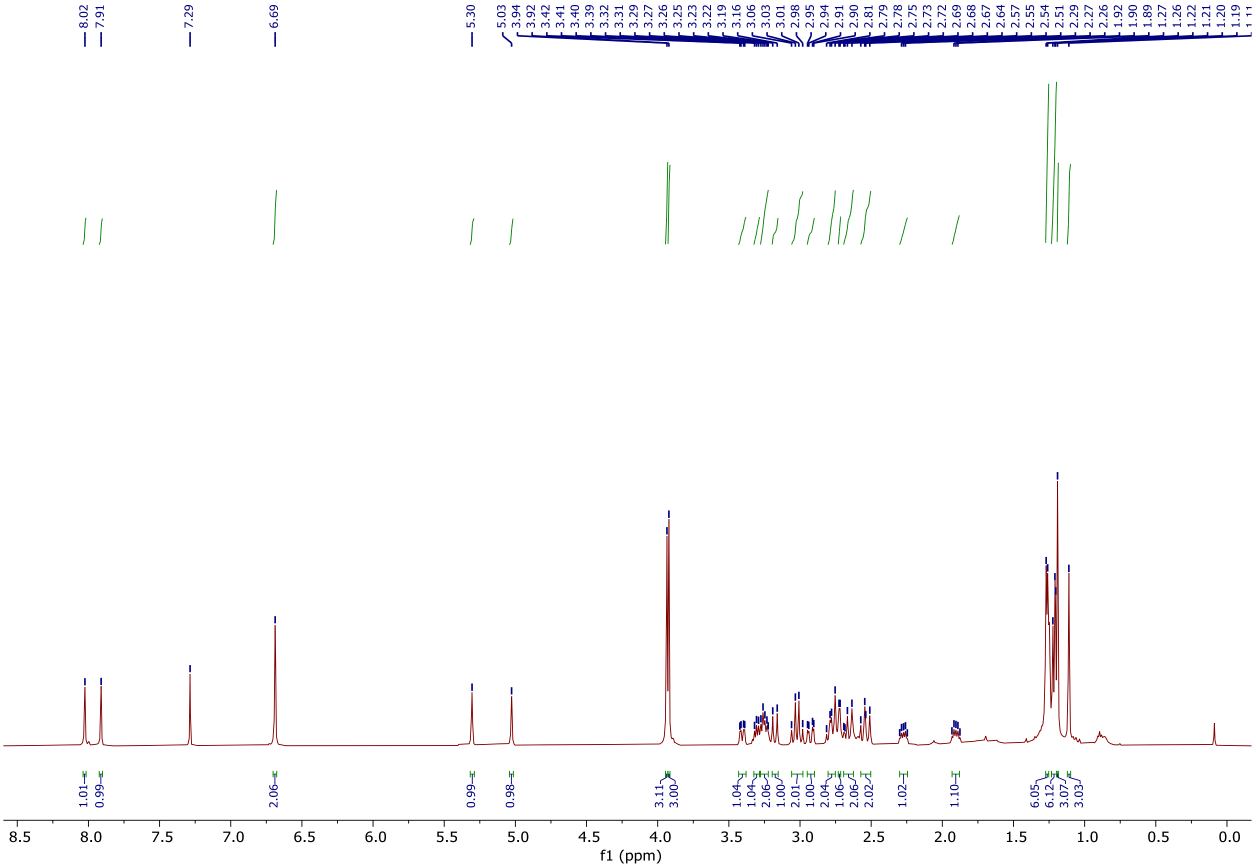

^{1}H NMR (500 MHz, $CDCl_3$) of compound (+)-**27**

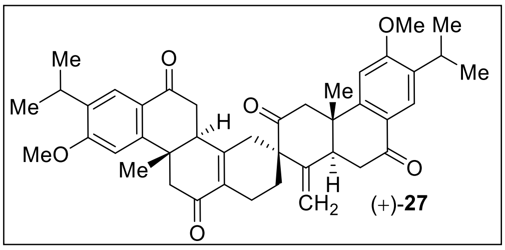

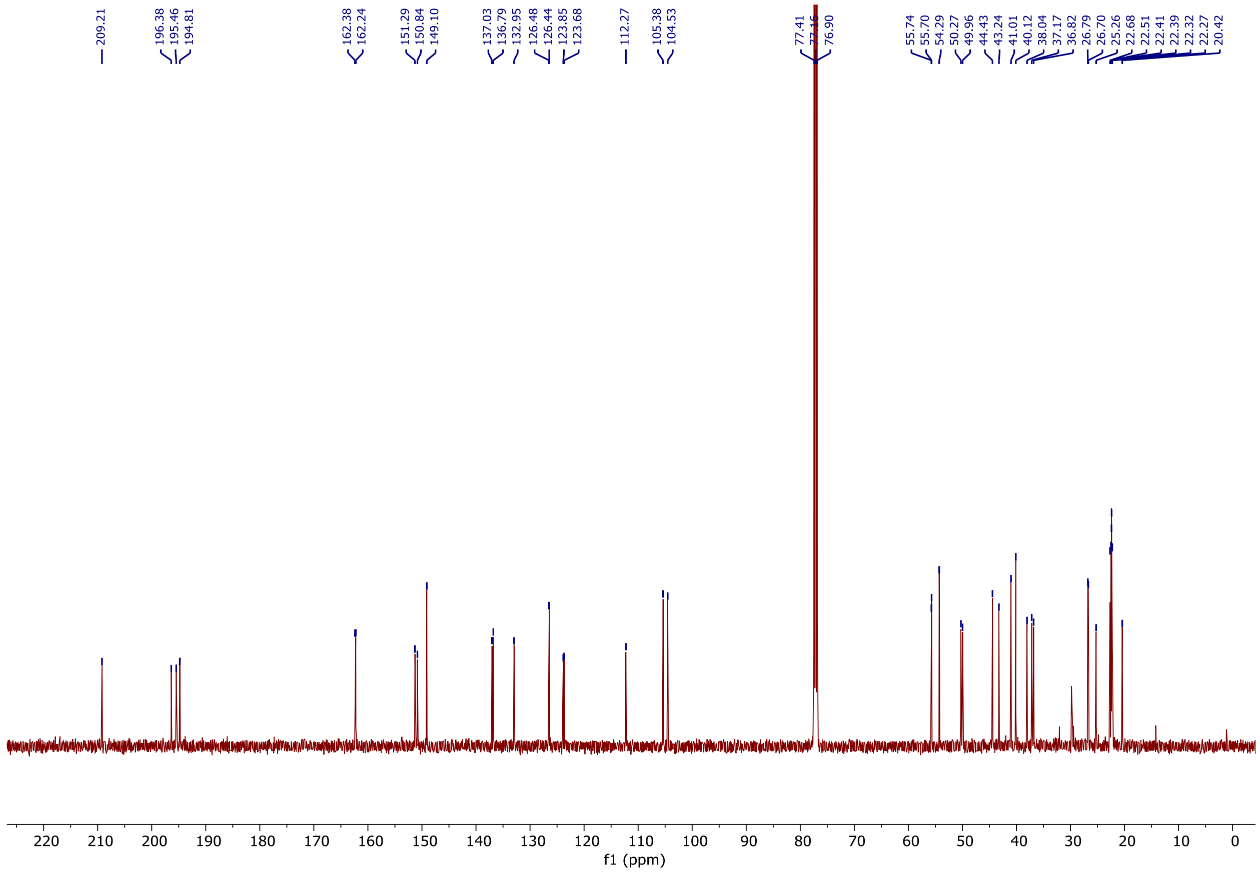

^{13}C NMR (126 MHz, $CDCl_3$) of compound (+)-**27**

Display Report

Analysis Info

Analysis Name	D:\Data\USER DATA 2022\JULY2022\05-07-22\Dr.A.Bisai-ABSK0284SP.d	Acquisition Date	05-07-2022 16:11:23
Method	tune mix_low.New.021117.m	Operator	Bruker
Sample Name	ABSK0284SP	Instrument	micrOTOF-Q 10330
Comment			

Acquisition Parameter

Source Type	ESI	Ion Polarity	Positive	Set Nebulizer	0.4 Bar
Focus	Not active	Set Capillary	4600 V	Set Dry Heater	180 °C
Scan Begin	50 m/z	Set End Plate Offset	-500 V	Set Dry Gas	4.0 l/min
Scan End	3000 m/z	Set Collision Cell RF	100.0 Vpp	Set Divert Valve	Source

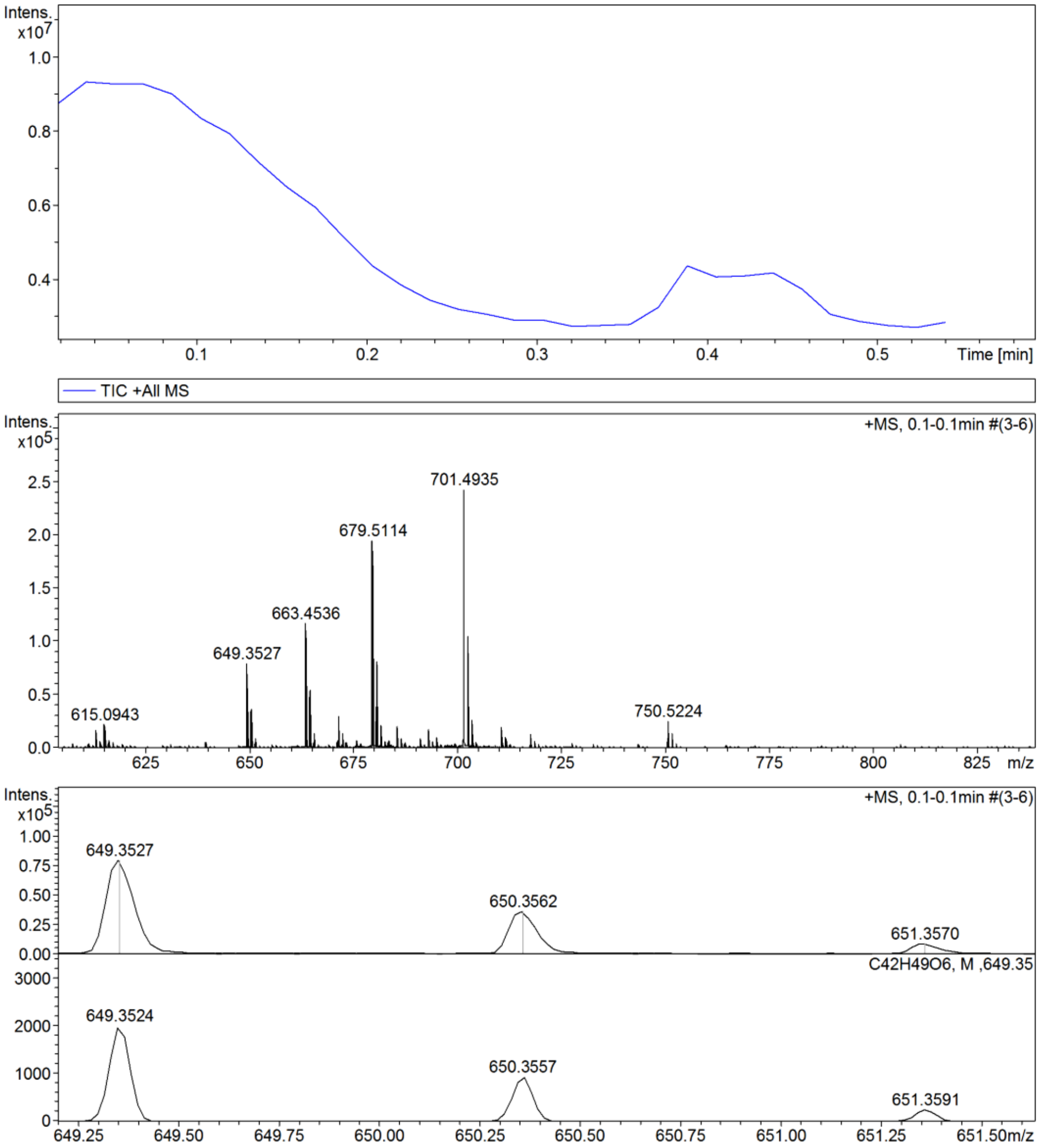

HRMS data of (+)-**27**

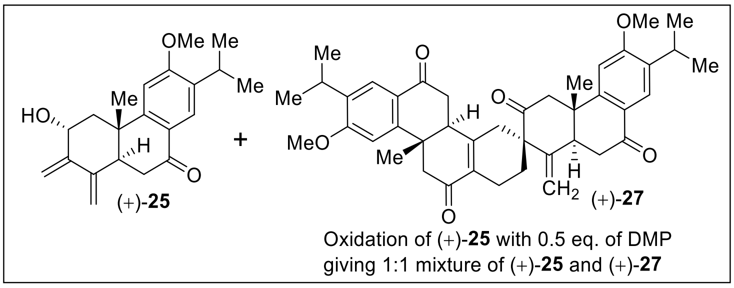

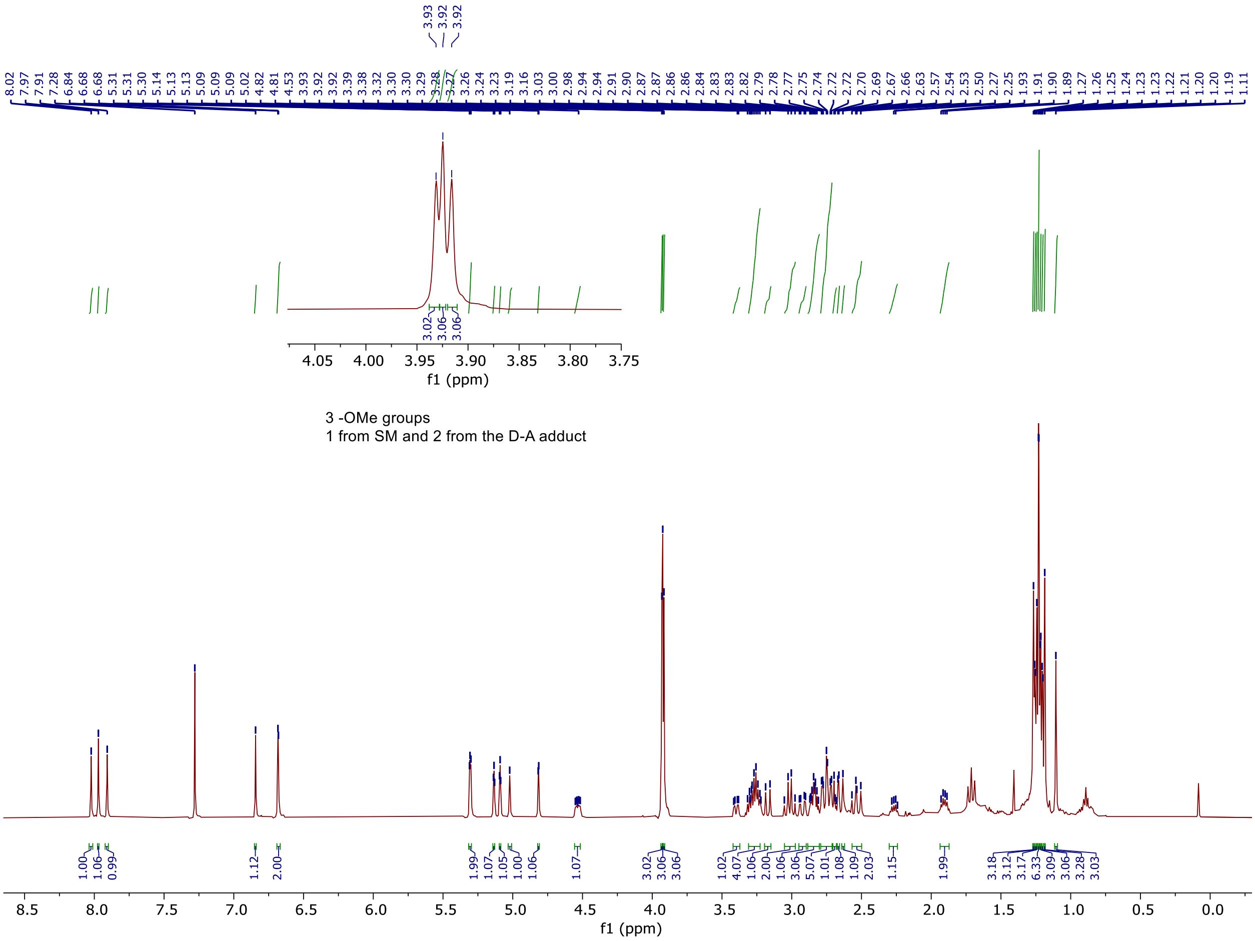

^{1}H NMR (500 MHz, $CDCl_3$) of 1:1 mixture of (+)-**25** and (+)-**27**

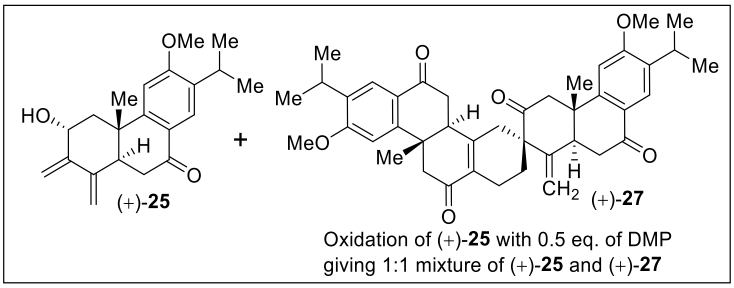

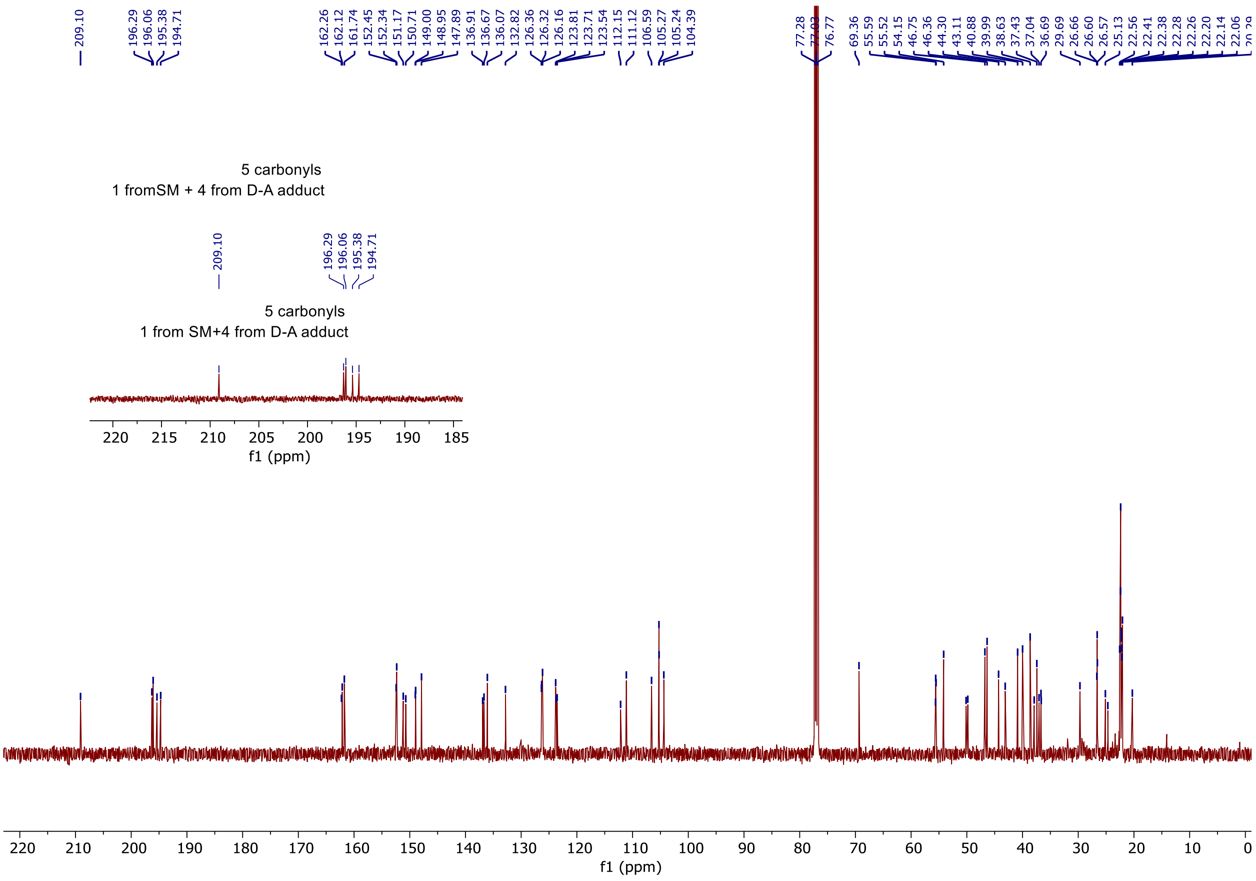

^{13}C NMR (126 MHz, $CDCl_3$) of 1:1 mixture of (+)-**25** and (+)-**27**

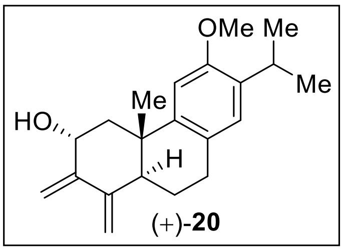

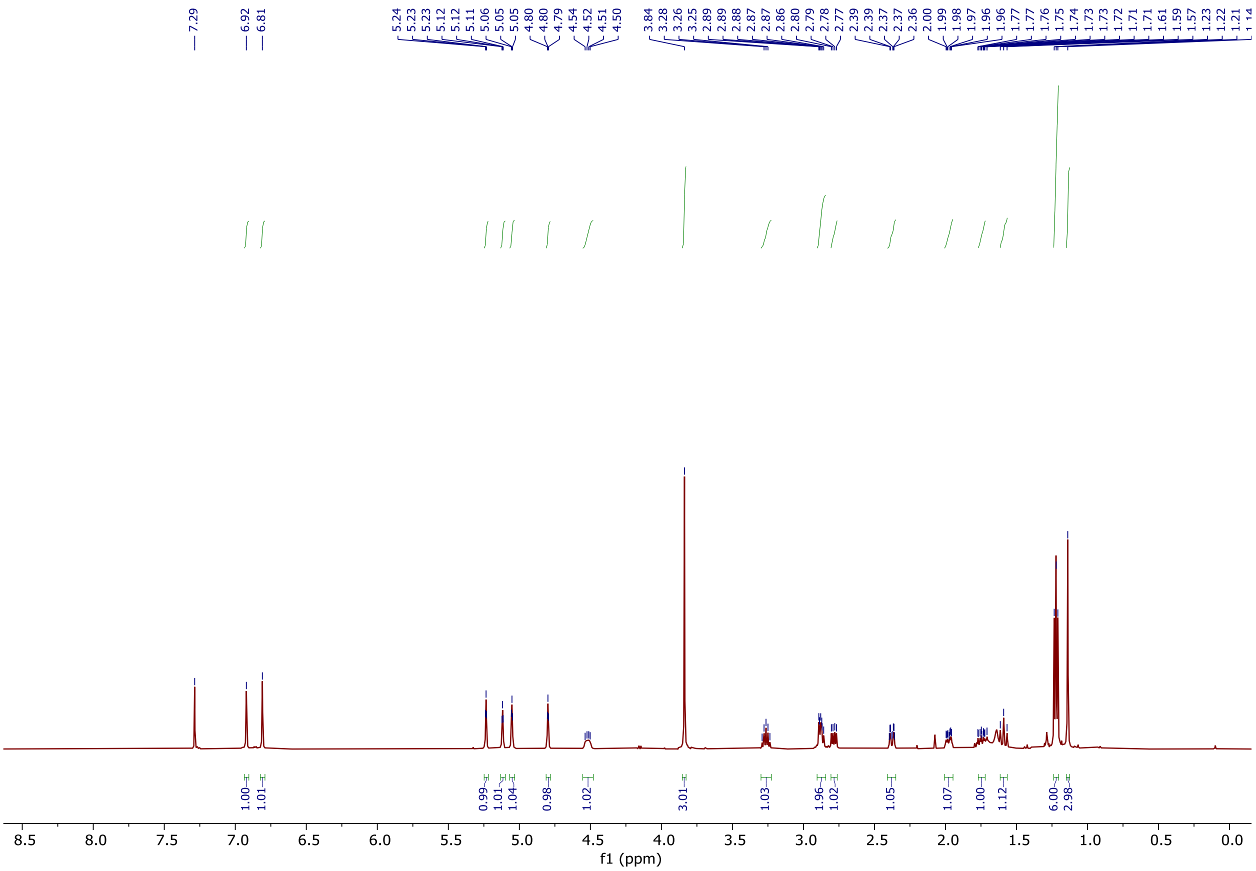

^{1}H NMR (500 MHz, $CDCl_3$) of compound (+)-**20**

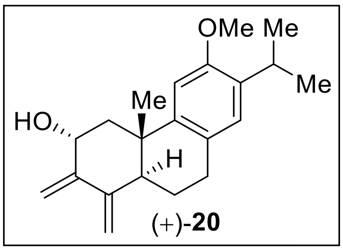

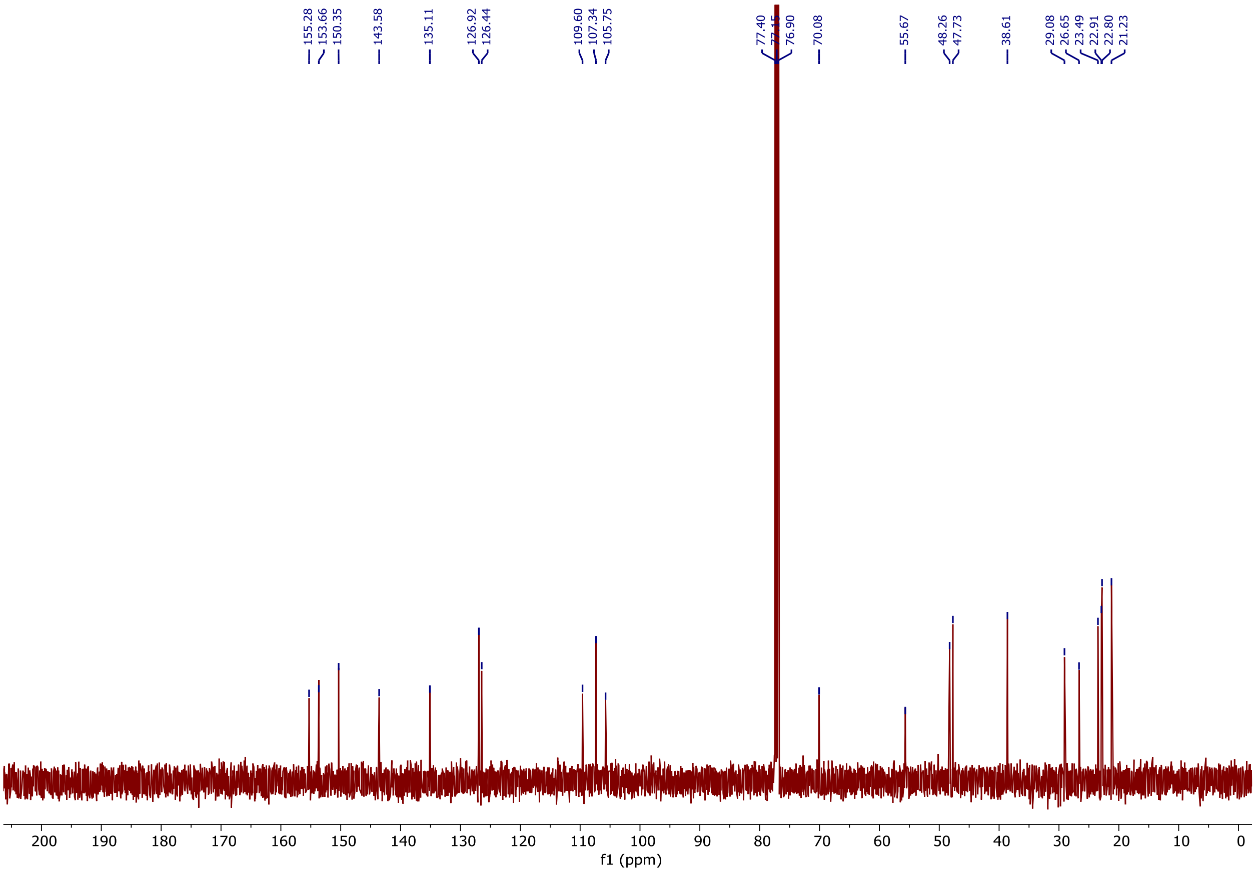

^{13}C NMR (126 MHz, $CDCl_3$) of compound (+)-**20**

Display Report

Analysis Info			
Analysis Name	D:\Data\User data\2022\OCT\ab_sk_02_100R.d	Acquisition Date	10/10/2022 11:29:49 AM
Method	Tune_pos_Standard_July2022.m	Operator	IISER Kolkata
Sample Name	ab_sk_02_100R	Instrument	maXis impact 8282001.00127
Comment			

Acquisition Parameter

Source Type	ESI	Ion Polarity	Positive	Set Nebulizer	0.4 Bar
Focus	Active	Set Capillary	4500 V	Set Dry Heater	200 °C
Scan Begin	50 m/z	Set End Plate Offset	-500 V	Set Dry Gas	4.0 l/min
Scan End	1000 m/z	Set Charging Voltage	2000 V	Set Divert Valve	Source
		Set Corona	0 nA	Set APCI Heater	0 °C

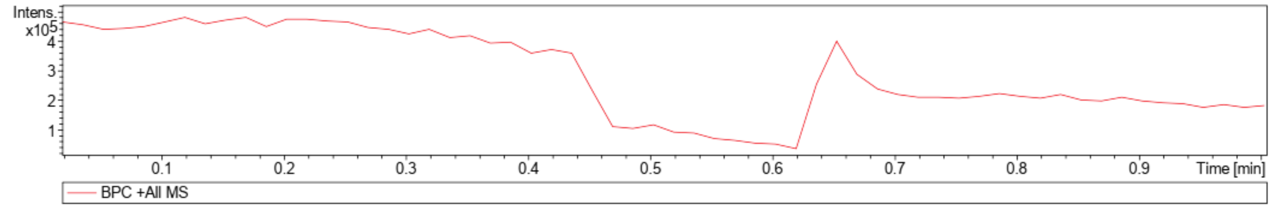

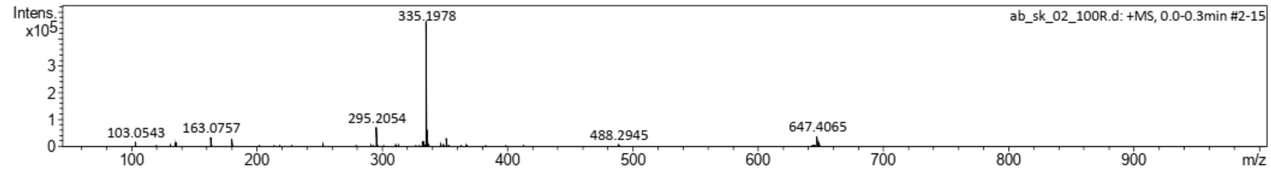

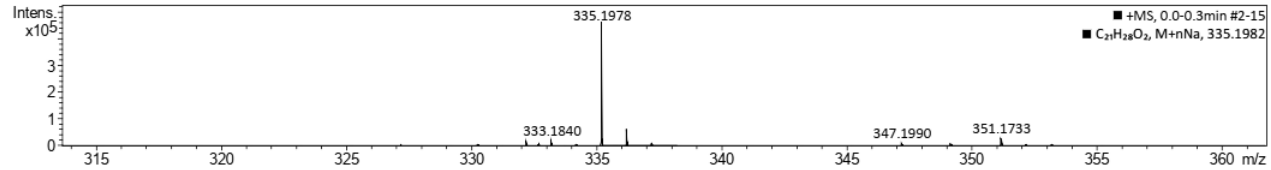

ab_sk_02_100R.d

HRMS data of (+)-**20**

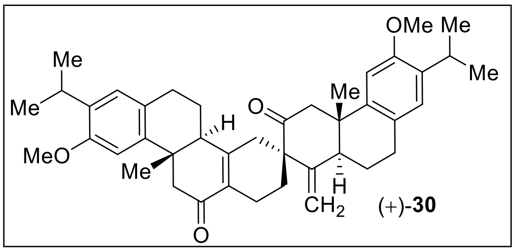

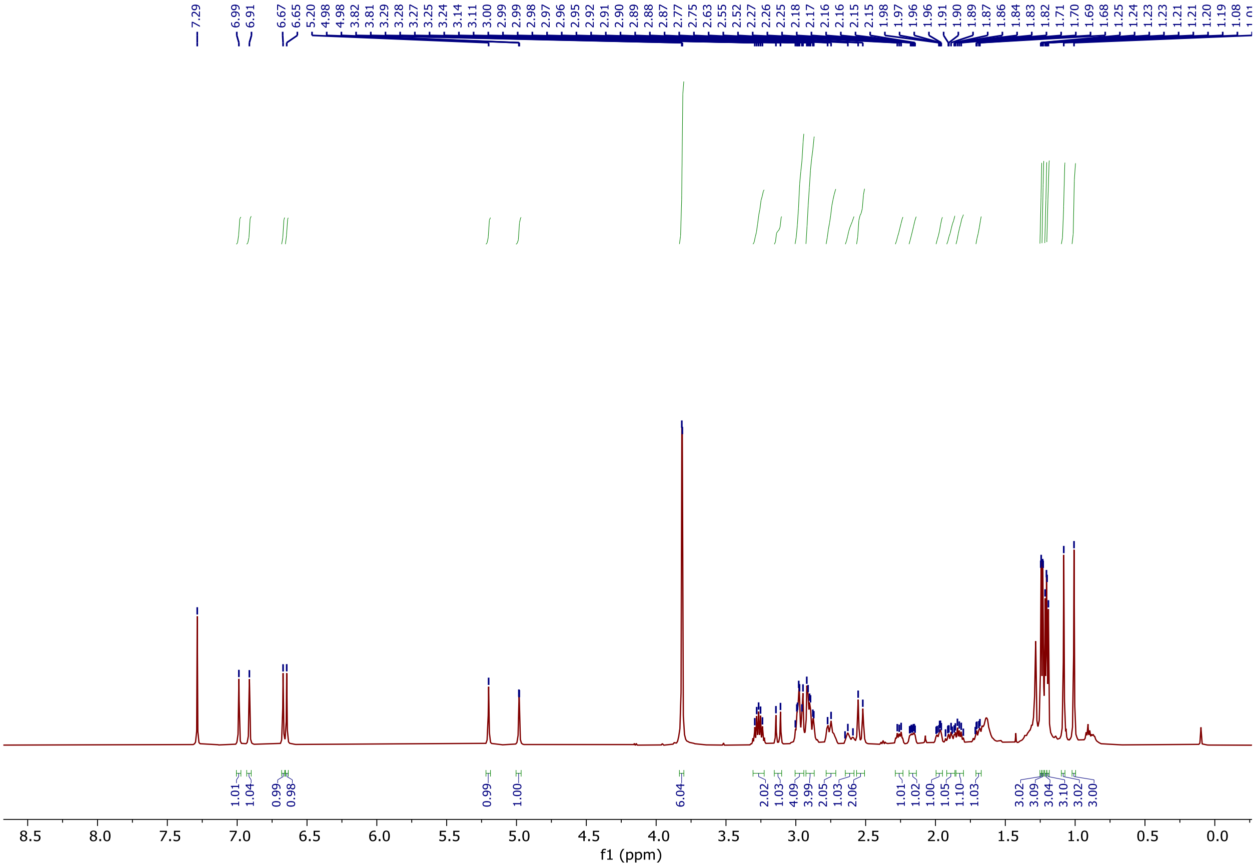

^{1}H NMR (500 MHz, $CDCl_3$) of compound (+)-**30**

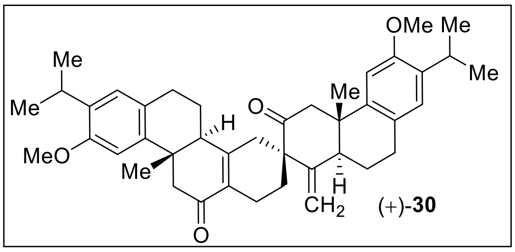

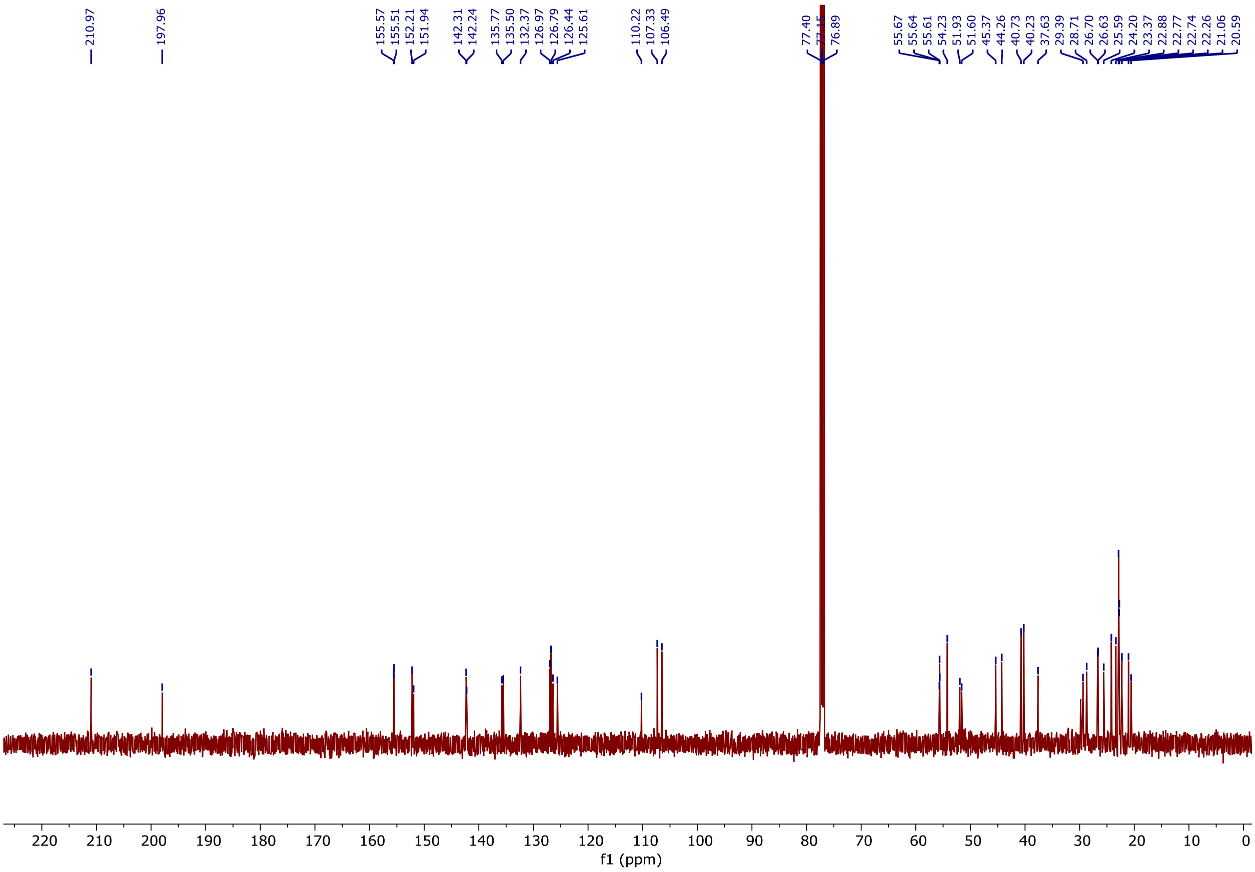

^{13}C NMR (126 MHz, $CDCl_3$) of compound (+)-**30**

Display Report

Analysis Info

Analysis Name	D:\Data\User data\2022\SEPT\ab_sk_02_101.d	Acquisition Date	10/10/2022 10:58:45 AM
Method	Tune_pos_Standard_July2022.m	Operator	IISER Kolkata
Sample Name	ab_sk_02_101	Instrument	maXis impact 8282001.00127
Comment			

Acquisition Parameter

Source Type	ESI	Ion Polarity	Positive	Set Nebulizer	0.4 Bar
Focus	Active	Set Capillary	4500 V	Set Dry Heater	200 °C
Scan Begin	50 m/z	Set End Plate Offset	-500 V	Set Dry Gas	4.0 l/min
Scan End	1000 m/z	Set Charging Voltage	2000 V	Set Divert Valve	Source
		Set Corona	0 nA	Set APCI Heater	0 °C

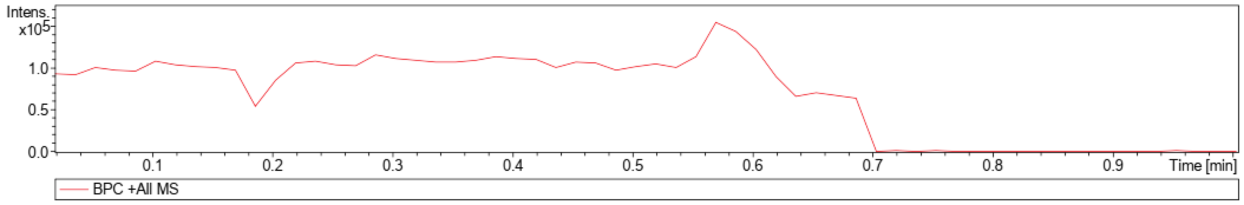

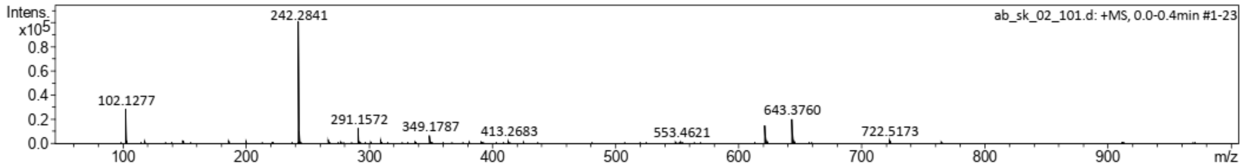

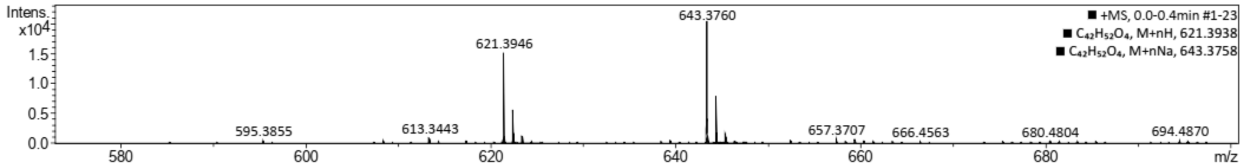

HRMS data of (+)-**30**

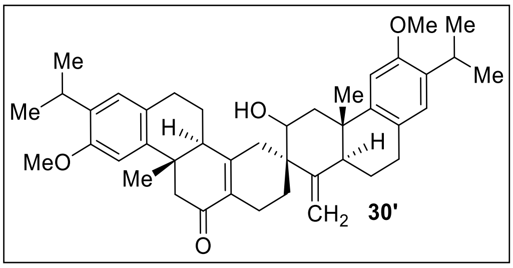

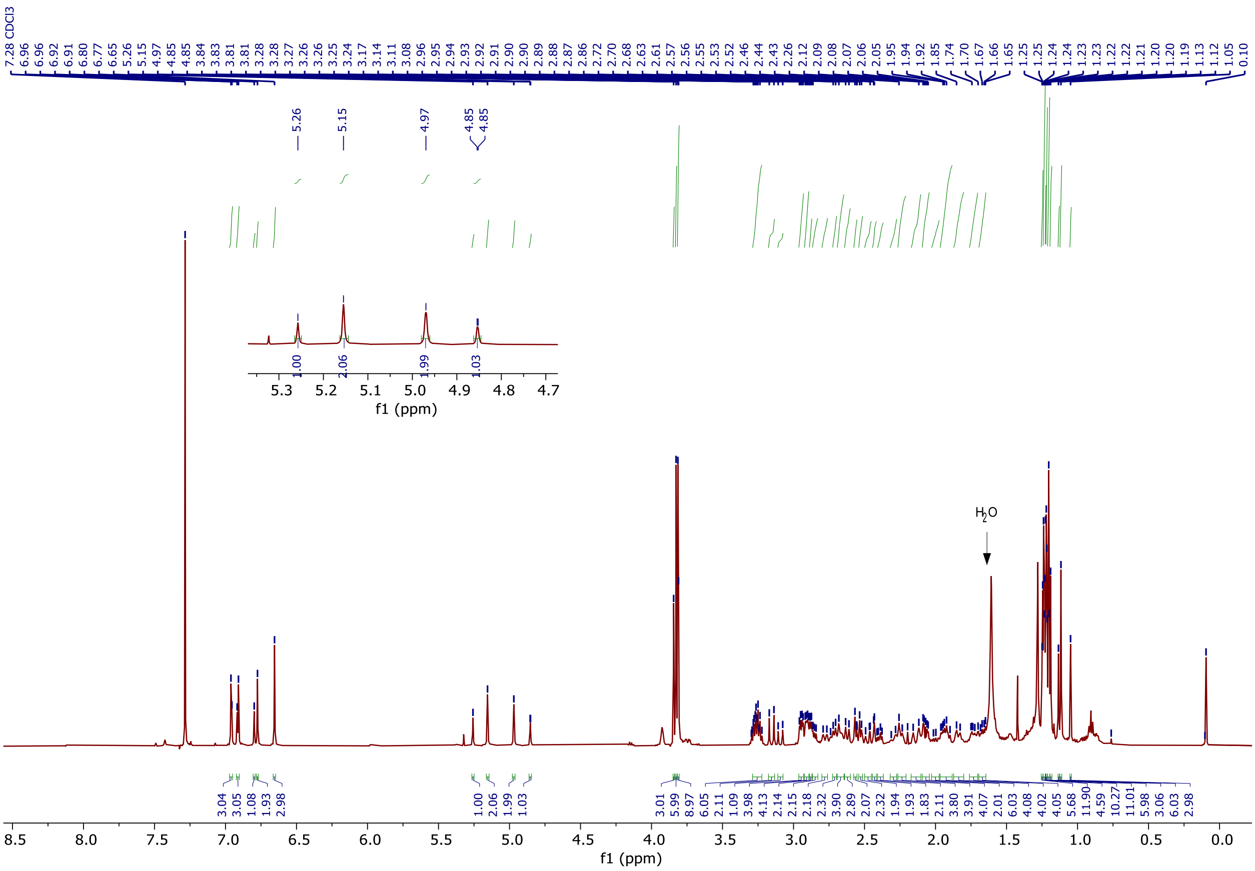

^{1}H NMR (500 MHz, $CDCl_3$) inseparable (2:1) diasteromeric mixture of **30’**

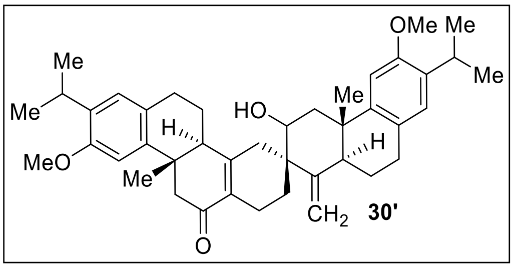

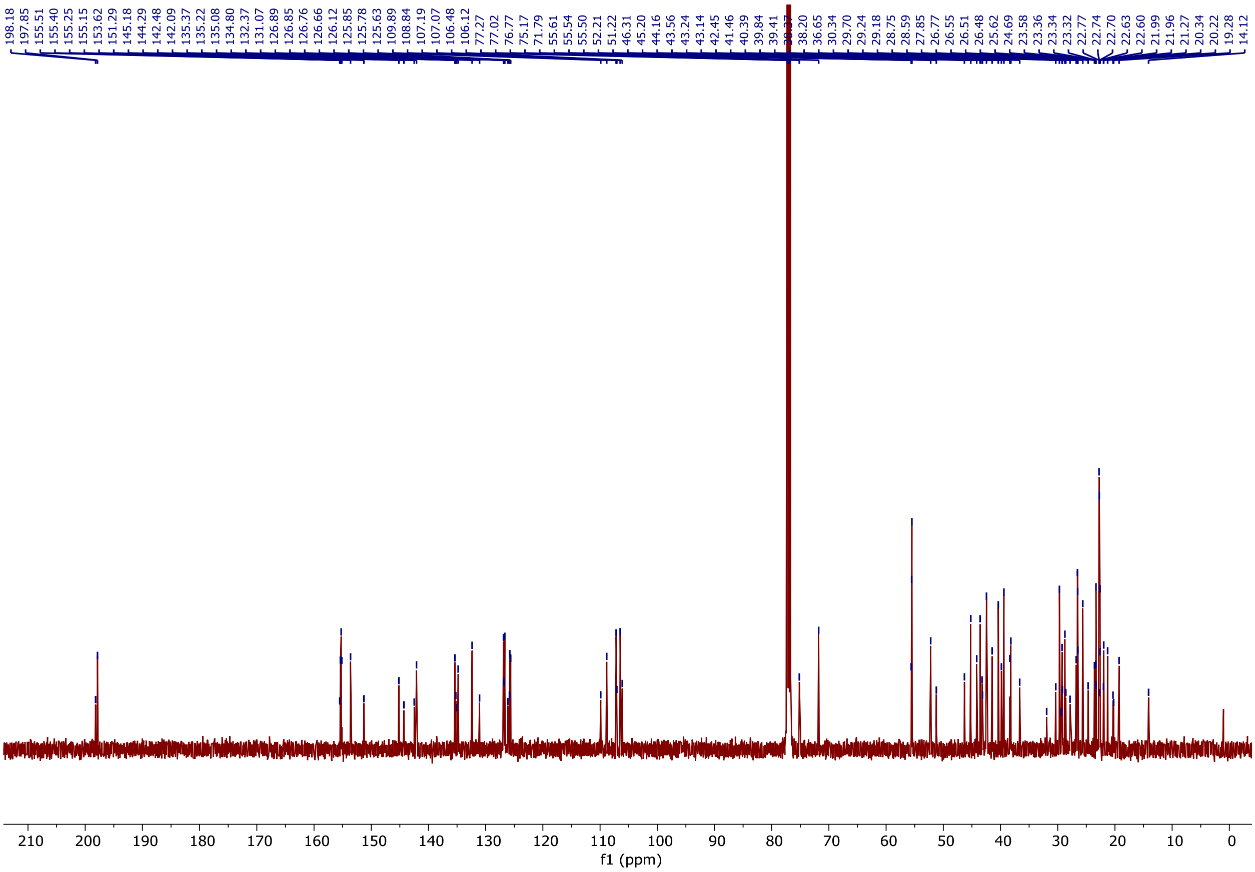

^{13}C NMR (126 MHz, $CDCl_3$) inseparable (2:1) diasteromeric mixture of **30’**

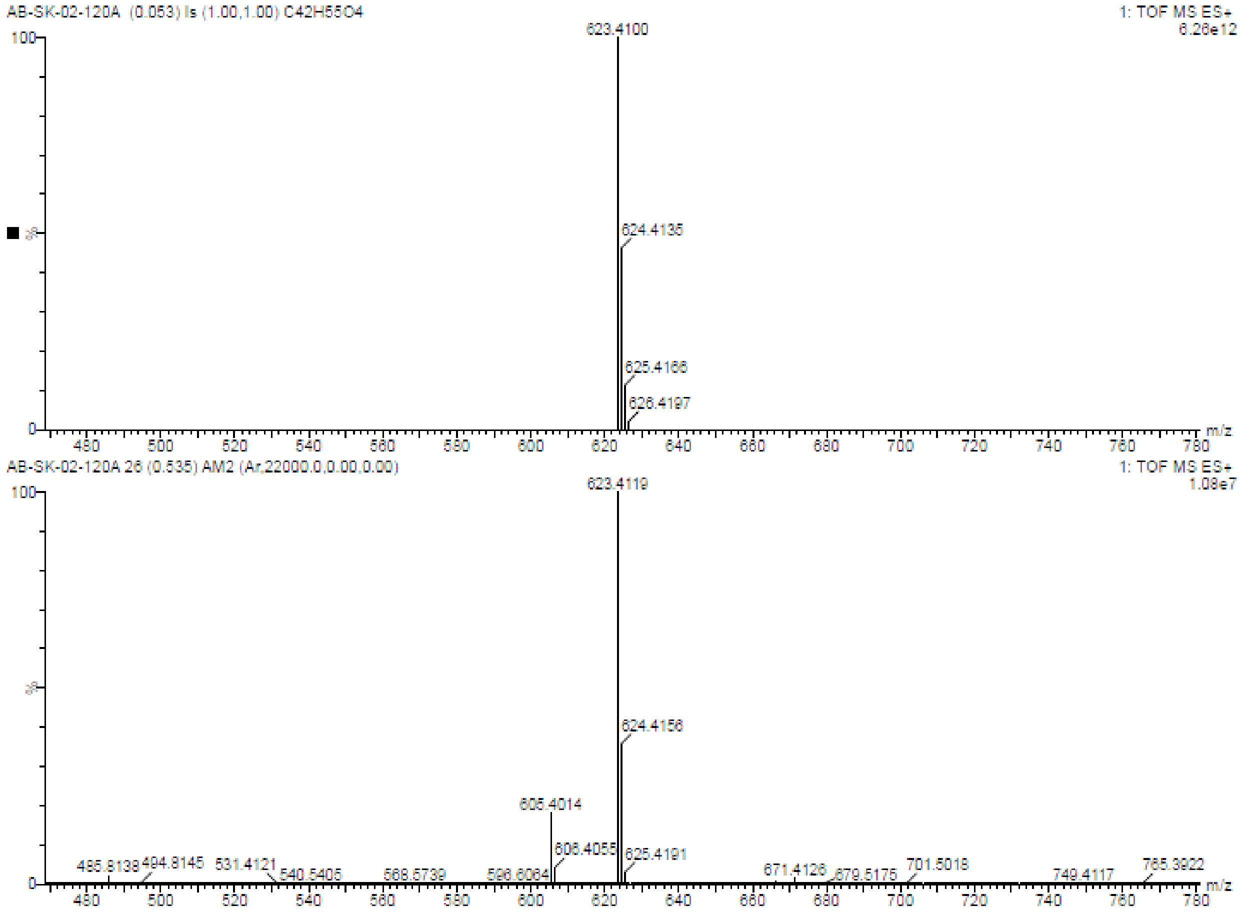

HRMS data of **30’**

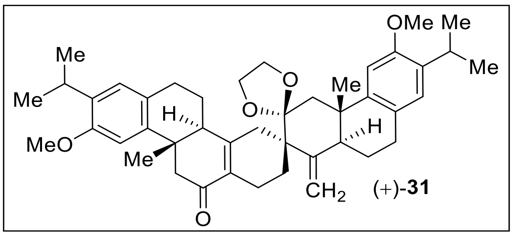

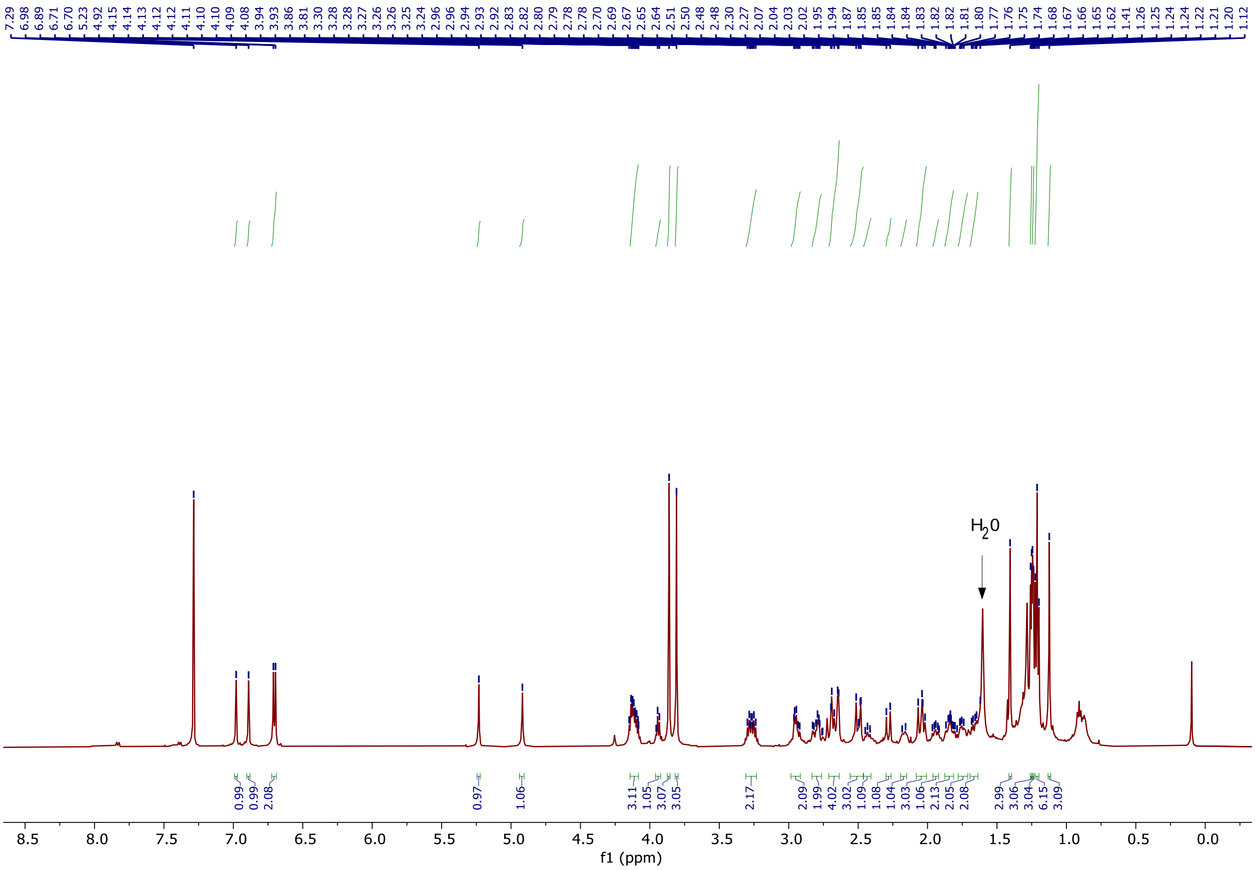

^1H NMR (500 MHz, $CDCl_3$) of compound (+)-**31**

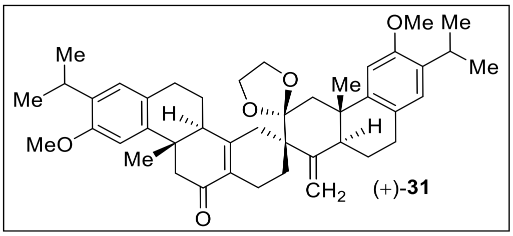

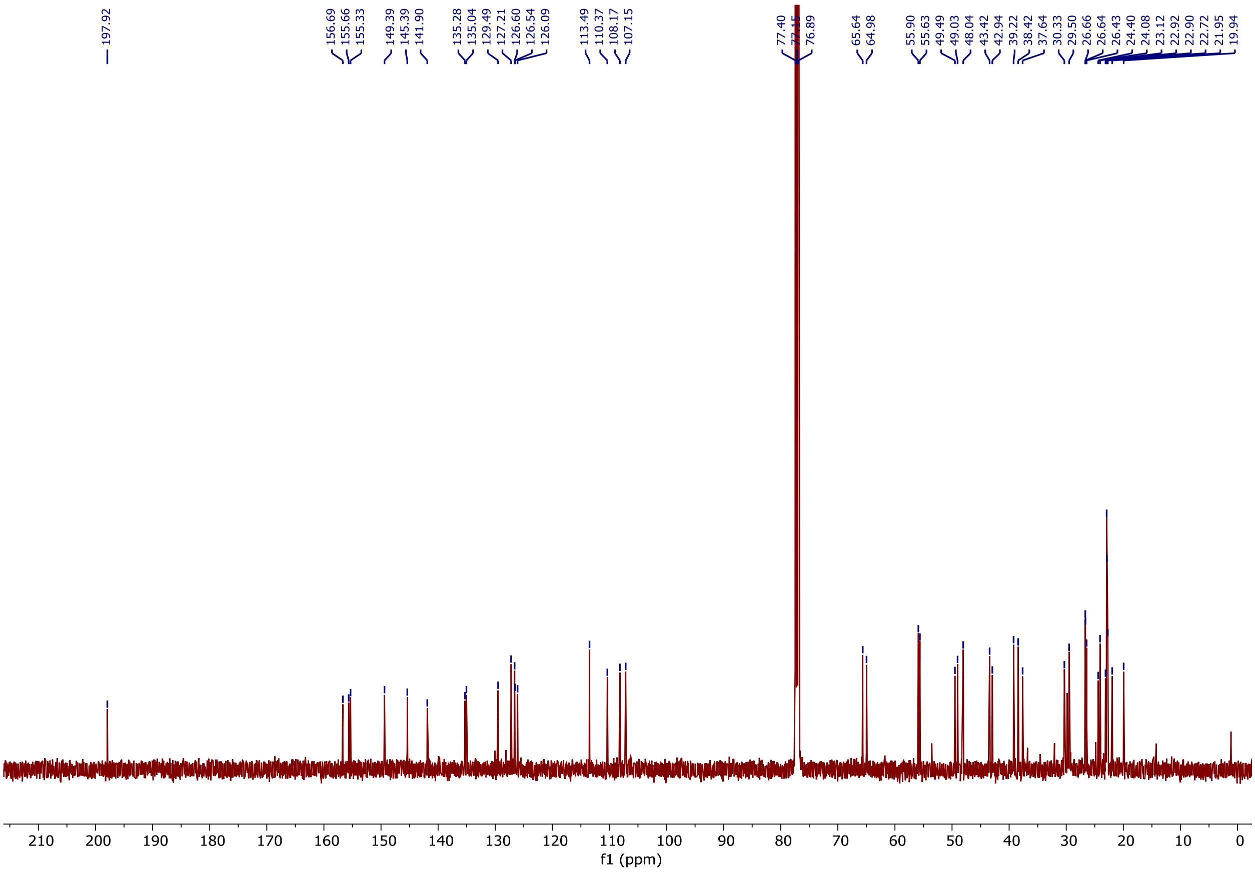

^{13}C NMR (126 MHz, $CDCl_3$) of compound (+)-**31**

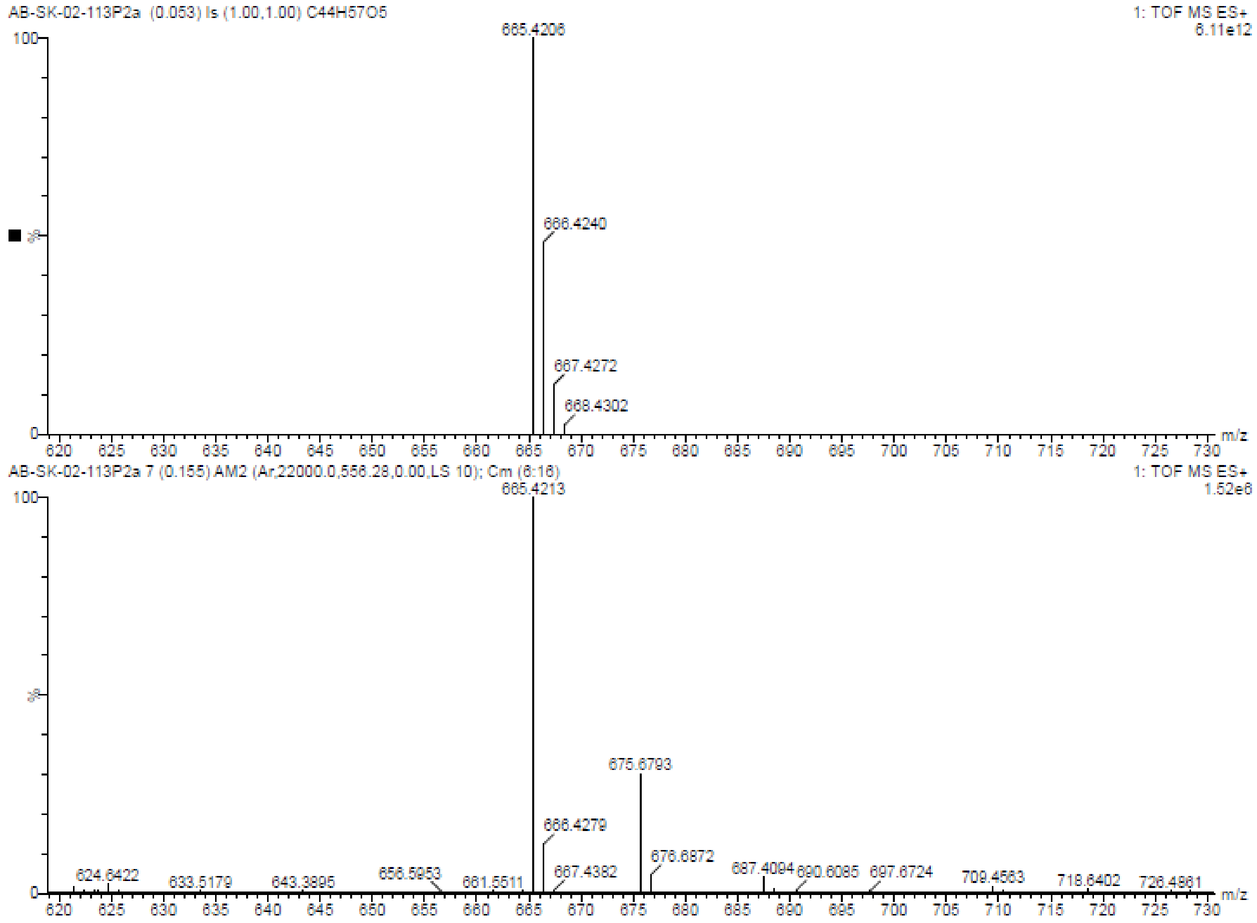

HRMS data of (+)**-31**

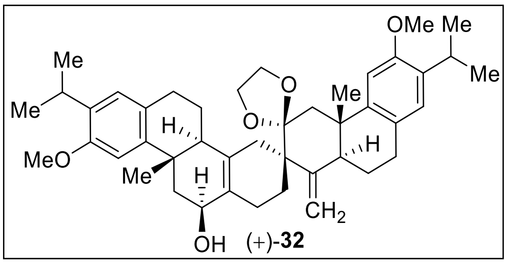

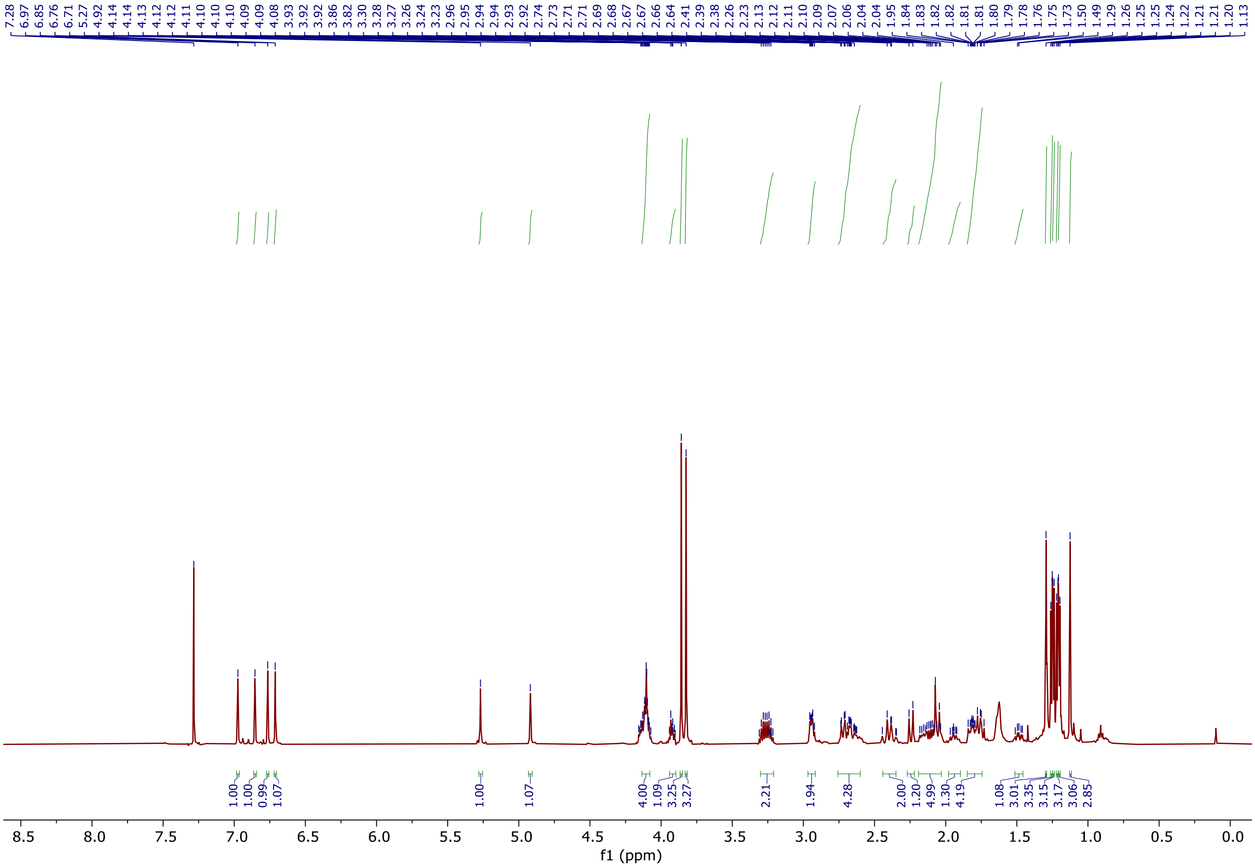

^{1}H NMR (500 MHz, $CDCl_3$) of compound (+)-**32**

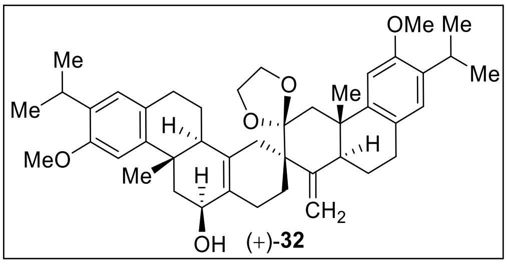

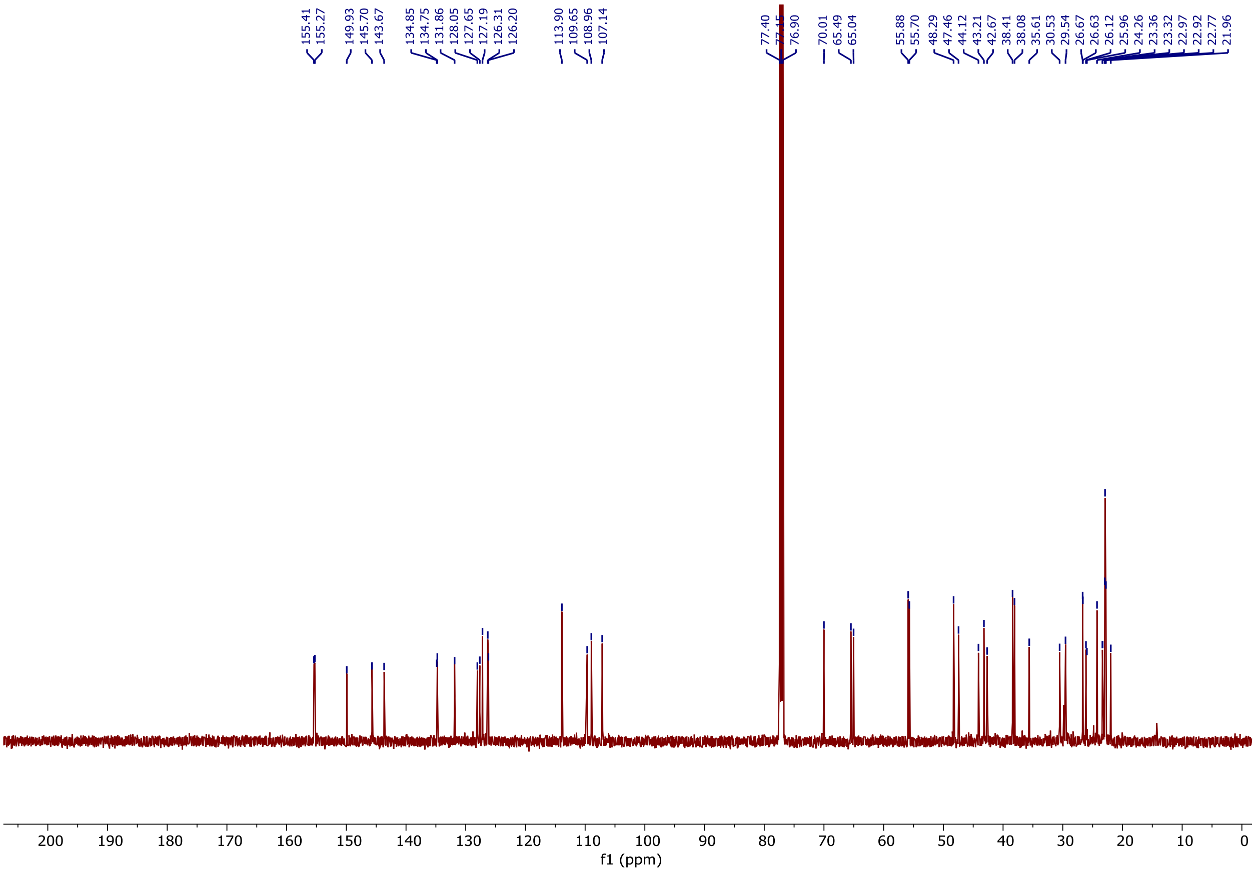

^{13}C NMR (126 MHz, $CDCl_3$) of compound (+)-**32**

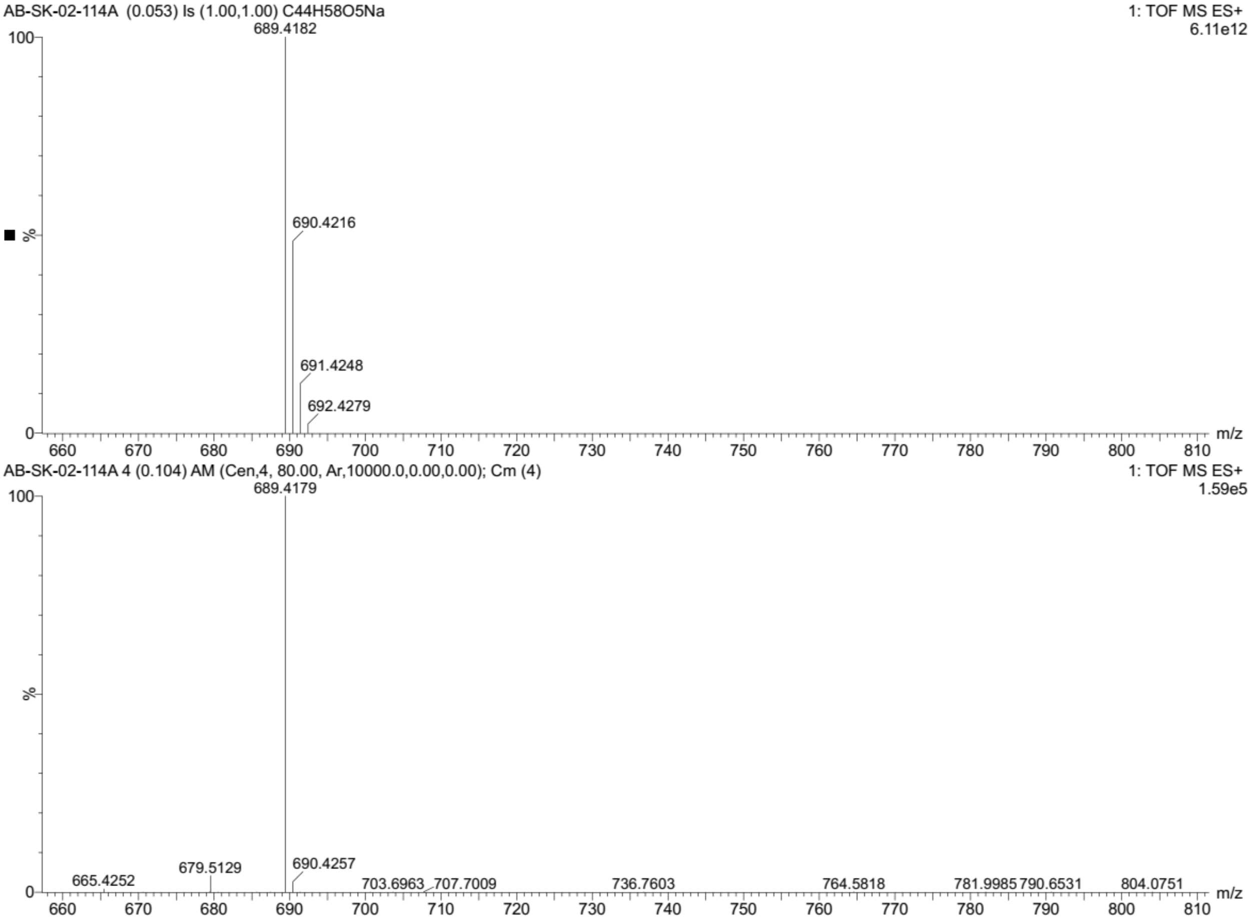

HRMS data of (+)-**32**

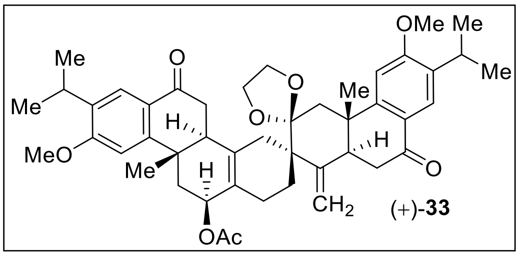

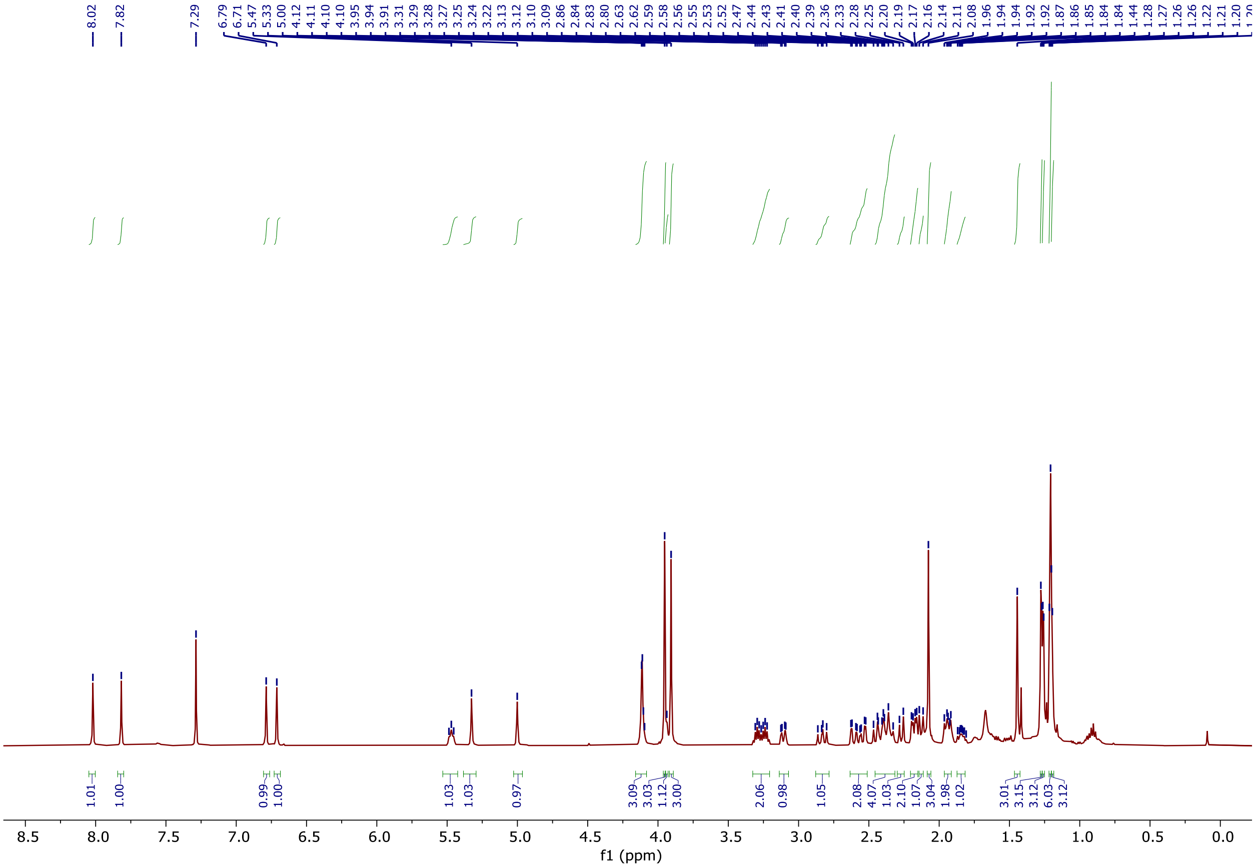

^{1}H NMR (500 MHz, $CDCl_3$) of compound (+)-**33**

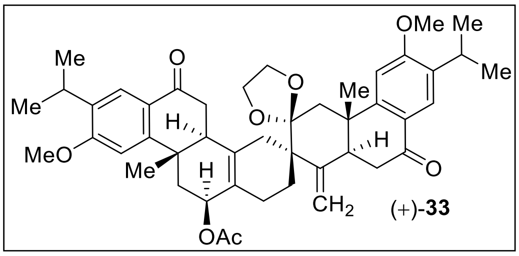

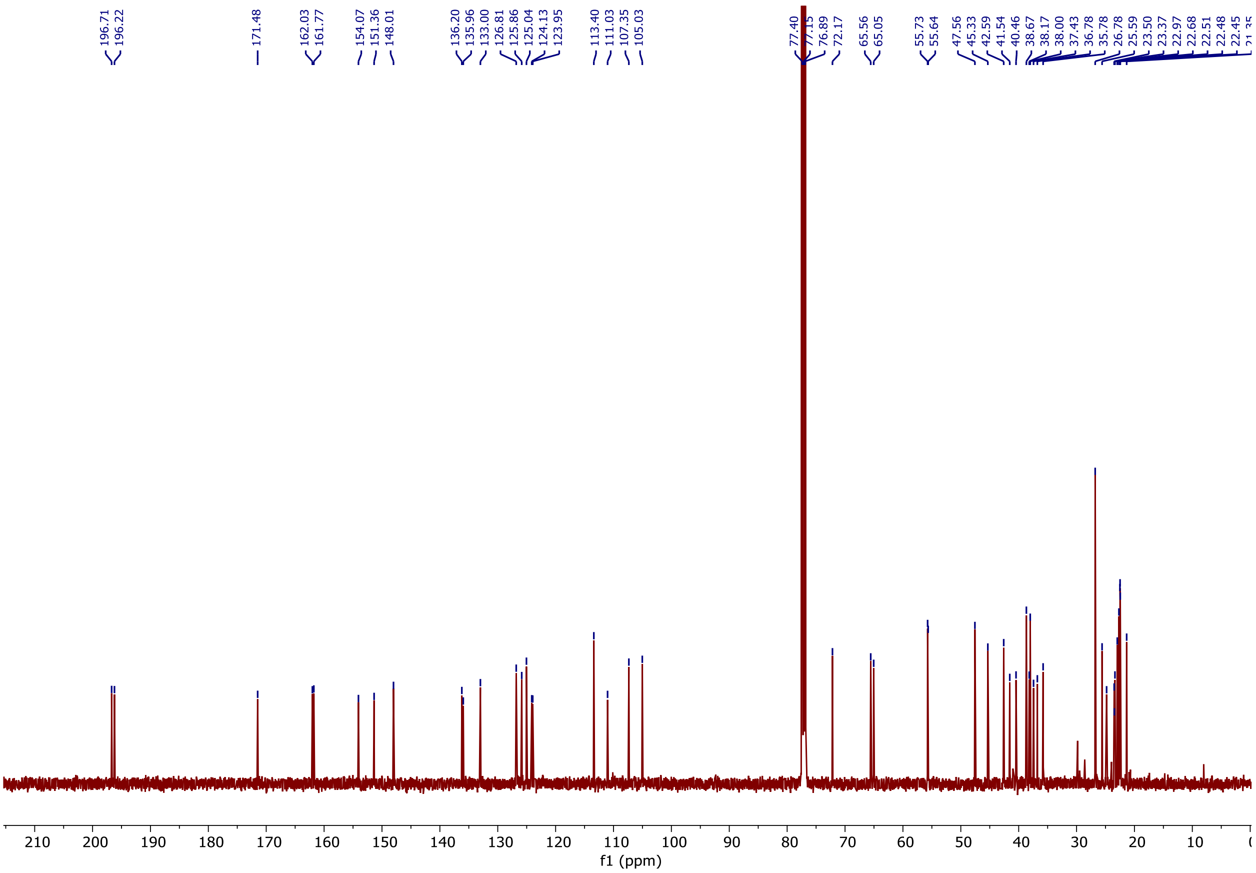

^{13}C NMR (126 MHz, $CDCl_3$) of compound (+)-**33**

Display Report

Analysis Info

Analysis Name	D:\Data\User data\2022\NOV\AB_SK_02122.d	Acquisition Date	11/17/2022 11:06:11 AM
Method	Tune_pos_Mid_July.m	Operator	IISER Kolkata
Sample Name	AB_SK_02122	Instrument	maXis impact 8282001.00127
Comment			

Acquisition Parameter

Source Type	ESI	Ion Polarity	Positive	Set Nebulizer	0.5 Bar
Focus	Active	Set Capillary	3400 V	Set Dry Heater	200 °C
Scan Begin	50 m/z	Set End Plate Offset	-500 V	Set Dry Gas	4.0 l/min
Scan End	3000 m/z	Set Charging Voltage	2000 V	Set Divert Valve	Source
		Set Corona	0 nA	Set APCI Heater	0 °C

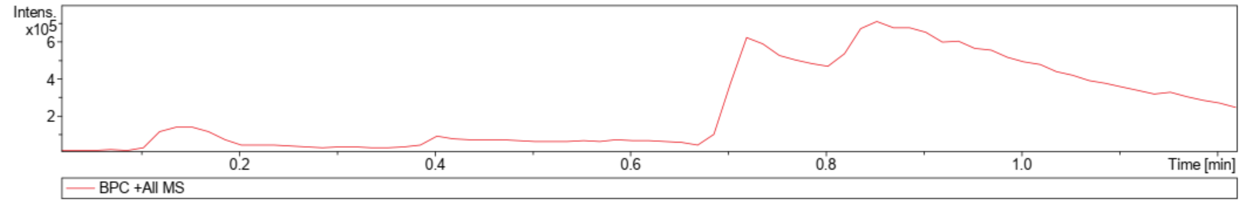

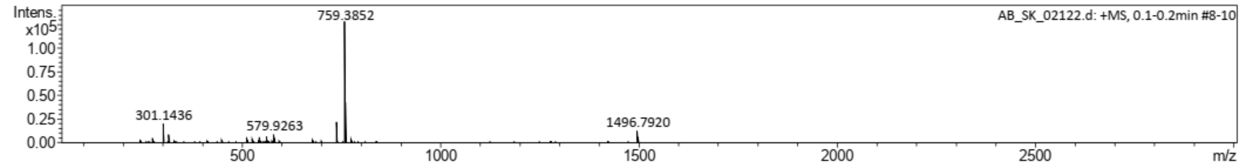

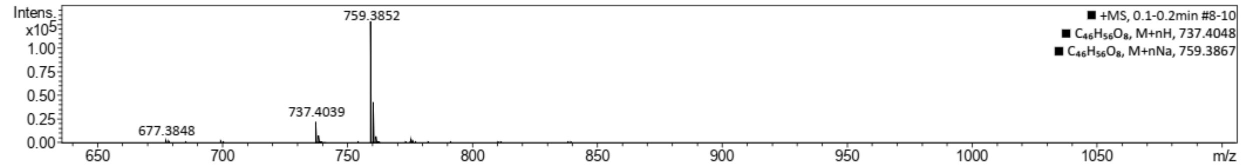

HRMS data of (+)-**33**

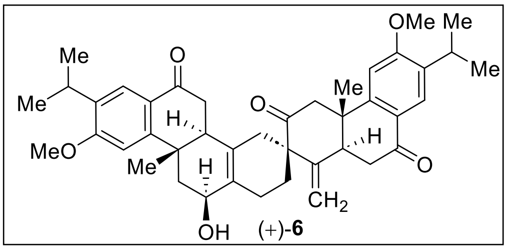

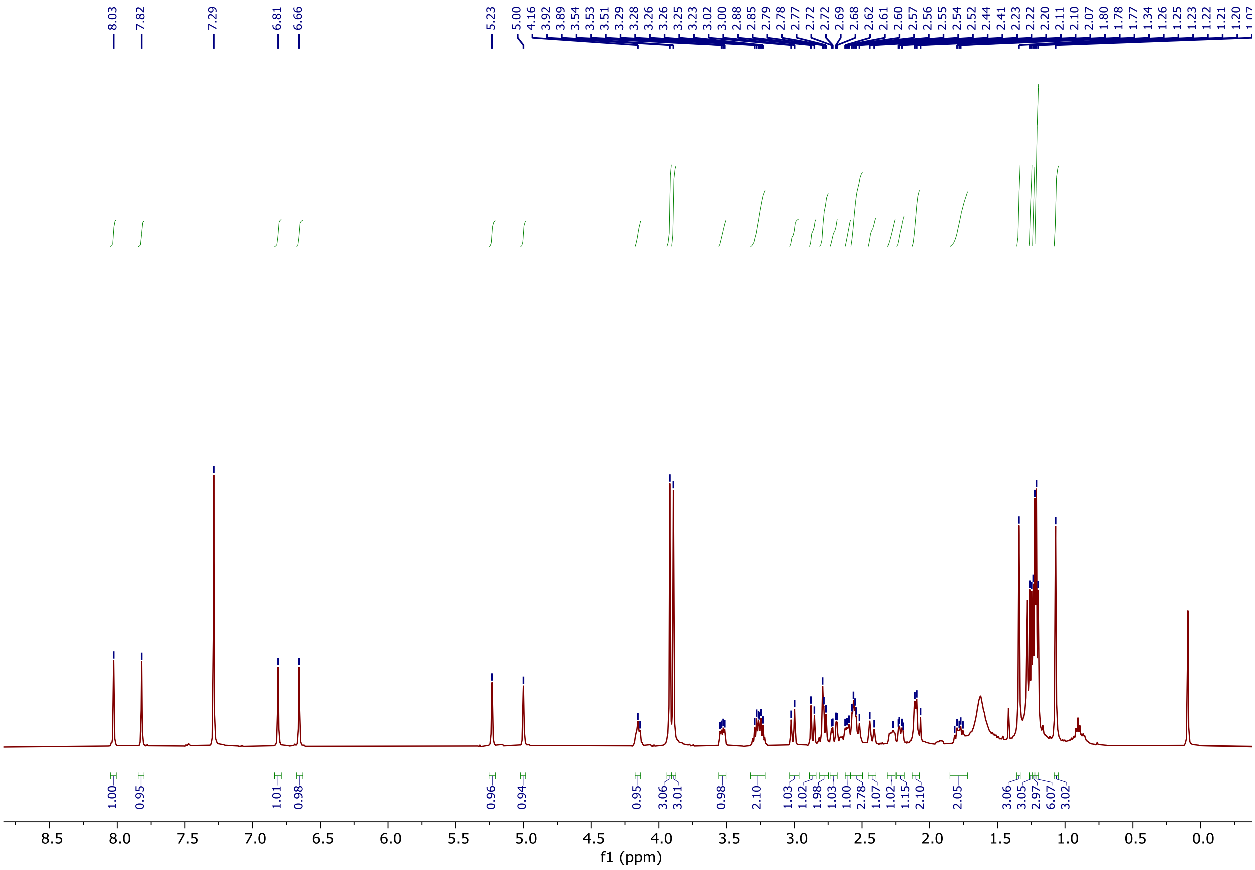

^{1}H NMR (500 MHz, $CDCl_3$) of compound (+)-**6**

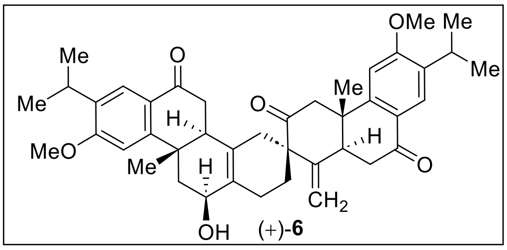

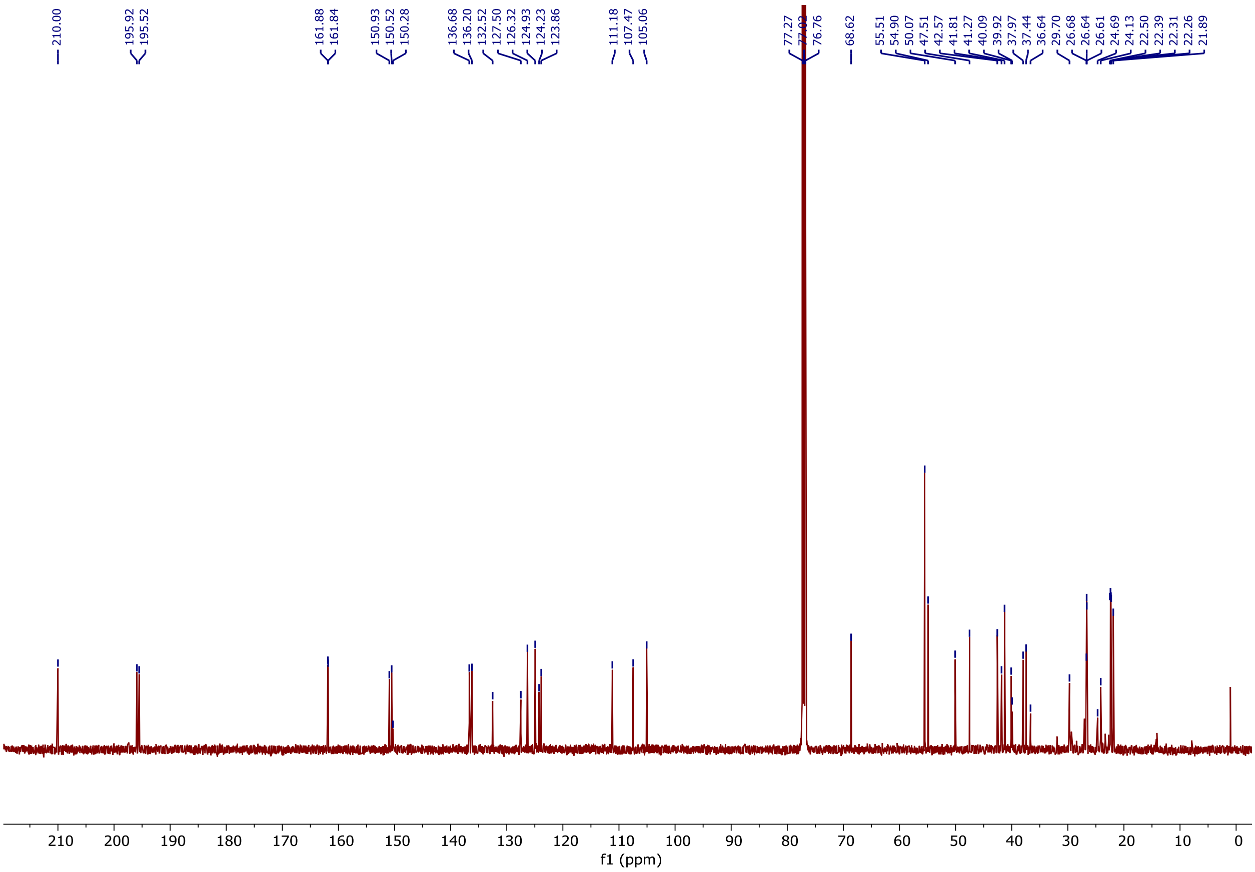

^{13}C NMR (126 MHz, $CDCl_3$) of compound (+)-**6**

.

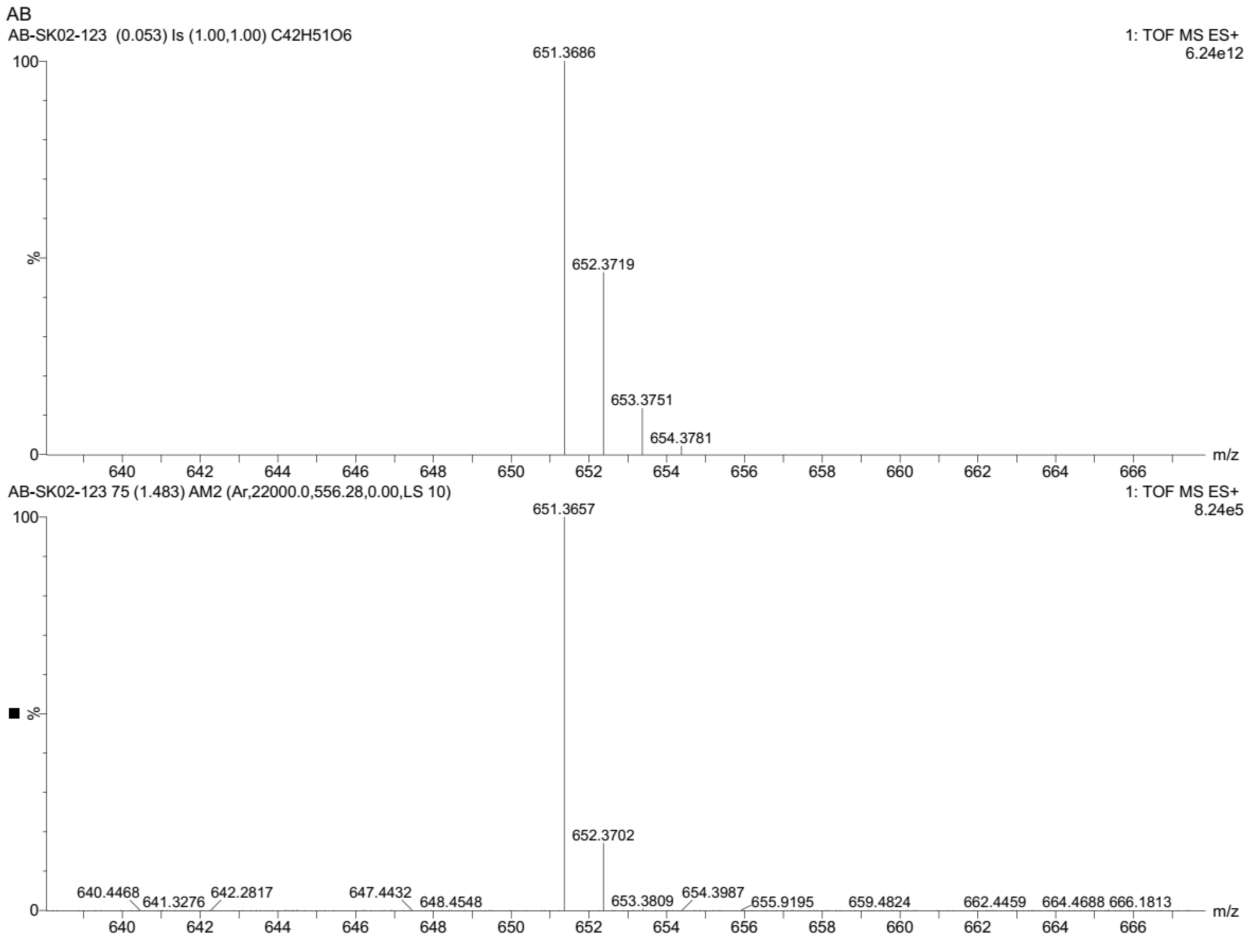

HRMS data of (+)-**6**

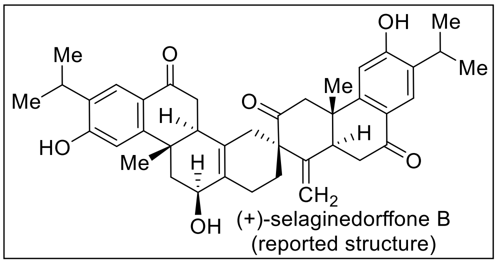

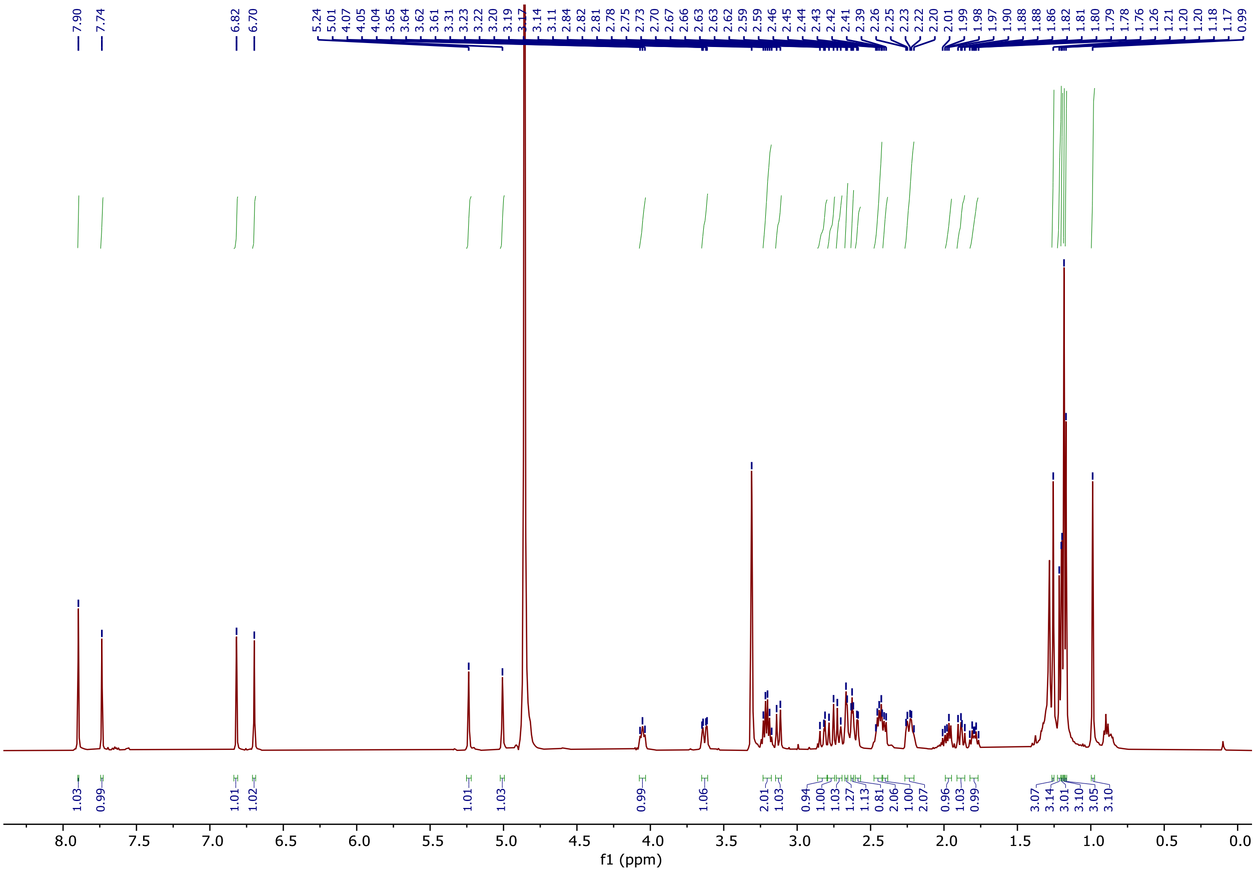

^{1}H NMR (500 MHz, CD_3OD) of compound (+)-**2**

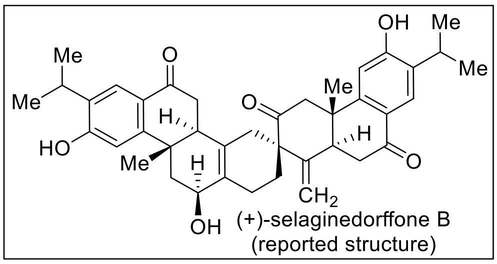

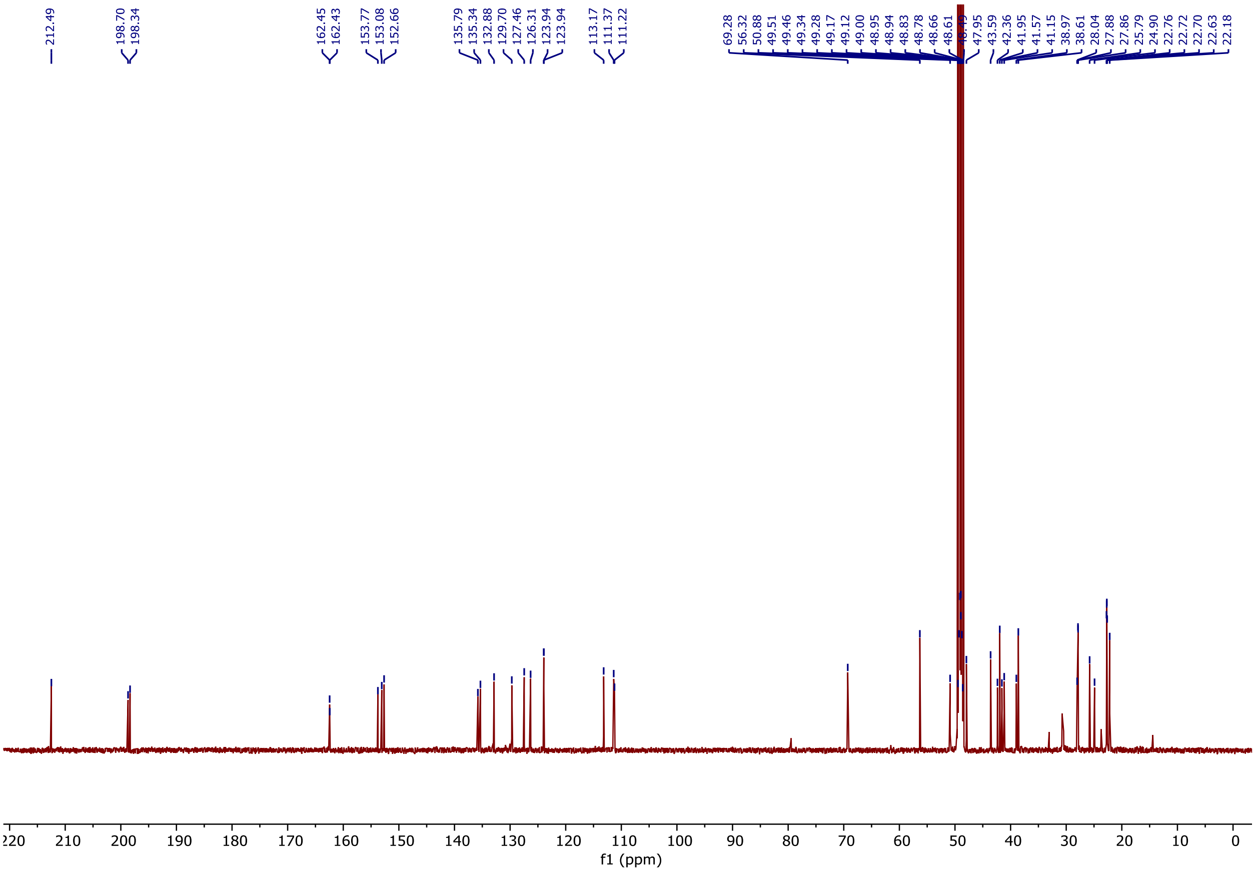

^{13}C NMR (126 MHz, CD_3OD) of compound (+)-**2**

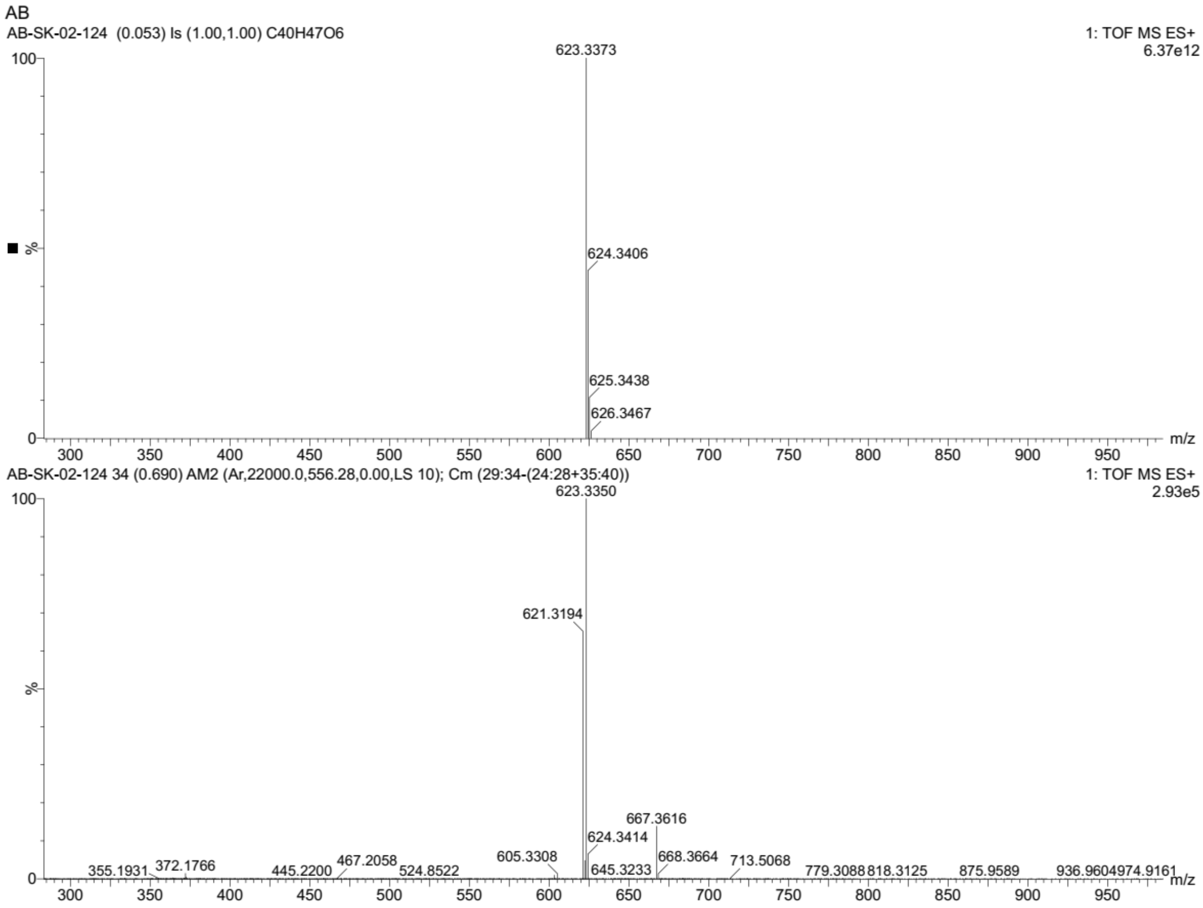

HRMS data of (+)-**2**

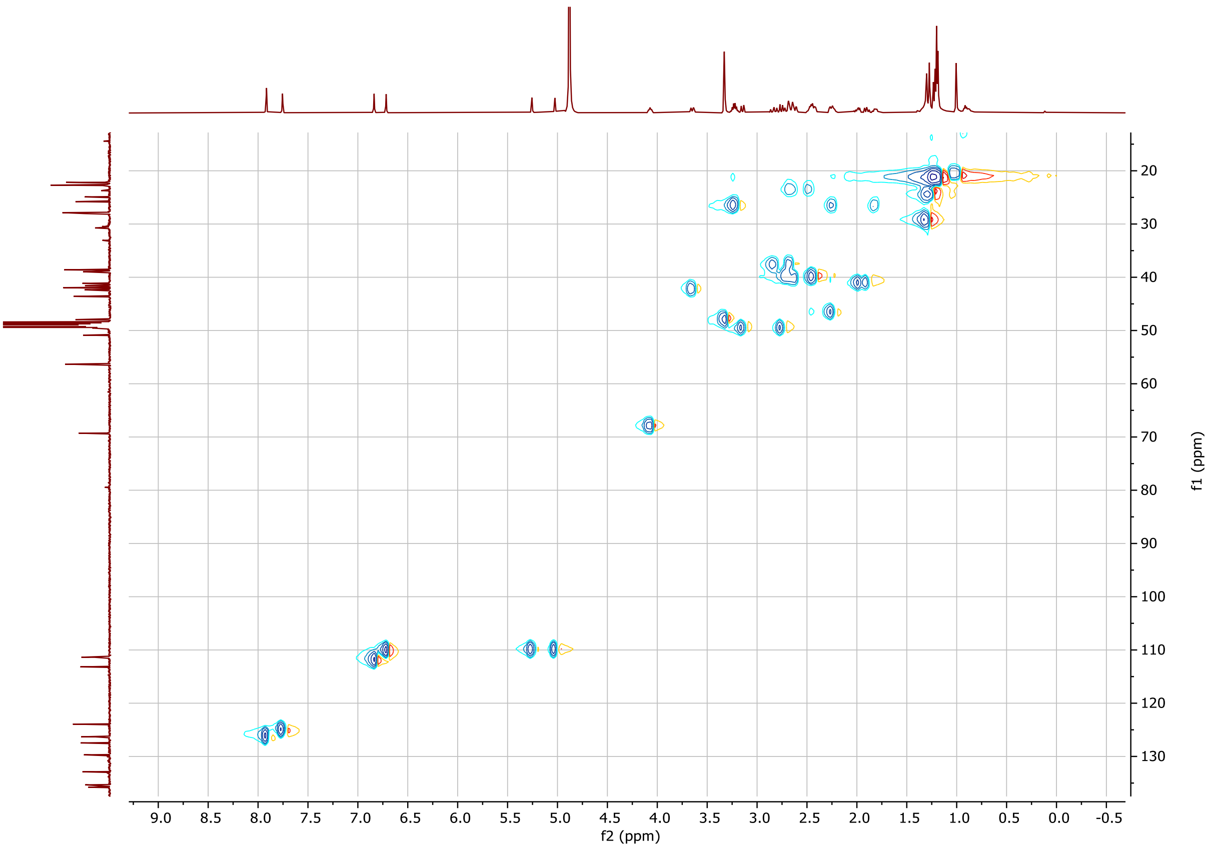

HSQC spectrum of (+)-**2**

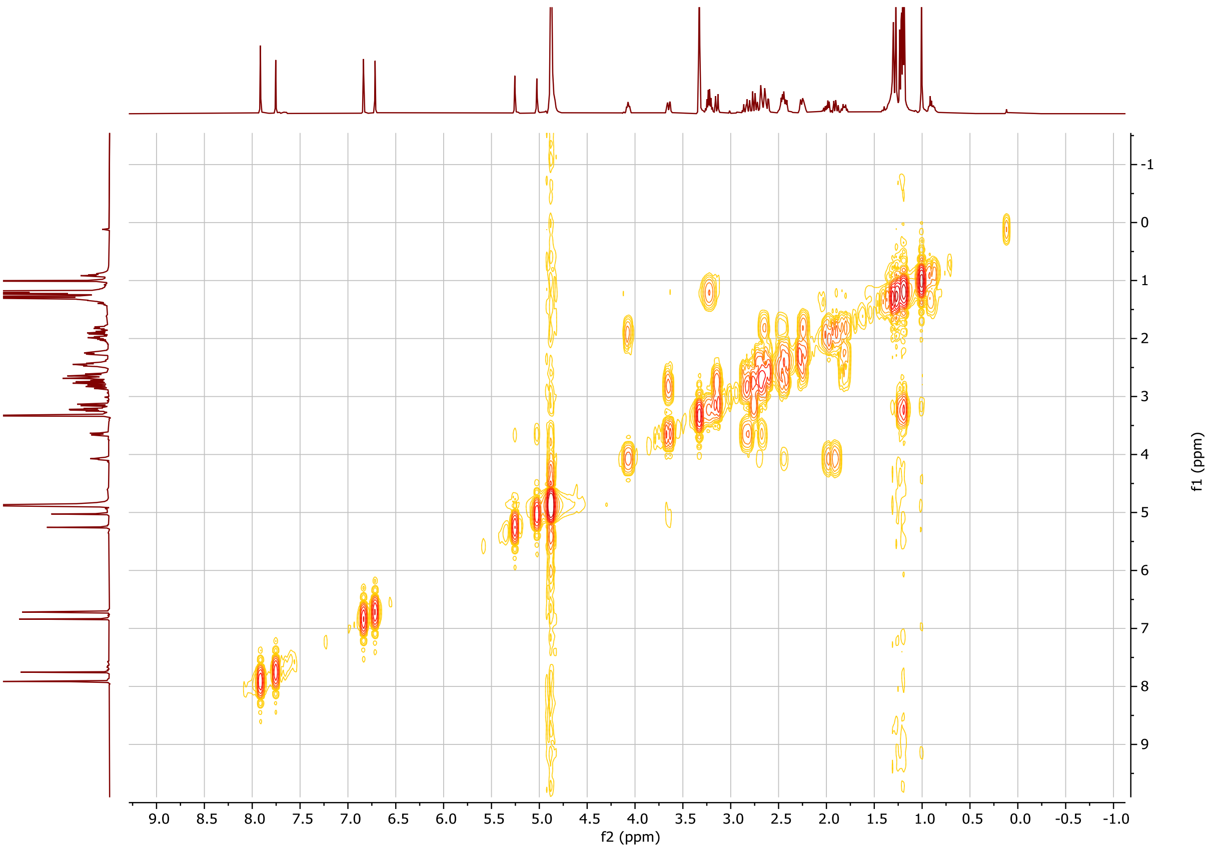

^{1}H-^{1}H COSY spectrum of (+)-**2**

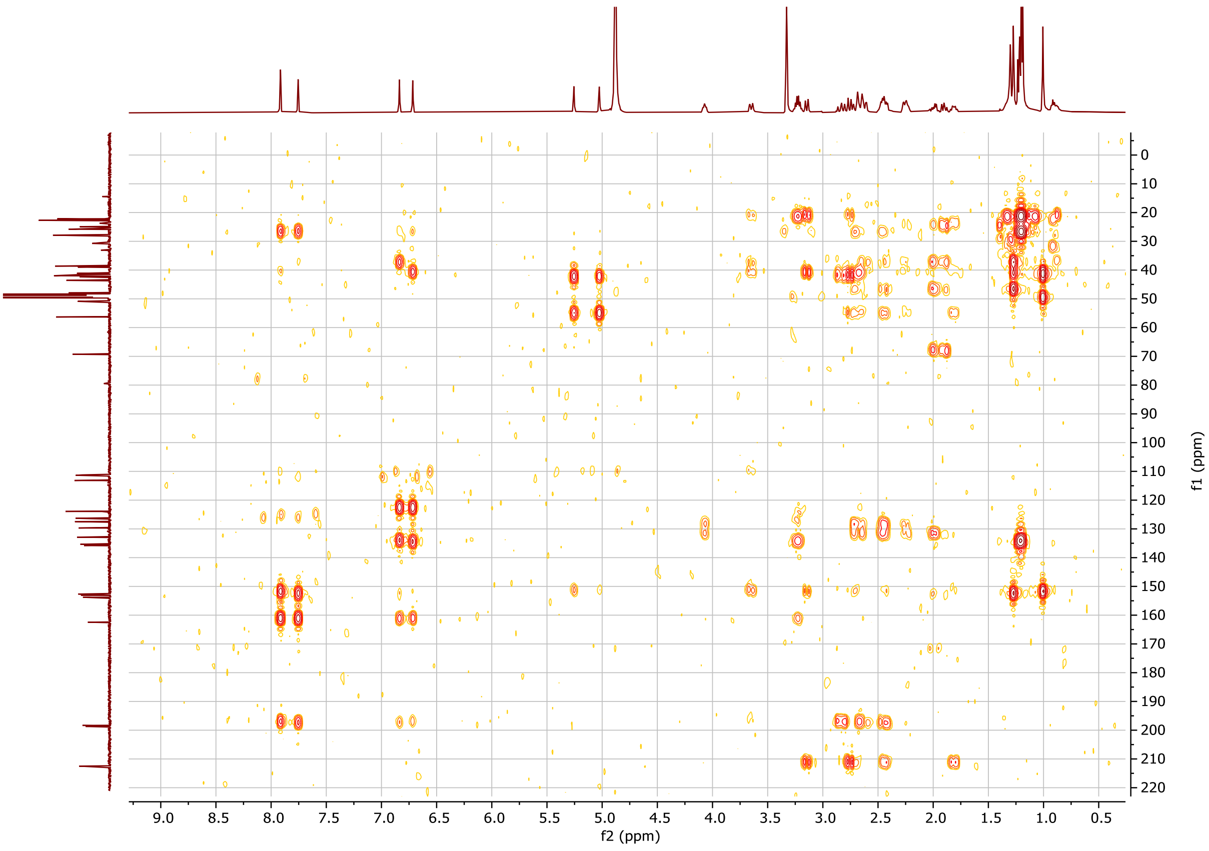

HMBC spectrum of (+)-**2**

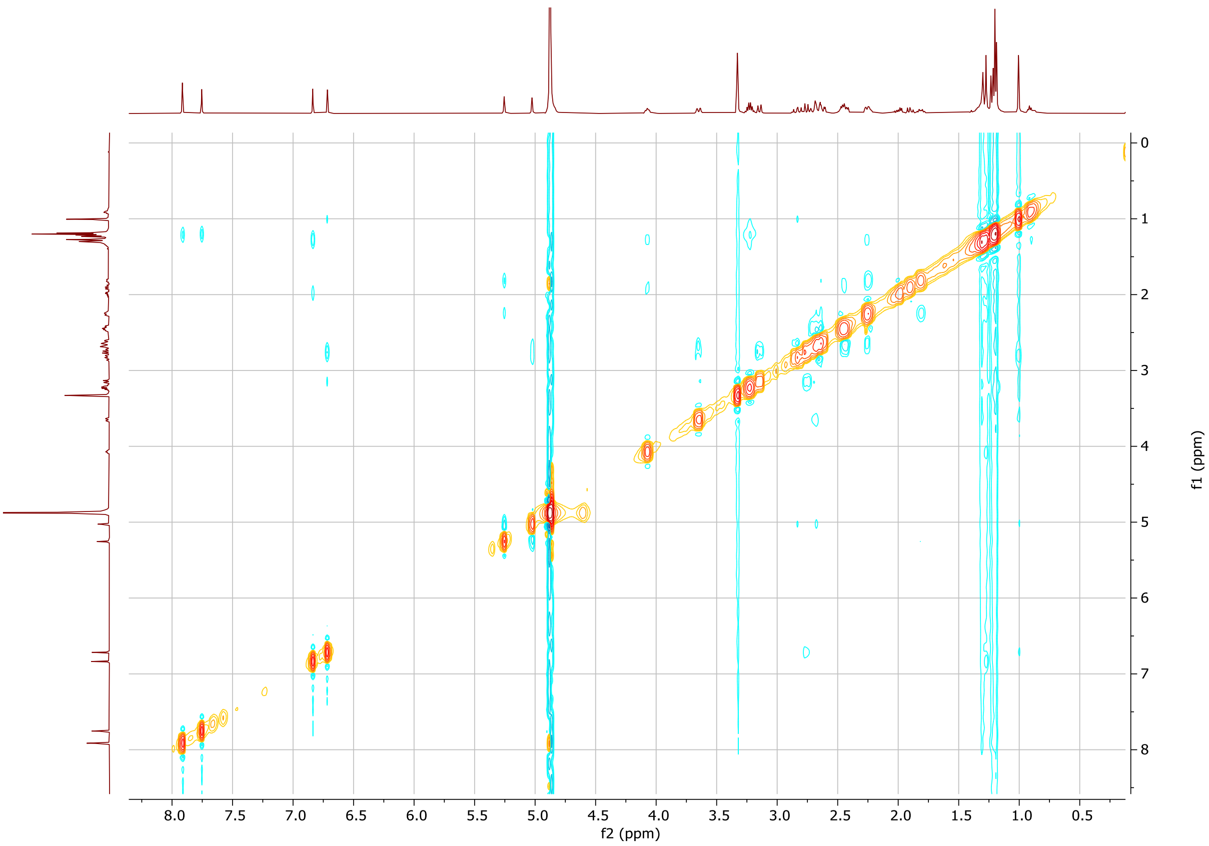

NOESY spectrum of (+)-**2**

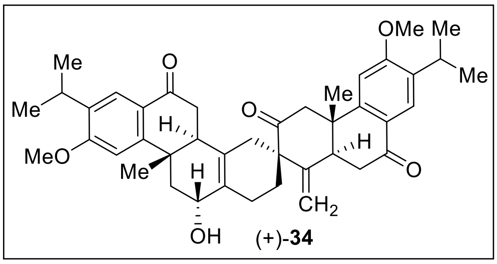

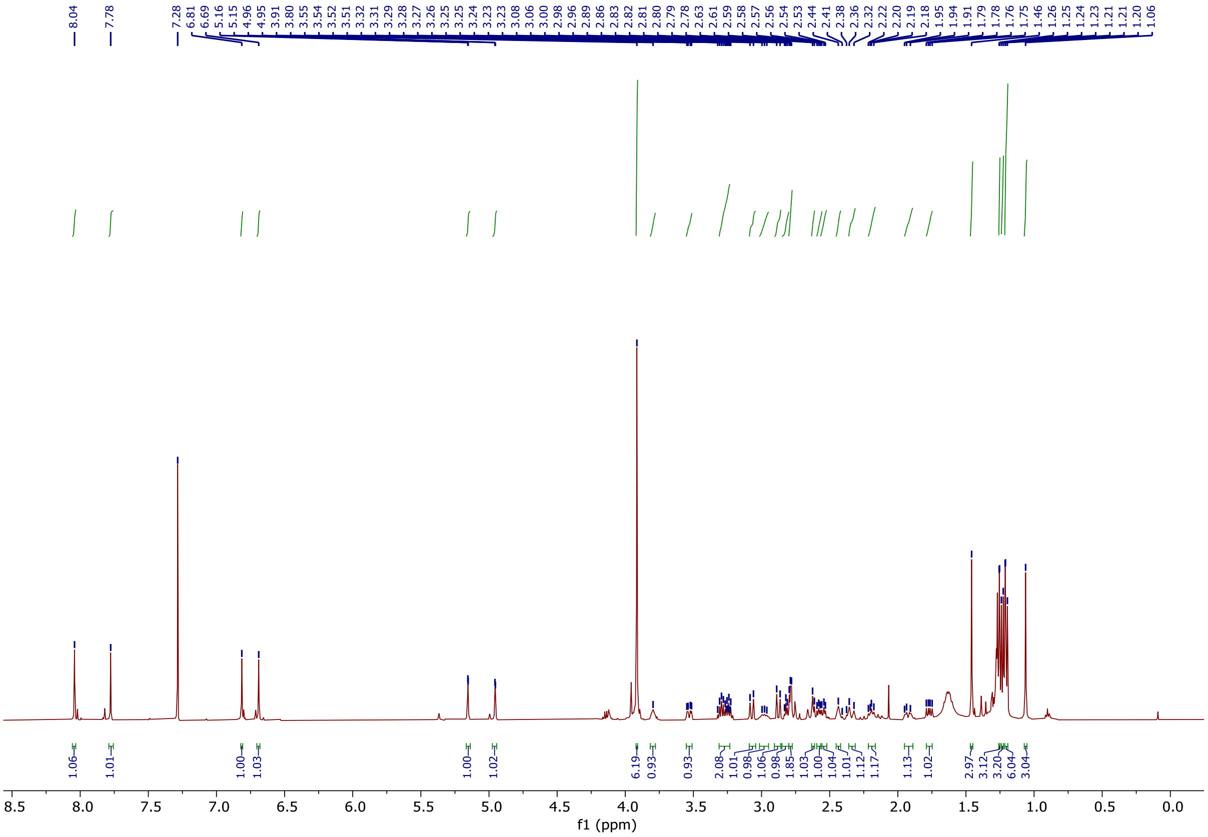

^{1}H NMR (500 MHz, CD_3OD) of compound (+)-**34**

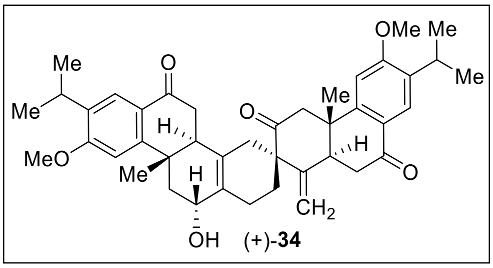

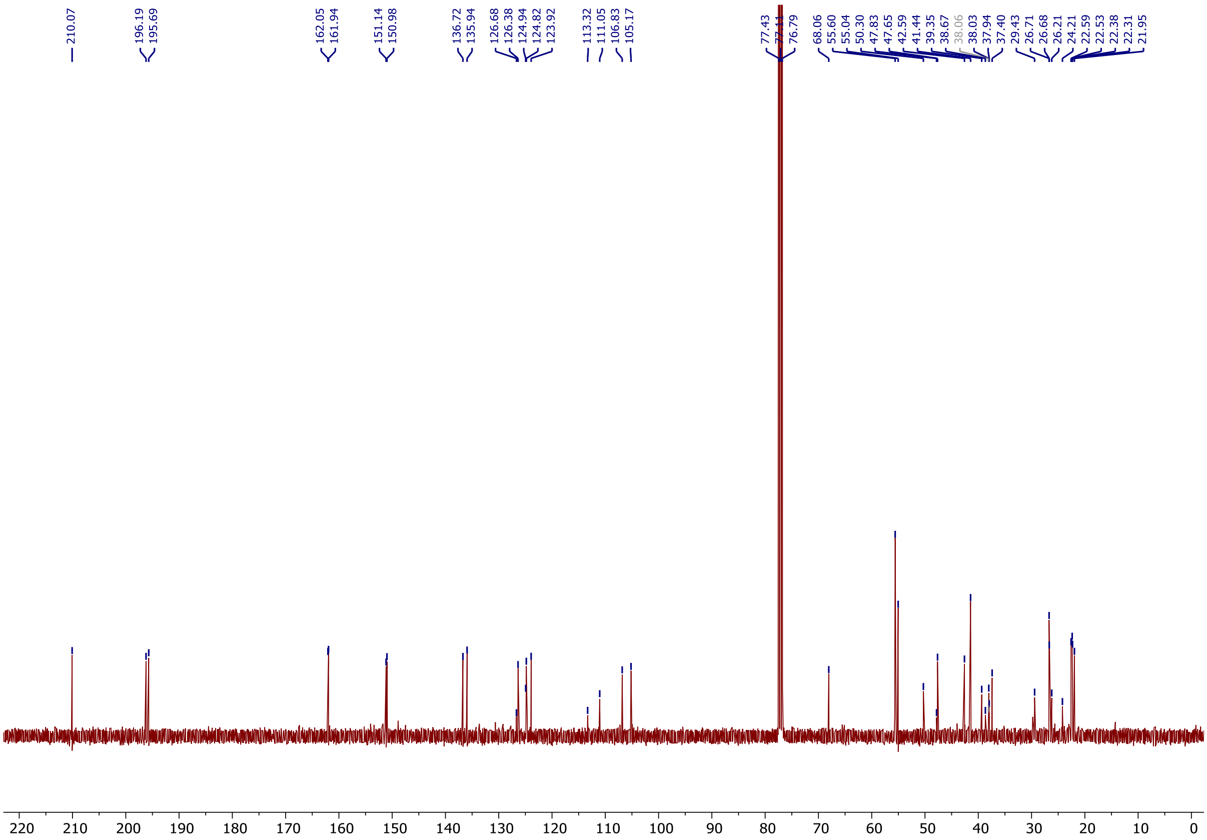

^{13}C NMR (101 MHz, CD_3OD) of compound (+)-**34**

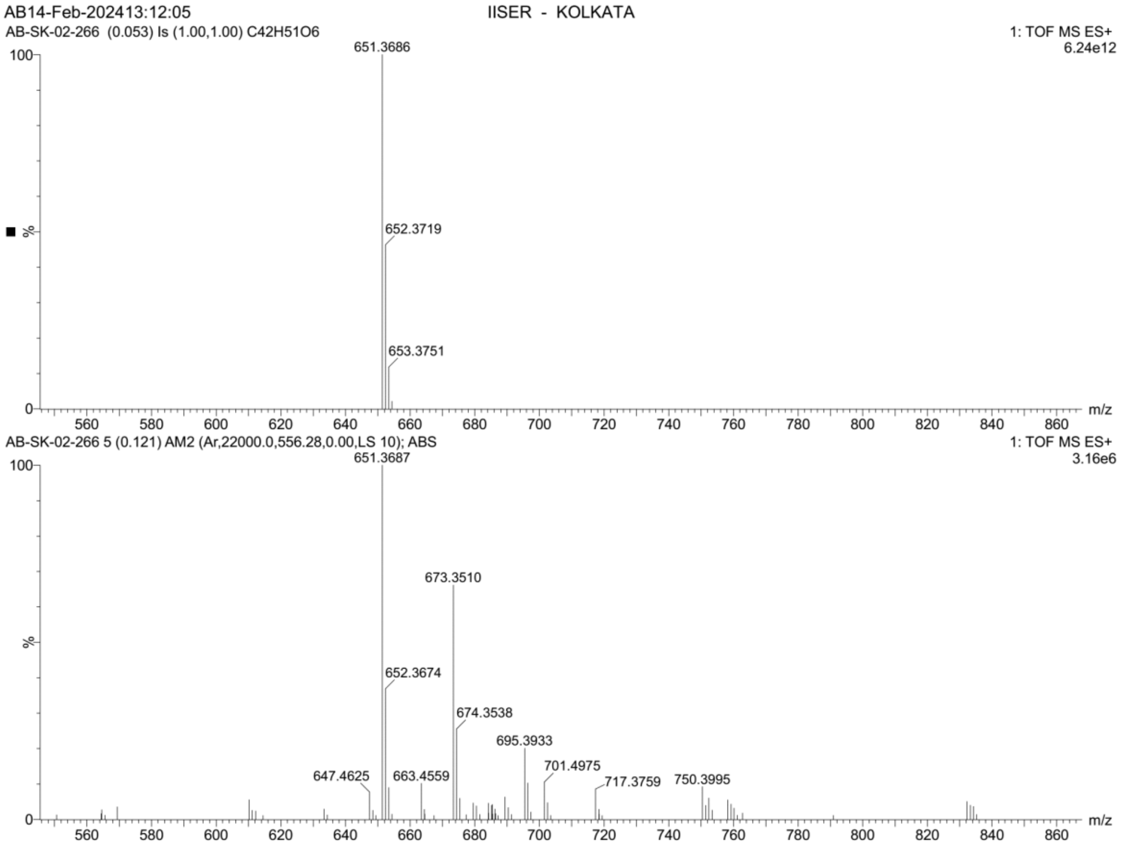

HRMS data of (+)-**34**

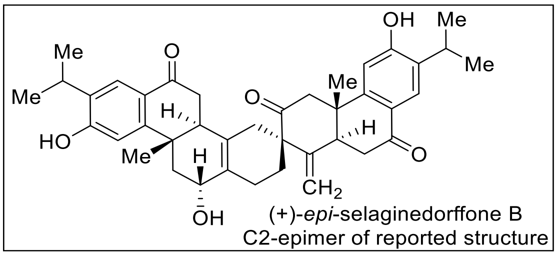

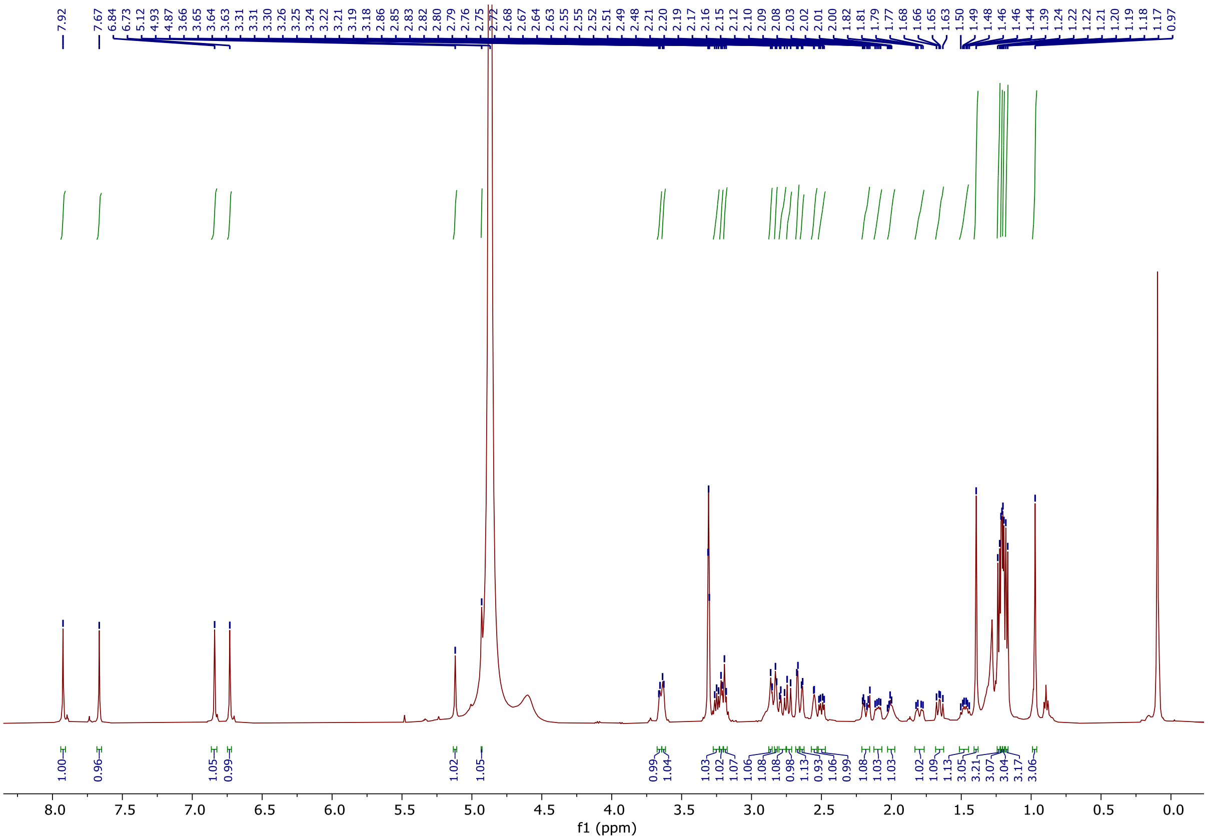

^{1}H NMR (500 MHz, CD_3OD) of compound (+)-***epi*-2**

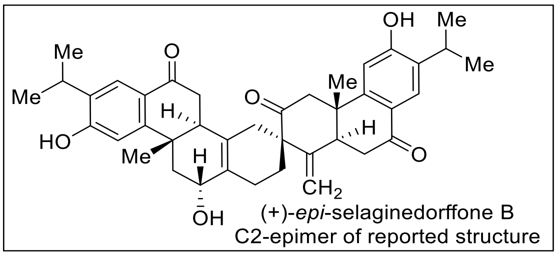

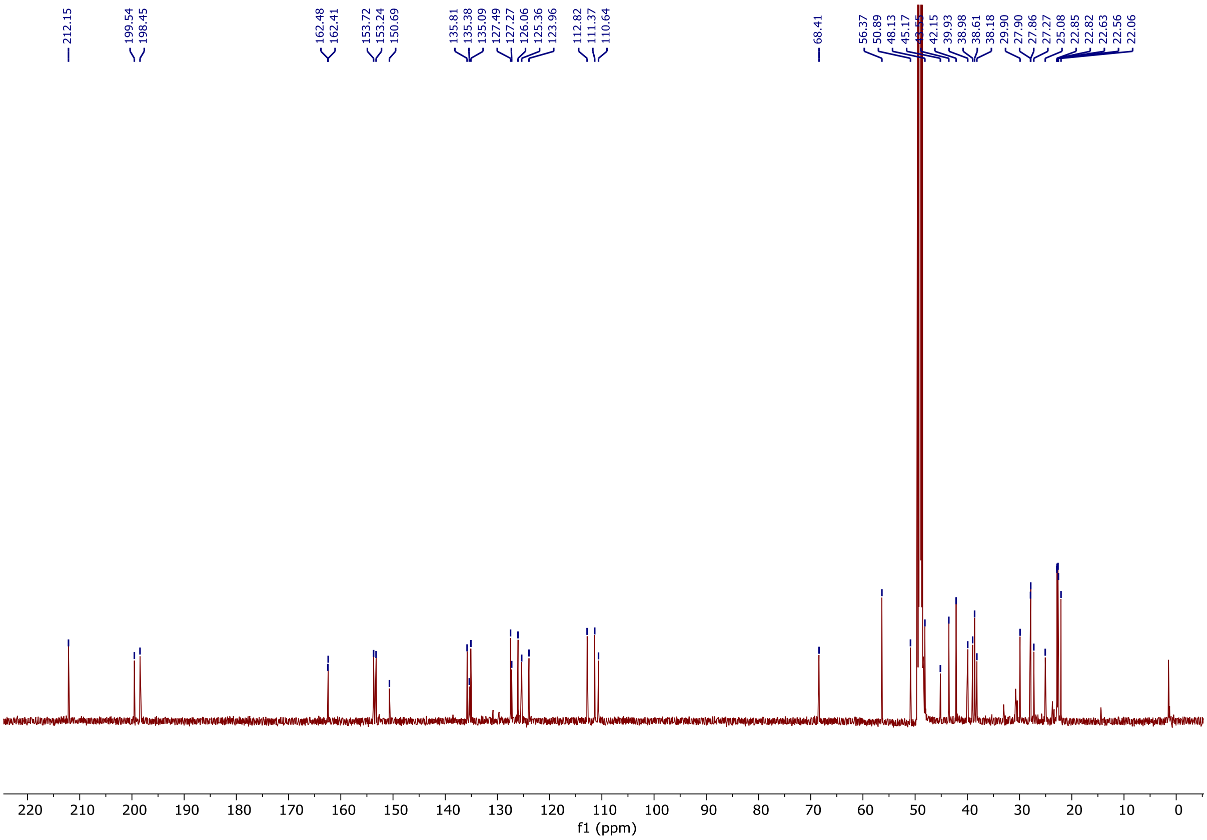

^{13}C NMR (126 MHz, CD_3OD) of compound (+)-***epi*-2**

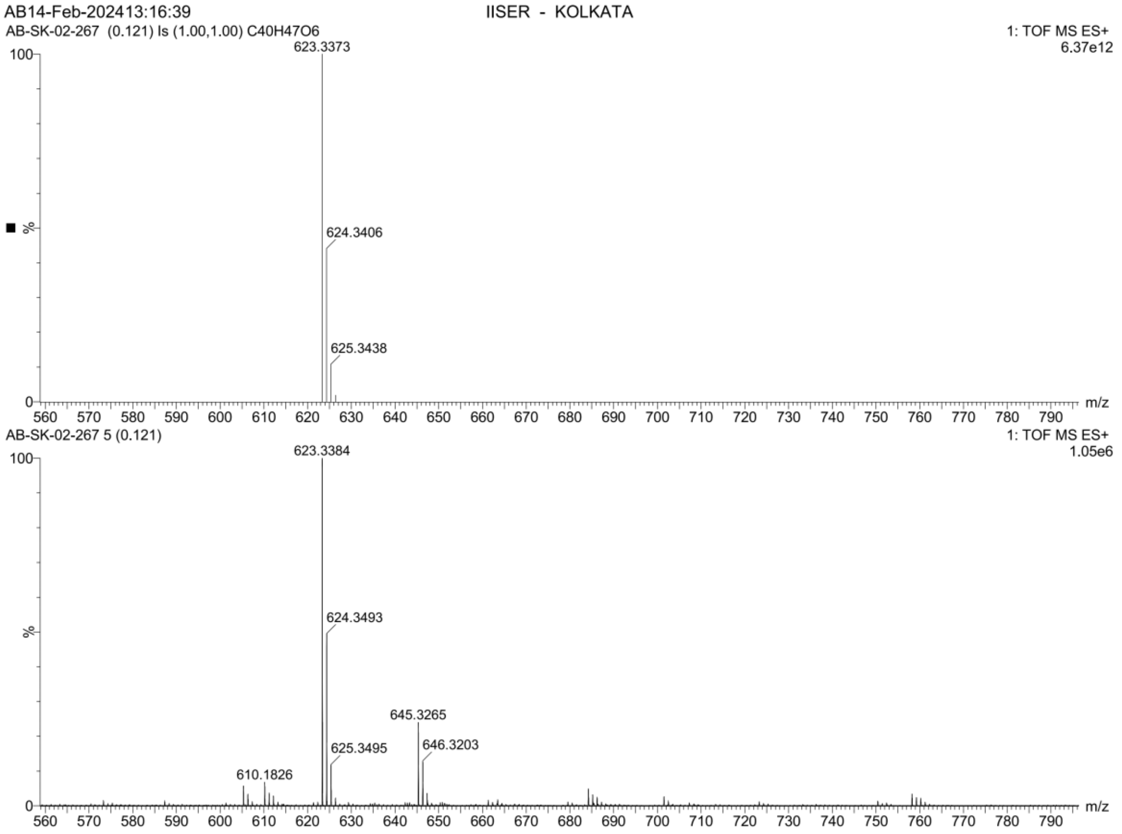

HRMS data of (+)-***epi*-2**

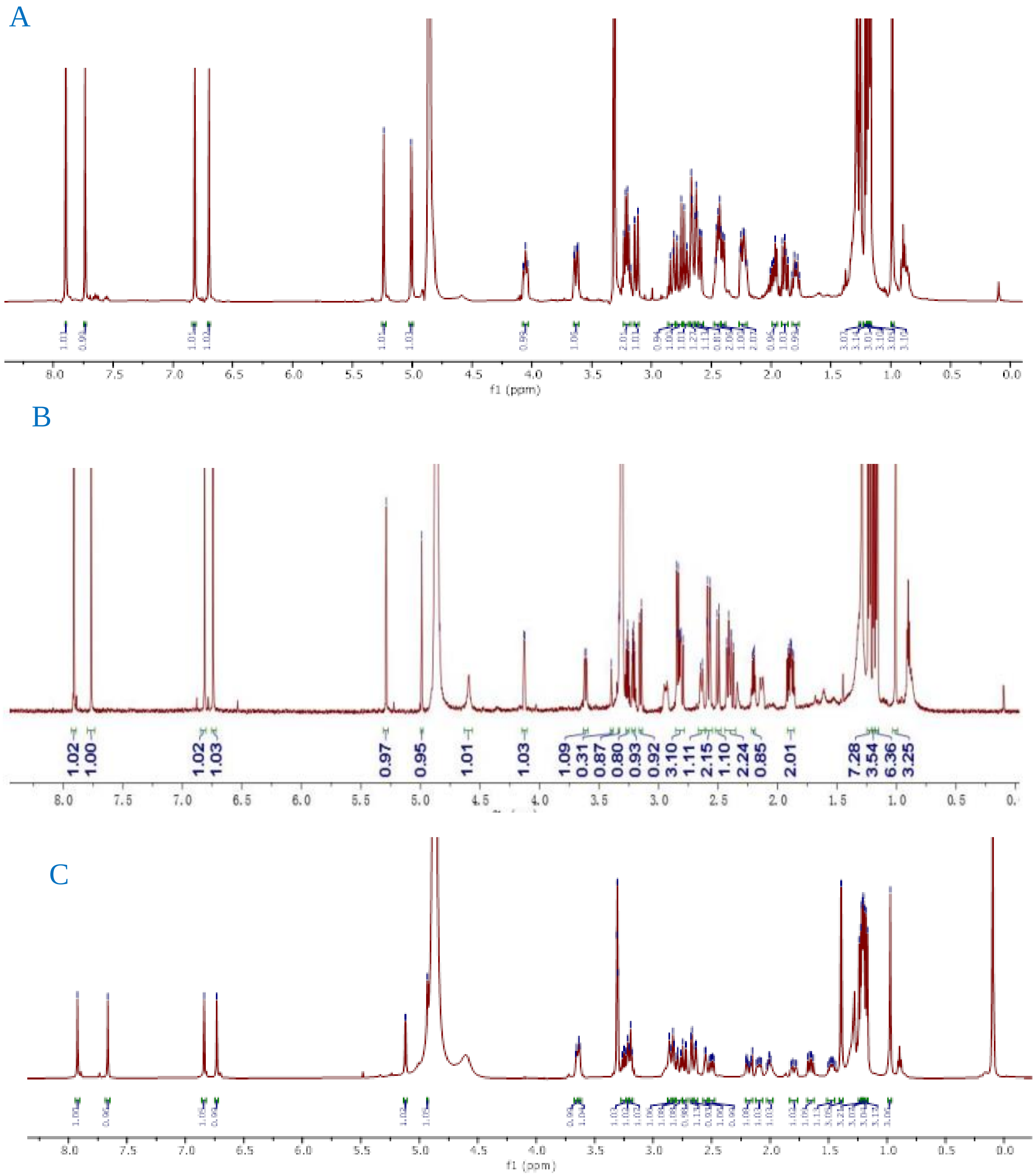

A. ^{1}H-NMR of reported structure of (+)-selaginedorffone B
B. ^{1}H-NMR by Long et al. (Isolation)
C. ^{1}H-NMR of reported structure of (+)-*epi*-selaginedorffone B
