# Supplementary material for: Biomimetic total synthesis of the reported structure of (+)-selaginedorffone B
Source: Chem Sci. 2024 Jul 25;15(36):14946–53. doi: 10.1039/d4sc04103h (PMC11376052; doi:10.1039/d4sc04103h)
Supplement: SC-015-D4SC04103H-s002 [file SC-015-D4SC04103H-s002.pdf]

Supplementary Information (SI) for Chemical Science.
This journal is © The Royal Society of Chemistry 2024

Supplemental material for:

Biomimetic Asymmetric Total Synthesis of the Reported Structure of Selaginedorffone B

Sourav Kundu,[a] Debgopal Jana,[b] Nilangshu Mandal,[c] Ayan Mondal,[b] Ranjit Murmu,[b] Nanda Kishore Roy,[b] Ayan Datta*[c] and Alakesh Bisai*[a,b]

[a]Department of Chemistry, Indian Institute of Science Education and Research Kolkata, Mohanpur Campus, Nadia, Kalyani, 741 246, West Bengal, India.

[b]Department of Chemistry, Indian Institute of Science Education and Research Bhopal, Bhopal Bypass Road, Bhopal 462 066, Madhya Pradesh, India.

[c]School of Chemical Sciences, Indian Association for the Cultivation of Science, 2A and 2B Raja S. C. Mullick Road, Jadavpur – 700032, Kolkata, West Bengal, INDIA

Table of Contents

Materials and Methods	S3
Synthesis of the racemic allyl alcohol (±)-**9**	S4-S5
Johnson-Claisen Rearrangement of (±)-**9**	S5-S7
Reduction of γ, δ-unsaturated ester (**11**)	S7-S8
Synthesis of the propargylic alcohol (±)-**12**	S8-S9
DMP oxidation of the propargyl alcohol **12**	S9-S10
Noyori's asymmetric transfer hydrogenation of **13**	S11-S13
Synthesis of the propargyl benzoate derivative (+)-**12'**	S13-S14
Synthesis of the alkyne-ene cyclization precursor (–)-**14**	S15-S16
In(III)-catalyzed alkyne-ene cyclization of (–)-**14**	S16-S18
Desilylation of TBS-protected allyl alcohol (+)-**15**	S18-S19
Oxidation of the allyl alcohol (+)-**16**	S20-S22
Wittig olefination of enone (+)- **18**	S23
Attempts toward the allylic oxidation of diene (+)-**19**	S24-S25
Attempts toward the Cα-functionalization of enone (+)-**18**	S24-S25
Rubottom oxidation of silyl enol ether (+)-**18**	S27-S30
Silylation of hydroxy enone (+)-**22'**	S30-S31

Wittig olefination of silyloxy enone (+)-**22** S31-S32
Benzylic oxidation of silyloxy diene (+)-**23** S32-S34
Desilylation of silyloxy diene (+)-**24** S34-S35
Mitsunobu reaction of hydroxy diene (+)-**25** S35-S36
Synthesis of the biomimetically hypothesized diene (+)-**3** S37
Attempts toward the oxidation of the hydroxy diene (+)-**25** S38-S40
Equivalent controlled DMP oxidation of the hydroxy diene (+)-**25** S41
DMP oxidation of the hydroxy diene (+)-**3** S41-S42
Desilylation of silyloxy diene (+)-**23** S42-S43
DMP oxidation of the hydroxy diene (+)-**20** S44-S45
Luche reduction of the enone (+)-**30** S45-S47
Chemoselective ketal protection of (+)-**30** S47-S49
Diastereoselective Luche reduction of the enone (+)-**31** S49-S50
Synthesis of the benzylic ketone (+)-**33** S50-S52
Synthesis of the bis-aryl methyl ether (+)-**6** S52-S53
Characterization of reported structure of Selaginedorffone B S54-S58
Synthesis of *epi*-selaginedorffone B S59-S68
X-Ray crystallographic information of compound (+)-**25**, (+)-**27**, (+)-**30** S65-S69
Computational Details S69-S81
References S82

Materials and Methods

Unless otherwise stated, reactions were carried out using oven dried glass ware with Teflon coated magnetic stirring bars were used to stir the reactions. The Syringe was used to transfer solvents and liquid reagents. Tetrahydrofuran (THF) Diethyl ether (Et_2O) was distilled over sodium/benzophenone ketyl. Dichloromethane CH_2Cl_2) was distilled over calcium hydride. All other solvents like MeOH, EtOAc, DMF, Dichloroethane (DCE) and reagents were used as received. Reaction temperatures above 25 °C were maintained by using an oil bath on a magnetic stirrer. Thin layer chromatography (TLC) analysis was performed by using silica gel precoated plates (0.25 mm) 60 (F-254), Visualized by UV irradiation, yellow dip stain and other stains. Silica gel of particle size 230-400 and 100-200 mesh were used to perform flash chromatography. Digital melting point apparatus is used to record the melting points. ^{1}H-NMR spectra were recorded by using 400, 500 MHz spectrometers, ^{13}C-NMR operating frequencies are 101, 126 MHz respectively. Chemical shifts (δ) are reported in ppm relative to the residual solvents ($CDCl_3$) signal (δ = 7.29 for 1H NMR and δ = 77.0 for ^{13}C NMR) and (CD_3OD) signal (δ = 3.31 for ^{1}H NMR and δ = 49 for ^{13}C NMR). Data for ^{1}H NMR spectra are reported as follows: chemical shift (multiplicity, coupling constants, and number of hydrogen). Abbreviations are as follows: s (singlet), d (doublet), t (triplet), q (quartet), m (multiplet), br (broad). IR spectra were recorded on a FT-IR system (Spectrum BX) and are reported in frequency of absorption (cm^{-1}). Only selected IR absorbencies are reported. High Resolution Mass Spectrometry (HRMS) data was recorded on MicrOTOF-Q-II mass spectrometer using methanol as solvent.

Synthesis of the allyl alcohol (±)-**9:**

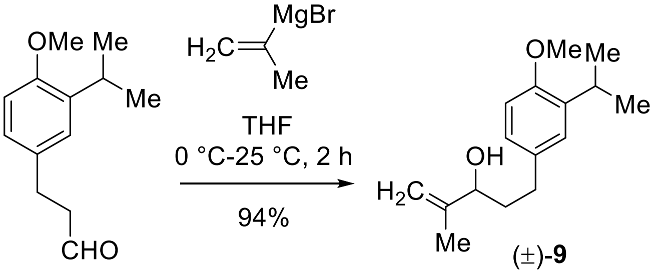

An oven-dried round-bottom flask was charged with the literature known 3-(3-isopropyl-4-methoxyphenyl)propanal[1] (15 g, 72.714 mmol, 1.0 equiv.) in anhydrous THF (120 mL) and was cooled to 0 °C, over an ice bath followed by addition of isopropenyl magnesium bromide (0.5 M in THF, 153 mL, 76.350 mmol, 1.05 equiv.) over a period of 30 minutes with continuous stirring at the same temperature. After the complete addition of the Grignard reagent, the ice bath was removed and stirred at 25 °C for an additional 2 h until the full consumption of the starting material. After completion of the reaction (as judged by running TLC), was quenched with saturated NH_4Cl solution (30 mL). The organic layer from the biphasic solution was separated by a separatory funnel and the aqueous phase was further extracted with EtOAc (25 mL X 2). The combined organic layers were washed with brine, dried over Na_2SO_4, and concentrated in vacuo under reduced pressure. Finally, the crude product was purified through column chromatography using EtOAc in *n*-hexane (10% EtOAc in *n*-hexane) to afford the allyl alcohol (±)-**9** as colorless oil (16.98 g; 94% yield).

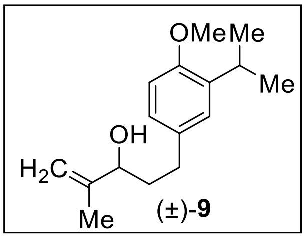

5-(3-isopropyl-4-methoxyphenyl)-2-methylpent-1-en-3-ol [(±)-**9]**: (±)-**9** was obtained as a colorless oil (72.714 mmol, 16.98 g of product, 94% yield); R_f = 0.4 (10% EtOAc in *n*-hexane).

^{1}H NMR (500 MHz, $CDCl_3$**)** δ 7.07 (s, 1H), 7.02 (d, *J* = 8.3 Hz, 1H), 6.80 (d, *J* = 8.3 Hz, 1H), 5.00 (s, 1H), 4.90 (s, 1H), 4.12 (t, *J* = 6.8 Hz, 1H), 3.83 (s, 3H), 3.36 – 3.29 (m, 1H), 2.69 (q, *J* = 8.0, 7.5 Hz, 1H), 2.60 (dt, *J* = 14.6, 7.9 Hz, 1H), 1.92 – 1.84 (m, 2H), 1.78 (s, 3H), 1.25 – 1.21 (m, 6H).

^{13}C NMR (126 MHz, $CDCl_3$) δ 155.0, 147.5, 136.9, 133.8, 126.2, 126.1, 111.2, 110.4, 75.5, 55.5, 36.9, 31.3, 26.7, 22.8, 17.6.

IR (neat) υ_{max} 3650, 2978, 2864, 2647, 1743, 1665, 1624, 1578, 1463, 1280, 1152, 836 cm^{-1}.

HRMS (ESI) *m/z*: $[M+Na]^+$ calcd. for $[C_{16}H_{24}O_2Na]^+$ 271.1674; found: 271.1664.

Attempts toward the synthesis of *E*-selective γ, δ-unsaturated ester via Johnson-Claisen Rearrangement:

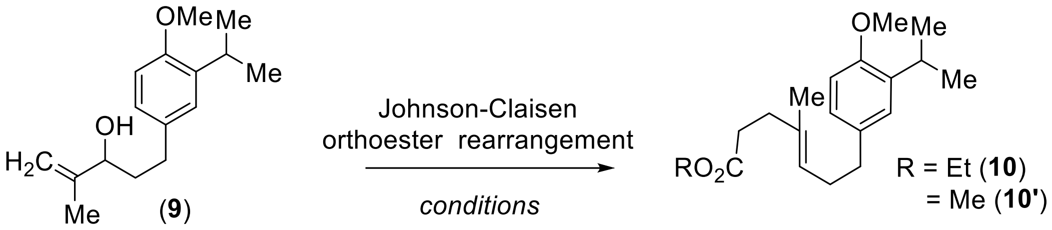

Entry	Solvent	Reagent (10 equiv.)	H^+ source (10 mol%)	Temp.(° C)	Time	Yield (%)[a,b]
1.	neat	$MeC(OMe)_3$	$C_2H_5CO_2H$	140	28 h	40
2.	neat	$MeC(OEt)_3$	*p*-NO_2-C_6H_4OH	160	28 h	44
3.	*o*-xylene	$MeC(OMe)_3$	$C_2H_5CO_2H$	180	36 h	53
4.	*o*-xylene	$MeC(OEt)_3$	$C_2H_5CO_2H$	180	36 h	59
5.	*o*-xylene	$MeC(OEt)_3$	*p*-NO_2-C_6H_4OH	180	30 h	62
6.	*p*-xylene	$MeC(OEt)_3$	*p*-NO_2-C_6H_4OH	180	30 h	66
7.	*p*-xylene	$MeC(OEt)_3$	*p*-NO_2-C_6H_4OH	microwave (180)	30 min	62
8.	*p*-xylene	$MeC(OMe)_3$	*p*-NO_2-C_6H_4OH	microwave (210)	15 min	76
9.	*p*-xylene	$MeC(OEt)_3$	*p*-NO_2-C_6H_4OH	microwave (210)	15 min	82
10.	*o*-xylene	$MeC(OEt)_3$	$C_2H_5CO_2H$	microwave (210)	15 min	73

[a]Optimization reactions were carried out on 0.40 mmol of substrate.
[b]Yields are isolated after column chromatography.

Synthesis of the *E*-selective γ, δ-unsaturated ester 10b:

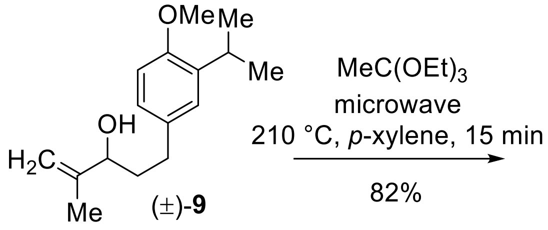

Allyl alcohol (±)-**9** (2.5 g, 10.06 mmol, 1.0 equiv.) was dissolved in minimum volume of *p*-xylene (5 mL) with triethylorthoacetate (18.45 mL, 100.66 mmol, 10.0 equiv.) in a microwave vial and *p*-nitrophenol (94 µL, 1.0 mmol, 0.1 equiv.) was added to it. The resulting solution was charged under microwave irradiation maintaining the temperature at 210 °C for 15 min. After 25 min, the reaction mixture was cooled down to 25 °C and was diluted with EtOAc (10 mL) and water (10 mL). The resulting biphasic mixture was transferred to a separatory funnel and the layers that formed were separated. The aqueous phase was further extracted with EtOAc (15 mL X 2). The combined organic layers were washed with 1 N HCl solution (30 mL), brine and dried over Na_2SO_4 and concentrated under reduced pressure. Then the crude product was purified by flash chromatography by eluting with a gradient of 5% EtOAc/*n*-hexane to afford the titled compound as pale-yellow oil (2.63 g, 82% yield).

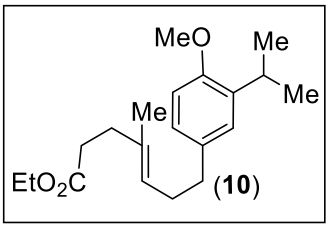

Ethyl (*E*)-7-(3-isopropyl-4-methoxyphenyl)-4-methylhept-4-enoate (**10**): **10** was obtained as pale-yellow oil (10.065 mmol, 2.628 g of product, 82% yield); R_f = 0.55 (10% EtOAc in *n*-hexane).

^{1}H NMR (500 MHz, $CDCl_3$): δ 7.04 (d, *J* = 2.3 Hz, 1H), 6.98 (dd, *J* = 8.3, 2.3 Hz, 1H), 6.79 (d, *J* = 8.2 Hz, 1H), 5.25 (ddt, *J* = 7.0, 5.6, 1.4 Hz, 1H), 4.15 (q, *J* = 7.2 Hz, 2H), 3.83 (s, 3H), 3.35 – 3.29 (m, 1H), 2.59 (dd, *J* = 8.9, 6.6 Hz, 2H), 2.44 – 2.39 (m, 2H), 2.31 (td, *J* = 9.1, 7.1 Hz, 4H), 1.59 (d, *J* = 1.3 Hz, 3H), 1.28 (t, *J* = 7.1 Hz, 3H), 1.24 (s, 3H), 1.23 (s, 3H).

^{13}C NMR (126 MHz, $CDCl_3$): δ 173.5, 154.9, 136.7, 134.1, 133.9, 126.2, 126.1, 124.7, 110.3, 60.2, 55.5, 35.3, 34.7, 33.3, 30.2, 26.7, 22.8, 15.9, 14.3.

IR (neat) υ_{max} 2996, 2914, 2836, 1743, 1671, 1568, 1455, 1233, 956, 835 cm^{-1}.

HRMS (ESI-TOF): *m/z*: $[M+Na]^+$ calcd. for $[C_{20}H_{30}O_3Na]^+$ 341.2093; found: 341.2105.

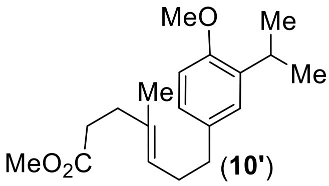

(**10'**)

Methyl (*E*)-7-(3-isopropyl-4-methoxyphenyl)-4-methylhept-4-enoate (**10'**): **10'** was obtained as pale-yellow oil; R_f = 0.55 (10% EtOAc in *n*-hexane).

^{1}H NMR (500 MHz, $CDCl_3$): δ 7.04 (s, 1H), 6.98 (d, *J* = 8.3 Hz, 1H), 6.79 (d, *J* = 8.2 Hz, 1H), 5.25 (d, *J* = 7.3 Hz, 1H), 3.83 (s, 3H), 3.69 (s, 3H), 3.32 (p, *J* = 7.1 Hz, 1H), 2.59 (t, *J* = 8.1 Hz, 2H), 2.42 (d, *J* = 7.8 Hz, 1H), 2.32 (q, *J* = 8.5 Hz, 4H), 1.59 (s, 3H), 1.23 (d, *J* = 6.9 Hz, 6H).

^{13}C NMR (126 MHz, $CDCl_3$): δ 173.9, 154.9, 136.7, 134.1, 133.8, 126.2, 126.1, 124.7, 110.3, 55.5, 51.5, 35.3, 34.6, 33.0, 30.2, 26.7, 22.8, 15.9.

IR (neat) υ_{max} 2978, 2934, 2871, 1767, 1649, 1573, 1467, 1237, 974, 856 cm^{-1}.

HRMS (ESI-TOF): *m/z*: $[M+Na]^+$ calcd. for $[C_{19}H_{28}O_3Na]^+$ 327.1936; found: 327.1924.

Reduction of γ, δ-unsaturated ester:

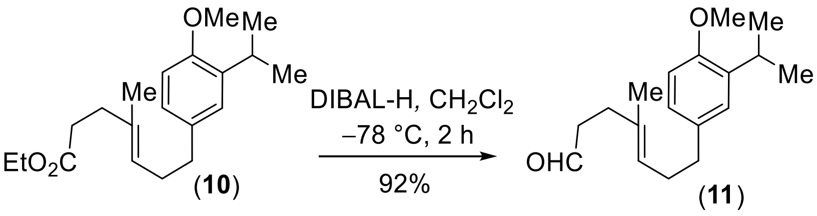

An oven-dried round-bottom flask was charged with the γ, δ-unsaturated ester (**10**) (12.0 g, 37.681 mmol, 1.0 equiv.) in dry CH_2Cl_2 (80 ml) and was cooled to –78 °C, over an acetone bath followed by the addition of a solution of DIBAL-H (1 M in hexanes, 39.50 mL, 39.565 mmol, 1.05 equiv.) in dropwise manner over 10 minutes at –78 °C. The reaction mixture was

stirred at the same temperature for an additional 2 h until the full consumption of the starting material. After complete consumption of the starting material, a saturated aqueous solution of potassium sodium tartrate (30 mL) was added slowly to the reaction mixture, and the resultant mixture was stirred vigorously for another 1 h at 25 °C. The resulting biphasic mixture was then transferred to a separating funnel and the organic layer was separated. The aqueous layer was further extracted with CH_2Cl_2 (30 mL X 2). The combined organic layers were concentrated under reduced pressure and the residue was purified by silica gel column chromatography with a gradient of 3-4% EtOAc/*n*-hexane afford pure γ, δ-unsaturated aldehyde (**11**) as pale-yellow oil (9.512 g, 92% yield).

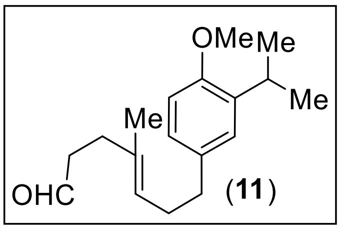

(**11**)

(*E*)-7-(3-isopropyl-4-methoxyphenyl)-4-methylhept-4-enal (**11**)**: 11** was obtained as a pale-yellow oil (37.681 mmol scale of reaction, 9.512 g of product, 92% yield); R_f = 0.45 (10% EtOAc in *n*-hexane).

^{1}H NMR (400 MHz, $CDCl_3$): δ 9.73 (t, *J* = 1.9 Hz, 1H), 6.99 (d, *J* = 2.3 Hz, 1H), 6.94 (dd, *J* = 8.2, 2.3 Hz, 1H), 6.75 (d, *J* = 8.3 Hz, 1H), 5.20 (tq, *J* = 7.0, 1.3 Hz, 1H), 3.79 (s, 3H), 3.28 (t, *J* = 6.9 Hz, 1H), 2.58 – 2.47 (m, 3H), 2.33 – 2.23 (m, 4H), 1.55 (s, 3H), 1.20 (s, 3H), 1.18 (s, 3H).

^{13}C NMR (126 MHz, $CDCl_3$): δ 202.7, 155.0, 136.7, 134.0, 133.5, 126.2, 126.1, 125.0, 110.3, 55.5, 42.2, 35.3, 31.8, 30.2, 26.7, 22.8, 22.8, 16.1.

IR (neat) υ_{max} 2980, 2895, 1785, 1660, 1455, 1233, 956, 840 cm^{-1}.

HRMS (ESI-TOF) *m/z*: $[M+Na]^+$ calcd. for $[C_{18}H_{26}O_2Na]^+$ 297.1830, found: 297.1821.

Racemic Synthesis of the propargylic alcohol:

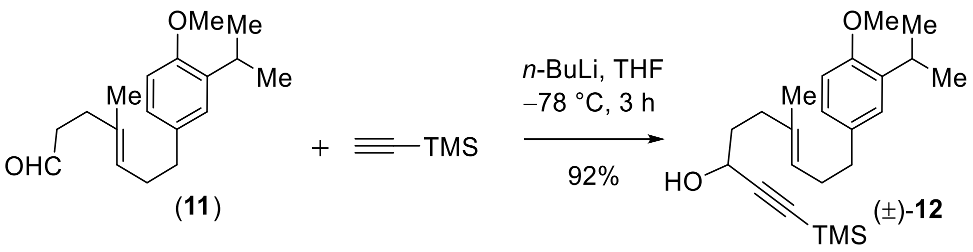

An oven-dried round-bottom flask was charged with a solution of trimethylsilyl acetylene (7.35 mL, 51.657 mmol, 1.5 equiv.) in anhydrous THF (40 mL) and was cooled to −78 °C, over an acetone bath followed by a slow addition of *n*-BuLi (2.5 M in *n*- hexane, 17.90 mL, 44.769 mmol, 1.3 equiv.) The resulting reaction mixture was allowed to stir at the same temperature for 30 minutes followed by a slow addition of the γ,δ-unsaturated aldehyde (9.45 g, 34.438 mmol, 1.0 equiv.) dissolved in anhydrous THF (30 mL) over a period of 10 minutes. The reaction mixture was then allowed to stir at 25 °C for an additional 2 h until the full consumption of the starting material. After completion of the reaction (as judged by running TLC), was quenched with saturated NH_4Cl solution (25 mL). The organic layer from the biphasic solution was separated by a separatory funnel and the aqueous phase was further extracted with EtOAc (20 mL X 2). The combined organic layers were washed with brine, dried over Na_2SO_4, and concentrated in vacuo under reduced pressure. Finally, the crude product was purified through flash column chromatography using EtOAc in *n*-hexane (10% EtOAc in *n*-hexane) to afford the racemic propargyl alcohol (±)-**12** as colorless oil (11.80 g; 92% yield).

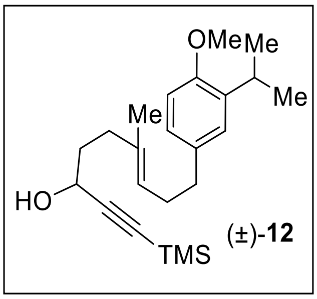

(*E*)-9-(3-isopropyl-4-methoxyphenyl)-6-methyl-1-(trimethylsilyl)non-6-en-1-yn-3-ol [(±)-**12**]**:** The racemic propargyl alcohol was obtained as a colourless oil (34.438 mmol scale, 11.8 g of product, 92% yield); R_f = 0.4 (10% EtOAc in *n*-hexane).

DMP oxidation of the propargyl alcohol:

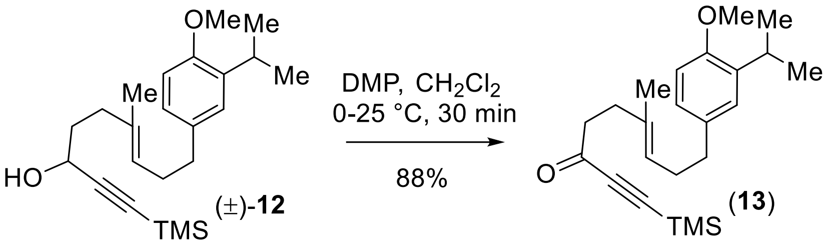

In an oven-dried round-bottom flask, the propargyl alcohol (±)-**12** (11.72 g, 31.452 mmol, 1.0 equiv.) in DCM (90 mL) was charged with Dess-Martin Periodinane (14.674 g, 34.597 mmol, 1.1 equiv.) at 0 ºC, over a period of 10 minutes with continuous stirring over an ice bath. After the complete addition of DMP, the ice bath was removed, and the reaction mixture was allowed to stir for additional 30 minutes until the full consumption of the starting material (monitored by TLC analysis). After completion of the reaction, was quenched with saturated $NaHCO_3$ (30 mL) at 0 ºC. The biphasic mixture was then transferred to a separatory funnel and the organic part was collected. The aqueous part was further extracted with CH_2Cl_2 (30 mL X 3). The combined organic layers were washed with brine, dried over Na_2SO_4 and concentrated in vacuo under reduced pressure. Finally, the crude product was purified through flash column chromatography using 5% EtOAc in *n*-hexane to afford the titled carbonyl product **13** as colorless oil (10.257 g, 88% yield).

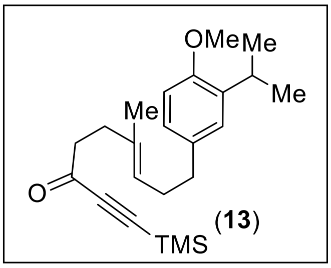

(*E*)-**9-(3-isopropyl-4-methoxyphenyl)-6-methyl-1-(trimethylsilyl)non-6-en-1-yn-3-one (13): 13** was obtained as a colurless oil (33.70 mmol, 10.257 g of product, 88% yield); R_f = 0.5 (10% EtOAc in *n*-hexane).

^{1}H NMR (400 MHz, $CDCl_3$): δ 7.00 (d, *J* = 2.3 Hz, 1H), 6.94 (dd, *J* = 8.2, 2.3 Hz, 1H), 6.75 (d, *J* = 8.2 Hz, 1H), 5.21 (tq, *J* = 7.1, 1.3 Hz, 1H), 3.79 (s, 3H), 3.32 – 3.25 (m, 1H), 2.66 – 2.62 (m, 2H), 2.56 (dd, *J* = 8.9, 6.7 Hz, 2H), 2.30 (dt, *J* = 23.4, 7.6 Hz, 5H), 1.55 (d, *J* = 1.4 Hz, 3H), 1.22 (d, *J* = 6.9 Hz, 1H), 1.20 (s, 3H), 1.19 (s, 3H), 0.24 (s, 9H).

^{13}C NMR (101 MHz, $CDCl_3$): δ 187.7, 155.0, 136.8, 134.2, 133.5, 126.3, 126.2, 125.1, 110.4, 102.1, 98.0, 55.6, 44.1, 35.4, 33.7, 30.3, 26.8, 22.8, 16.1, –0.7.

IR (neat) υ_{max} 2958, 2835, 2170, 1770, 1660, 1587, 1495, 1455, 1233, 1112, 1052, 840, 752 cm^{-1}.

HRMS (ESI-TOF) *m/z*: $[M+Na]^+$ calcd. for $[C_{23}H_{34}O_2SiNa]^+$ 393.2226, found: 393.2216.

Attempts toward the Asymmetric transfer hydrogenation with Noyori's protocol:

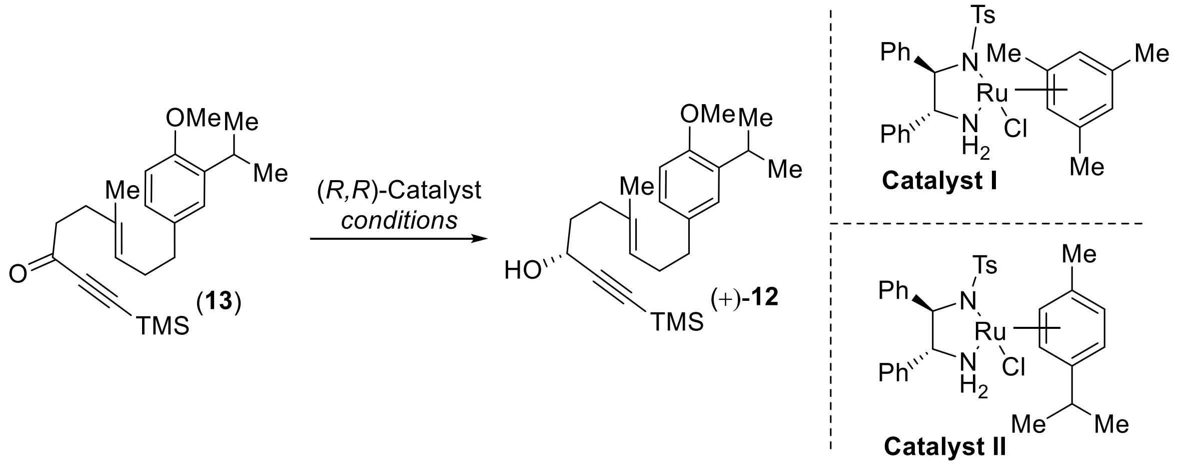

Entry	Reagents	Catalyst	Time	ee(%)	Yield(%)[a,b]
1.	^{i}PrOH	Cat-I (2 mol%)	18 h	n.r.	n.r.
2.	^{i}PrOH	Cat-II (2 mol%)	18 h	n.r.	n.r.
3.	HCOOH-Et_3N	Cat-I (2 mol%)	18 h	96	43 (81%, BRSM)
4.	HCOOH-Et_3N	Cat-II (2 mol%)	18 h	96	54 (76%, BRSM)
5.	HCOOH-Et_3N, ^{i}PrOH	Cat-I (2 mol%)	8 h	97	78
6.	HCOOH-Et_3N, THF	Cat-I (2 mol%)	8 h	98	88
7.	HCOOH-Et_3N, ^{i}PrOH	Cat-II (2 mol%)	6 h	98	83
8.	HCOOH-Et_3N, THF	Cat-II (2 mol%)	6 h	99	92
9.	HCOOH-Et_3N, THF	Cat-II (1 mol%)	6 h	99	92
10.	HCOOH-Et_3N, THF	Cat-I (1 mol%)	6 h	97	87

[a]Optimization reactions were carried out on 0.40 mmol of substrate.
[b]Yields are isolated after column chromatography.
All reactions were carried out in 3 mL of solvent at 25 °c with Et_3N(4 equiv.), HCO_2H (10 equiv.)
n.r.- no reaction

General procedure for Noyori's Asymmetric Hydrogenation:

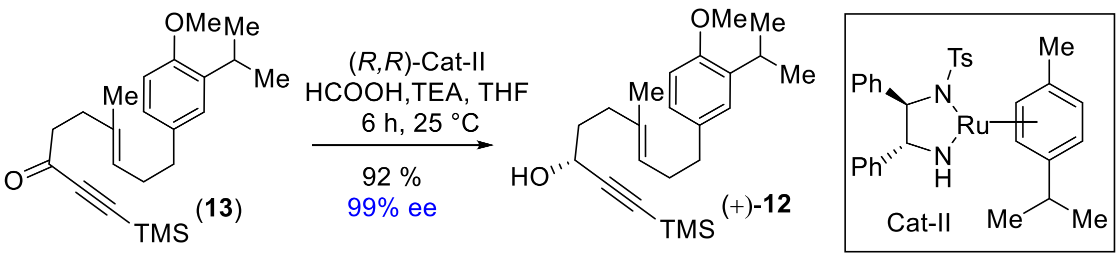

In a three-neck 250 mL round-bottomed flask with a N_2 inlet adapter, rubber septa, and magnetic stirrer bar were charged with ketone **(13)** (10.16 g, 27.414 mmol, 1.0 equiv.) in anhydrous THF (80 mL). Then Et_3N (15.28 mL, 109.657 mmol, 4.0 equiv.), HCOOH (10.34 mL, 274.14 mmol, 10.0 equiv.) and the Noyori's Ru-catalyst, (*R*, *R*)-**Cat-II** (164 mg, 0.274 mmol, 0.01 equiv.) were added sequentially. The reaction mixture was stirred at 25 ºC for an

additional 6 h until the completion of the reaction. Next the reaction mixture was diluted with ethyl acetate (30 mL) and water (30 mL). The resulting biphasic mixture was transferred to a separatory funnel and the layers that formed were separated. The aqueous phase was further extracted with EtOAc (25 mL X 2). The combined organic layers were washed with excess water, brine and dried over Na_2SO_4 and concentrated under reduced pressure. Then the crude product was purified by flash chromatography by eluting with a gradient of 5% EtOAc/*n*-hexane to afford (+)-**12** as colorless oil (9.397 g, 92% yield).

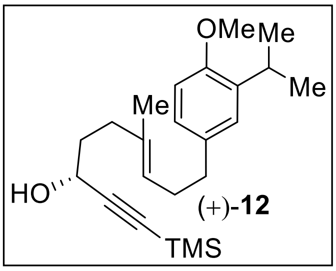

(*R*,*E*)-**9-(3-isopropyl-4-methoxyphenyl)-6-methyl-1-(trimethylsilyl)non-6-en-1-yn-3-ol** [(+)-**12**]**:** (+)-**12** was obtained as a colourless oil (27.414 mmol, 9.397 g of product, 92% yield); R_f = 0.4 (10% EtOAc in *n*-hexane).

^{1}H NMR (400 MHz, $CDCl_3$): δ 7.00 (d, *J* = 2.3 Hz, 1H), 6.95 (dd, *J* = 8.2, 2.3 Hz, 1H), 6.75 (d, *J* = 8.2 Hz, 1H), 5.26 – 5.20 (m, 1H), 4.27 (td, *J* = 6.5, 5.7 Hz, 1H), 3.79 (s, 3H), 3.32 – 3.23 (m, 1H), 2.57 (dd, *J* = 8.8, 6.7 Hz, 2H), 2.27 (q, *J* = 7.5 Hz, 2H), 2.13 (t, *J* = 7.6 Hz, 2H), 1.80 – 1.73 (m, 3H), 1.55 (s, 3H), 1.19 (d, *J* = 7.0 Hz, 6H), 0.17 (s, 9H).

^{13}C NMR (126 MHz, $CDCl_3$): δ 154.9, 136.7, 134.6, 134.2, 126.12, 124.9, 110.3, 106.8, 89.5, 62.6, 55.5, 35.9, 35.4, 35.2, 30.2, 26.7, 22.8, 15.9, −0.1.

IR (neat) υ_{max} 3360, 2980, 2859, 2835, 2170, 1660, 1587, 1455, 1233, 1052, 956, 840, 752 $cm^{-1.}$

HRMS (ESI-TOF) *m/z*: $[M+Na]^+$ calcd. for $[C_{23}H_{36}O_2SiNa]^+$ 395.2382, found: 395.2361.

$[\alpha]^{20}_{589}$ = +4.0 (*c* = 1.0, $CHCl_3$).

N.B:- Because of the poor UV-activity of the obtained propargyl alcohol, both the racemic and the chiral propargyl alcohol were converted into the corresponding *p*-nitro benzoate derivative and the % of ee was measured.

Preparation of the propargyl benzoate derivative:

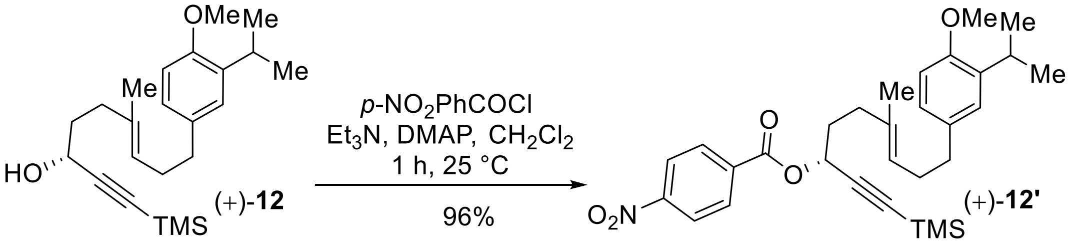

In an oven-dried round-bottom flask ketal protected propargyl alcohol (+)-**12** (50 mg, 0.134 mmol, 1.0 equiv.) was taken in dry CH_2Cl_2 (3 mL). To the solution were added Et_3N (56 µL, 0.402 mmol., 3 equiv.), DMAP (2 mg, 0.013 mmol., 0.1 equiv.) and *p*-nitro benzoyl chloride (28 mg, 0.161 mmol., 1.2 equiv.) sequentially. The resulting reaction mixture was allowed to stir at 25 °C until the full consumption of starting material (1 h). Upon completion of the reaction (as monitored by TLC analysis), the reaction mixture was diluted with water (3 mL). The resulting biphasic mixture was transferred to a separatory funnel and the layers that formed were separated. The aqueous phase was further extracted CH_2Cl_2 (5 mL X 2). The organic layers were combined, dried over Na_2SO_4 and concentrated on a rotary evaporator under reduced pressure. The crude acetate derivative was purified by flash column chromatography with 2% EtOAc in *n*-hexane to afford the propargyl benzoate derivative (+)-**12'** as yellow oil (67 mg, 96% yield).

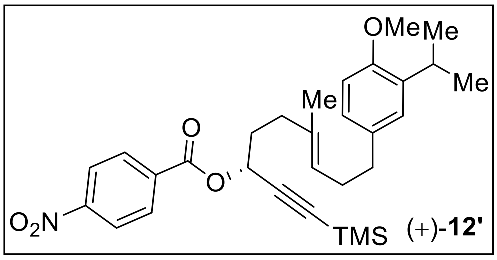

(*R*,*E*)-**9-(3-isopropyl-4-methoxyphenyl)-6-methyl-1-(trimethylsilyl)non-6-en-1-yn-3-yl 4-nitrobenzoate** [(+)-**12'**]: (+)-**12'** was obtained as a yellow oil (0.134 mmol, 67 mg of product, 96% yield, 99% ee); R_f = 0.8 (10% EtOAc in *n*-hexane).

^{1}H NMR (400 MHz, $CDCl_3$): δ 8.30 – 8.27 (m, 2H), 8.24 – 8.21 (m, 2H), 6.99 (d, *J* = 2.3 Hz, 1H), 6.94 (dd, *J* = 8.2, 2.3 Hz, 1H), 6.74 (d, *J* = 8.2 Hz, 1H), 5.58 (t, *J* = 6.7 Hz, 1H), 5.21 (td, *J* = 7.1, 1.3 Hz, 1H), 3.78 (s, 3H), 3.27 (p, *J* = 6.9 Hz, 1H), 2.58 – 2.52 (m, 2H), 2.25 (q, *J* =

7.5 Hz, 2H), 2.17 (d, J = 7.6 Hz, 2H), 2.06 – 1.94 (m, 2H), 1.58 – 1.56 (m, 3H), 1.19 (s, 3H), 1.17 (s, 3H), 0.17 (s, 9H).

^{13}C NMR (101 MHz, $CDCl_3$): δ 163.7, 155.0, 150.7, 136.8, 135.5, 134.1, 133.7, 131.0, 126.3, 126.2, 125.3, 123.6, 110.4, 101.9, 91.6, 77.3, 65.8, 55.6, 53.5, 35.4, 35.1, 33.3, 30.3, 29.8, 26.8, 22.8, 16.0, –0.14.

IR (neat) υ_{max} 3360, 2980, 2859, 2835, 2170, 1735, 1660, 1587, 1550, 1455, 1250, 1233, 1052, 956, 840, 752 cm^{-1}.

HRMS (ESI-TOF) *m/z*: $[M+H]^+$ calcd. for $[C_{30}H_{40}NO_5Si]^+$ 522.2676, found:522.2681.

$[\alpha]^{20}_{589}$ = +8.0 (c = 0.22, $CHCl_3$).

Enantiomeric excess of pure compound was determined *via* HPLC analysis using a Chiralpak AD-H column; solvent: *n*-hexane/2-propanol = 99/1; flow rate: 0.4 mL/min; detection at 254 nm): t_R minor= 12.090 min, t_R major = 13.185 min. $[\alpha]^{20}_{589}$ = –8.0 (c = 0.17, $CHCl_3$ for 99% ee).

Preparation of the alkyne-ene cyclization precursor:

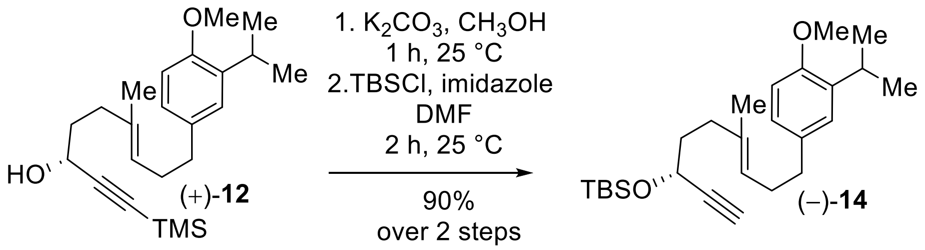

In an oven dried round-bottom flask, the TMS-protected propargyl alcohol (+)-**12** (9.18 g, 24.636 mmol, 1.0 equiv.) was taken in a mixed solvent system of methanol (60 mL). To this solution was added K_2CO_3 (4.085 g, 25.563 mmol, 1.2 equiv.) at 25 ºC and stirring was continued for an additional 1 h. After completion of the reaction (judged by TLC analysis), the solvent of the reaction mixture was evaporated to dryness and further diluted with ethyl acetate (30 mL) and water (20 mL). The resulting biphasic mixture was transferred to a separatory funnel and the layers that formed were separated. The aqueous phase was further extracted with ethyl acetate (20 mL X 2). The combined organic layers were dried over anhydrous Na_2SO_4

and concentrated in a rotary evaporator under reduced pressure. Next, the crude yellow oil was charged for the next step without further purification. [R_f = 0.30 (10% EtOAc in *n*-hexane)]

The crude product was dissolved in anhydrous DMF (60 ml). Imidazole (3.354 g, 49.272 mmol, 2.0 equiv.), TBSCl (4.455 g, 29.563 mmol, 1.2 equiv.) were sequentially added to the reaction vessel at 25 ºC and stirred for 2 h. After completion of the reaction, water (30 mL) was added, and the resulting mixture was extracted twice with ethyl acetate (30 X 2 mL). The combined organic extracts were washed with brine and excess water, dried over Na_2SO_4, filtered, and concentrated to give a crude product which was purified by flash chromatography on silica gel with 5% EtOAc in *n*-hexane to afford the TBS protected propargyl alcohol derivative (–)-**14** as colorless liquid (9.195 g, 90% yield over 2 steps).

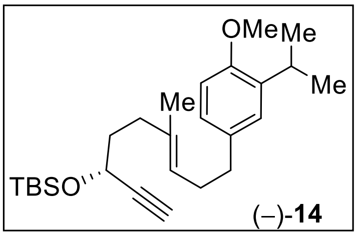

(–)-**14**

(*R*,*E*)-tert-butyl((9-(3-isopropyl-4-methoxyphenyl)-6-methylnon-6-en-1-yn-3-yl)oxy)dimethylsilane [(–)-**14**]: (–)-**14** was obtained as a colorless liquid (24.636 mmol, 9.195 g of product, 90% yield over 2 steps); R_f = 0.7 (10% EtOAc in *n*-hexane).

^{1}H NMR (400 MHz, $CDCl_3$): δ 7.01 (d, *J* = 2.3 Hz, 1H), 6.95 (dd, *J* = 8.2, 2.3 Hz, 1H), 6.75 (d, *J* = 8.2 Hz, 1H), 5.24 – 5.19 (m, 1H), 4.31 (td, *J* = 6.5, 2.1 Hz, 1H), 3.79 (s, 4H), 3.32 – 3.25 (m, 1H), 2.58 – 2.54 (m, 2H), 2.38 (d, *J* = 2.2 Hz, 1H), 2.30 – 2.24 (m, 2H), 2.09 (q, *J* = 7.0 Hz, 2H), 1.79 – 1.73 (m, 2H), 1.55 (s, 3H), 1.20 (s, 3H), 1.18 (s, 3H), 0.90 (s, 9H), 0.13 (s, 3H), 0.10 (s, 3H).

^{13}C NMR (101 MHz, $CDCl_3$): δ 155.0, 136.8, 134.8, 134.3, 126.3, 126.2, 124.4, 110.4, 85.7, 72.2, 62.5, 55.6, 37.1, 35.5, 35.2, 30.4, 26.8, 25.9, 25.9, 25.9, 25.9, 22.9, 18.3, 16.2, –4.4, –5.0.

IR (neat) $υ_{max}$ 3360, 2952, 2856, 1600, 1587, 1495, 1456, 1243, 1052, 956, 840, 752 cm^{-1}.

HRMS (ESI-TOF) *m/z*: $[M+Na]^+$ calcd. for $[C_{26}H_{42}O_2SiNa]^+$ 437.2852, found: 437.2849.

$[\alpha]^{20}_{589}$ = – 4.0 (*c* = 0.35, $CHCl_3$).

Attempts toward the In(III)-catalyzed alkyne-ene cyclization:

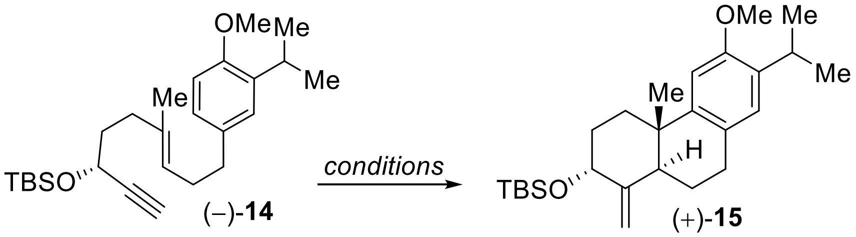

Entry	Catalyst (mol %)	Solvent	Temp.(° C)	Time	Yield (%)[a,b]
1.	$In(OTf)_3$ (10 mol%)	DCE	25	2 h	28
2.	$In(OTf)_3$ (10 mol%)	DCE	0	6 h	33
3.	$InBr_3$ (20 mol%)	DCM	0	3 h	61
4.	$In(OTf)_3$ (10 mol%)	DCM	–20	10 h	54
5.	$InCl_3$ (10 mol%)	DCM	–20	14 h	59
6.	$InCl_3$ (20 mol%)	DCM	–20	8 h	65
7.	$InBr_3$ (20 mol%)	DCM	–20	4 h	74
8.	$InBr_3$ (20 mol%)	DCE	–20	5 h	67
9.	InI_3 (20 mol%)	DCM	–20	4 h	72
10.	$InBr_3$ (10 mol%)	DCM	–30	8 h	72
11.	$InCl_3$ (10 mol%)	DCE	–40	24 h	53

[a]Optimization reactions were carried out on 0.40 mmol of substrate.
[b]Yields are isolated after column chromatography.

In(III)-catalyzed alkyne-ene cyclization:

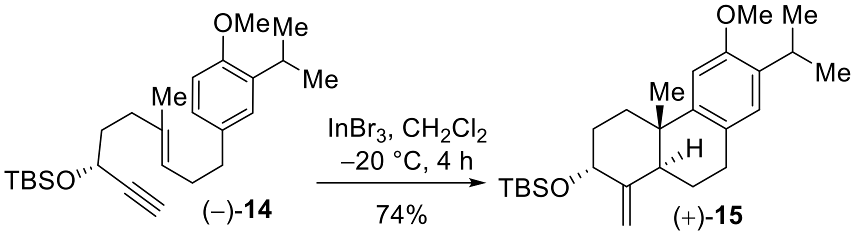

In an oven dried round-bottom flask, TBS- protected propargyl alcohol (–)-**14** (8.58 g, 20.689 mmol, 1.0 equiv.) was taken in anhydrous dichloromethane (80 mL) and was cooled to –20 ºC. To this solution was added solid $InBr_3$ (1.467 g, 4.137 mmol, 0.2 equiv.) at once and stirring was continued for an additional 4 h at –20 ºC. Upon completion of the reaction (as monitored by TLC analysis), the reaction mixture was quenched with saturated $NaHCO_3$ (20 mL). The resulting biphasic mixture was then transferred to a separatory funnel and the organic part was collected. The aqueous part was further extracted with CH_2Cl_2 (20 mL X 2). The combined organic layers were washed with brine, dried over Na_2SO_4 and concentrated in vacuo under reduced pressure. Finally, the crude product was purified through flash column chromatography using *n*-hexane to afford the TBS-protected tricyclic allyl alcohol (6.349 g, 74% yield).

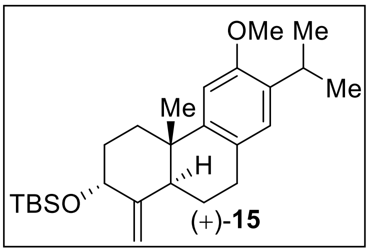

1,2,3,4,4a,9,10,10a-octahydrophenanthren-2-yl)oxy)dimethylsilane [(+)-**15**]: (+)-**15** was obtained as a colourless liquid (20.689 mmol, 6.349 g, 74% yield); R_f = 0.4 (in *n*-hexane).

^{1}H NMR (500 MHz, $CDCl_3$): δ 6.92 (s, 1H), 6.79 (s, 1H), 4.96 (t, *J* = 1.5 Hz, 1H), 4.66 (t, *J* = 1.7 Hz, 1H), 4.30 (d, *J* = 2.9 Hz, 1H), 3.83 (s, 3H), 3.29 – 3.23 (m, 1H), 2.90 – 2.85 (m, 2H), 2.81 – 2.77 (m, 1H), 2.06 (dt, *J* = 12.4, 6.1 Hz, 1H), 2.00 (s, 1H), 1.89 – 1.83 (m, 2H), 1.78 – 1.73 (m, 2H), 1.24 (s, 3H), 1.22 (s, 3H), 1.00 (s, 3H), 0.86 (s, 9H), 0.06 (s, 3H), 0.01 (s, 3H).

^{13}C NMR (101 MHz, $CDCl_3$): δ 155.1, 152.7, 145.3, 134.4, 126.9, 126.7, 108.1, 107.5, 73.5, 55.6, 41.6, 39.4, 32.6, 31.9, 29.4, 26.6, 25.9, 22.9, 22.8, 22.3, 21.1, 18.2, -4.6, -5.0.

IR (neat) ν_{max} 3084, 2967, 2872, 2831, 1643, 1621, 1562, 1433, 1254, 986 cm^{-1}.
HRMS (ESI) m/z: $[M + Na]^+$ calcd for $[C_{26}H_{42}O_2SiNa]^+$: 437.2852, found: 437.2873

$[\alpha]^{20}_{589}$ = +62.8 (*c* = 0.22, $CHCl_3$).

Desilylation of TBS-protected allyl alcohol:

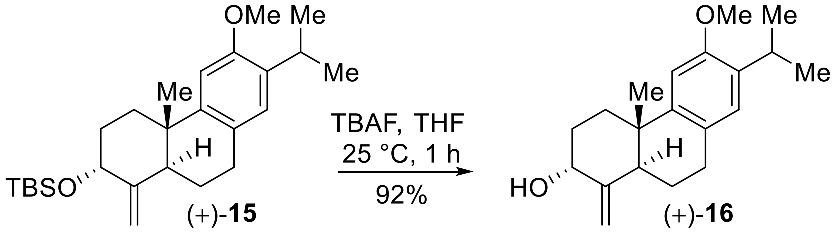

A solution of tetrabutylammonium fluoride in tetrahydrofuran (1.0 M, 16.7 mL, 16.71 mmol, 1.1 equiv.) was added to a solution of the silyloxy olefin (+)-**15** (6.3 g, 15.191 mmol, 1.0 equiv.) in tetrahydrofuran (40 mL) at 25 ºC. The reaction mixture was stirred for 1 h at 25 ºC until the full consumption of the starting material (monitored by TLC). The reaction mixture was diluted sequentially with saturated aqueous ammonium chloride solution (15 mL), and ethyl acetate (15 mL). The resulting biphasic mixture was transferred to a separatory funnel and the layers that formed were separated. The organic layer was washed with brine, dried over Na_2SO_4 and concentrated under reduced pressure. Then the crude product was purified by flash column chromatography by eluting with a gradient of 8-12% EtOAc/*n*-hexane to afford allyl alcohol (+)-**16** as colorless foam (4.2 g, 92% yield).

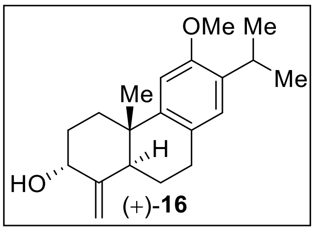

(2*R*,4a*S*,10a*R*)-7-isopropyl-6-methoxy-4a-methyl-1-methylene-1,2,3,4,4a,9,10,10a-octahydrophenanthren-2-ol [(+)-**16**]: (+)-**16** was obtained as colorless foam (15.191 mmol; 4.2 g, 92% yield). R_f = 0.25 (10% EtOAc in *n*-hexane).

^{1}H NMR (400 MHz, $CDCl_3$): δ 6.88 (d, *J* = 0.8 Hz, 1H), 6.74 (s, 1H), 5.07 (t, *J* = 1.5 Hz, 1H), 4.74 (t, *J* = 1.7 Hz, 1H), 4.36 (s, 1H), 3.79 (s, 3H), 3.26 – 3.19 (m, 1H), 2.88 – 2.83 (m, 2H), 2.75 (ddd, *J* = 13.1, 3.3, 1.7 Hz, 1H), 2.04 – 1.99 (m, 2H), 1.93 (td, *J* = 5.8, 3.1 Hz, 2H), 1.82 – 1.74 (m, 2H), 1.19 (d, *J* = 5.5 Hz, 3H), 1.18 (d, *J* = 5.4 Hz, 3H), 0.99 (s, 3H).

^{13}C NMR (126 MHz, $CDCl_3$): δ 155.0, 151.8, 144.6, 134.6, 126.7, 126.6, 110.0, 107.4, 72.9, 55.6, 41.6, 39.3, 32.5, 30.2, 29.1, 26.5, 22.9, 22.7, 21.9, 21.0.

IR (neat) ν_{max} 3641, 2958, 1749, 1622, 1456, 1254, 986 cm^{-1}.
HRMS (ESI) m/z: $[M + Na]^+$ calcd for $[C_{20}H_{28}ONa]^+$: 323.1968, found: 323.1982

$[\alpha]^{20}_{589}$ = +125.40 (*c* = 0.26, $CHCl_3$).

Oxidation of the allyl alcohol (+)-**17:**

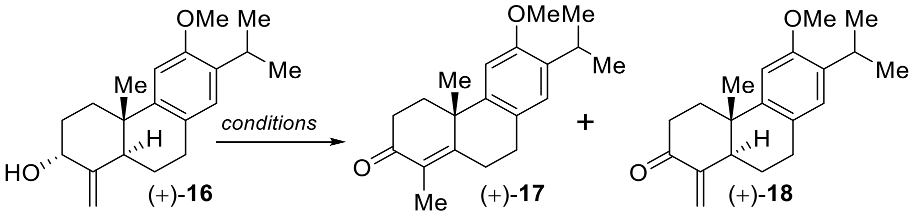

Entry	Oxidant	Solvent	Temp.	Time	Yield(%)[a,b]	Product Ratio (+)-17:(+)-18
1.	PCC	CH_2Cl_2	25 °C	2 h	65	1 : 1.5
2.	DMP	CH_2Cl_2	25 °C	2 h	72	1 : 1.8
3.	MnO_2	CH_2Cl_2	25 °C	24 h	-	SM
4.	DMSO $(COCl)_2$, Et_3N	CH_2Cl_2	–78 °C-25 °C	2 h	76	Only 18 formed

[a]Optimization reactions were carried out on 0.33 mmol of substrate.
[b]Yields are isolated after column chromatography.

Swern oxidation of the allyl alcohol (+)**-16:**

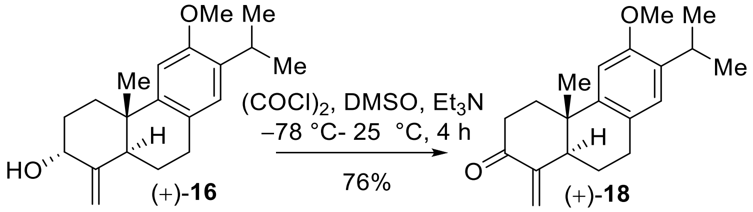

A flame-dried flask was charged with DMSO (4.73 mL, 66.568 mmol, 2.0 equiv.) in anhy. CH_2Cl_2 (60 mL) and cooled to −78 °C, over an acetone bath. Freshly distilled oxalyl chloride (3.38 mL, 39.941 mmol, 1.2 equiv.) was added dropwise manner over 10 minutes followed by slow addition of a solution of allyl alcohol (+)**-16** (10.0 g, 33.284 mmol, 1.0 equiv.), in 40 mL of anhy. CH_2Cl_2. The reaction was stirred at −78 °C for another 1 h, followed by slow addition of triethylamine (15.8 mL, 113.165 mmol, 5 equiv.). Then the reaction mixture was allowed to warm to 25 °C, over a time of 4 h. After completion of the reaction (as judged by running TLC), the reaction mixture was then poured over ice-cold 1 M HCl (30 mL). The organic layer from the biphasic mixture was separated using a separatory funnel. The aqueous phase was further extracted with CH_2Cl_2 (30 mL X 2). The combined organic layers were washed with brine, dried over Na_2SO_4 and concentrated under reduced pressure to afford the crude allylic alcohol, which was further purified by column chromatography with a gradient of 5-8% EtOAc/*n*-hexane afford pure enone (+)**-18** as colorless oil (7.549 g, 76% yield).

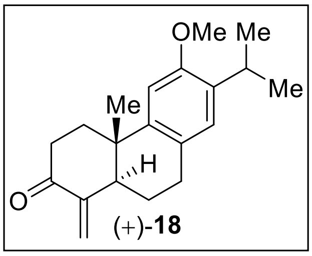

(4a*S*,10a*R*)**-7-isopropyl-6-methoxy-4a-methyl-1-methylene-3,4,4a,9,10,10a-hexahydrophenanthren-2(1H)-one** [(+)**-18**]: (+)**-18** was obtained as colourless oil (33.284 mmol; 7.549 g; 76% yield). R_f = 0.35 (10% EtOAc in *n*-hexane).

^{1}H NMR (500 MHz, $CDCl_3$): δ 6.91 (s, 1H), 6.77 (s, 1H), 6.00 – 5.96 (m, 1H), 5.22 (dd, *J* = 2.2, 1.2 Hz, 1H), 3.80 (s, 3H), 3.29 – 3.20 (m, 1H), 2.91 – 2.84 (m, 2H), 2.74 – 2.63 (m, 2H),

2.63 – 2.57 (m, 1H), 2.48 (ddd, *J* = 13.3, 7.2, 2.6 Hz, 1H), 2.06 – 1.94 (m, 2H), 1.78 – 1.66 (m, 1H), 1.20 (d, *J* = 5.5 Hz, 3H), 1.19 (d, *J* = 5.4 Hz, 3H), 1.15 (s, 3H).

^{13}C NMR (126 MHz, $CDCl_3$): δ 202.1, 155.3, 148.5, 143.0, 135.3, 126.7, 126.5, 118.5, 107.7, 55.6, 46.4, 37.4, 36.7, 36.2, 29.1, 26.5, 22.8, 22.6, 22.4, 21.0.

IR (neat) ν_{max} 3143, 3082, 1692, 1598, 1622, 1456, 1254, 986 cm^{-1}.
HRMS (ESI) m/z: $[M + H]^+$ calcd for $[C_{20}H_{27}O_2]^+$: 299.2006, found: 299.2010

$[\alpha]^{20}_{589}$ = +156.30 (*c* = 0.4, $CHCl_3$).

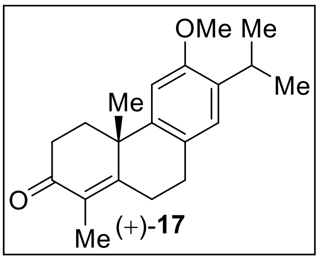

(*S*)-7-isopropyl-6-methoxy-1,4a-dimethyl-4,4a,9,10-tetrahydrophenanthren-2(3H)-one [(+)-**17**]: (+)-**17** was obtained as colourless oil. R_f = 0.32 (10% EtOAc in *n*-hexane).

^{1}H NMR (500 MHz, $CDCl_3$): δ 6.94 (s, 1H), 6.76 (s, 1H), 3.84 (s, 3H), 3.30 – 3.26 (m, 1H), 2.96 (dt, *J* = 15.1, 4.9 Hz, 1H), 2.92 – 2.88 (m, 1H), 2.80 – 2.72 (m, 2H), 2.60 – 2.51 (m, 2H), 2.39 (ddd, *J* = 13.2, 5.2, 2.5 Hz, 1H), 2.13 – 2.07 (m, 1H), 1.87 (d, *J* = 1.2 Hz, 3H), 1.56 (s, 3H), 1.24 (d, *J* = 6.9 Hz, 3H), 1.22 (d, *J* = 6.9 Hz, 3H).

^{13}C NMR (126 MHz, $CDCl_3$): δ 198.3, 162.7, 155.8, 142.6, 135.1, 128.5, 127.4, 125.9, 107.5, 55.6, 39.8, 36.2, 34.2, 29.4, 27.6, 26.9, 26.5, 22.7, 10.9.

IR (neat) ν_{max} 2994, 2951, 1733, 1625, 1463, 1278, 981 cm^{-1}.
HRMS (ESI) m/z: $[M + Na]^+$ calcd for $[C_{20}H_{26}O_2Na]^+$: 321.1825, found: 321.1796

$[\alpha]^{20}_{589}$ = +191.55 (*c* = 0.25, $CHCl_3$).

Wittig olefination of enone (+)**- 18:**

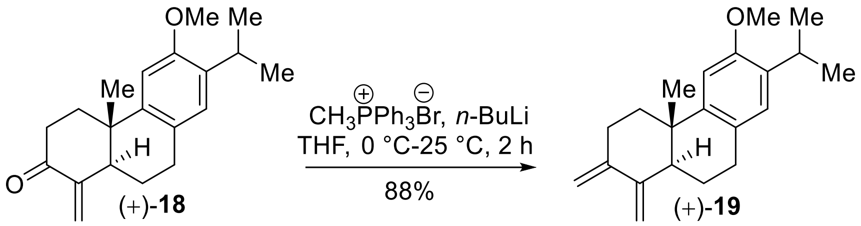

An oven-dried round-bottom flask was charged with $MePPh_3Br$ (2.0 g, 5.629 mmol, 2.1 equiv.) in THF (20 mL) at 0 °C, over an ice-water bath. *n*-BuLi (2.5 M in hexanes, 2.14 mL, 5.361 mmol, 2.0 equiv.) was added, and the ice bath was removed. The mixture was stirred for 30 minutes at 25 °C and was cooled again to 0 °C followed by a slow addition of a solution of enone (+)**-18** (800 mg, 2.680 mmol, 1 equiv.) dissolved in THF (10 mL) to the reaction mixture. Then the reaction mixture was allowed to warm to 25 °C with continuous stirring for 2 h. After completion of the reaction (as judged by running TLC), was quenched with saturated NH_4Cl solution (10 mL). The organic layer from the biphasic solution was separated by a separatory funnel and the aqueous phase was further extracted with EtOAc (10 mL X 2). The combined organic layers were washed with brine (5 mL), dried over Na_2SO_4, and concentrated in vacuo under the reduced pressure. Finally, the crude product was purified through column chromatography using EtOAc in *n*-hexane (1% EtOAc in *n*-hexane) to afford the olefin (+)**-19** as colorless gel (699 mg; 88% yield).

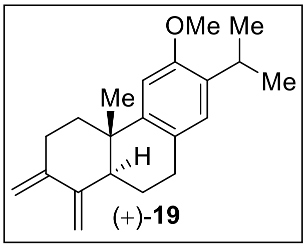

(4a*S*,10a*S***)-7-isopropyl-6-methoxy-4a-methyl-1,2-dimethylene-1,2,3,4,4a,9,10,10a-octahydrophenanthrene** [(+)**-19]:** (+)**-19** was obtained as colorless gel (2.68 mmol; 699 mg; 88% yield). R_f = 0.6 (2.5% EtOAc in *n*-hexane).

^{1}H NMR (400 MHz, $CDCl_3$): δ 6.92 (s, 1H), 6.80 (s, 1H), 5.16 (d, *J* = 2.0 Hz, 1H), 4.97 (q, *J* = 2.1 Hz, 1H), 4.71 (dd, *J* = 6.7, 2.2 Hz, 2H), 3.84 (s, 3H), 3.27 (p, *J* = 6.9 Hz, 1H), 2.93 – 2.83 (m, 2H), 2.63 – 2.53 (m, 1H), 2.48 (dd, *J* = 14.7, 4.9 Hz, 1H), 2.39 – 2.30 (m, 2H), 1.95

(dq, *J* = 11.7, 3.3, 2.8 Hz, 1H), 1.74 (qd, *J* = 13.4, 4.6 Hz, 2H), 1.23 (t, *J* = 5.8 Hz, 6H), 1.12 (d, *J* = 1.5 Hz, 3H).

^{13}C NMR (101 MHz, $CDCl_3$): δ 155.1, 151.6, 149.8, 144.4, 134.7, 126.8, 126.7, 126.6, 107.8, 107.6, 107.6, 107.3, 107.3, 55.6, 55.5, 47.8, 38.5, 30.9, 29.3, 26.5, 22.8, 22.7, 22.3, 22.2, 21.3.

IR (neat) ν_{max} 3012, 2972, 2954, 1692, 1658, 1469, 1274 cm^{-1}.
HRMS (ESI) m/z: $[M + H]^+$ calcd for $[C_{21}H_{29}O]^+$: 297.2213, found: 297.2187

$[\alpha]^{20}_{589}$ = +72.45 (*c* = 0.3, $CHCl_3$).

Attempts toward the allylic oxidation of diene (+)-19:

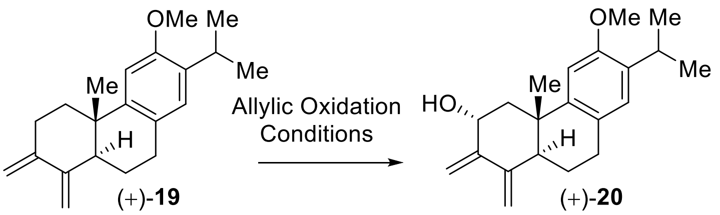

Entry	Reaction Condition	Solvent	Temp.	Time	Yield(%)[a,b]
1.	CrO_3<br>3,5-dimethyl pyrazole	CH_2Cl_2	–15 °C- 25 °C	1 h	decomposition
2.	CrO_3	CH_2Cl_2	25 °C	1 h	complex mixture
3.	PDC	PhH	80 °C	12 h	decomposition
4.	SeO_2	dioxane:H_2O	100 °C	40 min	complex mixture
5.	SeO_2-TBHP(aq.)	CH_2Cl_2	25 °C	4 h	complex mixture
6.	SeO_2	dioxane:HCOOH	100 °C	2 h	decomposition
7.	$Pd(OH)_2$-C, K_2CO_3<br>TBHP (decane)	CH_2Cl_2	25 °C	18 h	SM

[a]optimization reactions were carried out on 0.20 mmol of substrate.
[b]yields are isolated after column chromatography.

Attempts toward the Cα-functionalization of enone (+)**-18:**

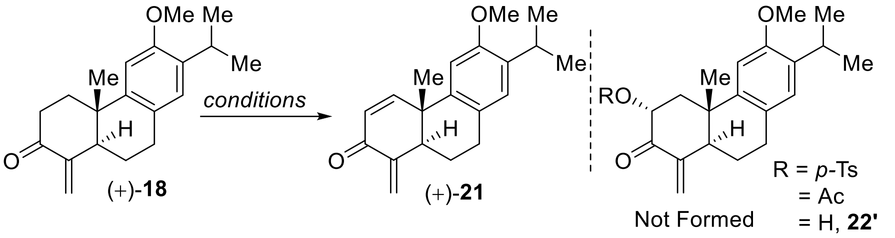

Entry	Reaction Condition	Solvent	Temp.	Time	Yield(%)[a,b]
1.	*P*-TSA<br>PhI, *m*-CPBA	CH_3CN	60 °C	6 h	68
2.	DMP, *P*-TSA	CH_3CN	100 °C	4 h	62
3.	PhI, aq. H_2O_2<br>$BF_3.OEt_2$, Ac_2O	CH_2Cl_2	25 °C	12 h	72
4.	LDA, DMDO	THF	–78 °C-25 °C	6 h	complex mixture
5.	LiHMDS, $P(OMe)_3$<br>O_2(g) balloon	THF	–78 °C-25 °C	6 h	SM
6.	I_2,CuO	DMF	100 °C	24 h	SM
7.	I_2, O_2(g)balloon	DMSO	25 °C	24 h	complex mixture

[a]optimization reactions were carried out on 0.20 mmol of substrate.
[b]yields are isolated after column chromatography.

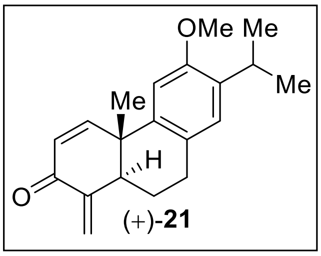

(4a*S*,10a*R*)**-7-isopropyl-6-methoxy-4a-methyl-1-methylene-4a,9,10,10a-tetrahydrophenanthren-2(1H)-one** [(+)-**21**]**:** (+)-**21** was obtained as colorless liquid. R_f = 0.37 (10% EtOAc in *n*-hexane).

^{1}H NMR (500 MHz, $CDCl_3$): δ 7.72 (d, *J* = 10.2 Hz, 1H), 6.98 (s, 1H), 6.90 (s, 1H), 6.26 (s, 1H), 6.15 (d, *J* = 10.1 Hz, 1H), 5.39 (s, 1H), 3.89 (s, 3H), 3.32 – 3.26 (m, 1H), 3.05 (d, *J* =

12.1 Hz, 1H), 2.95 (dd, J = 9.8, 4.3 Hz, 2H), 2.11 (d, J = 14.0 Hz, 1H), 1.96 – 1.89 (m, 1H), 1.28 (s, 3H), 1.24 (d, J = 6.9 Hz, 3H), 1.22 (d, J = 6.9 Hz, 3H).

^{13}C NMR (126 MHz, $CDCl_3$): δ 189.1, 160.0, 155.4, 145.8, 139.3, 135.8, 127.7, 127.5, 127.1, 119.1, 107.2, 55.7, 46.7, 41.5, 28.8, 27.1, 26.5, 22.8, 22.5, 20.7.

IR (neat) ν_{max} 3024, 2962, 1742, 1653, 1458, 1286, 972 cm^{-1}.
HRMS (ESI) m/z: $[M + H]^+$ calcd for $[C_{20}H_{25}O_2]^+$: 297.1849, found: 297.1863.
$[\alpha]^{20}_{589}$ = +123.76 (c = 0.45, $CHCl_3$).

Synthesis of the silyl enol ether (+)**-18a:**

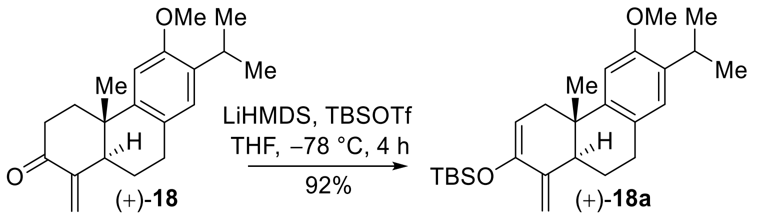

An oven-dried round-bottom flask purged with continuous N_2 (g) flow was charged with enone (+)**-18** (5.0 g, 16.754 mmol, 1.0 equiv.) in dry THF (40 mL) and was cooled to –78 °C. LiHMDS in *n*-hexane (1 M, 20.1 mL, 20.105 mmol, 1.2 equiv.) was slowly added within the reaction vessel over 10 minutes. After 15 min of stirring at the same temperature, *tert*-butyldimethylsilyl trifluoromethanesulfonate (TBS-OTf, 4.23 mL, 18.429 mmol, 1.1 equiv.) was directly added and chiller was put off allowing the reaction mixture to warm to 25 °C with continuous stirring. After complete consumption of the starting material (monitored by TLC) was quenched with saturated NH_4Cl solution (15 mL) and the biphasic mixture was separated with a separatory funnel. The aqueous phase was further extracted with EtOAc (10 mL X 3). The combined organic layers were washed with brine, dried over Na_2SO_4, and concentrated in vacuo under reduced pressure. Finally, the crude product was purified through column chromatography using EtOAc in *n*-hexane (1% EtOAc in *n*-hexane) to afford the silyl enol ether (+)**-18a** as a colorless liquid (6.361 g, 92% yield).

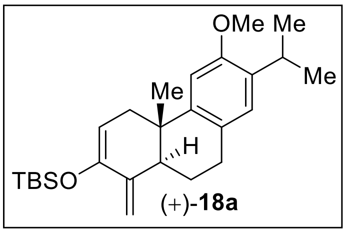

***tert*-butyl(((4a*S*,10a*R*)-7-isopropyl-6-methoxy-4a-methyl-1-methylene-1,4,4a,9,10,10a-hexahydrophenanthren-2-yl)oxy)dimethylsilane** [(+)-**18a**]: (+)-**18a** was obtained as colorless liquid (16.754 mmol, 6.361 g, 92% yield); R_f = 0.6 (2.5% EtOAc in *n*-hexane).

^{1}H NMR (500 MHz, $CDCl_3$): δ 6.90 (s, 1H), 6.74 (s, 1H), 5.46 (s, 1H), 5.12 – 5.06 (m, 1H), 4.98 (s, 1H), 3.81 (s, 3H), 3.28 – 3.21 (m, 1H), 2.88 – 2.81 (m, 2H), 2.62 (dd, *J* = 16.9, 6.0 Hz, 1H), 2.44 (dt, *J* = 10.2, 6.4 Hz, 2H), 2.10 (dq, *J* = 13.1, 2.7 Hz, 1H), 1.66 (dt, *J* = 12.4, 6.1 Hz, 1H), 1.22 (d, J = 4.6 Hz, 3H), 1.20 (d, *J* = 6.9 Hz, 3H), 1.06 (s, 3H), 1.00 (s, 9H), 0.19 (s, 3H), 0.18 (s, 3H).

^{13}C NMR (126 MHz, $CDCl_3$): δ 13C NMR (126 MHz, CDCl3) δ 155.4, 148.8, 144.2, 144.1, 134.9, 126.8, 126.5, 107.6, 106.3, 55.7, 44.2, 39.4, 37.2, 29.3, 26.7, 26.1, 26.1, 23.1, 23.0, 22.8, 21.1, 18.4, -4.0, -4.5.

IR (neat) υ_{max} 3110, 2883, 1712, 1584, 1367, 1331cm^{-1}.
HRMS (ESI) m/z: $[M + H]^+$ calcd for $[C_{26}H_{43}O_2Si]^+$: 413.2870, found: 413.2850

$[\alpha]^{20}_{589}$ = +64.52 (*c* = 0.32, $CHCl_3$).

Attempts toward the modified Rubottom oxidation of silyl enol ether (+)-**18a:**

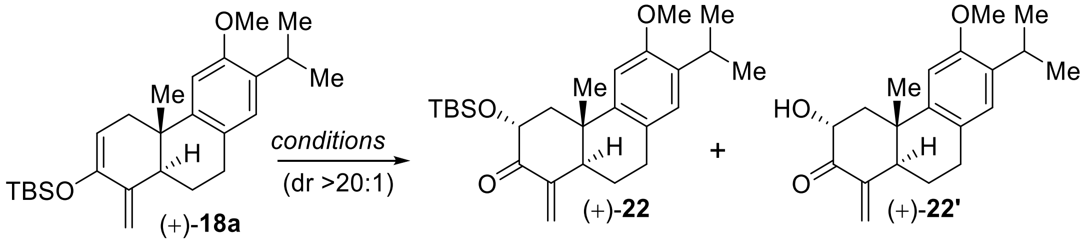

Entry	Oxidant	Buffer/Solvent	Temp./Time	Yield(%)[a,b]	Product Ratio (+)-22:(+)-22'
1.	DMDO	CH_2Cl_2	25 °C / 10 min	33	1.5:1
2.	DMDO	$NaHCO_3/CH_2Cl_2$	–20 °C-0 °C / 20 min	54	4:1
3.	DMDO	$AcOH/CH_2Cl_2$	–20 °C-0 °C / 20 min	SM	-
4.	*m*-CPBA	CH_2Cl_2	25 °C / 10 min	28[c]	0:1
5.	*m*-CPBA	$NaHCO_3/CH_2Cl_2$	–20 °C / 30 min	46	3:1
6.	*m*-CPBA	$AcOH/CH_2Cl_2$	–20 °C / 30 min	52	1:2.5
7.	*m*-CPBA	$NaHCO_3$/EtOAc	–20 °C / 30 min	73	4:1
8.	*m*-CPBA	AcOH/EtOAc	–20 °C / 30 min	63	1:3

[a]Optimization reactions were carried out on 0.24 mmol of substrate.
[b]Yields are isolated after column chromatography.
[c]Product isolated after treatment with TBAF

General procedure for modified Rubottom oxidation of silyl enol ether (+)**-18a:**

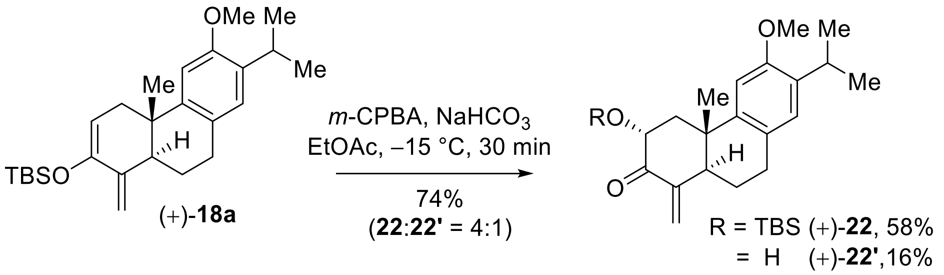

An oven-dried round-bottom flask charged with recrystallized *m*-CPBA (878 mg, 5.088 mmol, 1.05 equiv) in EtOAc (20 mL) was cooled to -15 °C with continuous stirring. To the cold reaction mixture solid $NaHCO_3$ (2.035 g, 24.231 mmol, 5 equiv.) was added in one portion followed by a slow addition of a solution of silyl enol ether (+)**-21** (2.0 g, 4.846 mmol, 1 equiv.) in EtOAc (10 mL) over 5 min and the reaction mixture was allowed to stir at the same temperature for additional 30 min until the full consumption of the starting material (monitored by TLC). After completion of the reaction, the reaction was quenched with 10% aqueous $Na_2S_2O_3$ (15 mL). The organic solvent from the biphasic mixture was separated using a separatory funnel. The aqueous phase was further extracted with EtOAc (15 mL X 3). The

combined organic layers were washed with brine, dried over Na_2SO_4, and concentrated in vacuo under reduced pressure. Finally, the crude product was purified through column chromatography by eluting with a gradient of 1.5-15% EtOAc/*n*-hexane to afford the separable mixture of silyloxy eneone [(+)**-23** (1.204 g, 58% yield)] along with the minor hydroxy enone [(+)**-17** (244 mg, 16% yield)].

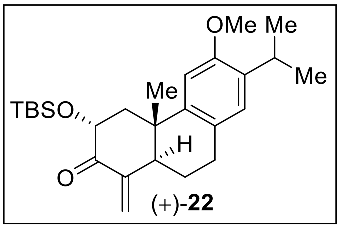

(3*R*,4a*S*,10a*R***)-3-((*tert*-butyldimethylsilyl)oxy)-7-isopropyl-6-methoxy-4a-methyl-1-methylene-3,4,4a,9,10,10a-hexahydrophenanthren-2(1H)-one** [(+)-**22]:** (+)-**22** was obtained as a colorless oil (4.846 mmol, 1.2 g, 58% yield); R_f = 0.35 (5% EtOAc in *n*-hexane).

^{1}H NMR (500 MHz, $CDCl_3$): δ 6.95 (s, 1H), 6.81 (s, 1H), 5.87 – 5.85 (m, 1H), 5.20 (dd, *J* = 2.7, 1.3 Hz, 1H), 4.47 – 4.42 (m, 1H), 3.87 (s, 3H), 3.30 – 3.25 (m, 1H), 2.91 (dd, *J* = 11.0, 6.2 Hz, 2H), 2.81 – 2.76 (m, 1H), 2.67 – 2.63 (m, 1H), 2.03 (dd, *J* = 14.7, 9.9 Hz, 2H), 1.79 – 1.73 (m, 1H), 1.24 (t, *J* = 3.4 Hz, 6H), 1.22 (s, 3H), 0.98 (d, *J* = 1.6 Hz, 9H), 0.23 (s, 3H), 0.16 (s, 3H).

^{13}C NMR (126 MHz, $CDCl_3$): δ 202.1, 155.3, 149.2, 142.5, 135.5, 126.9, 126.1, 117.8, 107.3, 77.3, 74.6, 55.7, 47.1, 46.6, 37.5, 28.8, 26.5, 25.9, 23.3, 22.8, 22.8, 22.6, 20.7, 18.5, -4.3, -5.1.

IR (neat) υ_{max} 3123, 2978, 1745, 1662, 1618, 1352,1316, cm^{-1};
HRMS (ESI) m/z: $[M + Na]^+$ calcd for $[C_{26}H_{40}O_3SiNa]^+$: 451.2639, found: 451.2628

$[\alpha]^{20}_{589}$ = +159.15 (*c* = 0.25, $CHCl_3$)

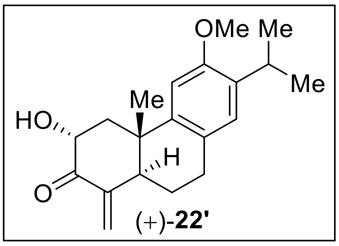

(3*R*,4a*S*,10a*R*)-3-hydroxy-7-isopropyl-6-methoxy-4a-methyl-1-methylene-3,4,4a,9,10,10a-hexahydrophenanthren-2(1H)-one [(+)-**22'**]**:** (+)-**22'** was obtained as a colorless gel (4.846 mmol, 244 mg, 16% yield); R_f = 0.25 (20% EtOAc in *n*-hexane).

^{1}H NMR (500 MHz, $CDCl_3$): δ 6.92 (s, 1H), 6.76 (s, 1H), 5.20 (s, 1H), 4.81 (s, 1H), 4.13 (q, *J* = 6.5 Hz, 1H), 3.82 (s, 3H), 3.29 – 3.24 (m, 1H), 2.87 (dd, *J* = 10.4, 6.1 Hz, 2H), 2.32 – 2.28 (m, 1H), 2.18 (td, *J* = 8.4, 7.2, 4.1 Hz, 2H), 1.86 (ddd, *J* = 9.7, 5.7, 2.9 Hz, 2H), 1.71 – 1.67 (m, 1H), 1.22 (t, *J* = 6.8 Hz, 6H), 1.05 (s, 3H).

^{13}C NMR (126 MHz, $CDCl_3$): δ 175.6, 155.1, 152.6,149.4,134.8, 126.7, 126.6, 107.6, 103.2, 73.1, 55.6, 46.3, 39.2, 36.8, 33.1, 29.4, 26.5, 22.8, 22.7, 21.6.

IR (neat) υ_{max} 3625, 3128, 3084, 1741, 1689, 1582, 1356 cm^{-1}.
HRMS (ESI) m/z: $[M + H]^+$ calcd for $[C_{20}H_{27}O_3]^+$: 313.1798, found: 313.1785

$[\alpha]^{20}_{589}$ = +215.48 (*c* = 0.32, $CHCl_3$).

Silylation of hydroxy enone (+)-**22'**:

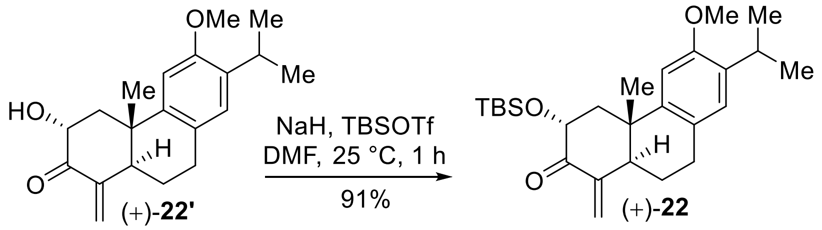

An oven-dried round-bottom flask was charged with hydroxy enone (+)-**22'** (225 mg, 0.715 mmol, 1 equiv.) in anhy. DMF (5 mL) and cooled to 0 °C with continuous stirring over an ice-water bath. Solid NaH [43 mg (60% in oil), 1.073 mmol, 1.5 equiv.] was added in one portion to the reaction vessel, and the reaction mixture was stirred for 15 min at 0°C followed by addition of *tert*-butyldimethylsilyl trifluoromethanesulfonate (197 μL, 0.858 mmol, 1.2 equiv). Then the reaction mixture was allowed to warm to 25 °C with continuous stirring for 1h until the full consumption of the starting material. Upon completion of the reaction (monitored by TLC) was quenched with saturated NH_4Cl solution (20 mL). The organic layer from the biphasic solution was separated using a separatory funnel. The aqueous phase was

further extracted with EtOAc (15 mL X 2). The combined organic layers were washed with brine (5 mL), dried over Na_2SO_4, and concentrated in vacuo under reduced pressure. Finally, the crude product was purified through column chromatography using 1.5% EtOAc in *n*-hexane to afford the silyloxy enone (+)-**22** as a colorless oil (279 mg, 91% yield).

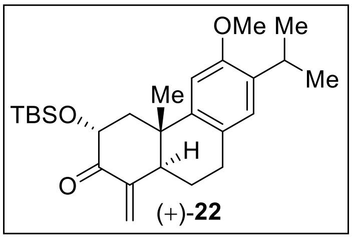

(3*R*,4a*S*,10a*R*)-3-((*tert*-butyldimethylsilyl)oxy)-7-isopropyl-6-methoxy-4a-methyl-1-methylene-3,4,4a,9,10,10a-hexahydrophenanthren-2(1H)-one [(+)-**22**]**:** (+)-**22** was obtained as a colorless oil (0.715 mmol, 279 mg, 91% yield); R_f = 0.35 (5% EtOAc in *n*-hexane).

Wittig olefination of silyloxy enone (+)-**22:**

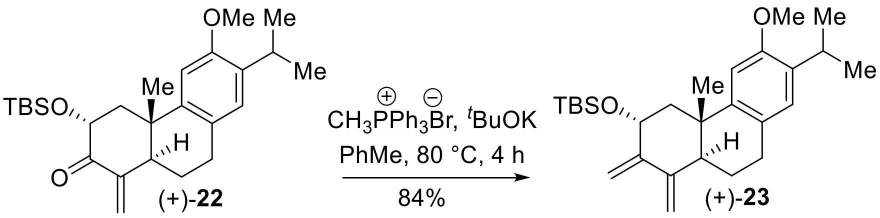

An oven-dried round-bottom flask was charged with $MePPh_3Br$ (8.333 g, 23.326 mmol, 2.0 equiv.) in $PhCH_3$ (50 mL) and cooled to 0 °C with continuous stirring over an ice-water bath. Solid ^{t}BuOK (2.617 g, 23.326 mmol, 2.0 equiv.) was added in portion wise to the reaction vessel, and the reaction mixture was allowed to stir for 30 min at 25 °C giving an orange solution, which again was cooled to 0 °C. A solution of silyloxy enone (+)-**22** (5.0 g, 11.663 mmol, 1 equiv.) dissolved in $PhCH_3$ (20 mL) was added to the reaction mixture at 0 °C. Then the reaction mixture was allowed to heat to 80 °C with a refluxing condenser over a preheated oil bath for 4 h. Upon completion of the reaction (monitored by TLC) was quenched with saturated NH_4Cl solution (20 mL). The organic layer from the biphasic solution was separated using a separatory funnel. The aqueous phase was further extracted with EtOAc (25 mL X 2). The combined organic layers were washed with brine (5 mL), dried over Na_2SO_4, and concentrated in vacuo under reduced pressure. Finally, the crude product was purified through

column chromatography using EtOAc in *n*-hexane (1% EtOAc in *n*-hexane) to afford the silyloxy diene (+)-**23** as a colorless foam (4.180 g, 84% yield).

Note:- A total of 13.0 g of compound (+)-**23** was prepared in several batches of reaction, following the above mentioned procedure.

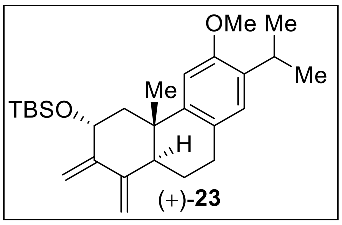

tert*-butyl(((**3*R*,4a*S*,10a*S)-7-isopropyl-6-methoxy-4a-methyl-1,2-dimethylene-1,2,3,4,4a,9,10,10a-octahydrophenanthren-3-yl)oxy)dimethylsilane** [(+)-**23**]**:** (+)-**23** was obtained as a colorless foam (11.663 mmol, 4.18 g, 84% yield); R_f = 0.45 (2.5% EtOAc in *n*-hexane).

^{1}H NMR (500 MHz, $CDCl_3$): δ 6.91 (s, 1H), 6.77 (s, 1H), 5.19 (t, *J* = 1.8 Hz, 1H), 5.04 (dt, *J* = 8.9, 2.6 Hz, 2H), 4.76 (d, *J* = 2.0 Hz, 1H), 4.50 (ddd, *J* = 10.9, 5.5, 2.7 Hz, 1H), 3.84 (s, 3H), 3.29 – 3.23 (m, 1H), 2.89 – 2.83 (m, 2H), 2.54 (dd, *J* = 12.3, 5.7 Hz, 1H), 2.36 (dd, *J* = 12.1, 2.2 Hz, 1H), 1.96 (ddd, *J* = 11.0, 5.7, 3.0 Hz, 1H), 1.77 – 1.70 (m, 1H), 1.22 (t, *J* = 7.3 Hz, 6H), 1.13 (s, 3H), 0.18 (d, *J* = 1.4 Hz, 6H).

^{13}C NMR (126 MHz, $CDCl_3$): δ 155.1, 153.0, 150.7, 143.8, 134.8, 126.8, 126.5, 108.8, 107.3, 106.3, 70.4, 55.6, 48.3, 47.5, 38.2, 29.0, 26.5, 26.0, 23.5, 22.8, 22.7, 21.1, 18.4, -4.6, -4.7.

IR (neat) υ_{max} 3148, 3015, 2989, 1664, 1592, 1360, 1281, 1065 cm^{-1}.
HRMS (ESI) m/z: $[M + H]^+$ calcd for $[C_{27}H_{43}O_2Si]^+$: 427.3027, found: 427.3026

$[\alpha]^{20}_{589}$ = +104.83 (*c* = 0.15, $CHCl_3$).

Benzylic oxidation of silyloxy diene (+)-**23:**

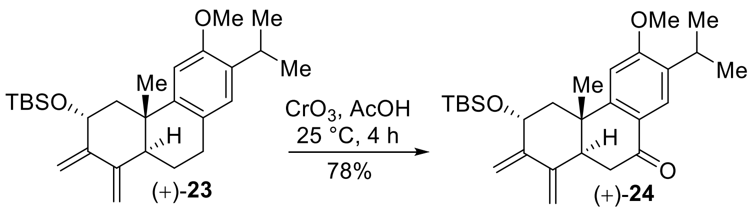

In an oven-dried round-bottom flask silyloxy diene (+)-**23** (5.2 g, 12.186 mmol, 1.0 equiv.) was dissolved in 30 mL of acetic acid. To the reaction mixture solid CrO_3 (2.436 g, 24.372 mmol, 2.0 equiv.) was added at 25 ºC and stirring was continued for additional 4 h. After completion of the reaction (monitored by TLC), the reaction mixture was diluted with ethyl acetate (20 mL) and quenched with saturated aqueous sodium bicarbonate solution (20 mL). The biphasic mixture was separated using a separatory funnel and the organic part was collected. The aqueous phase was further extracted with EtOAc (25 mL X 3). The combined organic layers were washed with brine and dried over Na_2SO_4 and concentrated under reduced pressure. Then the crude product was purified by flash silica gel column chromatography with 3% EtOAc in *n*-hexane to afford (+)-**24** as colorless liquid (4.188 g, 78% yield).

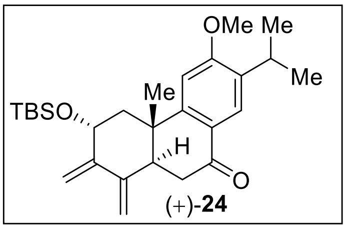

(3*R*,4a*S*,10a*S***)-3-((tert-butyldimethylsilyl)oxy)-7-isopropyl-6-methoxy-4a-methyl-1,2-dimethylene-2,3,4,4a,10,10a-hexahydrophenanthren-9(1H)-one** [(+)-**24]:** (+)-**24** was obtained as a colorless liquid (12.186 mmol, 4.188 g of product, 78% yield); R$_f$ = 0.25 (5% EtOAc in *n*-hexane).

^{1}H NMR (500 MHz, $CDCl_3$): δ 7.97 (s, 1H), 6.80 (s, 1H), 5.27 (t, *J* = 1.6 Hz, 1H), 5.10 (t, *J* = 2.4 Hz, 1H), 5.06 (t, *J* = 2.4 Hz, 1H), 4.79 (t, *J* = 1.7 Hz, 1H), 4.50 (ddd, *J* = 9.2, 5.3, 2.5 Hz, 1H), 3.94 (s, 3H), 3.28 (p, *J* = 6.9 Hz, 1H), 2.89 – 2.82 (m, 1H), 2.76 (dd, *J* = 17.6, 4.1 Hz, 1H), 2.70 – 2.63 (m, 1H), 2.59 (dd, *J* = 12.2, 5.5 Hz, 1H), 1.80 (t, *J* = 11.9 Hz, 1H), 1.25 (d, *J* = 7.1 Hz, 6H), 1.23 (s, 3H), 1.01 (s, 9H), 0.20 (s, 3H), 0.19 (s, 3H).

^{13}C NMR (126 MHz, $CDCl_3$): δ 196.2, 161.6, 152.7, 151.9, 148.3, 135.9, 126.1, 123.9, 110.5, 107.3, 105.1, 77.2, 69.9, 55.4, 47.2, 46.4, 38.4, 37.5, 26.6, 25.9, 22.4, 22.4, 22.3, 18.4, -4.7, -4.7.

IR (neat) υ_{max} 3107, 2972, 2881, 1720, 1676, 1344 cm^{-1}.
HRMS (ESI) m/z: $[M + H]^+$ calcd for $[C_{27}H_{41}O_3Si]^+$: 441.2819, found: 441.2816

$[\alpha]^{20}{}_{589}$= +68.35 (*c* = 0.12, $CHCl_3$).

Desilylation of silyloxy diene (+)-**24:**

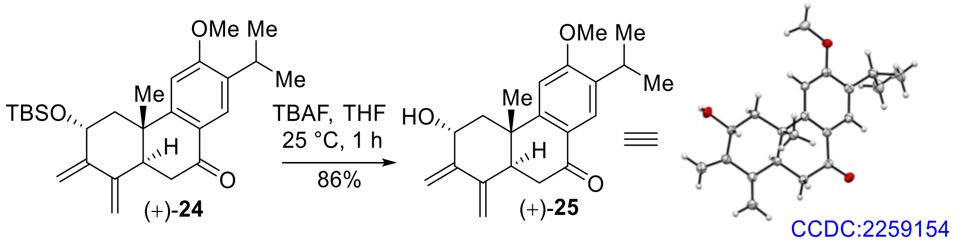

A solution of tetrabutylammonium fluoride in tetrahydrofuran (1.0 M, 10.0 mL, 9.984 mmol, 1.1 equiv.) was added to a solution of the silyloxy diene (+)-**24** (4.0 g, 9.076 mmol, 1.0 equiv.) in tetrahydrofuran (35 mL) at 25 ºC. The reaction mixture was stirred for 1 h at 25 ºC until the full consumption of the starting material (monitored by TLC). The product mixture was diluted sequentially with saturated aqueous ammonium chloride solution (20 mL), and ethyl acetate (15 mL). The resulting biphasic mixture was transferred to a separatory funnel and the layers that formed were separated. The organic layer was washed with brine, dried over Na_2SO_4 and concentrated under reduced pressure. Then the crude product was purified by flash column chromatography by eluting with a gradient of 8-12% EtOAc/*n*-hexane to afford hydroxy diene (+)-**25** as colorless solid (2.547 g, 86% yield).

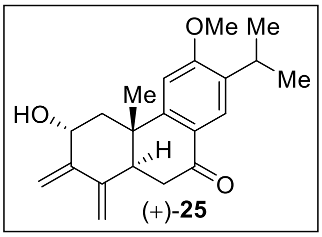

(3*R*,4a*S*,10a*S*)-3-hydroxy-7-isopropyl-6-methoxy-4a-methyl-1,2-dimethylene-2,3,4,4a,10,10a-hexahydrophenanthren-9(1H)-one [(+)-**25**]: (+)-**25** was obtained as a colorless solid (9.076 mmol scale, 2.547 g, 86% yield); R_f = 0.25 (30% EtOAc in *n*-hexane). Crystallization through slow diffusion with methanol afforded colorless needle shaped crystals, which was characterized by single crystal X-ray crystallography.

^{1}H NMR (500 MHz, $CDCl_3$): δ 7.99 (s, 1H), 6.85 (s, 1H), 5.32 (t, *J* = 1.5 Hz, 1H), 5.13 (dt, *J* = 24.8, 1.9 Hz, 2H), 4.83 (t, *J* = 1.5 Hz, 1H), 4.54 (d, *J* = 10.8 Hz, 1H), 3.94 (s, 3H), 3.28 (p, *J* = 6.9 Hz, 1H), 2.86 (td, *J* = 12.0, 10.8, 4.9 Hz, 2H), 2.78 (dd, *J* = 17.5, 4.1 Hz, 1H), 2.68 (dd, *J* = 17.5, 13.6 Hz, 1H), 1.72 (t, *J* = 11.9 Hz, 1H), 1.26 – 1.22 (m, 9H).

^{13}C NMR (126 MHz, $CDCl_3$): δ 196.1, 161.7, 152.4, 152.3, 147.9, 136.1, 126.2, 123.8, 111.2, 106.6, 1052, 77.2, 69.4, 55.5, 46.7, 46.3, 38.6, 37.4, 26.6, 22.4, 22.1.

IR (neat) υ_{max} 3654, 3145, 2862, 2814,1681, 1362, 1206, 789 cm^{-1}.
HRMS (ESI) m/z: $[M + H]^+$ calcd for $[C_{12}H_{27}O_3]^+$: 327.1955, found: 327.1968

$[\alpha]^{20}_{589}$ = +112.81 (*c* = 0.33, $CHCl_3$).

Mitsunobu reaction of hydroxy diene (+)-**25**:

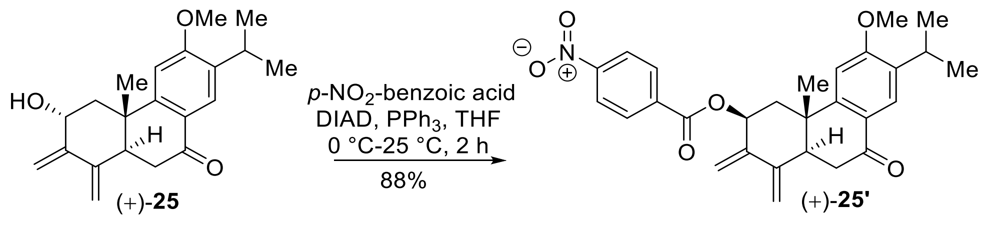

In an oven-dried round-bottom flask (+)-**25** (150 mg, 0.459 mmol, 1.0 equiv.) was dissolved in 6 mL of anhy. THF and cooled to 0 °C, over an ice-water bath. Required amount of *p*-nitrobenzoic acid (154 mg, 0.919 mmol, 2.0 equiv.), and triphenyl phosphine (242 mg, 0.92 mmol, 2.0 equiv.) were sequentially added to the reaction mixture. Then diisopropylazodicarboxylate (DIAD, 180 μL, 0.919 mmol, 2.0 equiv.) was added in it, and the reaction mixture was allowed to warm to 25 °C with continuous stirring for additional 2 h. After completion, the reaction mixture was concentrated and the resulting crude material

was dissolved in EtOAc (10 mL), washed with aqueous $NaHCO_3$ (10 mL), dried over Na_2SO_4, and concentrated in vacuum. Finally, the crude product was purified through column chromatography by eluting with 5-8% EtOAc in *n*-hexane to afford the benzoate derivative (+)-**25'** as yellow foam (192 mg, 88% yield).

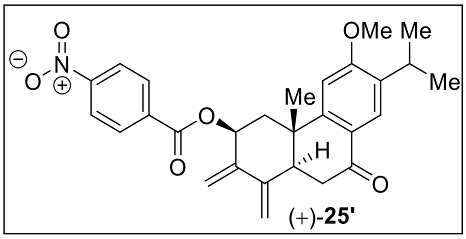

(3*S*,4a*S*,10a*S***)-7-isopropyl-6-methoxy-4a-methyl-1,2-dimethylene-9-oxo-1,2,3,4,4a,9,10,10a-octahydrophenanthren-3-yl 4-nitrobenzoate** [(+)-**25'**]**:** (+)-**25'** was obtained as yellow foam (0.459 mmol; 192 mg; 88% yield). R_f = 0.45 (20% EtOAc in *n*-hexane).

^{1}H NMR (500 MHz, $CDCl_3$): δ 8.35 – 8.29 (m, 2H), 8.21 (d, *J* = 8.9 Hz, 2H), 7.29 (s, 1H), 6.78 (s, 1H), 6.03 (dd, *J* = 4.4, 2.3 Hz, 1H), 5.40 (d, *J* = 2.1 Hz, 2H), 5.31 (d, *J* = 1.7 Hz, 1H), 4.97 (s, 1H), 3.93 (s, 3H), 3.32 – 3.25 (m, 1H), 3.00 – 2.93 (m, 1H), 2.87 (dd, *J* = 14.9, 2.4 Hz, 1H), 2.85 – 2.79 (m, 2H), 2.27 (dd, *J* = 14.8, 4.3 Hz, 1H), 1.45 (s, 3H), 1.25 (d, *J* = 7.1 Hz, 3H), 1.23 (d, *J* = 6.7 Hz, 3H).

^{13}C NMR (126 MHz, $CDCl_3$): δ 195.9, 164.0, 161.9, 152.8, 150.8, 145.9, 144.9, 136.0, 130.8, 126.4, 123.8, 123.8, 117.7, 112.1, 105.4, 75.3, 55.7, 46.5, 41.9, 38.3, 37.4, 26.7, 23.9, 22.6, 22.5.

IR (neat) υ_{max} 3154, 3074, 3026, 1738, 1648, 1584, 1529, 989, 815 cm^{-1}.
HRMS (ESI-TOF) m/z: $[M + H]^+$ calcd for $[C_{28}H_{30}NO_6]^+$: 476.2073, found: 476.2064

$[\alpha]^{20}_{589}$ = +117.2 (*c* = 0.54, $CHCl_3$).

Synthesis of the biomimetically hypothesized diene (+)-**3:**

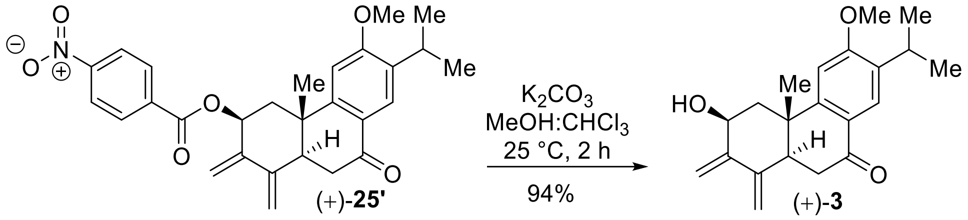

In an oven dried round-bottom flask, benzoate (+)-**25'** (160 mg, 0.336 mmol, 1.0 equiv.) was taken in a mixed solvent system of methanol and chloroform [MeOH: $CHCl_3$ (4:1)] (5 mL). To this solution was added K_2CO_3 (70 mg, 0.50 mmol, 1.5 equiv.) at 25 ºC and stirring was continued for 2 h until the completion of the reaction (judged by TLC analysis), the reaction mixture was diluted with water (6 mL). The organic layer was separated from the biphasic solution using a separatory funnel and the aqueous phase was further extracted with CH_2Cl_2 (8 mL X 2). The organic layers were combined, washed with brine, dried over Na_2SO_4 and concentrated on a rotary evaporator under reduced pressure. Finally, the crude product was purified through column chromatography with 20% EtOAc in *n*-hexane to afford the biomimetically hypothesized diene (+)-**3**, as a colorless gel (103 mg, 94% yield).

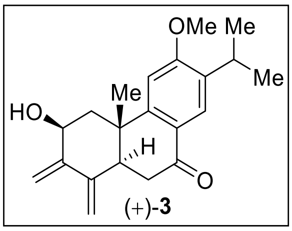

(3*S*,4a*S*,10a*S***)-3-hydroxy-7-isopropyl-6-methoxy-4a-methyl-1,2-dimethylene-2,3,4,4a,10,10a-hexahydrophenanthren-9(1H)-one** [(+)-**3**]**:** (+)-**3** was obtained as a colorless gel. (0.336 mmol, 103 mg, 94% yield); R_f = 0.22 (30% EtOAc in *n*-hexane).

^{1}H NMR (500 MHz, $CDCl_3$): δ 8.02 (s, 1H), 7.91 (s, 1H), 6.69 (s, 2H), 5.30 (s, 1H), 5.03 (s, 1H), 3.94 (s, 3H), 3.92 (s, 3H), 3.41 (dd, *J* = 13.1, 4.0 Hz, 1H), 3.32 – 3.28 (m, 1H), 3.25 (dt, *J* = 11.6, 5.9 Hz, 2H), 3.18 (d, *J* = 15.8 Hz, 1H), 3.01 (t, *J* = 12.9 Hz, 2H), 2.93 (dd, *J* = 17.5, 4.3 Hz, 1H), 2.77 (d, *J* = 17.8 Hz, 2H), 2.72 (d, *J* = 3.9 Hz, 1H), 2.69 – 2.62 (m, 2H), 2.57 – 2.50 (m, 2H), 2.27 (dt, *J* = 13.1, 6.4 Hz, 1H), 1.93 – 1.88 (m, 1H), 1.27 (d, *J* = 5.3 Hz, 6H), 1.23 – 1.20 (m, 6H), 1.19 (s, 3H), 1.11 (s, 3H).

^{13}C NMR (126 MHz, $CDCl_3$): δ 196.4, 161.8, 153.9, 150.3, 146.3, 135.9, 126.1, 123.7, 113.4, 111.3, 105.8, 72.5, 55.6, 46.4, 43.6, 38.3, 37.5, 26.7, 24.0, 22.6, 22.5.

IR (neat) υ_{max} 3648, 3141, 2868, 2831,1662, 1573,1356, 789 cm^{-1}.

HRMS (ESI-TOF) m/z: $[M + H]^+$ calcd for $[C_{21}H_{27}O_3]^+$: 327.1960, found: 327.1968

$[\alpha]^{25}_{589}$ = +168.4 (*c* = 0.12, $CHCl_3$).

Attempts toward the oxidation of the hydroxy diene (+)-25: The Diels Alder reaction

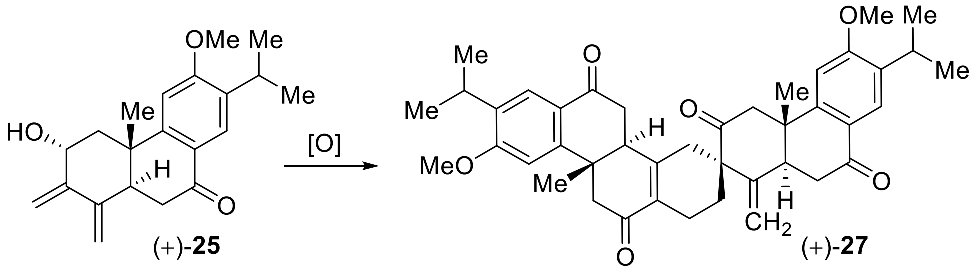

Diels-Alder Product formed exclusively

Salient Features:
1. excellent regioselectivity
2. excellent diastereoselectivity
3. mild reaction condition
4. no additional catalyst

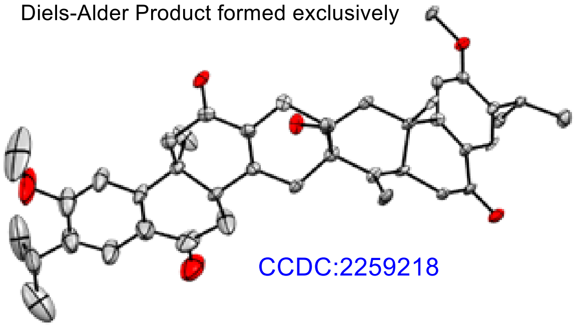

Entry	Reaction Condition	Solvent	Temp.	Time	Yield(%)[a,b]	Result
1.	MnO_2, Na_2CO_3	CH_2Cl_2	25 °C	24 h	-	SM
2.	SO_3.Py, Et_3N (Parikh-Doering Ox^n)	DMSO:CH_2Cl_2	0°C-25 °C	8 h	-	SM
3.	$(COCl)_2$, DMSO,Et_3N (Swern Ox^n)	CH_2Cl_2	–78 °C-25 °C	8 h	-	decomposition
4.	DMP, $NaHCO_3$	CH_2Cl_2	0°C-25 °C	1 h	92	D-A Product
5.	MnO_2	CH_2Cl_2	25 °C	24 h	-	SM
6.	TPAP, NMO (Ley-Grifith Ox^n)	CH_2Cl_2	25 °C	2 h	91	D-A Product
7.	DMP	CH_2Cl_2	0°C-25 °C	1 h	94	D-A Product

[a]optimization reactions were carried out on 0.15 mmol of substrate.
[b]yields are isolated after column chromatography.

DMP oxidation of the hydroxy diene (+)-**25:**

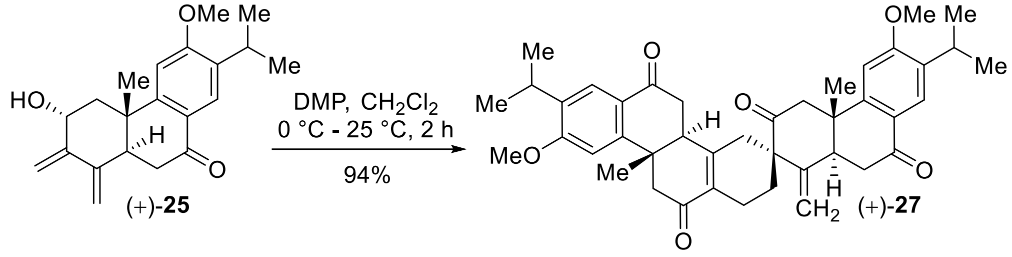

In an oven-dried round-bottom flask, the hydroxy diene (+)**-25** (64 mg, 0.196 mmol, 1.0 equiv.) in DCM (5 mL) was charged with Dess-Martin Periodinane (91 mg, 0.215 mmol, 1.1 equiv.) at 0 ºC. Then the reaction mixture was allowed to warm to 25 °C with continuous stirring until the reaction was completed (2 h). Upon completion of the reaction (as monitored by TLC analysis), the reaction mixture was quenched with saturated $NaHCO_3$ (5 mL). The biphasic mixture was then transferred to a separatory funnel and the organic part was collected. The aqueous part was further extracted with CH_2Cl_2 (5 mL X 3). The combined organic layers were washed with brine, saturated $Na_2S_2O_3$ (5 mL), dried over Na_2SO_4 and concentrated in vacuo under reduced pressure. Finally, the crude product was purified through flash column chromatography using 20% EtOAc in *n*-hexane to afford the Diels-Alder enone product (+)-**27** as colorless solid (60 mg, 94% yield).

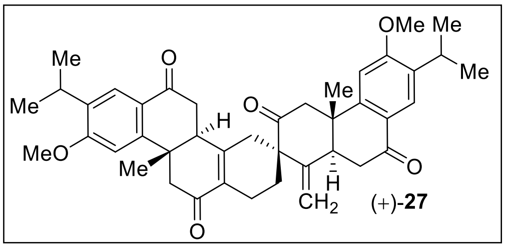

(3*S*,4b*S*,4a'*S*,10b*S*,10a'*S***)-7',8-diisopropyl-6',9-dimethoxy-4a',10b-dimethyl-1'-methylene-1,4,4b,4',4a',5,10b,10',10a',11-decahydro-1'H,2H-spiro[chrysene-3,2'-phenanthrene]-3',6,9',12-tetraone** [(+)-**27**]**:** (+)-**27** was obtained as a colorless solid (0.196 mmol, 60 mg, 94% yield); R_f = 0.25 (30% EtOAc in *n*-hexane). Crystallization through slow diffusion with methanol afforded colorless needle shaped crystals, which was characterized by single crystal X-ray crystallography.

^{1}H NMR (500 MHz, $CDCl_3$): δ 8.02 (s, 1H), 7.91 (s, 1H), 6.69 (s, 2H), 5.30 (s, 1H), 5.03 (s, 1H), 3.94 (s, 3H), 3.92 (s, 3H), 3.41 (dd, *J* = 13.1, 4.0 Hz, 1H), 3.32 – 3.28 (m, 1H), 3.25 (dt, *J* = 11.6, 5.9 Hz, 2H), 3.18 (d, *J* = 15.8 Hz, 1H), 3.01 (t, *J* = 12.9 Hz, 2H), 2.93 (dd, *J* = 17.5, 4.3 Hz, 1H), 2.77 (d, *J* = 17.8 Hz, 2H), 2.72 (d, *J* = 3.9 Hz, 1H), 2.69 – 2.62 (m, 2H), 2.57 – 2.50 (m, 2H), 2.27 (dt, *J* = 13.1, 6.4 Hz, 1H), 1.93 – 1.88 (m, 1H), 1.27 (d, *J* = 5.3 Hz, 6H), 1.23 – 1.19 (m, 9H), 1.11 (s, 3H).

^{13}C NMR (126 MHz, $CDCl_3$): δ 209.2, 196.2, 195.4, 194.8, 162.4, 162.2, 151.3, 150.8, 149.1, 137.0, 136.8, 132.9, 126.5, 126.4, 123.8, 123.7, 112.3, 105.4, 104.5, 55.7, 55.7, 54.3, 50.3, 50.0, 44.4, 43.2, 41.0, 40.1, 38.0, 37.2, 36.8, 26.8, 26.7, 25.3, 22.7, 22.5, 22.4, 22.4, 22.3, 22.3, 20.4.

IR (neat) υ_{max} 3138, 3083, 3062, 1768, 1651, 1648, 1532, 1336, 998, 801 cm^{-1}.
HRMS (ESI) m/z: $[M + H]^+$ calcd for $[C_{42}H_{49}O_6]^+$: 649.3524, found: 649.3527

$[\alpha]^{20}_{589}$ = +86.42 (*c* = 0.21, $CHCl_3$).

Equivalent controlled DMP oxidation of the hydroxy diene (+)-25:

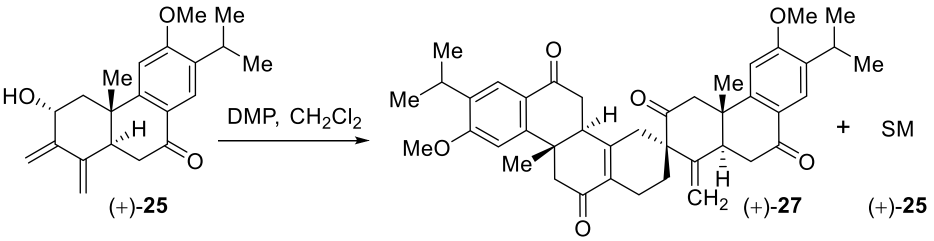

Entry	Eq. of DMP	Temp.	Time	Product Ratio (+)-25:(+)-27
1.	1.1	0°C-25 °C	2 h	0 : 1
2.	1.1	–30 °C	10 h	0 : 1
3.	0.5	0°C-25 °C	2 h	1 : 1
4.	0.5	–30 °C	10 h	1 : 1
5.	0.25	–30 °C	10 h	complex mixture

Optimization reactions were carried out on 0.15 mmol of substrate.

For the oxidation of (+)-**25** with 0.5 equiv. of DMP, please check the spectral data portion.

DMP oxidation of the hydroxy diene (+)-3:

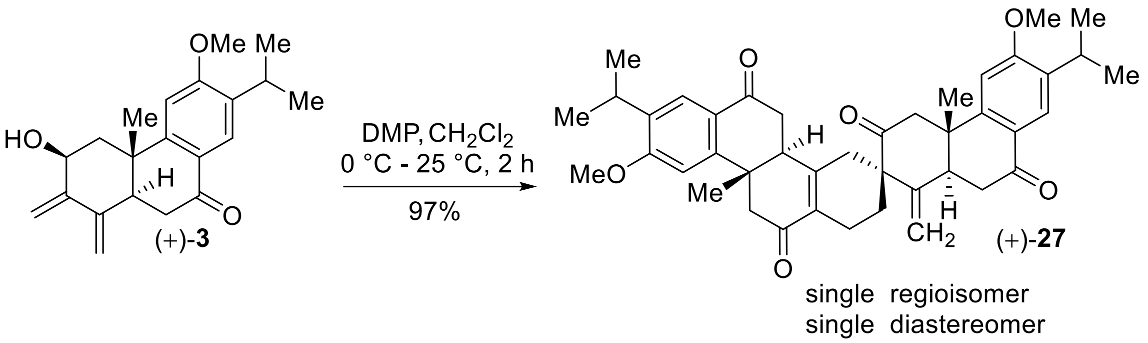

An oven-dried round-bottom flask the hydroxy diene (+)-**3** (80 mg, 0.245 mmol, 1.0 equiv.) in DCM (5 mL) was charged with Dess-Martin Periodinane (114 mg, 0.27 mmol, 1.1 equiv.) at 0 ºC. Then the reaction mixture was allowed to warm to 25 °C with continuous stirring, until the reaction was completed (2 h). Upon completion of the reaction (as monitored by TLC analysis), the reaction mixture was quenched with saturated $NaHCO_3$ (5 mL) and separated in a separatory funnel. The aqueous part was further extracted with CH_2Cl_2 (10 mL X 2). The combined organic layers were washed with brine (5 mL), dried over Na_2SO_4, and concentrated in vacuo under the reduced pressure. Finally, the crude product was purified through flash column chromatography using EtOAc in *n*-hexane (20% EtOAc in *n*-hexane) to afford the Diels-Alder enone product (+)-**27** as colorless solid (78 mg, 97% yield).
[All the physical properties and the spectroscopic data for the obtained compound found to be identical with the previously synthesized Diels-Alder enone product (+)-**27]**

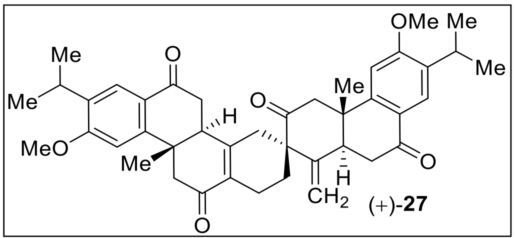

(3*S*,4b*S*,4a'*S*,10b*S*,10a'*S*)**-7',8-diisopropyl-6',9-dimethoxy-4a',10b-dimethyl-1'-methylene-1,4,4b,4',4a',5,10b,10',10a',11-decahydro-1'H,2H-spiro[chrysene-3,2'-**

phenanthrene]-3',6,9',12-tetraone [(+)-**27**]**:** (+)-**27** was obtained as a colorless solid (0.245 mmol, 78 mg, 97% yield); R*f* = 0.25 (30% EtOAc in n-hexane).

Desilylation of silyloxy diene (+)-**23:**

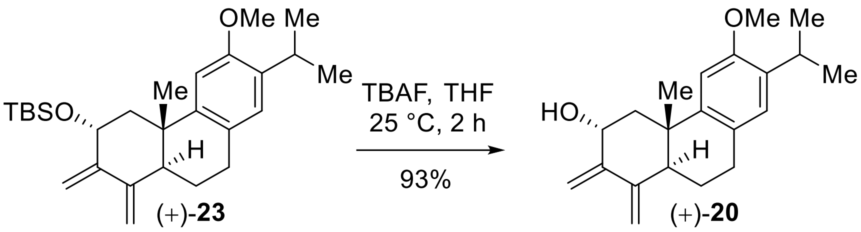

A solution of tetrabutylammonium fluoride in tetrahydrofuran (1.0 M, 18.044 mmol, 18.0 mL, 1.1 equiv.) was added to a solution of silyloxy diene (+)-**23** (7.0 g, 16.404 mmol, 1.0 equiv.) in tetrahydrofuran (50 mL) at 25 ºC. The reaction mixture was stirred for 2 h at 25 ºC until the completion of the reaction (monitored by TLC). The reaction mixture was diluted with saturated aqueous ammonium chloride solution (20 mL), and ethyl acetate (20 mL). The resulting biphasic mixture was transferred to a separatory funnel and the layers that formed were separated. The organic layer was washed with brine, dried over Na_2SO_4 and concentrated under reduced pressure. Then the crude product was purified by flash column chromatography by eluting with a gradient of 8-12% EtOAc/*n*-hexane to afford (+)-**20** as colorless gel (4.766 g, 93% yield).

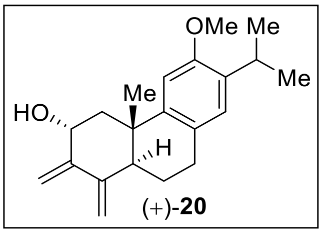

(3*R*,4a*S*,10a*S***)-7-isopropyl-6-methoxy-4a-methyl-1,2-dimethylene-1,2,3,4,4a,9,10,10a-octahydrophenanthren-3-ol** [(+)-**20**]**:** (+)-**20** was obtained as a colorless oil (5.68 mmol, 4.766 g, 93% yield); R_f = 0.3 (20% EtOAc in *n*-hexane).

^{1}H NMR (500 MHz, $CDCl_3$): δ 1H NMR (500 MHz, CDCl3) δ 6.92 (s, 1H), 6.81 (s, 1H), 5.23 (t, *J* = 1.7 Hz, 1H), 5.12 (t, *J* = 2.2 Hz, 1H), 5.05 (t, *J* = 2.1 Hz, 1H), 4.80 (t, *J* = 1.9 Hz, 1H), 4.52 (dd, *J* = 12.1, 5.8 Hz, 1H), 3.84 (s, 3H), 3.26 (p, *J* = 6.9 Hz, 1H), 2.90 – 2.84 (m,

2H), 2.79 (dd, J = 12.1, 5.8 Hz, 1H), 2.38 (dq, J = 12.2, 2.1 Hz, 1H), 2.01 – 1.95 (m, 1H), 1.77 – 1.72 (m, 1H), 1.59 (t, J = 11.9 Hz, 1H), 1.24 – 1.20 (m, 6H), 1.14 (s, 3H).

^{13}C NMR (126 MHz, $CDCl_3$): δ 155.3, 153.7, 150.3, 143.6, 135.1, 126.9, 126.4, 109.6, 107.3, 105.7, 70.1, 55.7, 48.3, 47.7, 38.6, 29.1, 26.6, 23.5, 22.9, 22.8, 21.2.

IR (neat) υ_{max} 3648, 3132, 2982, 2816, 1681, 1593, 1322, 1009, 789 cm^{-1}.
HRMS (ESI) m/z: $[M+Na]^+$ calcd for $[C_{21}H_{28}O_2Na]^+$: 335.1982, found: 335.1978

$[\alpha]^{20}_{589}$ = +73.25 (c = 0.12, $CHCl_3$).

General procedure for the oxidation of the hydroxy diene (+)**-20:**

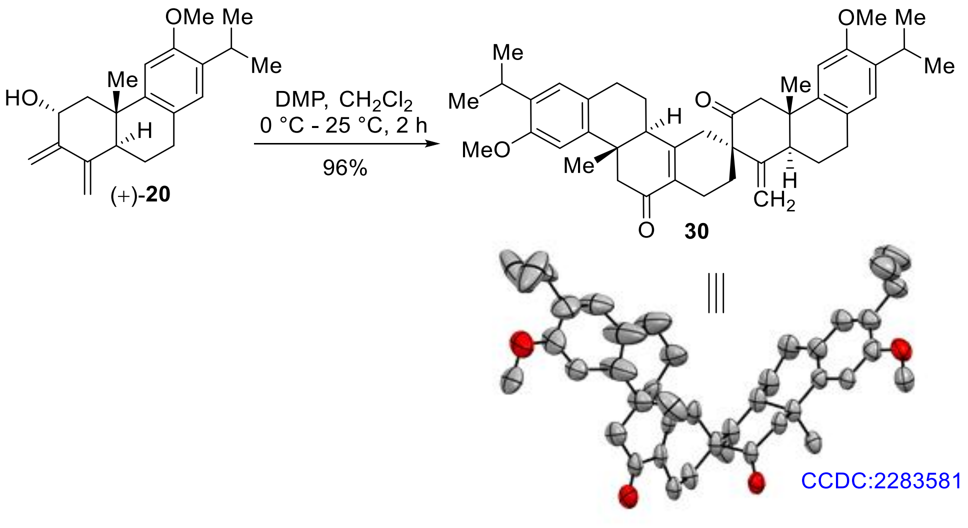

In an oven-dried round-bottom flask, the hydroxy diene (+)**-20** (1.2 g, 3.84 mmol, 1.0 equiv.) in CH_2Cl_2 (20 mL) was charged with Dess-Martin Periodinane (1.791 g, 4.224 mmol, 1.1 equiv.) at 0 ºC. Then the reaction mixture was warmed to 25 °C with continuous stirring until the reaction was completed (2 h). Upon completion of the reaction (as monitored by TLC analysis), the reaction mixture was quenched with saturated $NaHCO_3$ (10 mL). The resulting biphasic mixture was transferred to a separatory funnel and the layers that formed were separated. The aqueous phase was further extracted with CH_2Cl_2 (10 mL X 2). The combined organic layers were washed with brine, saturated $Na_2S_2O_3$ (5 mL), dried over Na_2SO_4, and concentrated in vacuo under reduced pressure. Finally, the crude product was purified through

flash column chromatography using EtOAc in *n*-hexane (10% EtOAc in *n*-hexane) to afford the Diels-Alder enone product (+)-**30** as colorless solid (1.152 g, 96% yield).

Note:- A total of 4.6 g of compound (+)-**30** was prepared in several batches of reaction following the above mentioned procedure.

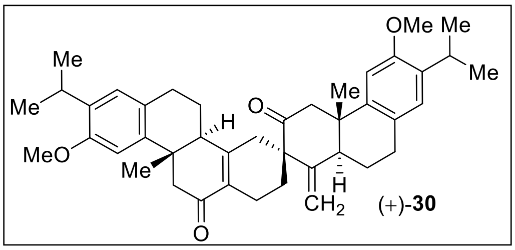

(3*S*,4b*S*,4a'*S*,10b*S*,10a'*S***)-7',8-diisopropyl-6',9-dimethoxy-4a',10b-dimethyl-1'-methylene-1,1',4b,4',4a',5,6,9',10b,10',10a',11-dodecahydro-2H,3'H-spiro[chrysene-3,2'-phenanthrene]-3',12(4H)-dione** [(+)-**30**]**:** (+)-**30** was obtained as colorless solid (3.84 mmol, 1.152 g, 96% yield); R_f = 0.45 (20% EtOAc in *n*-hexane). Crystallization through slow diffusion with methanol afforded colorless needle shaped crystals, which was characterized by single crystal X-ray crystallography.

^{1}H NMR (500 MHz, $CDCl_3$): δ 6.99 (s, 1H), 6.91 (s, 1H), 6.67 (s, 1H), 6.65 (s, 1H), 5.20 (s, 1H), 4.98 (d, *J* = 1.5 Hz, 1H), 3.81 (d, *J* = 2.0 Hz, 6H), 3.27 (dt, *J* = 13.7, 6.9 Hz, 2H), 3.13 (d, *J* = 16.3 Hz, 1H), 2.98 (ddd, *J* = 15.3, 10.1, 4.6 Hz, 4H), 2.93 – 2.87 (m, 4H), 2.76 (d, *J* = 12.4 Hz, 2H), 2.61 (d, *J* = 18.3 Hz, 1H), 2.54 (d, *J* = 16.6 Hz, 2H), 2.29 – 2.23 (m, 1H), 2.19 – 2.14 (m, 1H), 2.00 – 1.95 (m, 1H), 1.92 – 1.86 (m, 1H), 1.82 (dd, *J* = 14.0, 7.1 Hz, 1H), 1.70 (dd, *J* = 10.1, 2.7 Hz, 1H), 1.24 (d, *J* = 1.5 Hz, 3H), 1.23 (d, *J* = 1.6 Hz, 3H), 1.21 (d, *J* = 4.8 Hz, 3H), 1.20 (d, *J* = 4.7 Hz, 3H), 1.08 (s, 3H), 1.01 (s, 3H).

^{13}C NMR (126 MHz, $CDCl_3$): δ 211.0, 198.0, 155.6, 155.5, 152.2, 151.9, 142.3, 142.2, 135.5, 132.4, 127.0, 126.8, 126.4, 125.6, 110.2, 107.3, 106.5, 55.7, 55.6, 55.6, 54.2, 51.9, 51.6, 45.4, 44.3, 40.7, 40.2, 37.6, 29.4, 28.7, 26.7, 26.6, 25.6, 24.2, 23.4, 22.9, 22.8, 22.7, 22.3, 21.1, 20.6.

IR (neat) υ_{max} 3154, 3032, 3078, 1725,1638, 1614,1336, 801 cm^{-1}.

HRMS (ESI) m/z: $[M + Na]^+$ calcd for $[C_{42}H_{52}O_4Na]^+$: 643.3758, found: 643.3760

$[\alpha]^{20}_{589}$ = +95.35 (*c* = 0.20, $CHCl_3$).

Desymmetrization of the carbonyls: Luche reduction of the enone (+)-30:

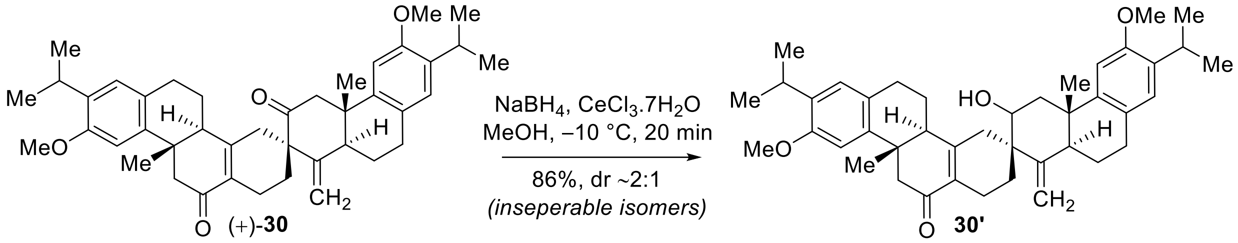

In an oven-dried round-bottom flask enone (+)-**30** (50 mg, 0.08 mmol, 1.0 equiv.) in 3 mL of methanol was charged with cerium (III) chloride heptahydrate (30 mg, 0.12 mmol, 1.5 equiv.) in one portion. After being stirred for 10 min, the mixture was cooled to −10°C and sodium borohydride (5 mg, 0.12 mmol, 1.5 equiv.) was added. The reaction mixture was stirred for an additional 20 min until the full consumption of the starting material. After completion of the reaction (monitored by TLC analysis), it was quenched with saturated NH_4Cl solution (4 mL). The resulting biphasic mixture was transferred to a separatory funnel and the layers that formed were separated. The aqueous phase was further extracted with EtOAc (6 mL X 2). The combined organic layers were dried over anhydrous Na_2SO_4 and concentrated in a rotary evaporator under reduced pressure. The residue was purified by silica gel column chromatography with 25% EtOAc in *n*-hexane to afford an inseparable diastereomeric mixture (2:1) of **30'** as white foam (43 mg, 86% yield).

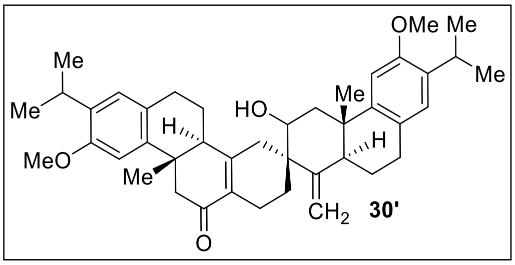

(3*S*,4b*S*,4a'*S*,10b*S*,10a'*S***)-3'-hydroxy-7',8-diisopropyl-6',9-dimethoxy-4a',10b-dimethyl-1'-methylene-1,3',4b,4',4a',5,6,9',10b,10',10a',11-dodecahydro-1'H,2H-spiro[chrysene-3,2'-phenanthren]-12(4H)-one** [**30'**]: **30'** was obtained as a white foam (0.08 mmol, 43 mg, 86% yield); R_f = 0.20 (30% EtOAc in *n*-hexane).

^{1}H NMR (500 MHz, $CDCl_3$): δ 6.96 (d, *J* = 3.2 Hz, 3H), 6.91 (d, *J* = 4.7 Hz, 3H), 6.80 (s, 1H), 6.77 (s, 2H), 6.65 (s, 3H), 5.26 (s, 1H), 5.15 (s, 2H), 4.97 (s, 2H), 4.85 (d, *J* = 1.2 Hz, 1H), 3.84 (s, 3H), 3.83 (s, 6H), 3.81 (d, *J* = 2.5 Hz, 9H), 3.26 (ddd, *J* = 13.9, 6.9, 2.7 Hz, 6H),

3.15 (d, *J* = 16.5 Hz, 2H), 3.09 (d, *J* = 15.9 Hz, 1H), 2.95 (dd, *J* = 9.0, 4.1 Hz, 4H), 2.92 – 2.89 (m, 4H), 2.89 – 2.87 (m, 2H), 2.87 – 2.83 (m, 2H), 2.78 (d, *J* = 12.9 Hz, 2H), 2.71 (d, *J* = 8.5 Hz, 2H), 2.68 (s, 4H), 2.62 (d, *J* = 11.8 Hz, 3H), 2.56 (d, *J* = 6.2 Hz, 2H), 2.53 (d, *J* = 6.6 Hz, 2H), 2.50 – 2.45 (m, 2H), 2.43 (d, *J* = 2.5 Hz, 2H), 2.39 (t, *J* = 6.5 Hz, 2H), 2.30 (d, *J* = 15.1 Hz, 2H), 2.25 (d, *J* = 11.5 Hz, 4H), 2.14 (d, *J* = 19.9 Hz, 4H), 2.09 – 2.04 (m, 4H), 1.97 – 1.88 (m, 6H), 1.84 (d, *J* = 12.6 Hz, 4H), 1.74 (dd, *J* = 8.3, 5.2 Hz, 4H), 1.67 (dd, *J* = 12.2, 5.0 Hz, 4H), 1.25 (d, *J* = 2.2 Hz, 6H), 1.24 (d, *J* = 3.9 Hz, 12H), 1.23 (s, 5H), 1.22 (d, *J* = 1.8 Hz, 10H), 1.20 (d, *J* = 1.9 Hz, 11H), 1.19 (s, 6H), 1.13 (s, 3H), 1.12 (s, 6H), 1.05 (s, 3H).

^{13}C NMR (126 MHz, $CDCl_3$): δ 197.8, 155.5, 155.4, 155.2, 155.1, 153.6, 145.2, 142.5, 142.1, 135.4, 135.2, 134.8, 132.8, 126.9, 126.8, 126.7, 126.6, 126.1, 125.8, 125.7, 125.6, 109.9, 108.8, 107.0, 107.1, 106.5, 106.1, 75.2, 71.8, 55.6, 55.54, 55.50, 52.2, 51.2, 46.3, 45.2, 44.2, 43.6, 43.4, 43.1, 42.5, 41.5, 40.4, 39.9, 39.4, 38.4, 38.2, 36.6, 30.3, 29.7, 29.24, 29.18, 28.7, 28.6, 27.8, 26.7, 26.55, 26.51, 26.48, 25.6, 23.6, 23.4, 23.3, 22.8, 22.7, 22.63, 22.60, 22.0, 21.9, 21.3, 20.3, 20.2, 19.3, 14.1.

HRMS (ESI) m/z: $[M + H]^+$ calcd for $[C_{42}H_{55}O_4]^+$: 623.4100, found: 623.4119

Desymmetrization of the carbonyls: Attempts toward the chemoselective ketal protection of (+)-**30:**

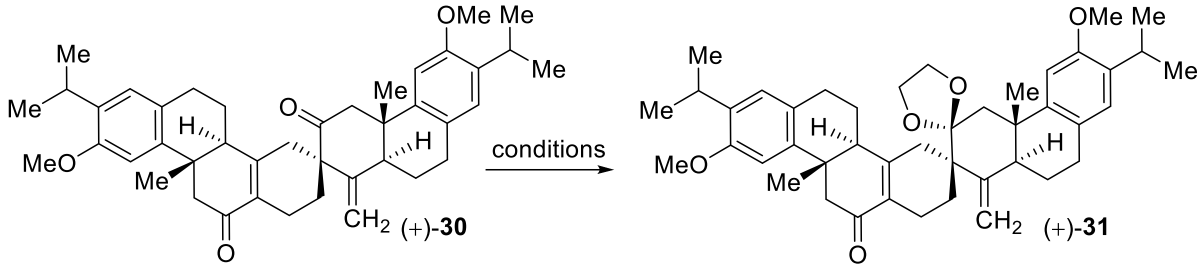

Entry	Lewis Acid	Solvent	Temp.	Time	Yield(%)[a,b]
1.	$(CH_2OTMS)_2$, TMSOTf	CH_2Cl_2	–78 °C-25 °C	14 h	SM
2.	$(CH_2OH)_2$, *P*-TSA.H_2O	CH_2Cl_2	25 °C	48 h	SM
3.	$(CH_2OH)_2$, *P*-TSA.H_2O	PhH	100 °C	24 h	61 (78, BRSM)
4.	$(CH_2OH)_2$, *P*-TSA.H_2O	$PhCH_3$	140 °C	10 h	71
5.	$(CH_2OH)_2$, $Bi(OTf)_3$	$PhCH_3$	140 °C	6 h	83
6.	$(CH_2OH)_2$, $Cu(OTf)_2$	$PhCH_3$	140 °C	8 h	79
7.	$(CH_2OH)_2$, $Zn(OTf)_2$	$PhCH_3$	140 °C	8 h	76

[a]Optimization reactions were carried out on 0.10 mmol of substrate.
[b]Yields are isolated after column chromatography.

Chemoselective ketal protection of (+)-**30:**

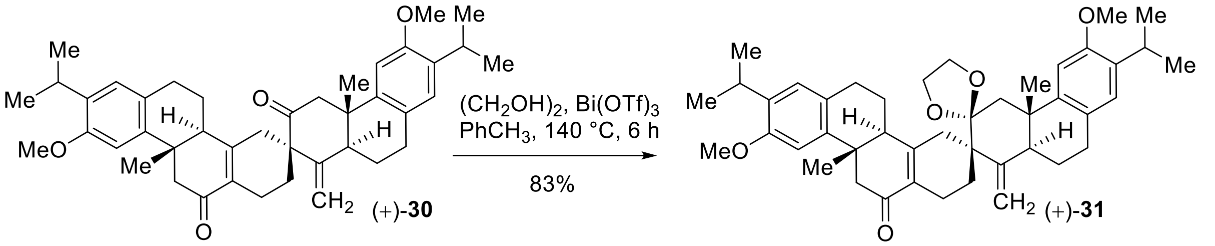

In an oven-dried round-bottom flask, the Diels-Alder adduct eneone (+)-**30** (3.96 g, 6.378 mmol, 1.0 equiv.) was charged with ethylene glycol (1.78 mL, 31.89 mmol, 5.0 equiv.) and $Bi(OTf)_3$ (418 mg, 0.637 mmol, 0.10 equiv.) in $PhCH_3$ (30 mL). Using a Dean-Stark trap, the reaction mixture was then refluxed on a preheated silicon oil bath at 140 °C for 6 h until the reaction was completed. Upon completion of the reaction (as monitored by TLC analysis), the reaction mixture was cooled down to 25 °C and then quenched with saturated $NaHCO_3$ solution (25 mL). The resulting biphasic mixture was transferred to a separatory funnel and the layers that formed were separated. The aqueous phase was further extracted with EtOAc (20 mL X 2). The combined organic layers were washed with brine, dried over Na_2SO_4 and concentrated in vacuo under reduced pressure. Finally, the crude product was purified through column chromatography by eluting with a gradient of 12-16% EtOAc/*n*-hexane to afford the ketal protected enone (+)-**31** as colorless oil (3.519 g, 83% yield).

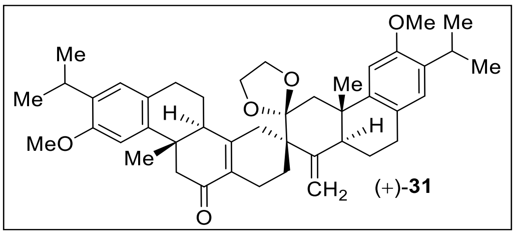

(3*S*,4b*S*,4a'*S*,10b*S*,10a'*S*)-7',8-diisopropyl-6',9-dimethoxy-4a',10b-dimethyl-1'-methylene-1,1',4b,4',4a',5,6,9',10b,10',10a',11-dodecahydro-2H-dispiro[chrysene-3,2'-phenanthrene-3',2''-[1,3]dioxolan]-12(4H)-one [(+)-**31**]: The compound (+)-**31** was obtained as colorless oil (6.378 mmol; 3.519 g; 83%). R_f = 0.28 (20% EtOAc in *n*-hexane).

^{1}H NMR (500 MHz, $CDCl_3$): δ 6.98 (s, 1H), 6.89 (s, 1H), 6.70 (d, *J* = 7.6 Hz, 2H), 5.23 (s, 1H), 4.92 (s, 1H), 4.11 (ddd, *J* = 15.3, 7.2, 4.5 Hz, 3H), 3.94 (t, *J* = 5.7 Hz, 1H), 3.86 (s, 3H), 3.81 (s, 3H), 3.31 – 3.23 (m, 2H), 2.98 – 2.92 (m, 2H), 2.80 (td, *J* = 10.0, 8.0, 3.9 Hz, 2H), 2.71 – 2.64 (m, 4H), 2.56 – 2.46 (m, 3H), 2.43 (t, *J* = 8.5 Hz, 1H), 2.28 (d, *J* = 13.8 Hz, 1H), 2.17 (d, *J* = 12.2 Hz, 1H), 2.04 (t, *J* = 12.4 Hz, 3H), 1.96 – 1.92 (m, 1H), 1.88 – 1.81 (m, 2H), 1.78 – 1.71 (m, 2H), 1.66 (dd, *J* = 12.8, 5.1 Hz, 2H), 1.41 (s, 3H), 1.25 (d, *J* = 4.0 Hz, 3H), 1.24 (d, *J* = 3.9 Hz, 3H), 1.22 (d, *J* = 6.8 Hz, 6H), 1.12 (s, 3H).

^{13}C NMR (126 MHz, $CDCl_3$): δ 197.9, 156.7, 155.7, 155.3, 149.4, 145.4, 141.9, 135.3, 135.0, 129.5, 127.2, 126.6, 126.5, 126.1, 113.5, 110.4, 108.2, 107.1, 65.6, 65.0, 55.9, 55.6, 49.5, 49.0, 48.0, 43.4, 42.9, 39.2, 38.4, 37.6, 30.3, 29.5, 26.7, 26.6, 26.4, 24.4, 24.1, 23.1, 22.9, 22.9, 22.7, 21.9, 19.9.

IR (neat) υ_{max} 3152, 3078, 3045, 1732, 1637, 1623, 1508, 1349, 801 cm^{-1}.
HRMS (ESI) m/z: $[M + H]^+$ calcd for $[C_{44}H_{57}O_5]^+$: 665.4206, found: 665.4213

$[\alpha]^{25}_{589}$ = +95.20 (*c* = 0.12, $CHCl_3$).

Diastereoselective Luche reduction of the enone (+)-**31:**

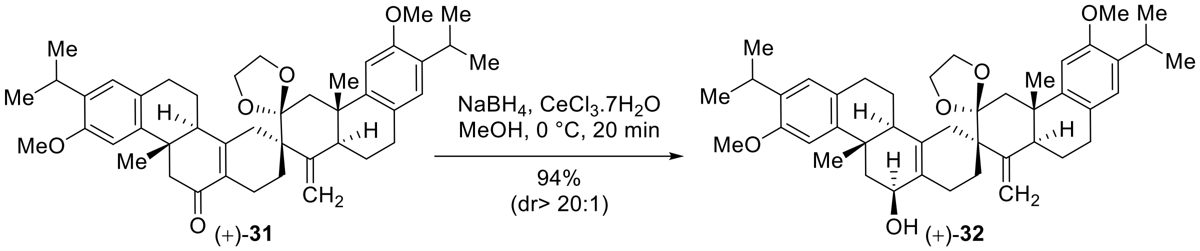

(+)-**31** (+)-**32**

In an oven-dried round-bottom flask the ketal protected enone (+)**-31** (3.48 g, 5.233 mmol, 1.0 equiv.) in 25 mL of methanol was added cerium (III) chloride heptahydrate (1.93 g, 7.85 mmol, 1.5 equiv.) in one portion to 25°C. After being stirred for 10 min, the mixture was cooled to 0°C, and sodium borohydride (297 mg, 7.85 mmol, 1.5 equiv.) was added portion wise with continuous stirring until the completion of the reaction at the same temperature. Upon completion of the reaction, it was quenched with 15 mL of saturated NH_4Cl solution. The resulting biphasic mixture was transferred to a separatory funnel and the layers that formed were separated. The aqueous phase was further extracted with EtOAc (20 mL X 2). The combined organic layers were dried over anhydrous Na_2SO_4 and concentrated in a rotary evaporator under reduced pressure. The residue was purified by silica gel column chromatography with 18% EtOAc in *n*-hexane to afford the ketal protected allyl alcohol (+)-**32** as colorless oil (3.281 g, 94% yield).

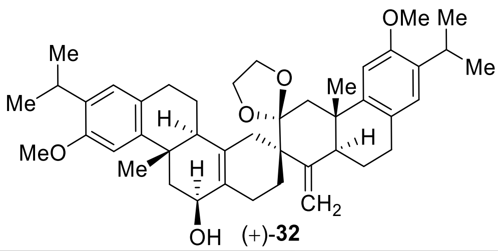

(+)-**32**

(3*S*,4b*S*,4a'*S*,10b*S*,10a'*S*,12*S***)-7',8-diisopropyl-6',9-dimethoxy-4a',10b-dimethyl-1'-methylene-1,1',4,4b,4',4a',5,6,9',10b,10',10a',11,12-tetradecahydro-2H-dispiro[chrysene-3,2'-phenanthrene-3',2''-[1,3]dioxolan]-12-ol** [(+)**-32]:** The compound (+)**-32** was obtained as colorless oil (5.233 mmol; 3.281 g; 94%). R$_f$ = 0.42 (30% EtOAc in *n*-hexane).

^{1}H NMR (500 MHz, $CDCl_3$): δ 6.97 (s, 1H), 6.85 (s, 1H), 6.76 (s, 1H), 6.71 (s, 1H), 5.27 (s, 1H), 4.92 (s, 1H), 4.13 – 4.08 (m, 4H), 3.94 – 3.90 (m, 1H), 3.86 (s, 3H), 3.82 (s, 3H), 3.26 (dq, *J* = 20.5, 6.9 Hz, 2H), 2.97 – 2.92 (m, 2H), 2.76 – 2.60 (m, 4H), 2.38 (dd, *J* = 16.4, 13.5 Hz, 2H), 2.24 (d, *J* = 13.8 Hz, 1H), 2.19 – 2.03 (m, 5H), 1.98 – 1.90 (m, 1H), 1.85 – 1.74 (m,

4H), 1.48 (dt, *J* = 12.5, 6.3 Hz, 1H), 1.29 (s, 3H), 1.26 (d, *J* = 5.1 Hz, 3H), 1.24 (d, *J* = 5.2 Hz, 3H), 1.22 (d, *J* = 5.1 Hz, 3H), 1.20 (d, *J* = 5.2 Hz, 3H), 1.13 (s, 3H).

^{13}C NMR (126 MHz, $CDCl_3$): δ 155.4, 155.3, 149.9, 145.7, 143.7, 134.8, 134.7, 131.9, 128.1, 127.6, 127.2, 126.3, 126.2, 113.9, 109.6, 109.0, 107.1, 70.0, 65.5, 65.0, 55.9, 55.7, 48.3, 47.5, 44.1, 43.2, 42.7, 38.4, 38.1, 35.6, 30.5, 29.5, 26.7, 26.6, 26.1, 26.0, 24.3, 23.4, 23.3, 23.0, 22.9, 22.8, 22.0.

IR (neat) υ_{max} 3630, 3143, 3081, 3064,1682, 1636, 1542, 1328, 801 cm^{-1}.
HRMS (ESI) m/z: $[M + Na]^+$ calcd for $[C_{44}H_{58}O_5Na]^+$: 689.4182, found: 689.4179

$[\alpha]^{20}_{589}$ = +71.6 (*c* = 0.3, $CHCl_3$).

Acetate protection of ketal protected allyl alcohol (+)-**32:**

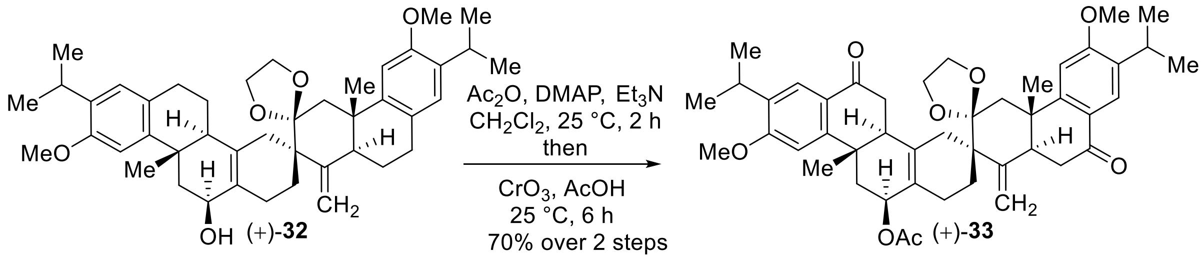

In an oven-dried round-bottom flask ketal protected allyl alcohol (+)-**32** (3.24 g, 4.857 mmol, 1.0 equiv.) was taken in dry CH_2Cl_2 (30 mL). To the solution were added Et_3N (2.0 mL, 14.573 mmol., 3 equiv.), DMAP (59 mg, 0.485 mmol., 0.1 equiv.) and acetic anhydride (550 mL, 5.83 mmol., 1.2 equiv.) sequentially. The resulting reaction mixture was allowed to stir at 25 °C until the full consumption of starting material (2 h). Upon completion of the reaction (as monitored by TLC analysis), the reaction mixture was diluted with water (20 mL). The resulting biphasic mixture was transferred to a separatory funnel and the layers that formed were separated. The aqueous phase was further extracted CH_2Cl_2 (20 mL X 2). The organic layers were combined, dried over Na_2SO_4 and concentrated on a rotary evaporator under reduced pressure. The crude acetate derivative was charged for the next step without further purification. R_f = 0.52 (20% EtOAc in *n*-hexane).

In an oven-dried round-bottom flask the crude acetate (4.857 mmol, 1.0 equiv.) was dissolved in 30 mL of acetic acid. To the reaction mixture solid CrO_3 (2.914 g, 29.142 mmol, 6.0 equiv.) was added and allowed to stir at 25 ºC for 6 h until the full consumption of the starting material. After completion of the reaction (monitored by TLC), the reaction mixture was diluted with ethyl acetate (30 mL) and further water (30 mL) was added. The resulting biphasic mixture was transferred to a separatory funnel and the layers that formed were separated. The aqueous phase was further extracted with EtOAc (25 mL X 2). The combined organic layers were washed with saturated aqueous $NaHCO_3$ solution (30 mL), brine and dried over Na_2SO_4 and concentrated under reduced pressure. Then the crude product was purified by flash chromatography by eluting with a gradient of 25-30% EtOAc/*n*-hexane to afford (+)-**33** as colorless gel (2.505 g, 70% over 2 steps).

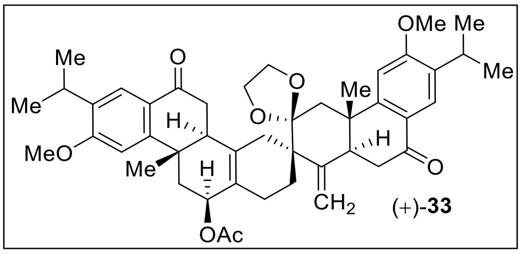

(3*S*,4b*S*,4a'*S*,10b*S*,10a'*S*,12*S***)-7',8-diisopropyl-6',9-dimethoxy-4a',10b-dimethyl-1'-methylene-6,9'-dioxo-1,1',4,4b,4',4a',5,6,9',10b,10',10a',11,12-tetradecahydro-2H-dispiro[chrysene-3,2'-phenanthrene-3',2''-[1,3]dioxolan]-12-yl acetate** [(+)-**33**]**:** The compound (+)-**33** was obtained as colorless gel (4.857 mmol; 2.505 g; 70% over 2 steps). R_f = 0.22 (30% EtOAc in *n*-hexane).

^{1}H NMR (500 MHz, $CDCl_3$): δ 8.02 (s, 1H), 7.82 (s, 1H), 6.79 (s, 1H), 6.71 (s, 1H), 5.53 – 5.42 (m, 1H), 5.33 (s, 1H), 5.00 (s, 1H), 4.11 (d, *J* = 2.6 Hz, 3H), 3.95 (s, 3H), 3.94 (s, 1H), 3.91 (s, 3H), 3.33 – 3.21 (m, 2H), 3.11 (dd, *J* = 13.7, 3.6 Hz, 1H), 2.83 (dd, *J* = 17.7, 13.6 Hz, 1H), 2.57 (ddd, *J* = 31.2, 17.0, 3.9 Hz, 2H), 2.46 – 2.31 (m, 4H), 2.27 (d, *J* = 13.7 Hz, 1H), 2.18 (dd, *J* = 13.0, 5.6 Hz, 2H), 2.13 (d, *J* = 13.5 Hz, 1H), 2.08 (s, 3H), 1.96 – 1.91 (m, 2H), 1.87 – 1.82 (m, 1H), 1.44 (s, 3H), 1.27 (d, *J* = 3.3 Hz, 3H), 1.26 (d, *J* = 3.1 Hz, 3H), 1.21 (d, *J* = 4.2 Hz, 6H), 1.20 (s, 3H).

^{13}C NMR (126 MHz, $CDCl_3$): δ 196.7, 196.2, 171.5, 162.0, 161.8, 154.1, 151.4, 148.0, 136.2, 136.0, 133.0, 126.8, 125.9, 125.0, 124.1, 123.9, 113.4, 111.0, 107.3, 105.0, 72.2, 65.6, 65.1,

55.7, 55.6, 47.6, 45.3, 42.6, 41.5, 40.5, 38.7, 38.2, 38.0, 37.4, 36.8, 35.8, 26.8, 25.6, 24.8, 23.5, 23.4, 23.0, 22.7, 22.5, 22.5, 22.4, 21.3.

IR (neat) υ_{max} 3118, 3082, 3038, 1789, 1716, 1668, 1614,1367, 978 cm^{-1}.
HRMS (ESI) m/z: $[M + Na]^+$ calcd for $[C_{46}H_{56}O_8Na]^+$: 759.3867, found: 759.3852.

$[\alpha]^{20}_{589}$ = +85.4 (c = 0.15, $CHCl_3$).

Sequential deketalization and deacetylation of the allyl alcohol acetate (+)-**33:**

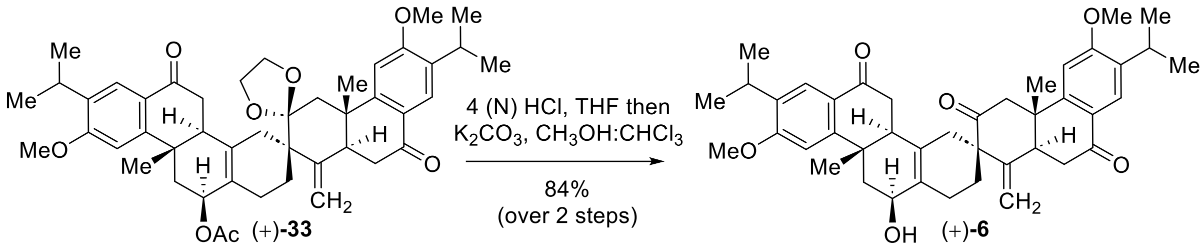

A round-bottom flask charged with allyl alcohol acetate (+)-**33** (2.43 g, 3.297 mmol, 1.0 equiv.) in a 1:1 mixture of 4 (*N*) HCl-THF (30 mL), was stirred for 4 h at 25 °C. After full consumption of the starting material (monitored by TLC), the reaction mixture was transferred to a separatory funnel and the organic phase was collected. The aqueous phase was extracted with EtOAc (20 mL X 2). The combined organic layers were washed with excess saturated $NaHCO_3$ solution (20 mL), dried over Na_2SO_4 and concentrated on a rotary evaporator under reduced pressure. Next, the crude yellow oil was charged for the next step without further purification. [R_f = 0.30 (30% EtOAc in *n*-hexane)].

In an oven dried round-bottom flask, the crude yellow oil was taken in a mixed solvent system of methanol and chloroform [MeOH: $CHCl_3$ (3:1)] (25 mL). To this solution was added K_2CO_3 (684 mg, 4.945 mmol, 1.5 equiv.) and stirred for 2 h at 25 ºC. After completion of the reaction (judged by TLC analysis), the reaction mixture was diluted with water (15 mL). The resulting biphasic mixture was transferred to a separatory funnel and the layers that formed were separated. The aqueous phase was further extracted with dichloromethane (20 mL X 2), the combined organic layers were dried over Na_2SO_4 and concentrated on a rotary evaporator under reduced pressure. The residue was purified by silica gel column chromatography by eluting with a gradient of 30-35% EtOAc/*n*-hexane to provide (+)-**6** as a pale-yellow liquid (1.8 g, 84% yield over 2 steps).

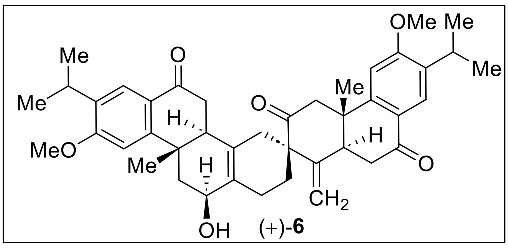

(3*S*,4b*S*,4a'*S*,10b*S*,10a'*S*,12*S*)-12-hydroxy-7',8-diisopropyl-6',9-dimethoxy-4a',10b-dimethyl-1'-methylene-1,2,4,4b,4',4a',5,10b,10',10a',11,12-dodecahydro-1'H,6H-spiro[chrysene-3,2'-phenanthrene]-3',6,9'-trione [(+)-**6**]**:** The compound (+)-**6** was obtained as pale-yellow liquid (3.297 mmol; 1.8 g; 84% over 2 steps). R_f = 0.22 (30% EtOAc in *n*-hexane).

^{1}H NMR (500 MHz, $CDCl_3$): δ 8.03 (s, 1H), 7.82 (s, 1H), 6.81 (s, 1H), 6.66 (s, 1H), 5.23 (s, 1H), 5.00 (s, 1H), 4.15 (d, *J* = 8.3 Hz, 1H), 3.92 (s, 3H), 3.89 (s, 3H), 3.53 (dd, *J* = 11.6, 5.5 Hz, 1H), 3.26 (dt, *J* = 16.4, 7.0 Hz, 2H), 3.01 (d, *J* = 12.6 Hz, 1H), 2.86 (d, *J* = 12.6 Hz, 1H), 2.81 – 2.75 (m, 2H), 2.70 (dd, *J* = 16.2, 4.4 Hz, 1H), 2.60 (d, *J* = 7.0 Hz, 1H), 2.58 – 2.50 (m, 3H), 2.43 (d, *J* = 16.4 Hz, 1H), 2.27 (s, 1H), 2.24 – 2.19 (m, 1H), 2.10 (d, *J* = 7.1 Hz, 2H), 1.85 – 1.72 (m, 2H), 1.34 (s, 3H), 1.25 (d, *J* = 6.9 Hz, 3H), 1.23 (s, 3H), 1.20 (d, *J* = 6.2 Hz, 6H), 1.07 (s, 3H).

^{13}C NMR (126 MHz, $CDCl_3$): δ 210.0, 195.9, 195.5, 161.9, 161.8, 150.9, 150.5, 150.3, 136.7, 136.2, 132.5, 127.5, 126.3, 124.9, 124.2, 123.9, 111.2, 107.5, 105.1, 68.6, 55.5, 54.9, 50.1, 47.5, 42.6, 41.8, 41.3, 40.1, 39.9, 38.0, 37.4, 29.7, 26.7, 26.6, 26.6, 24.1, 22.5, 22.4, 22.3, 22.3, 21.9.

IR (neat) υ_{max} 3618, 3149, 3062, 3037, 1768, 1732, 1630,1529, 1364, 983 cm^{-1}.
HRMS (ESI) m/z: $[M + H]^+$ calcd for $[C_{42}H_{51}O_6]^+$: 651.3686, found: 651.3657.

$[\alpha]^{20}_{589}$ = +44.3 (*c* = 0.3, $CHCl_3$).

Attempts toward the Demethylation of aryl methyl ether (+)-**6:**

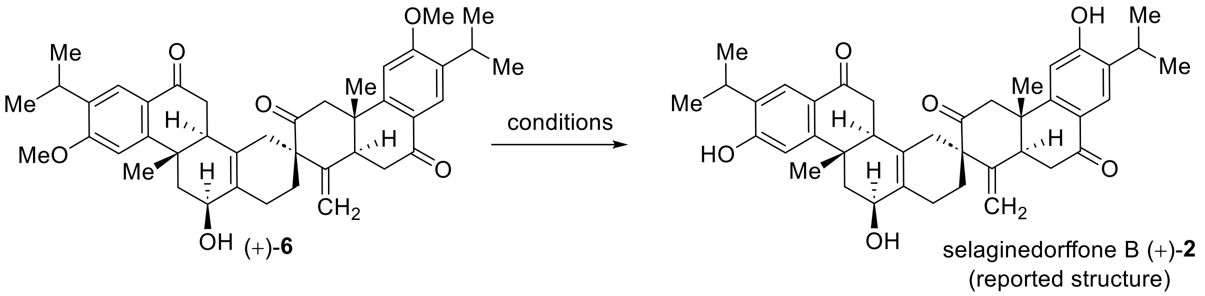

entry	demethylating agent	solvent	temp.	time	yield(%)[a,b]
1.	BBr_3	CH_2Cl_2	–78 °C	12 h	complex Mixture
2.	BBr_3	CH_2Cl_2	25 °C	4 h	decomposition
3.	$AlCl_3$, NaI	CH_2Cl_2:CH_3CN	25 °C	4 h	decomposition
4.	EtSH, NaH	DMF	140 °C	8 h	94

[a]reactions were carried out on 0.12 mmol of substrate.
[b]yields are isolated after column chromatography.

Demethylation of aryl methyl ether (+)-6: Completion of the reported structure Selaginedorffone B

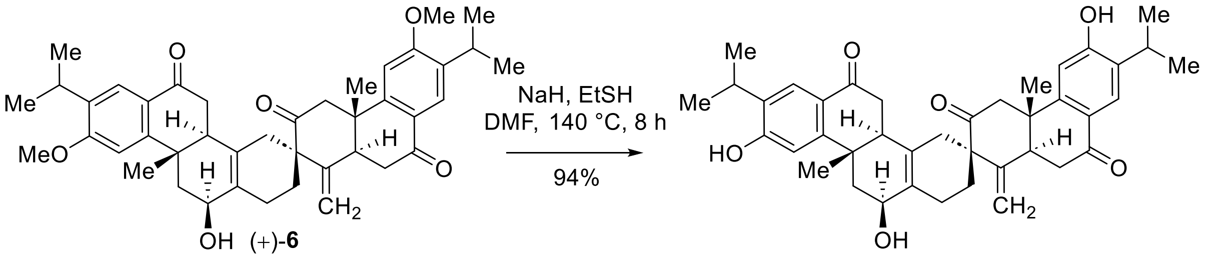

selaginedorffone B(+)-**2**
(reported structure)

To an ice cooled suspension of NaH [1.243 g (60% in oil), 31.112 mmol, 15.0 equiv.] in DMF (15 mL) were added EtSH (2.24 mL, 31.112 mmol, 15.0 equiv.) and allyl alcohol (+)-**6** (1.35 g, 2.074 mmol, 1.0 equiv.) in DMF (10 mL), and the mixture was refluxed for 8 h. After cooling to 0 °C, the reaction mixture was poured into an ice-cold solution of 1 (*N*) HCl (15 ml) and further diluted with EtOAc (15 mL). The resulting biphasic mixture was transferred to a separatory funnel and the layers that formed were separated. The organic layer was washed with excess brine solution, dried over Na_2SO_4 and concentrated under reduced pressure. The residue was purified by column chromatography on silica gel, eluting with

hexane–EtOAc (1:19) to give the reported structure of selaginedorffone B as colorless solid (1.214 g, 94% yield).

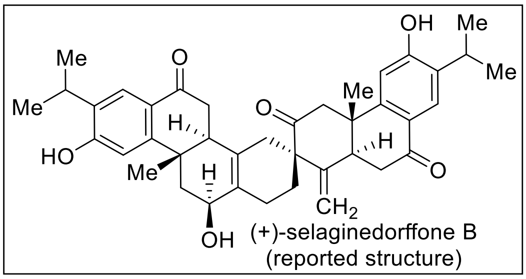

(3*S*,4b*S*,4a'*S*,10b*S*,10a'*S*,12*S*)**-6',9,12-trihydroxy-7',8-diisopropyl-4a',10b-dimethyl-1'-methylene-1,2,4,4b,4',4a',5,10b,10',10a',11,12-dodecahydro-1'H,6H-spiro[chrysene-3,2'-phenanthrene]-3',6,9'-trione** [(+)-**2**]**:** The compound (+)-**2** was obtained as colorless solid (2.074 mmol; 1.214 g; 94%). R$_f$ = 0.25 (in EtOAc).

^{1}H NMR (500 MHz, CD_3OD): δ 1H NMR 7.90 (s, 1H), 7.74 (s, 1H), 6.82 (s, 1H), 6.70 (s, 1H), 5.24 (s, 1H), 5.01 (s, 1H), 4.05 (t, *J* = 8.3 Hz, 1H), 3.63 (dd, *J* = 13.4, 3.7 Hz, 1H), 3.21 (q, *J* = 7.0 Hz, 2H), 3.13 (d, *J* = 12.7 Hz, 1H), 2.86-2.81 (m, 1H), 2.77 (d, *J* = 15.6 Hz, 1H), 2.71 (d, *J* = 11.7 Hz, 1H), 2.66 (d, *J* = 4.0 Hz, 1H), 2.63 (t, *J* = 3.6 Hz, 1H), 2.59 (d, *J* = 4.2 Hz, 1H), 2.45 (dd, *J* = 12.5, 6.0 Hz, 2H), 2.40 (d, *J* = 6.8 Hz, 1H), 2.23 (dt, *J* = 13.2, 6.5 Hz, 2H), 1.97 (d, *J* = 6.7 Hz, 1H), 1.90-1.86 (m, 1H), 1.82-1.76 (m, 1H), 1.26 (s, 3H), 1.21 (s, 3H), 1.20 (d, *J* = 2.6 Hz, 3H), 1.18 (s, 3H), 1.17 (s, 3H), 0.99 (s, 3H).

^{13}C NMR (126 MHz, CD_3OD): δ 212.5, 198.7, 198.3, 162.45, 162.43, 153.8, 153.1, 152.6, 135.8, 135.3, 132.9, 129.7, 127.5, 126.3, 123.9, 123.9, 113.2, 111.4, 111.2, 69.3, 56.3, 50.9, 47.9, 43.6, 42.4, 41.9, 41.6, 41.1, 39.0, 38.6, 28.0, 27.9, 27.8, 25.8, 24.9, 22.8, 22.72, 22.70, 22.6, 22.2.

IR (neat) υ_{max} 3652, 3617, 3063, 3056, 1767, 1728, 1683, 1637, 1598, 1361, 967 cm^{-1}.
HRMS (ESI-TOF) m/z: [M + H]$^+$ calcd for [$C_{40}H_{47}O_6$]$^+$: 623.3373, found: 623.3364

$[\alpha]^{20}_{589}$ = +92.6 (*c* = 0.12, MeOH); Isolation $[\alpha]^{22.4}_{D}$ = + 73.3 (c 0.07, MeOH).[2]

Comparison of ^{1}H-NMR Data of (+)-selaginedorffone B [(+)-**2**] (reported structure) of this report with isolated (+)-**2** by Long[2]:

Note: Discrepancies between the NMR spectra of the synthetic sample and isolated data provided by Long et al. [2], particularly within the aliphatic region of the ^{1}H-NMR spectrum can be seen. These differences may arise from several factors associated with the isolation sample: its impurity, low concentration, and the employment of a higher magnetic field strength (800MHz) during its ^{1}H-NMR analysis.

Long's isolation report (^{1}H-NMR, **800** MHz, CD_3OD)[2]	This report (^{1}H-NMR, **500** MHz, CD_3OD)	Difference (Δδ, ppm)
7.91 (s, 1H)	7.90 (s, 1H)	0.01
7.77 (s, 1H)	7.74 (s, 1H)	0.03
6.81 (s, 1H)	6.82 (s, 1H)	–0.01
6.74 (s, 1H)	6.70 (s, 1H)	0.04
5.29 (s, 1H)	5.24 (s, 1H)	0.05
4.99 (s, 1H)	5.01 (s, 1H)	–0.02
4.13 (br d, *J* = 5.7 Hz, 1H)	4.05 (t, *J* = 8.3 Hz, 1H)	0.08
3.61 (br d, *J* = 13.4 Hz, 1H)	3.63 (dd, *J* = 13.4, 3.7 Hz, 1H)	–0.02
3.26 (m, 1H)	3.24 – 3.17 (q, *J* = 7.0 Hz, 2H)	–
3.21 (m, 1H)		
3.15 (d, *J* = 13.1 Hz, 1H)	3.13 (d, *J* = 12.7 Hz, 1H)	0.02
2.94 (m, 1H)	–	–
2.84 (d, *J* = 13.1 Hz, 1H)	2.86 – 2.81 (m, 1H)	0.01
2.83 (d, *J* = 17.4 Hz, 1H)	2.77 (d, *J* = 15.6 Hz, 1H)	0.06
2.80 (dd, *J* = 17.2, 13.4 Hz, 1H)	2.71 (d, *J* = 11.7 Hz, 1H)	0.09
2.64 (br d, *J* = 15 Hz, 1H)	2.66 (d, *J* = 4.0 Hz, 1H)	–0.02
–	2.63 (t, *J* = 3.6 Hz, 1H)	–
2.59 (br d, *J* = 17.2 Hz, 1H)	2.59 (d, *J* = 4.2 Hz, 1H)	0.00
2.57 (br d, *J* = 17.2 Hz, 1H)	2.45 (dd, *J* = 12.5, 6.0 Hz, 2H)	–
2.50 (d, *J* = 14.0 Hz, 1H)		
2.41 (br d, *J* = 17.0 Hz, 1H)	2.40 (d, *J* = 6.8 Hz, 1H)	0.01
2.39 (dd, *J* = 17.4, 15.0 Hz, 1H)	2.23 (dt, *J* = 13.2, 6.5 Hz, 2H)	–
2.21 (m, 1H)		

2.13 (m, 1H)	1.97 (d, *J* = 6.7 Hz, 1H)	0.16
1.90 (dd, *J* = 14.0, 13.4 Hz, 1H)	1.90–1.86 (m, 1H)	0.02
1.87 (m, 1H)	1.82 – 1.76 (m, 1H)	0.08
1.24 (d, *J* = 6.8 Hz, 3H)	1.26 (s, 3H)	–0.02
1.22 (d, *J* = 6.8 Hz, 3H)	1.21 (s, 3H)	0.01
1.19 (d, *J* = 6.8 Hz, 3H)	1.20 (d, *J* = 2.6 Hz, 3H)	–0.01
1.17 (d, *J* = 6.8 Hz, 3H)	1.18 (s, 3H	–0.01
1.16 (s, 3H)	1.17 (s, 3H)	–0.01
1.01 (s, 3H)	0.99 (s, 3H)	0.02

Comparison of ^{13}C-NMR Data of (+)-selaginedorffone B [(+)-**2**] (reported structure) of this report with isolated (+)-**2** by Long[2]:

Note: The low concentration of the isolation sample led to the absence of several peaks in the isolation ^{13}C NMR data. Accordingly, Long et al. utilized HMBC and HSQC spectra to determine the 13C chemical shift values, which might be the cause for the discrepancies.

Long's isolation report (^{13}C-NMR, **201** MHz, CD_3OD)[2]	This report (^{13}C-NMR, **126** MHz, CD_3OD)	Difference (Δδ, ppm)
212.0	212.5	–0.5
199.6	198.7	0.9
198.5	198.3	0.3
162.7	162.45	0.25
162.5	162.43	0.27
155.8	153.8	2.0
153.2	153.1	0.1
151.7	152.6	–0.9
135.9	135.8	0.1
134.9	135.3	–0.4
132.2	132.9	–0.7
127.7	129.7	–2.0
127.5	127.5	0.0

127.5	126.3	1.2
123.9	123.9	0.0
123.8	123.9	–0.1
111.5	113.2	–1.7
111.4	111.4	0.0
110.7	111.2	–0.5
68.5	69.3	–0.8
56.3	56.3	0.0
50.8	50.9	–0.1
45.1	47.9	–2.8
43.7	43.6	0.1
43.1	42.4	0.7
41.7	41.9	–0.2
-	41.6	–
-	41.1	–
38.3	39.0	–0.7
36.7	38.6	–1.9
36.5	-	–
27.9	28.0	–0.1
26.7	27.8	–1.1
26.7	25.8	0.9
23.4	24.9	–1.5
22.8	22.8	0.0
22.8	22.72	0.08
22.7	22.70	0.0
22.7	22.6	0.1
22.2	22.2	0.0

Because of the discrepancies in the NMR-spectrum between the synthetic compound and the isolation compound, the epimerization at the allyl hydroxy centre of (+)-**6** has been performed.

Epimerization of the allyl hydroxy center of the reported structure of Selaginedorffone B

Mitsunobu Reaction of the allyl alcohol (+)-6:

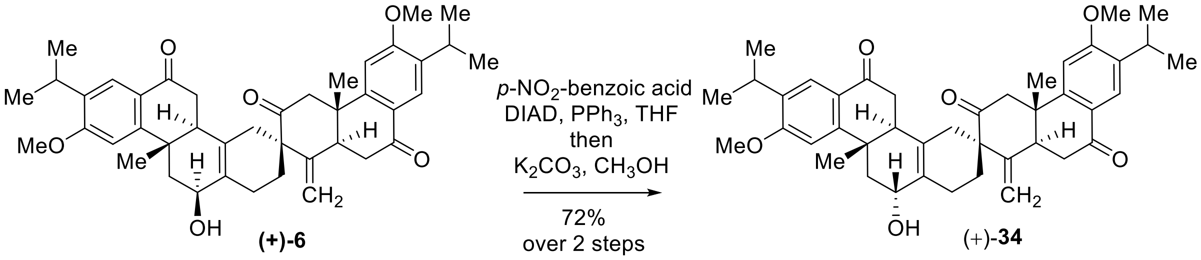

In an oven-dried round-bottom flask (+)-**6** (220 mg, 0.338 mmol, 1.0 equiv.) was dissolved in 8 mL of anhy. THF and cooled to 0 °C, over an ice-water bath. Required amount of *p*-nitrobenzoic acid (113 mg, 0.676 mmol, 2.0 equiv.), and triphenyl phosphine (178 mg, 0.676 mmol, 2.0 equiv.) were sequentially added to the reaction mixture. Then diisopropylazodicarboxylate (DIAD, 133 μL, 0.676 mmol, 2.0 equiv.) was added in it, and the reaction mixture was allowed to warm to 25 °C with continuous stirring for additional 3 h. After completion, the reaction mixture was concentrated and the resulting crude material was dissolved in EtOAc (10 mL), washed with aqueous $NaHCO_3$ (10 mL), dried over Na_2SO_4, and concentrated in vacuum. Finally, the crude product was charged for the next step without further purification.

In an oven dried round-bottom flask, the crude benzoate (0.338 mmol, 1.0 equiv.) was taken in a mixed solvent system of methanol and chloroform [MeOH: $CHCl_3$ (1:1)] (6mL). To this solution was added K_2CO_3 (140 mg, 1.014 mmol, 3.0 equiv.) at 25 ºC and stirring was continued for 2 h until the completion of the reaction (judged by TLC analysis), the reaction mixture was diluted with water (6 mL). The organic layer was separated from the biphasic solution using a separatory funnel and the aqueous phase was further extracted with CH_2Cl_2 (8 mL X 2). The organic layers were combined, washed with brine, dried over Na_2SO_4 and concentrated on a rotary evaporator under reduced pressure. Finally, the crude product was purified through column chromatography with a solvent gradient of 20-25% EtOAc in *n*-hexane to afford the epimerized allyl alcohol product (+)-**34** as a colorless gel (158 mg, 72% yield over 2 steps).

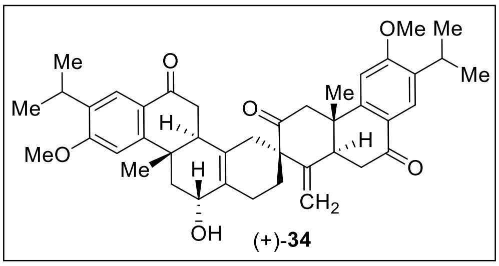

(3*S*,4b*S*,4a'*S*,10b*S*,10a'*S*,12*R*)-12-hydroxy-7',8-diisopropyl-6',9-dimethoxy-4a',10b-dimethyl-1'-methylene-1,2,4,4b,4',4a',5,10b,10',10a',11,12-dodecahydro-1'H,6H-spiro[chrysene-3,2'-phenanthrene]-3',6,9'-trione [(+)-**34**]**:** The compound (+)-**34** was obtained as colorless gel (0.338 mmol; 158 mg; 72% over 2 steps). R_f = 0.18 (30% EtOAc in *n*-hexane).

^{1}H NMR (500 MHz, CD_3OD): δ 8.04 (s, 1H), 7.78 (s, 1H), 6.81 (s, 1H), 6.69 (s, 1H), 5.15 (d, *J* = 1.2 Hz, 1H), 4.95 (d, *J* = 1.8 Hz, 1H), 3.91 (s, 6H), 3.80 (s, 1H), 3.53 (dd, *J* = 12.7, 4.5 Hz, 1H), 3.27 (ddd, *J* = 27.1, 13.8, 6.9 Hz, 3H), 3.07 (d, *J* = 12.6 Hz, 1H), 3.01 – 2.96 (m, 1H), 2.88 (d, *J* = 12.6 Hz, 1H), 2.82 (t, *J* = 5.1 Hz, 1H), 2.80 – 2.77 (m, 2H), 2.62 (d, *J* = 6.6 Hz, 1H), 2.58 (dd, *J* = 11.3, 5.9 Hz, 1H), 2.54 (d, *J* = 5.4 Hz, 1H), 2.44 (s, 1H), 2.34 (d, *J* = 17.3 Hz, 1H), 2.22 – 2.18 (m, 1H), 1.95 – 1.90 (m, 1H), 1.79 – 1.75 (m, 1H), 1.46 (s, 3H), 1.25 (d, *J* = 1.2 Hz, 3H), 1.23 (d, *J* = 6.7 Hz, 3H), 1.22 – 1.19 (m, 6H), 1.06 (s, 3H).

^{13}C NMR (101 MHz, CD_3OD): δ 210.1, 196.2, 195.7, 162.0, 161.9, 151.1, 151.0, 136.7, 135.9, 126.7, 126.4, 124.9, 124.8, 123.9, 113.3, 111.0, 106.8, 105.2, 68.1, 55.6, 55.0, 50.3, 47.8, 47.6, 42.6, 41.4, 39.3, 38.7, 38.0, 37.9, 37.4, 29.4, 26.7, 26.7, 26.2, 24.2, 22.6, 22.5, 22.4, 22.3, 21.9.

IR (neat) υ_{max} 3568, 3136, 3084, 3018, 1718, 1743, 1661, 1610, 1519, 1373, 986 cm^{-1}.
HRMS (ESI-TOF) m/z: $[M + H]^+$ calcd for $[C_{40}H_{51}O_6]^+$: 651.3686, found: 651.3687

$[\alpha]^{20}_{589}$ = +106.3 (*c* = 0.22, MeOH).

Demethylation of aryl methyl ether (+)-**34: Synthesis of the *epi*-selaginedorffone B (C2 epimer of the reported structure of selaginedorffone B)**

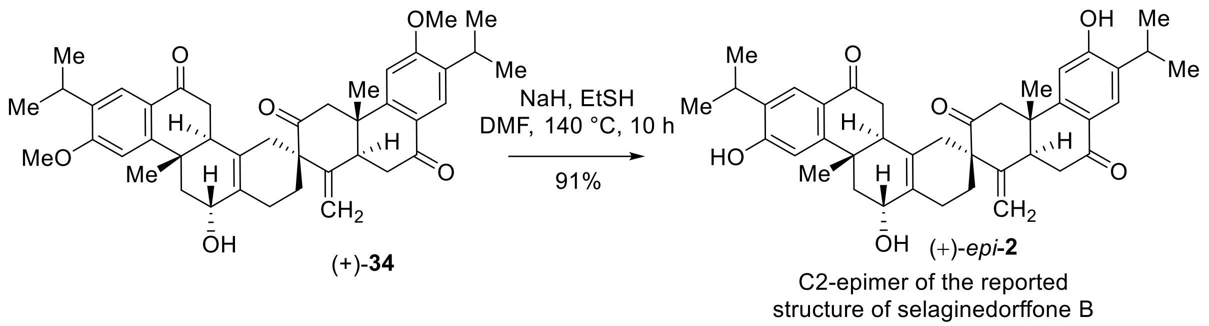

C2-epimer of the reported structure of selaginedorffone B

To an ice cooled suspension of NaH [83 mg (60% in oil), 2.074 mmol, 15.0 equiv.] in DMF (3 mL) were added EtSH (150 μL, 2.074 mmol, 15.0 equiv.) and allyl alcohol (+)-**34** (90 mg, 0.138 mmol, 1.0 equiv.) in DMF (2 mL), and the mixture was refluxed for 10 h. After cooling to 0 °C, the reaction mixture was poured into an ice-cold solution of 1 (N) HCl (5 ml) and further diluted with EtOAc (4 mL). The resulting biphasic mixture was transferred to a separatory funnel and the layers that formed were separated. The organic layer was washed with excess brine solution, dried over Na_2SO_4 and concentrated under reduced pressure. The residue was purified by column chromatography on silica gel, eluting with hexane–EtOAc (1:19) to give the C2-epimer of the reported structure of selaginedorffone B as colorless solid (78 mg, 91% yield).

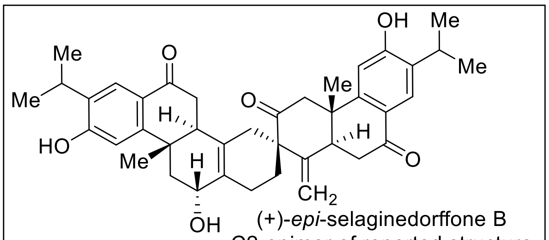

(+)-*epi*-selaginedorffone B
C2-epimer of reported structure

(3*S*,4b*S*,4a'*S*,10b*S*,10a'*S*,12*R***)-6',9,12-trihydroxy-7',8-diisopropyl-4a',10b-dimethyl-1'-methylene-1,2,4,4b,4',4a',5,10b,10',10a',11,12-dodecahydro-1'H,6H-spiro[chrysene-3,2'-phenanthrene]-3',6,9'-trione** [(+)-*epi*-**2**]**:** The compound (+)-*epi*-**2** was obtained as colorless solid (0.138 mmol; 78 mg; 91%). R_f = 0.22 (in EtOAc).

^{1}H NMR (500 MHz, CD_3OD): δ 1H NMR (500 MHz, CD_3OD) δ 7.92 (s, 1H), 7.67 (s, 1H), 6.84 (s, 1H), 6.73 (s, 1H), 5.12 (s, 1H), 4.93 (s, 1H), 3.66 (d, *J* = 4.0 Hz, 1H), 3.63 (d, *J* = 4.5 Hz, 1H), 3.27 – 3.23 (m, 1H), 3.21 (d, *J* = 5.0 Hz, 1H), 3.19 (d, *J* = 6.4 Hz, 1H), 2.86 (s, 1H), 2.82 (d, *J* = 4.2 Hz, 1H), 2.80 – 2.75 (m, 1H), 2.73 (d, *J* = 12.5 Hz, 1H), 2.67 (d, *J* = 4.0 Hz, 1H), 2.64 (d, *J* = 3.8 Hz, 1H), 2.57 – 2.53 (m, 1H), 2.50 (dd, *J* = 13.2, 5.4 Hz, 1H), 2.21 – 2.16

(m, 1H), 2.10 (dd, *J* = 13.1, 6.5 Hz, 1H), 2.03 – 1.98 (m, 1H), 1.80 (dd, *J* = 18.4, 6.4 Hz, 1H), 1.65 (dd, *J* = 13.3, 9.4 Hz, 1H), 1.48 (dt, *J* = 11.6, 5.8 Hz, 1H), 1.39 (s, 3H), 1.23 (d, *J* = 6.9 Hz, 3H), 1.21 (d, *J* = 3.5 Hz, 3H), 1.20 (d, *J* = 3.5 Hz, 3H), 1.17 (d, *J* = 6.9 Hz, 3H), 0.97 (s, 3H).

^{13}C NMR (126 MHz, CD_3OD): δ 212.1, 199.5, 198.4, 162.5, 162.4, 153.7, 153.2, 150.7, 135.8, 135.4, 135.1, 127.5, 127.3, 126.1, 125.4, 124.0, 112.8, 111.4, 110.6, 68.4, 56.4, 50.9, 48.1, 45.2, 43.5, 42.1, 39.9, 39.0, 38.6, 38.2, 29.9, 27.9, 27.9, 27.3, 25.1, 22.8, 22.8, 22.6, 22.6, 22.1.

IR (neat) υ_{max} 3643, 3623, 3074, 3024, 1753, 1746, 1624, 1572, 1539, 1351, 926 cm^{-1}.
HRMS (ESI-TOF) m/z: $[M + H]^+$ calcd for $[C_{40}H_{47}O_6]^+$: 623.3373, found: 623.3384

$[\alpha]^{20}_{589}$ = +61.6 (*c* = 0.12, MeOH); Isolation $[\alpha]^{22.4}_{D}$ = + 73.3 (*c* 0.07, MeOH)[2].

Comparison of ^{1}H-NMR Data of (+)-*epi*-selaginedorffone B [(+)-*epi*-**2**] (C2-epimer of the reported structure, this report) with isolated (+)-**2** by Long[2]:

Long's isolation report (^{1}H-NMR, **800** MHz, CD_3OD)[2]	This report (^{1}H-NMR, **500** MHz, CD_3OD)	Difference (Δδ, ppm)
7.91 (s, 1H)	7.92 (s, 1H)	–0.01
7.77 (s, 1H)	7.67 (s, 1H)	0.10
6.81 (s, 1H)	6.84 (s, 1H)	–0.03
6.74 (s, 1H)	6.73 (s, 1H)	0.01
5.29 (s, 1H)	5.12 (s, 1H)	0.17
4.99 (s, 1H)	4.93 (s, 1H)	0.06
4.13 (br d, *J* = 5.7 Hz, 1H)	–	–
3.61 (br d, *J* = 13.4 Hz, 1H)	3.66 (d, *J* = 4.0 Hz, 1H)	–0.05
–	3.63 (d, *J* = 4.5 Hz, 1H)	–
3.26 (m, 1H)	3.27 – 3.23 (m, 1H)	
3.21 (m, 1H)	3.21 (d, *J* = 5.0 Hz, 1H)	0.0

3.15 (d, *J* = 13.1 Hz, 1H)	3.19 (d, *J* = 6.4 Hz, 1H)	–0.04
2.94 (m, 1H)	2.86 (s, 1H)	0.08
2.84 (d, *J* = 13.1 Hz, 1H)	2.82 (d, *J* = 4.2 Hz, 1H)	0.03
2.83 (d, *J* = 17.4 Hz, 1H)	2.80 – 2.75 (m, 1H)	
2.80 (dd, *J* = 17.2, 13.4 Hz, 1H)	2.73 (d, *J* = 12.5 Hz, 1H)	0.07
2.64 (br d, *J* = 15 Hz, 1H)	2.67 (d, *J* = 4.0 Hz, 1H)	–0.02
–	2.64 (d, *J* = 3.8 Hz, 1H)	–
2.59 (br d, *J* = 17.2 Hz, 1H)	–	0.00
2.57 (br d, *J* = 17.2 Hz, 1H)	2.57 – 2.53 (m, 1H)	
2.50 (d, *J* = 14.0 Hz, 1H)	2.50 (dd, *J* = 13.2, 5.4 Hz, 1H)	
2.41 (br d, *J* = 17.0 Hz, 1H)	–	0.01
2.39 (dd, *J* = 17.4, 15.0 Hz, 1H)		
2.21 (m, 1H)	2.21 – 2.16 (m, 1H)	
2.13 (m, 1H)	2.10 (dd, *J* = 13.1, 6.5 Hz, 1H)	0.16
1.90 (dd, *J* = 14.0, 13.4 Hz, 1H)	2.03 – 1.98 (m, 1H)	0.02
–	1.80 (dd, *J* = 18.4, 6.4 Hz, 1H)	
–	1.65 (dd, *J* = 13.3, 9.4 Hz, 1H)	
1.87 (m, 1H)	1.48 (dt, *J* = 11.6, 5.8 Hz, 1H)	0.08
1.24 (d, *J* = 6.8 Hz, 3H)	1.39 (s, 3H)	–0.02
1.22 (d, *J* = 6.8 Hz, 3H)	1.23 (d, J= 6.9 Hz, 3H)	0.01
1.19 (d, *J* = 6.8 Hz, 3H)	1.21 (dd, *J* = 3.5 Hz, 6H)	–0.01
1.17 (d, *J* = 6.8 Hz, 3H)	1.20 (d, *J* = 3.5 Hz, 6H)	–0.01
1.16 (s, 3H)	1.17 (d, *J* = 6.9 Hz, 3H)	–0.01
1.01 (s, 3H)	0.97 (s, 3H)	0.02

Comparison of ^{13}C-NMR Data of (+)-*epi*-selaginedorffone B [(+)-*epi*-**2**] (C2-epimer of the reported structure, this report) with natural (+)-**2** by Long[2]:

Long's isolation report (^{13}C-NMR, **201** MHz, CD_3OD)[2]	This report (^{13}C-NMR, **126** MHz, CD_3OD)	Difference (Δδ, ppm)
212.0	212.1	–0.1

199.6	199.5	0.1
198.5	198.4	0.1
162.7	162.5	0.2
162.5	162.4	0.1
155.8	153.7	2.1
153.2	153.2	0.0
151.7	150.7	1.0
135.9	135.8	0.1
134.9	135.4	–0.5
132.2	135.1	–2.9
127.7	127.5	0.2
127.5	127.3	0.2
127.5	126.1	1.4
123.9	125.4	–1.5
123.8	124.0	–0.2
111.5	112.8	–1.3
111.4	111.4	0.0
110.7	110.6	0.1
68.5	68.4	0.1
56.3	56.4	–0.1
50.8	50.9	–0.1
45.1	48.1	–3.0
43.7	45.2	–1.5
43.1	43.5	–0.4
41.7	42.1	–0.4
-	39.9	–
-	39.0	–
38.3	38.6	–0.3
36.7	38.2	–1.5
36.5	29.9	6.6
27.9	27.90	0.0

26.7	27.86	–1.16
26.7	27.3	–0.6
23.4	25.1	–1.7
22.8	22.85	–0.05
22.8	22.82	–0.02
22.7	22.63	0.07
22.7	22.56	0.14
22.2	22.1	0.1

Note: The NMR spectrum for *epi*-selaginedorffone B, [(+)-*epi*-**2**] (C2-epimer of the reported structure) shows greater deviations than those observed for the previous compound.

The unambiguous determination of the structures of intermediate compounds **27** and **30** in the synthetic route through X-Ray crystallography, which are only a few steps away from the final product, as well as comprehensive 2D NMR analysis strongly suggests that the structure of the synthetic sample is matching with the reported structure. Consequently, it raises the possibility that the initial structural assignment of selaginedorffone B by the isolation chemists may have been incorrect.

Crystal Data and Structure Refinement of the hydroxy diene (+)-**25**

Crystallization through slow diffusion with methanol afforded colorless needle shaped crystals, which was characterized by single crystal X-ray crystallography.

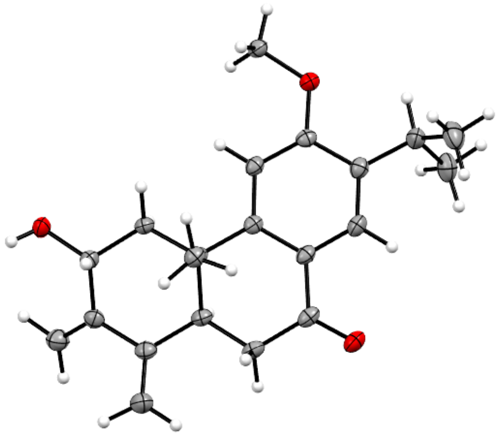

CCDC:2259154

Figure S1. Single crystal XRD structure of compound hydroxy diene (+)-**25**.

SK_0283A_ayan

Table 1 Crystal data and structure refinement for SK_0283A_ayan.

Identification code	SK_0283A_ayan
Empirical formula	$C_{42}H_{52}O_6$
Formula weight	652.83
Temperature/K	99.99(10)
Crystal system	orthorhombic
Space group	$P2_12_12_1$
a/Å	6.79620(10)
b/Å	21.0544(2)
c/Å	25.6129(2)
α/°	90
β/°	90
γ/°	90
Volume/Å^3	3664.95(7)
Z	4
ρ_{calc}g/cm^3	1.183
μ/mm^{-1}	0.615
F(000)	1408.0
Crystal size/mm^3	0.54 × 0.34 × 0.26
Radiation	Cu Kα (λ = 1.54184)
2Θ range for data collection/°	5.434 to 136.606
Index ranges	$-8 \le h \le 8$, $-23 \le k \le 25$, $-30 \le l \le 28$
Reflections collected	38876
Independent reflections	6176 [R_{int} = 0.1162, R_{sigma} = 0.0436]
Data/restraints/parameters	6176/0/475
Goodness-of-fit on F^2	1.047
Final R indexes [I>=2σ (I)]	R_1 = 0.0405, wR_2 = 0.1041
Final R indexes [all data]	R_1 = 0.0420, wR_2 = 0.1058
Largest diff. peak/hole / e Å^{-3}	0.18/-0.34
Flack parameter	-0.07(10)

Crystal Data and Structure Refinement of the Diels-Alder adduct (+)-27

Crystallization through slow diffusion with methanol afforded colorless needle shaped crystals, which was characterized by single crystal X-ray crystallography.

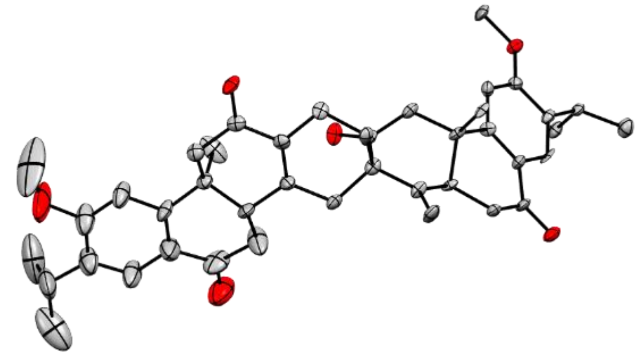

CCDC:2259218

Figure S2. Single crystal XRD structure of the Diels-Alder adduct (+)-**27** (Hydrogens are omitted for beter visibility).

SK-0284_auto_kp260423

Table 1 Crystal data and structure refinement for SK-0284_auto_kp260423.

Identification code	SK-0284_auto_kp260423
Empirical formula	$C_{42}H_{46}O_6$
Formula weight	646.79
Temperature/K	293(2)
Crystal system	triclinic
Space group	P1
a/Å	6.2642(3)
b/Å	8.0505(2)
c/Å	36.8967(6)
α/°	86.519(2)
β/°	86.864(2)
γ/°	67.266(3)
Volume/Å^3	1711.99(10)
Z	2
ρ_{calc}g/cm^3	1.255
μ/mm^{-1}	0.658
F(000)	692.0
Crystal size/mm^3	0.947 × 0.129 × 0.059
Radiation	CuKα (λ = 1.54184)
2Θ range for data collection/°	4.802 to 136.8
Index ranges	-5 ≤ h ≤ 7, -9 ≤ k ≤ 9, -42 ≤ l ≤ 44
Reflections collected	17137
Independent reflections	8151 [R_{int} = 0.0472, R_{sigma} = 0.0466]
Data/restraints/parameters	8151/3/880
Goodness-of-fit on F^2	1.828

Final R indexes [I>=2σ (I)]	R_1 = 0.1227, wR_2 = 0.3578
Final R indexes [all data]	R_1 = 0.1242, wR_2 = 0.3615
Largest diff. peak/hole / e Å^{-3}	1.16/-0.72
Flack parameter	0.07(14)

Crystal Data and Structure Refinement of the Diels-Alder adduct (+)-30

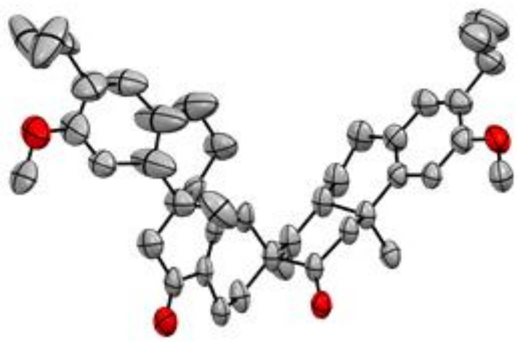

CCDC:2283581

Figure S3. Single crystal XRD structure of of the Diels-Alder adduct (+)-**30** (Hydrogens are omitted for beter visibility).

SK02101ayan_cm01-finalcif (1)

Table 1 Crystal data and structure refinement for SK02101ayan_cm01-finalcif (1).

Identification code	SK02101ayan_cm01-finalcif (1)
Empirical formula	$C_{88.5}H_{95}O_8$
Formula weight	1286.64
Temperature/K	100.00(10)
Crystal system	triclinic
Space group	P1
a/Å	7.45270(10)
b/Å	16.7911(2)
c/Å	18.3652(3)
α/°	105.9310(10)
β/°	93.8630(10)
γ/°	90.3130(10)
Volume/Å^3	2204.23(5)
Z	1
ρ_{calc}g/cm^3	0.969
μ/mm^{-1}	0.474

F(000)	690.0
Crystal size/mm^3	0.371 × 0.205 × 0.13
Radiation	Cu Kα (λ = 1.54184)
2Θ range for data collection/°	5.016 to 136.372
Index ranges	-8 ≤ h ≤ 8, -20 ≤ k ≤ 20, -22 ≤ l ≤ 22
Reflections collected	38540
Independent reflections	13589 [R_{int} = 0.0412, R_{sigma} = 0.0354]
Data/restraints/parameters	13589/3/1031
Goodness-of-fit on F^2	1.128
Final R indexes [I>=2σ (I)]	R_1 = 0.0813, wR_2 = 0.2294
Final R indexes [all data]	R_1 = 0.0849, wR_2 = 0.2368
Largest diff. peak/hole / e Å^{-3}	0.73/-0.35
Flack parameter	-0.2(3)

Computational Details

DFT calculations were carried out using the Gaussian 16 RevA.03 suite of programs at the B3LYP(GD3BJ)/6-31G(d) level of theory.[3] Saddle point structures were validated through the presence of a single imaginary frequency. Additionally, single-point solvation energy corrections were conducted at the SMD(DCM)-B3LYP(GD3BJ)/6-311++G(d,p) level of theory at 298 K.[4] Confirmation of minima and saddle points was obtained through intrinsic reaction coordinate (IRC) calculations.[5] Visualization and representation of geometries were performed using ChemDraw, ChemCraft, and CYLview software.[6-8]

HOMO-LUMO coefficients

HOMO Coefficients

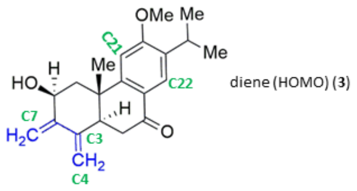

3(HOMO-2) Alpha occ 86 **OE=-0.242** is C4-p=0.2020 C21-p=0.1504 C22-p=0.1346 C7-p=0.1036 C3-p=0.1002

Figure S4. HOMO coefficients of molecule **3**.

In molecule **3**, the HOMO–2 serves as the highest occupied molecular orbital (HOMO). The coefficients of this HOMO–2 unequivocally indicate that the C4, C3, and C7 atoms within the diene moiety contribute 20%, 10%, and 10%, respectively, to the construction of the highest

occupied molecular orbital for molecule **3**. Conversely, the C21 and C22 atoms of the benzene ring contribute 15% and 13.5%, respectively, to the formation of the HOMO–2 orbital.

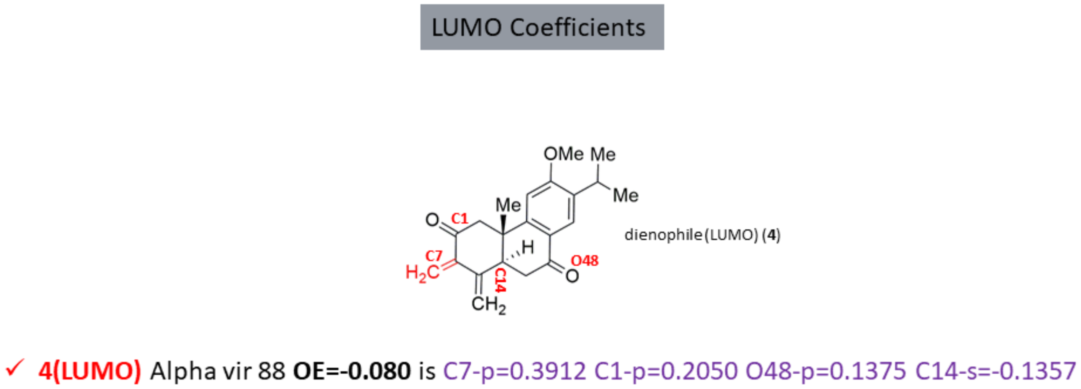

Figure S5. LUMO coefficients of molecule **4**.

In molecule **4**, the coefficients of the lowest unoccupied molecular orbital (LUMO) clearly represent that the C7 atom within the diene moiety contribute majorly 39% to the construction of the highest occupied molecular orbital for molecule **3**. Conversely, the C1, C14, and O48 atoms contribute 20.5% and 13.6%, 13.7% respectively, to the formation of the LUMO orbital.

[4+2]-DA reaction

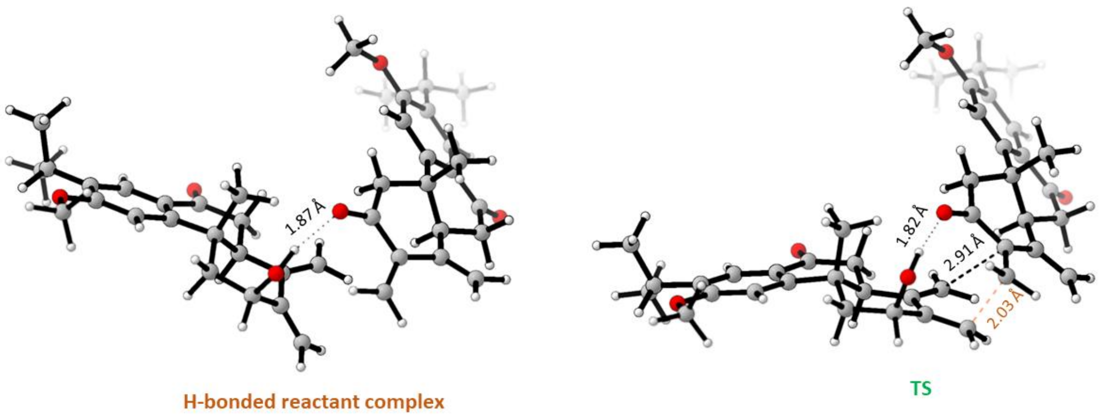

Figure S6. H-bonded reactant complex and TS for the [4+2]-mode of reaction.

Here we provide representations of the optimized geometries for both the H-bonded reactant complex and the transition state structure. In the H-bonded complex, the H-bonding distance is d(OH…O) = 1.87 Å, whereas in the transition state (TS), the H-bonding strengthens, evidenced by a reduction in distance to d(OH…O) = 1.82 Å. It is widely recognized that in catalyzed reactions, the TS structure tends to become asynchronous. In this instance, we have also observed a significant disparity in the bond-forming distances, with a difference of (2.91 Å – 2.03 Å) = 0.88 Å.

Interactions in TS

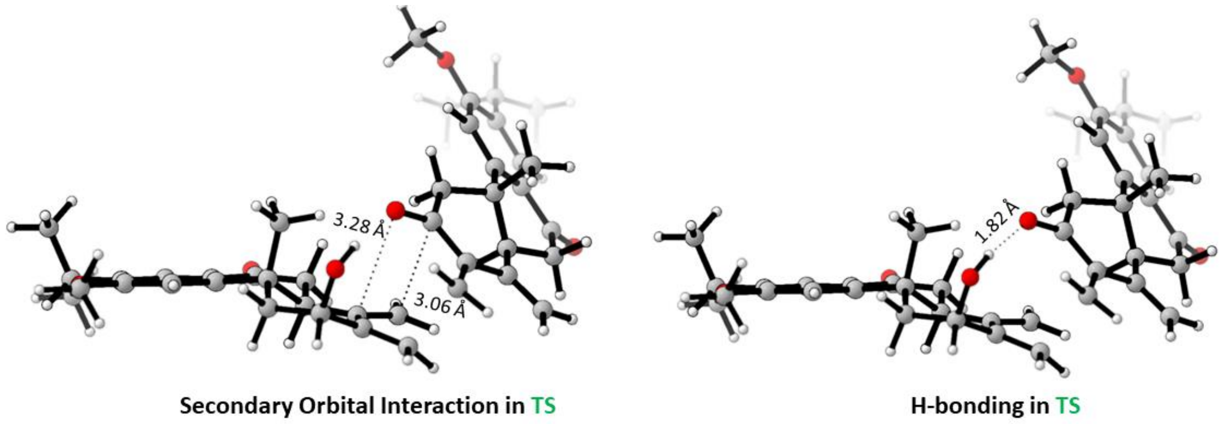

Figure S7. H-bonding and secondary orbital interaction in the TS geometry.

The artwork above illustrates the synergy between H-bonding interaction and secondary orbital interaction. It showcases the H-bonding distance d(OH…O) = 1.82 Å. Moreover, a secondary orbital interaction is observed between the carbonyl group of molecule **4** and the double bond of molecule **3**, with distances d(C…O) and d(C…C) measured at 3.28 and 3.06 Å, respectively.

Optimized Cartesian coordinates

3 --

C	-3.31422300	-1.67500800	0.44919800
C	-4.00776000	-0.39304300	0.86252800
C	-3.50300900	0.84538300	0.22432500
C	-4.32980800	1.74044500	-0.32831400
H	-3.97474300	2.66036300	-0.78010000
H	-5.40191000	1.56895300	-0.34834700
C	-5.01159000	-0.40468200	1.74786000
H	-5.51308900	0.50950300	2.05169600
H	-5.35026500	-1.33078000	2.20711100
C	-1.79483200	-1.52194700	0.35081500
H	-1.38966600	-1.51168400	1.37018300
H	-1.42073800	-2.42688700	-0.13805300
C	-1.29669300	-0.25299700	-0.38792000
C	-1.99258600	0.97902100	0.27150600
C	0.21907500	-0.10473800	-0.22767700
C	-1.45970300	2.29792500	-0.28035100
H	-1.70191200	2.39510400	-1.34717800
H	-1.91120000	3.15765800	0.22302200
C	0.04611600	2.41831800	-0.13287700
C	0.82621500	1.15886100	-0.11818400

C	1.04946900	-1.23138100	-0.24987300
C	2.22564600	1.25929800	-0.01163300
H	0.60867100	-2.21310200	-0.35150200
H	2.63430600	2.25990800	0.06886400
C	2.43641400	-1.11080200	-0.14423700
C	3.05865800	0.15426100	-0.01795400
O	3.28033800	-2.18121500	-0.15391400
C	2.73282700	-3.48136300	-0.30522100
H	3.58302200	-4.16500900	-0.29543300
H	2.05536000	-3.72930200	0.52163200
H	2.19434000	-3.58098000	-1.25602700
C	4.56816900	0.24312200	0.12928400
H	5.00078900	-0.51063100	-0.53988500
C	4.98730500	-0.12550300	1.56587400
H	4.63058100	-1.12357000	1.83545800
H	6.07918700	-0.11276200	1.66442500
H	4.57228700	0.59394300	2.28154500
C	5.13824600	1.60976500	-0.26641300
H	4.82355800	1.90227800	-1.27388400
H	4.82234000	2.39684500	0.42770500
H	6.23297400	1.57689700	-0.24592100
H	-3.53530900	-2.45591300	1.19297500
O	-3.78615000	-2.10072000	-0.83740300
C	-1.60316600	-0.33167000	-1.90137100
H	-1.17589400	-1.24894200	-2.31818700
H	-2.67766800	-0.34804600	-2.08625800
H	-1.15531400	0.51167300	-2.43566000
O	0.57936100	3.51744200	-0.04915000
H	-1.71267300	0.93654200	1.33687600
H	-4.75406000	-2.03570500	-0.81751400

4 ---

C	3.32298000	-1.64529000	-0.43594200
C	4.14580700	-0.39255100	-0.53928300
C	3.54750600	0.87953700	-0.06883100
C	4.30366800	1.81798400	0.51694000
H	3.89747200	2.76141000	0.86241800
H	5.36571000	1.66041700	0.67737800
C	5.36985800	-0.49331300	-1.08161500
H	5.99361300	0.37858100	-1.25140600
H	5.75265200	-1.46530200	-1.37492900
C	1.81294400	-1.48915500	-0.33479100
H	1.43313200	-1.46542400	-1.36693200
H	1.43706500	-2.40977000	0.11773400
C	1.32345300	-0.22393300	0.40428900
C	2.04499300	0.99759400	-0.24633800
C	-0.19259900	-0.07558300	0.25364800
C	1.46662000	2.32669600	0.23349600
H	1.63820100	2.45089000	1.31169600

H	1.94446600	3.17607800	-0.26196400
C	-0.02649200	2.43163600	-0.00993000
C	-0.80297800	1.17309400	0.05121600
C	-1.01848500	-1.20182000	0.35614600
C	-2.20256700	1.25963000	-0.07100600
H	-0.57405300	-2.17257600	0.52741400
H	-2.61438400	2.24993100	-0.22694300
C	-2.40519600	-1.09564100	0.23690000
C	-3.03116800	0.15503200	0.01322200
O	-3.24454500	-2.16438700	0.32274800
C	-2.69338200	-3.45022200	0.56447200
H	-3.54171000	-4.13506700	0.59952100
H	-2.01330900	-3.75172200	-0.24191900
H	-2.15782800	-3.48215000	1.52150900
C	-4.54001800	0.22646100	-0.14803700
H	-4.97324200	-0.47687000	0.57350500
C	-4.94889200	-0.25088100	-1.55541500
H	-4.58704700	-1.26479700	-1.74782300
H	-6.04013600	-0.25027300	-1.66019400
H	-4.53286100	0.41487800	-2.32066200
C	-5.11731700	1.61660400	0.14059300
H	-4.80978800	1.98560100	1.12491300
H	-4.80099400	2.35044600	-0.60940500
H	-6.21165400	1.57725700	0.11688400
C	1.65999500	-0.34762600	1.90662700
H	1.15567600	-1.22102000	2.33158800
H	2.73701500	-0.46009000	2.06695000
H	1.32409100	0.53105300	2.46492900
O	-0.55369400	3.51733700	-0.21370500
H	1.83701900	0.92353800	-1.32606200
O	3.82680300	-2.75476600	-0.48733900

H-bonded Reactant Complex ---

O	0.51757000	-2.61222500	1.77605800
C	1.60179100	-2.47224800	1.21686500
C	2.25778100	-3.57859200	0.46262400
C	-2.33001600	-3.61790600	-0.40694600
C	-1.28214800	-3.21738200	-1.43546800
C	-0.90162500	-1.78574500	-1.50978200
C	3.67026700	-3.39456000	0.04845100
C	1.52517300	-4.66754500	0.16214500
H	0.49733500	-4.76572900	0.49279100
H	1.93737000	-5.46436900	-0.44917200
C	0.37246100	-1.41823400	-1.71034400
H	0.67178100	-0.38234100	-1.82606900
H	1.15780800	-2.16364000	-1.77747000
C	2.25469700	-1.10423600	1.23683800
H	1.98151500	-0.64605900	2.19128400

H	1.72972100	-0.53337600	0.45856800
C	3.77398700	-1.04764700	0.97523500
C	-0.75548400	-4.13488100	-2.25763000
H	-0.03187300	-3.86997500	-3.02289100
H	-1.04658900	-5.18017300	-2.19257900
C	4.07698300	-1.96435000	-0.25044400
H	3.42595400	-1.59977000	-1.06075900
C	-3.42740900	-2.55151400	-0.25147500
H	-4.10665400	-2.63564400	-1.11058000
H	-3.99666100	-2.83058500	0.64011800
C	-2.95449800	-1.08147600	-0.16613200
C	-2.05839300	-0.80511000	-1.41207200
C	4.51285100	-4.43477000	-0.00563500
H	5.54369300	-4.33254000	-0.32369300
H	4.19120100	-5.43201900	0.27840500
C	-4.15493800	-0.13042700	-0.20602300
C	-1.64099900	0.66061900	-1.49556400
H	-0.97674300	0.91761000	-0.65887300
H	-1.08289500	0.87224000	-2.41204200
C	-2.82299300	1.61388100	-1.46132000
C	-4.06221800	1.13760000	-0.80734100
C	4.19576000	0.38915200	0.65852600
C	5.51920100	-1.81592900	-0.72901600
H	5.71100200	-2.41993400	-1.62006400
H	6.21693900	-2.16462000	0.04450400
C	5.88097800	-0.38000300	-1.05929100
C	5.18168300	0.67944300	-0.29877800
C	3.61584600	1.45852300	1.35167000
C	5.54399500	2.01614800	-0.55015600
H	2.86251800	1.26052600	2.10168600
H	6.30968300	2.17748100	-1.30000700
C	3.98681300	2.77833100	1.08666800
C	4.97128500	3.08506900	0.11567400
C	-5.35875900	-0.47248100	0.41965900
C	-5.16691500	2.00881700	-0.78622300
H	-5.45182200	-1.43599500	0.89991000
H	-5.03744700	2.96752000	-1.27501000
C	-6.44155200	0.40930700	0.43686600
C	-6.36697500	1.68111100	-0.17996800
O	3.43964700	3.84824600	1.72513900
O	-7.63105800	0.11585900	1.03337900
C	-7.78902600	-1.14884200	1.65786400
H	-8.80656600	-1.16086600	2.05116400
H	-7.66479500	-1.96831000	0.93900700
H	-7.07706400	-1.27998300	2.48232100
C	2.43467100	3.62333400	2.70253200
H	1.56041100	3.12305200	2.26780000
H	2.81707400	3.02546800	3.53932800
H	2.14563400	4.61108400	3.06373700
C	-7.55872100	2.62154500	-0.12063800

C	5.32210900	4.53475900	-0.17210800
C	4.52555100	-1.52320700	2.23752000
H	4.28693300	-0.86847500	3.08151500
H	4.24524100	-2.54738900	2.50448200
H	5.60953500	-1.49411600	2.09474800
H	-2.82021800	-4.53089000	-0.76067500
O	-1.76044200	-4.00336500	0.84418800
H	-1.05975000	-3.38320400	1.12061600
C	-2.19426400	-0.80195300	1.15094200
H	-2.77775900	-1.16158000	2.00407900
H	-1.21606300	-1.28422200	1.18378900
H	-2.04176000	0.27284700	1.29015900
O	-2.72874300	2.73421800	-1.94740800
O	6.73327400	-0.12860900	-1.90096500
H	-2.70237200	-1.01064000	-2.28282700
H	-8.45860700	2.00715500	-0.24624600
C	-7.64749900	3.28500000	1.26761900
C	-7.55176500	3.68188800	-1.22746200
H	-8.48524200	4.25445700	-1.20104000
H	-6.72850300	4.39417700	-1.10104000
H	-7.45634000	3.22875800	-2.22003200
H	-7.70806600	2.53300700	2.05936800
H	-6.76395200	3.90785700	1.45058300
H	-8.53512400	3.92522200	1.33329000
H	5.32128000	5.06200200	0.78958500
C	6.70594300	4.71050000	-0.80723800
C	4.23330200	5.18131700	-1.05096100
H	3.24977200	5.10754700	-0.57865600
H	4.45428500	6.24173900	-1.21918500
H	4.18470700	4.68458800	-2.02701700
H	6.74359500	4.29193000	-1.81939300
H	6.94599100	5.77619500	-0.88645500
H	7.48832100	4.22713600	-0.21228200

TS --

O	-0.26591400	2.28460100	1.84503600
C	-1.27927600	2.25281300	1.12332300
C	-1.66676900	3.38242800	0.29717800
C	2.62364700	3.52003400	0.35479300
C	1.46904100	3.37916400	-0.62477800
C	1.10425600	2.08112100	-1.13007600
C	-2.83890000	3.29212400	-0.58959900
C	-0.87433600	4.53225700	0.43413000
H	-0.25714000	4.60184600	1.32085500
H	-1.25638900	5.47981200	0.06881700
C	-0.12791700	1.90163100	-1.68558600
H	-0.46822100	0.92698500	-2.01234300
H	-0.78670400	2.72437800	-1.91618800

C	-2.13210600	0.99998300	1.14895500
H	-2.10375700	0.63907100	2.18103000
H	-1.62317400	0.24030700	0.54194500
C	-3.57577200	1.18586200	0.64059400
C	0.68594100	4.51061500	-0.85923400
H	0.06930500	4.55604000	-1.75233700
H	1.10360700	5.46616700	-0.55286300
C	-3.48114600	1.91478700	-0.73110200
H	-2.79475500	1.30422000	-1.33722400
C	3.65065300	2.38676400	0.26484600
H	4.28794700	2.56982700	-0.61170300
H	4.28369300	2.48444300	1.15180900
C	3.07636000	0.96187000	0.14939400
C	2.12898700	0.94557500	-1.08345600
C	-3.29892700	4.35767300	-1.27168000
H	-4.15275400	4.29739100	-1.93346200
H	-2.84437900	5.33856100	-1.18555000
C	4.19227200	-0.05603500	-0.10689600
C	1.53413300	-0.44602800	-1.30089000
H	0.82967600	-0.69462800	-0.49566800
H	0.97299000	-0.51454700	-2.23663300
C	2.58889000	-1.54252400	-1.36676400
C	3.92316900	-1.24925200	-0.80105000
C	-4.24904000	-0.17746700	0.47133800
C	-4.82245000	1.93285900	-1.46474500
H	-4.72981300	2.35726300	-2.46739600
H	-5.54625900	2.55811500	-0.92414700
C	-5.43170000	0.55326400	-1.63175500
C	-5.11135500	-0.45574700	-0.60077600
C	-4.04046000	-1.18224800	1.42419300
C	-5.71807200	-1.72161200	-0.70654500
H	-3.39049600	-0.98876100	2.26632400
H	-6.37265600	-1.87558500	-1.55648700
C	-4.65131300	-2.43155300	1.30366000
C	-5.51173700	-2.72914200	0.21845600
C	5.47831900	0.12765300	0.40927200
C	4.93551000	-2.21120100	-0.96764400
H	5.70828900	1.03065000	0.95623900
H	4.66931900	-3.10763600	-1.51572000
C	6.47075200	-0.84068400	0.23526400
C	6.21570500	-2.04163700	-0.46877300
O	-4.46855900	-3.43980600	2.20018000
O	7.73700900	-0.70265600	0.71905300
C	8.07488400	0.48220400	1.42353100
H	9.12400300	0.37305200	1.70217500
H	7.95462800	1.37124900	0.79192400
H	7.46633400	0.59684800	2.32922500
C	-3.61916200	-3.21823600	3.31580300
H	-2.59418400	-2.98979000	2.99824700
H	-3.99339400	-2.40318200	3.94790700

H	-3.62486300	-4.15000700	3.88297200
C	7.31410700	-3.08139900	-0.61288900
C	-6.12844800	-4.11243100	0.10107000
C	-4.38884500	2.01103500	1.66294200
H	-4.43231900	1.48299200	2.62076200
H	-3.93653900	2.99320500	1.83234300
H	-5.41765800	2.16215900	1.32314400
H	3.15694200	4.44949400	0.11941300
O	2.13132700	3.71554800	1.67938300
H	1.32569200	3.16874200	1.79850400
C	2.36507900	0.52111700	1.45094100
H	3.01748800	0.71270100	2.30870700
H	1.42169500	1.04085400	1.61563600
H	2.16035300	-0.55369300	1.43338000
O	2.31767400	-2.61968700	-1.88262100
O	-6.17601700	0.31422500	-2.57429100
H	2.78790600	1.14096000	-1.94558500
H	8.25054400	-2.53702300	-0.78547800
C	7.47849200	-3.87039400	0.70082100
C	7.09719300	-4.03519400	-1.79300200
H	7.97097000	-4.68488400	-1.91258500
H	6.22782900	-4.68363900	-1.63543200
H	6.94581200	-3.48963200	-2.73063600
H	7.68844000	-3.19945500	1.53854300
H	6.56304300	-4.42926300	0.92798000
H	8.30407500	-4.58725000	0.61960700
H	-6.40200100	-4.42884400	1.11499100
C	-7.39482100	-4.14215700	-0.76185500
C	-5.08608700	-5.11738100	-0.42744900
H	-4.20132400	-5.14162300	0.21479700
H	-5.51026000	-6.12754900	-0.46571400
H	-4.76892400	-4.84245100	-1.44016600
H	-7.17289400	-3.93215500	-1.81433700
H	-7.85202300	-5.13650100	-0.71736800
H	-8.13434900	-3.41009400	-0.41994600

6 --

O	-0.73777600	0.58666100	0.80322200
O	-7.23736000	0.37855000	2.85773900
O	-5.70439500	-2.52847200	-2.52982600
O	2.15167000	3.46442400	1.50902700
C	-1.46080800	1.03328700	-0.06922000
C	-6.72629100	-1.64550600	-0.07709800
H	-7.12862300	-2.52120400	-0.57293200
C	-5.08087000	0.14543200	-0.13773900
C	-2.90092500	1.41263800	0.24438500
H	-2.94632400	2.50650500	0.32776500
H	-3.10384900	1.00615300	1.23572500

C	-5.21328600	-1.52477200	-2.03112100
C	-0.98210000	1.25968200	-1.50932400
C	-7.25949700	-1.20446500	1.12094600
C	0.37028500	0.55386900	-1.72719700
H	0.27206200	-0.47161600	-1.36159100
H	0.55299400	0.46996600	-2.80674200
C	-8.38755100	-1.91708700	1.84723300
H	-9.03696900	-1.14133100	2.27086100
C	-6.68454000	-0.03934500	1.68565800
C	1.48544500	2.47555400	-0.59048400
C	-5.62189700	0.61828900	1.06396000
H	-5.20618700	1.50923500	1.51688900
C	-4.56403700	2.11023300	-1.55881700
H	-5.33123300	1.78544700	-2.26749000
H	-3.80612600	2.67057500	-2.11360900
H	-5.04474400	2.78441500	-0.84283900
C	-5.65944700	-0.99221700	-0.72271200
C	-3.18438800	-0.03481300	-1.80108000
H	-2.73024200	-0.81765400	-1.16960000
C	1.55333800	1.21710100	-1.05516300
C	-4.13728700	-0.74120500	-2.76048000
H	-4.64137600	-0.01280200	-3.41017500
H	-3.60389800	-1.43313400	-3.41783000
C	-2.04654300	0.70935000	-2.46770200
C	2.84266100	0.40125200	-0.96074500
H	3.46115300	0.67625100	-1.83142900
C	-7.82754400	-2.73943700	3.02451600
H	-7.16089700	-3.52829000	2.65700800
H	-8.64195900	-3.21467500	3.58365100
H	-7.26289300	-2.10581600	3.71420500
C	0.24269800	3.32765600	-0.70103700
H	0.53069800	4.34826500	-0.98787300
H	-0.21055000	3.43032700	0.29443200
C	-3.94069400	0.90901200	-0.81279400
C	2.60679500	3.12332100	0.18450600
C	3.89990800	2.30395200	0.21756400
H	4.47891700	2.67318300	1.06880000
H	4.48313800	2.51638800	-0.68828500
C	5.00539100	0.02665700	0.26956800
C	-0.77358700	2.78470900	-1.70066200
H	-0.41187900	2.94278000	-2.72232500
H	-1.72438100	3.32064700	-1.61689500
C	-9.24073800	-2.79898100	0.92888200
H	-9.62086000	-2.23797800	0.06839500
H	-10.09768700	-3.19474200	1.48445000
H	-8.67377300	-3.65668600	0.54956200
C	7.33756700	-0.17211400	0.94062700
C	3.67579800	0.78197500	0.29352700
C	-1.94998800	0.87868000	-3.78813900
C	2.93394600	0.38215900	1.59567900

H	2.99809700	-0.69599900	1.76754400
H	1.87040000	0.63762000	1.54902200
H	3.39098300	0.87574200	2.45955100
C	6.13502100	0.53793200	0.91724600
H	6.07626600	1.49831200	1.40940000
C	7.46276400	-1.43214500	0.30813700
C	-6.71790200	1.54210000	3.48265900
H	-5.66074500	1.41511900	3.74750500
H	-7.30623400	1.67942000	4.39091400
H	-6.82752800	2.42442400	2.83956400
C	2.64578400	-1.11722700	-1.04265900
H	2.03453800	-1.47393100	-0.20252300
H	2.12885700	-1.42123100	-1.95583100
O	8.46210600	0.28813200	1.55818400
C	6.33352500	-1.92841900	-0.31995800
H	6.35577300	-2.88915200	-0.82128400
O	4.04639900	-2.98298800	-1.53931300
C	3.96247300	-1.87613300	-1.02159900
C	8.94830600	-3.21802900	-0.74431300
H	8.23963100	-4.04728100	-0.63879900
H	9.95669600	-3.64455400	-0.71026800
H	8.79920800	-2.77059200	-1.73286500
C	5.11142700	-1.23249100	-0.34634600
C	8.78126000	-2.18408500	0.37484100
H	9.58048100	-1.44016200	0.27036000
C	8.94964000	-2.84613900	1.75638200
H	8.88639200	-2.10461500	2.55773700
H	9.92210800	-3.34739900	1.82810600
H	8.16683900	-3.59641000	1.91850100
C	8.42003200	1.55284300	2.20026400
H	7.68590600	1.56386000	3.01566600
H	8.18321500	2.35479900	1.48987600
H	9.41901800	1.71203800	2.60889200
H	-2.68343700	0.47912500	-4.47938200
H	-1.11975600	1.41685500	-4.23641500
H	2.83483500	4.10147600	-0.25763400
H	1.76395000	2.65944200	1.88684700

27 --

O	-0.70911100	0.44013300	0.86642000
O	-7.20283500	-0.04078200	2.92576700
O	-5.72370400	-2.11044400	-2.84972000
O	2.58109700	4.14952900	1.09980900
C	-1.44270500	1.02187700	0.08810900
C	-6.72464900	-1.60163900	-0.28424000
H	-7.13637200	-2.39021800	-0.90323200
C	-5.06763000	0.16857000	-0.08632300
C	-2.87762400	1.34939500	0.47001900

H	-2.92033700	2.41648000	0.72485800
H	-3.07421100	0.79186800	1.38636300
C	-5.22318900	-1.19643200	-2.20839500
C	-0.97564900	1.46499700	-1.30423000
C	-7.24688600	-1.34338600	0.97051600
C	0.37346000	0.79310900	-1.62198700
H	0.27575800	-0.27597000	-1.42028700
H	0.56764900	0.87750200	-2.69990400
C	-8.37498600	-2.15124300	1.58914000
H	-9.01690900	-1.44465500	2.12917700
C	-6.66048000	-0.27999200	1.70032300
C	1.49447300	2.54603700	-0.24462600
C	-5.59753500	0.45795800	1.17688500
H	-5.17255800	1.26783600	1.75588900
C	-4.54528900	2.32242000	-1.19780000
H	-5.31845800	2.11287300	-1.94253900
H	-3.78733900	2.95667700	-1.66606600
H	-5.01783700	2.88308900	-0.38510600
C	-5.65758900	-0.86454300	-0.83189300
C	-3.18226200	0.23002600	-1.77078500
H	-2.72926000	-0.64128200	-1.26728500
C	1.55736900	1.34635700	-0.86495300
C	-4.14565700	-0.31861800	-2.81874300
H	-4.64766500	0.50232500	-3.34861400
H	-3.62119800	-0.90786300	-3.57579800
C	-2.04323300	1.05804800	-2.32858900
C	2.84321100	0.52584600	-0.87559500
H	3.45923400	0.91645700	-1.70357600
C	-7.81286400	-3.14443200	2.62523300
H	-7.15364100	-3.87254000	2.13833900
H	-8.62677500	-3.69406100	3.11224800
H	-7.23987300	-2.62503600	3.39845800
C	0.25543100	3.40484900	-0.21611900
H	0.54597800	4.45272100	-0.34205700
H	-0.18533100	3.35123100	0.78988100
C	-3.92593400	1.01861800	-0.64617700
C	2.64243900	3.07272400	0.51684400
C	3.91948400	2.25269200	0.54607600
H	4.43960900	2.52154600	1.46885700
H	4.55095400	2.59152400	-0.28889300
C	4.99980100	-0.02446800	0.29968800
C	-0.76803500	3.00247800	-1.27281800
H	-0.41877400	3.30891800	-2.26486700
H	-1.71901800	3.51676600	-1.10367400
C	-9.23914600	-2.88036300	0.55436700
H	-9.62094000	-2.19424800	-0.20926800
H	-10.09518200	-3.35070100	1.04993800
H	-8.67980600	-3.67421500	0.04645300
C	7.32974300	-0.32012000	0.94083200
C	3.68062500	0.73820800	0.41729300

C	-1.94710900	1.41277100	-3.61169600
C	2.93652600	0.21772600	1.67088100
H	2.84633300	-0.87196200	1.65375900
H	1.93077400	0.63948100	1.74807600
H	3.50132000	0.48393700	2.57027900
C	6.13279100	0.39688200	1.00380100
H	6.08099300	1.29192400	1.60770000
C	7.44487600	-1.49597900	0.16027600
C	-6.67193800	1.01224100	3.71576800
H	-5.61396100	0.84101900	3.95059000
H	-7.25354700	1.01359400	4.63857800
H	-6.78031000	1.98215800	3.21424700
C	2.63762400	-0.96717800	-1.15827700
H	2.01104700	-1.42261000	-0.37982800
H	2.13130500	-1.14253900	-2.11025900
O	8.45700500	0.05340600	1.60752000
C	6.31301400	-1.90276800	-0.52407700
H	6.32868600	-2.79497900	-1.13923100
O	4.01755500	-2.77498400	-1.87077700
C	3.94587400	-1.73951300	-1.22103700
C	8.91727500	-3.15330400	-1.10041500
H	8.20226400	-3.98384800	-1.09614200
H	9.92227100	-3.58861400	-1.11787700
H	8.77312100	-2.58852000	-2.02773900
C	5.09634100	-1.19834400	-0.46636000
C	8.75722700	-2.26100500	0.13554500
H	9.56221200	-1.51614700	0.12180800
C	8.91876000	-3.08635500	1.42712100
H	8.86044300	-2.44683000	2.31234400
H	9.88732300	-3.59989900	1.43769400
H	8.13021900	-3.84478200	1.49724500
C	8.42541200	1.23159800	2.39890900
H	7.69155500	1.14874300	3.21027200
H	8.19527700	2.11552600	1.79121700
H	9.42570200	1.33152100	2.82255200
H	-2.68306800	1.11911000	-4.35155400
H	-1.11590700	2.00736100	-3.98009900

References:

1. D. Vrubliauskas, C. D. Vanderwall, *Angew. Chem. Int. Ed.*, 2020, **59**, 6115 –6121.
2. L. Y. Ke, Y. Zhang, M. Y. Xia, J. X. Zhuo, Y. H. Wang, C. L. Long, *J. Nat. Prod.* 2018, **81**, 418.
3. M. Frisch, G. Trucks, H. Schlegel, G. Scuseria, M. Robb, J. Cheeseman, G. Scalmani, V. Barone, G. Petersson, H. Nakatsuji, Gaussian 16, Revision A. 03, Gaussian, Inc., Wallingford CT. 2016.
4. A. V. Marenich, C. J. Cramer, D. G. Truhlar, *J. Phys. Chem. B*, 2009, **113**, 6378–6396.
5. K. Fukui, *J. Phys.Chem.*, 1970, **74**, 4161.
6. K. R. Cousins, Computer review of ChemDraw ultra 12.0. *ACS Publications*, 2011.
7. http://www.chemcraftprog.com
8. C. Y. Legault, CYLview, 1.0b; *Université de Sherbrooke*, 2009. www.cylview.org
